# Supplementary material for: Formal enantioconvergent substitution of alkyl halides via catalytic asymmetric photoredox radical coupling
Source: Nat Commun. 2018 Jun 22;9:2445. doi: 10.1038/s41467-018-04885-3 (PMC6015005; doi:10.1038/s41467-018-04885-3)
Supplement: Supplementary file 1 — Supplementary Information [file 41467_2018_4885_MOESM1_ESM.pdf]

Supporting Information

Enantioconvergent Formal Substitution of Alkyl Halides via Catalytic Asymmetric Photoredox Radical Coupling

Li et al.

Supplementary Figures

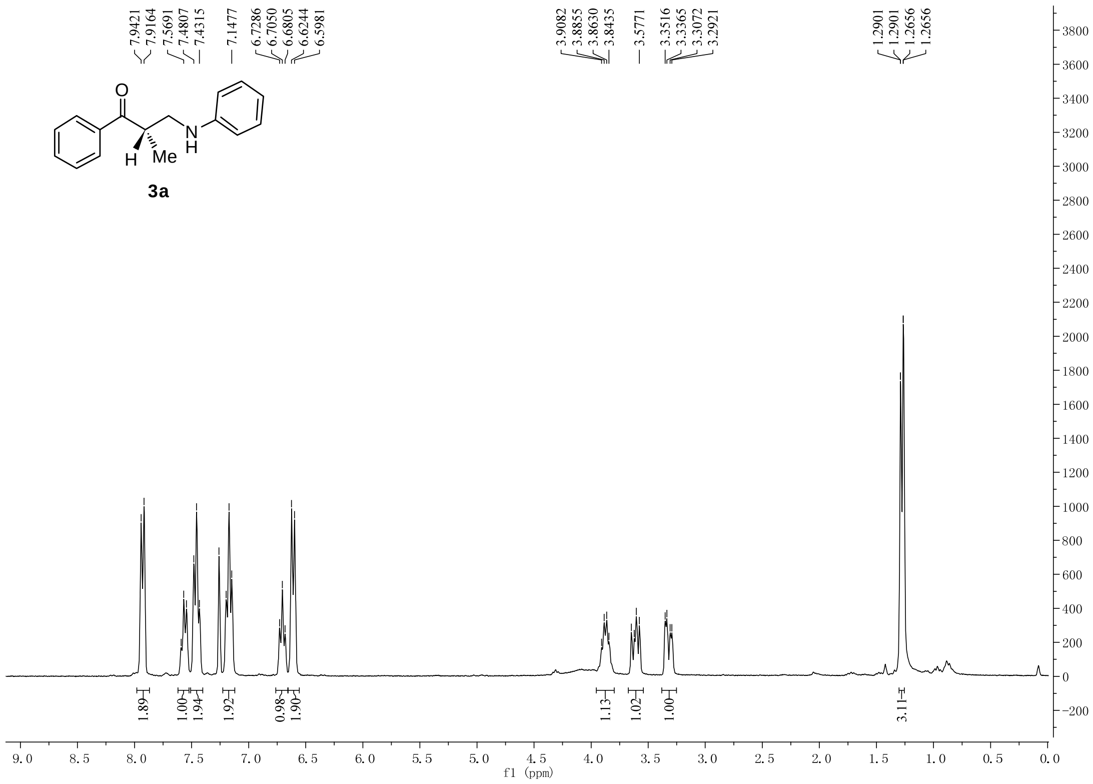

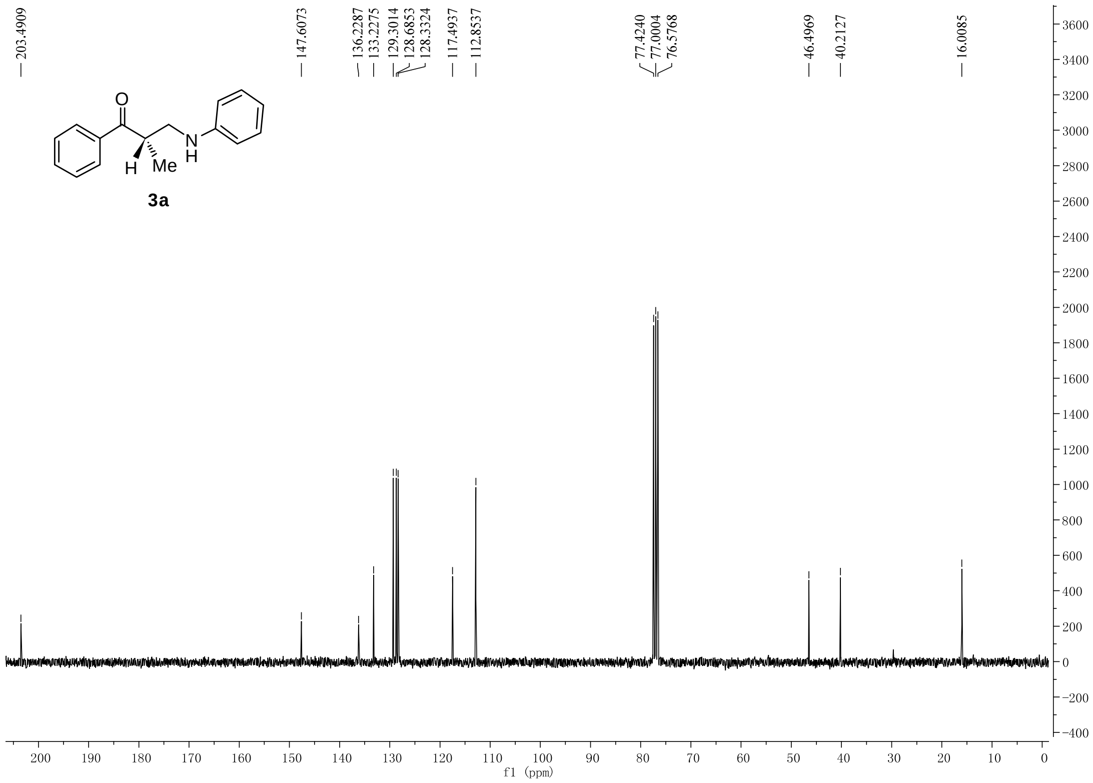

Supplementary Figure 1. ^{1}H and ^{13}C NMR spectra for compound **3a**

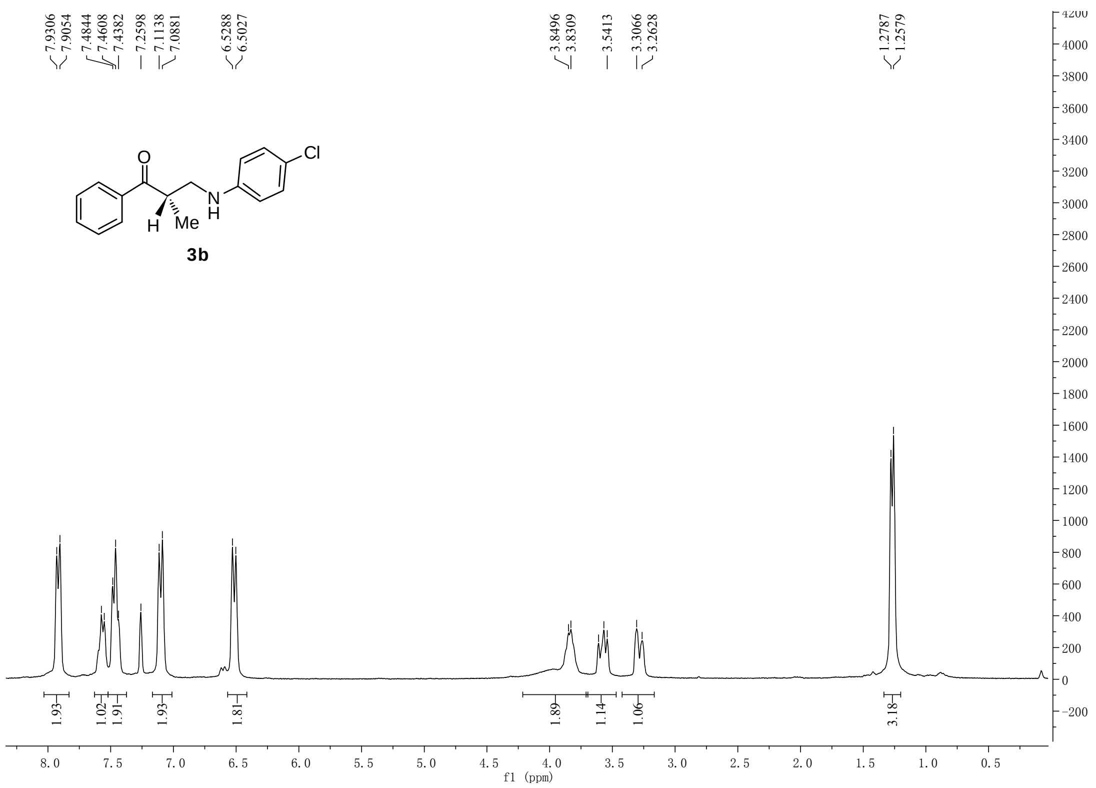

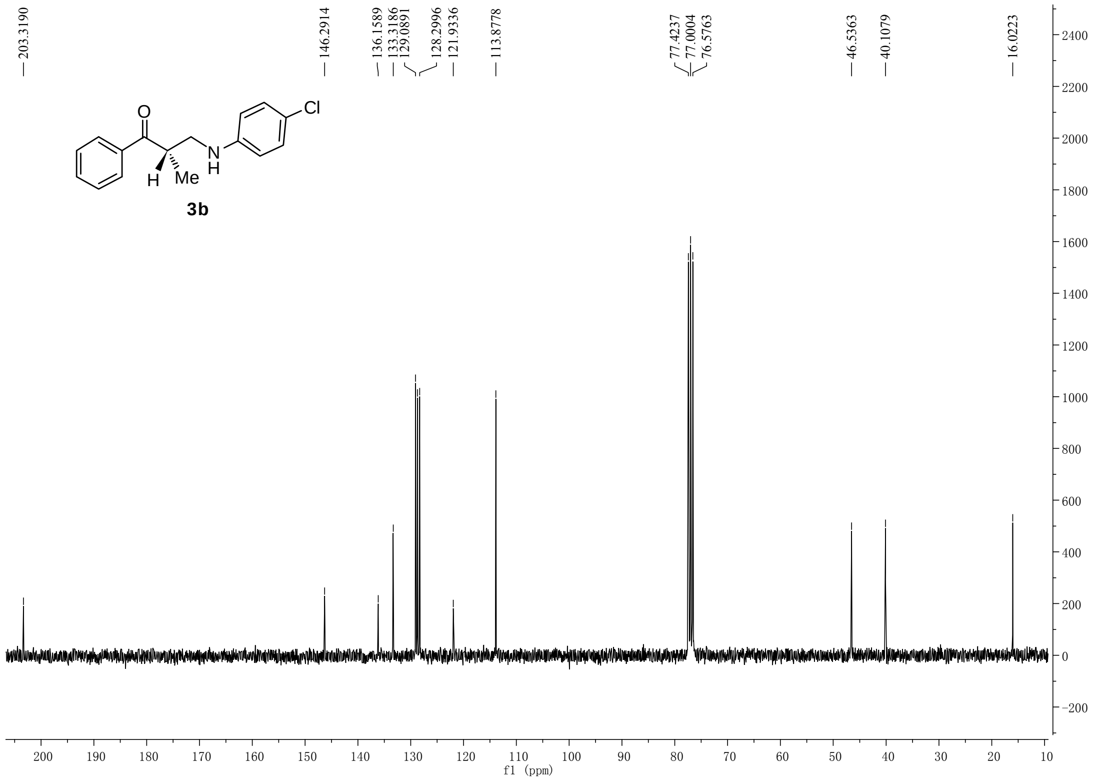

Supplementary Figure 2. ^{1}H and ^{13}C NMR spectra for compound **3b**

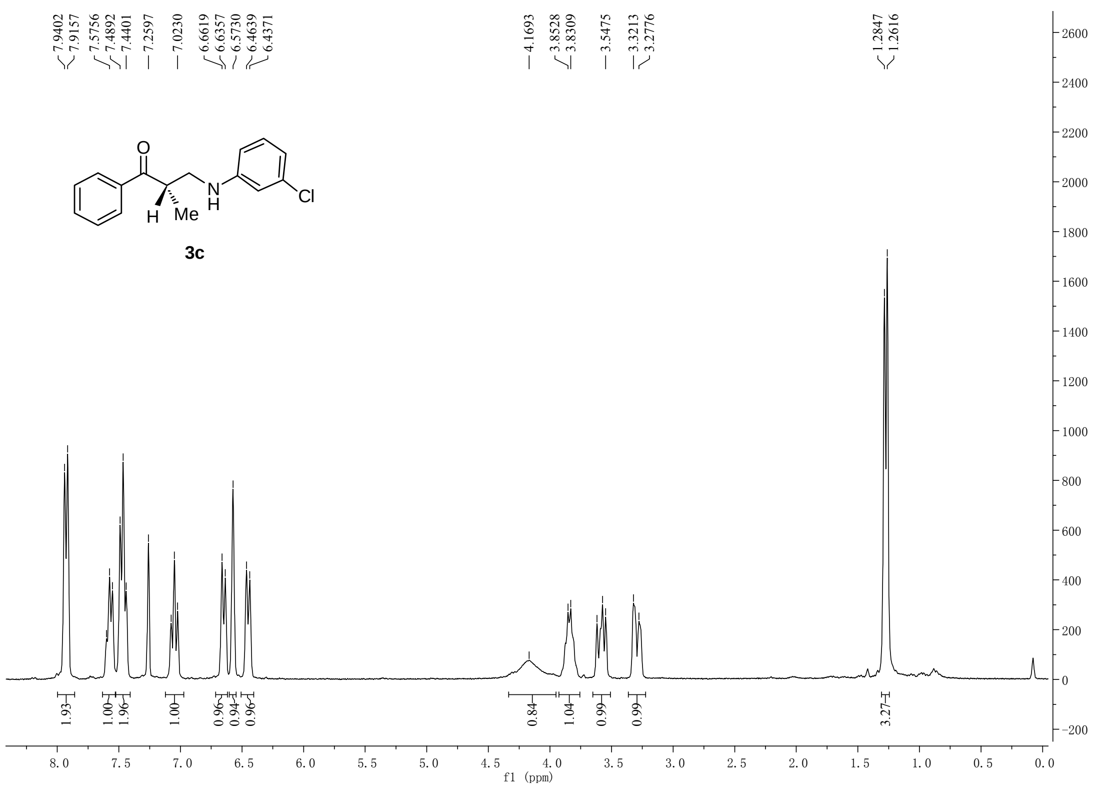

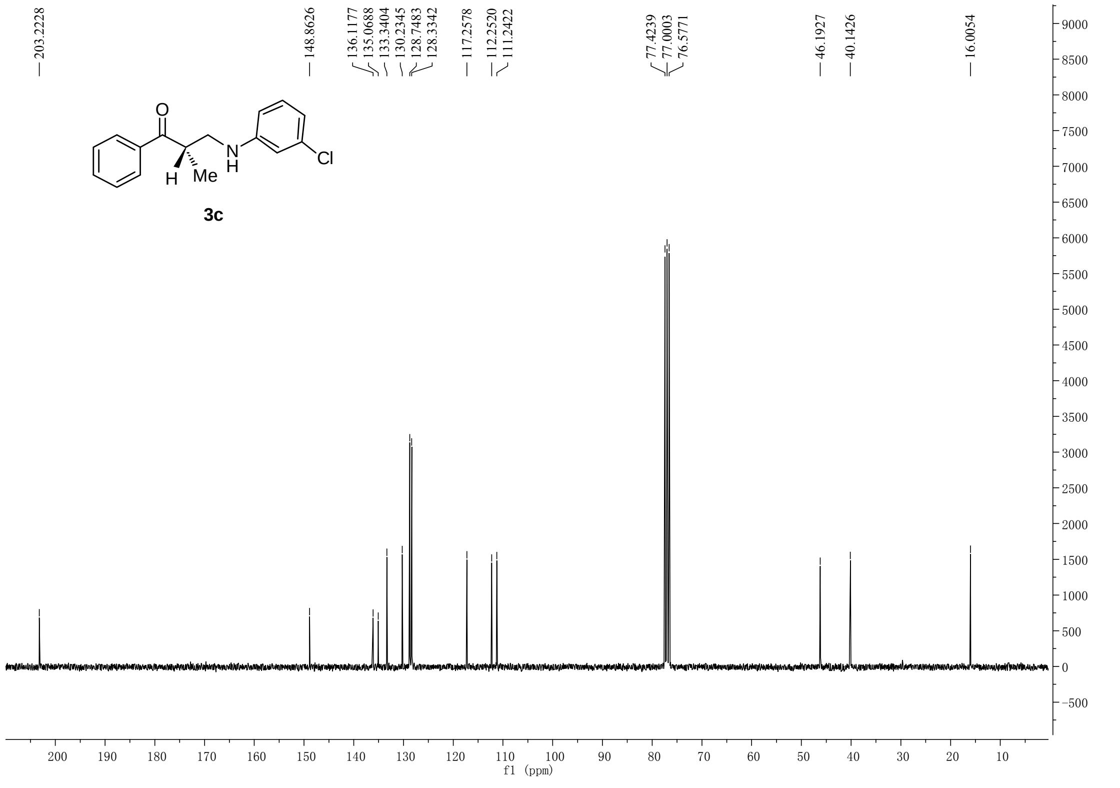

Supplementary Figure 3. ^{1}H and ^{13}C NMR spectra for compound **3c**

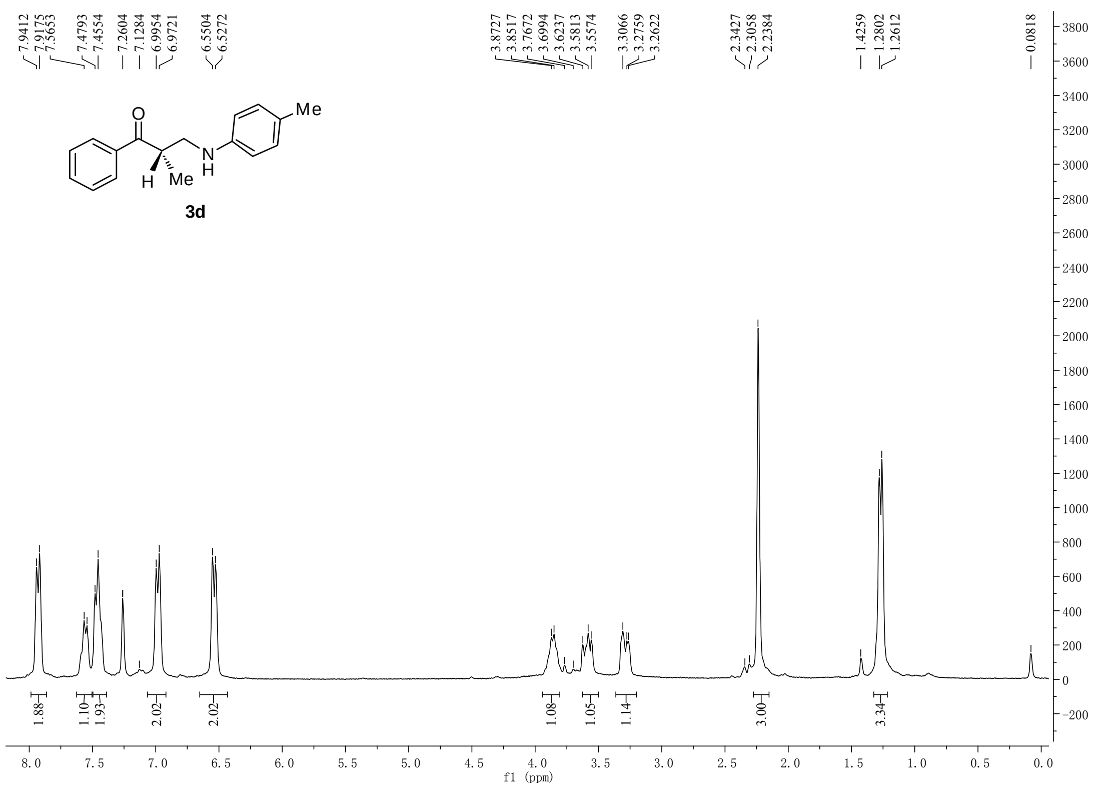

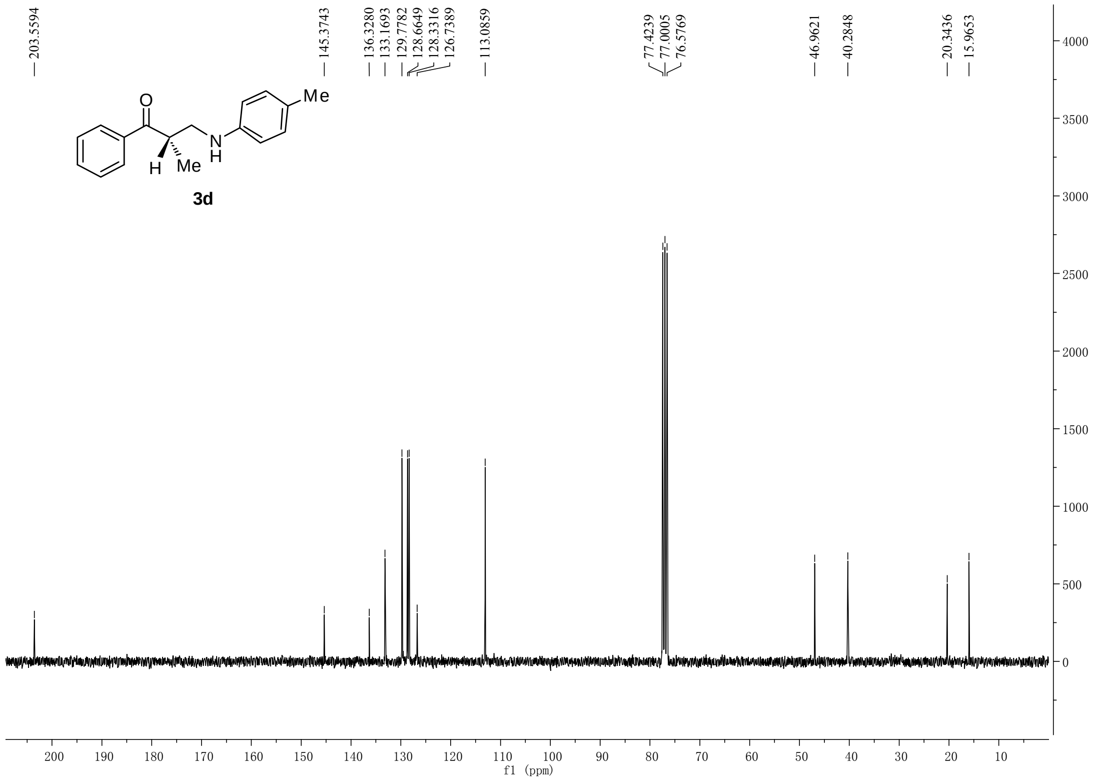

Supplementary Figure 4. ^{1}H and ^{13}C NMR spectra for compound **3d**

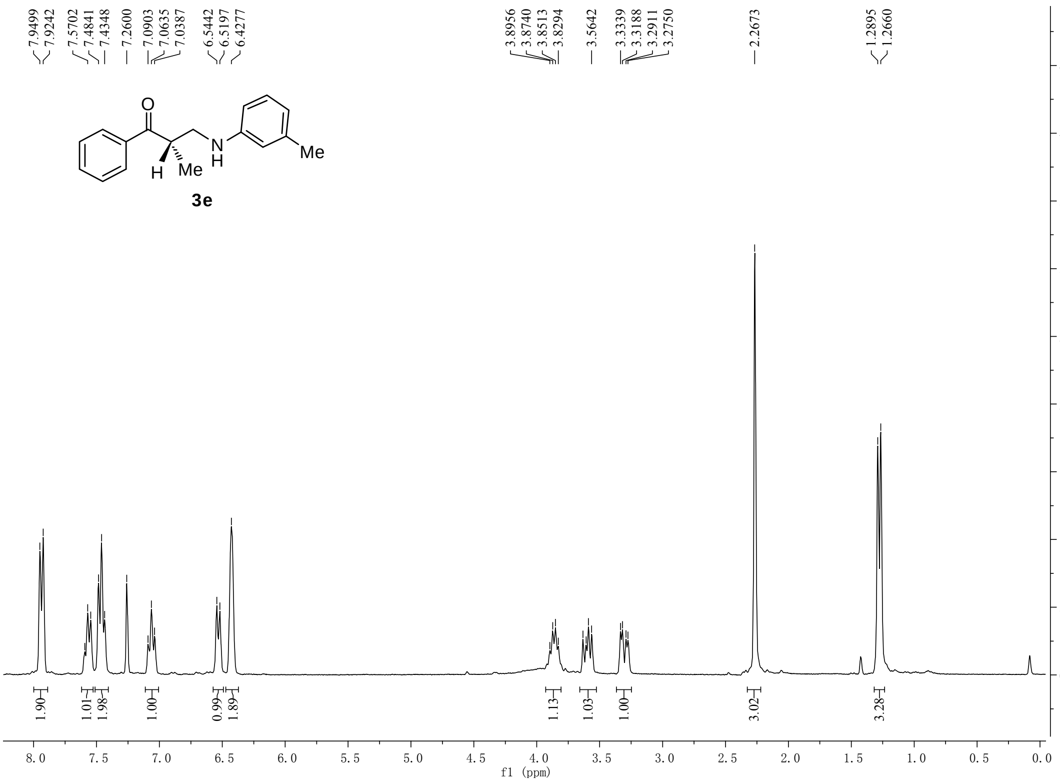

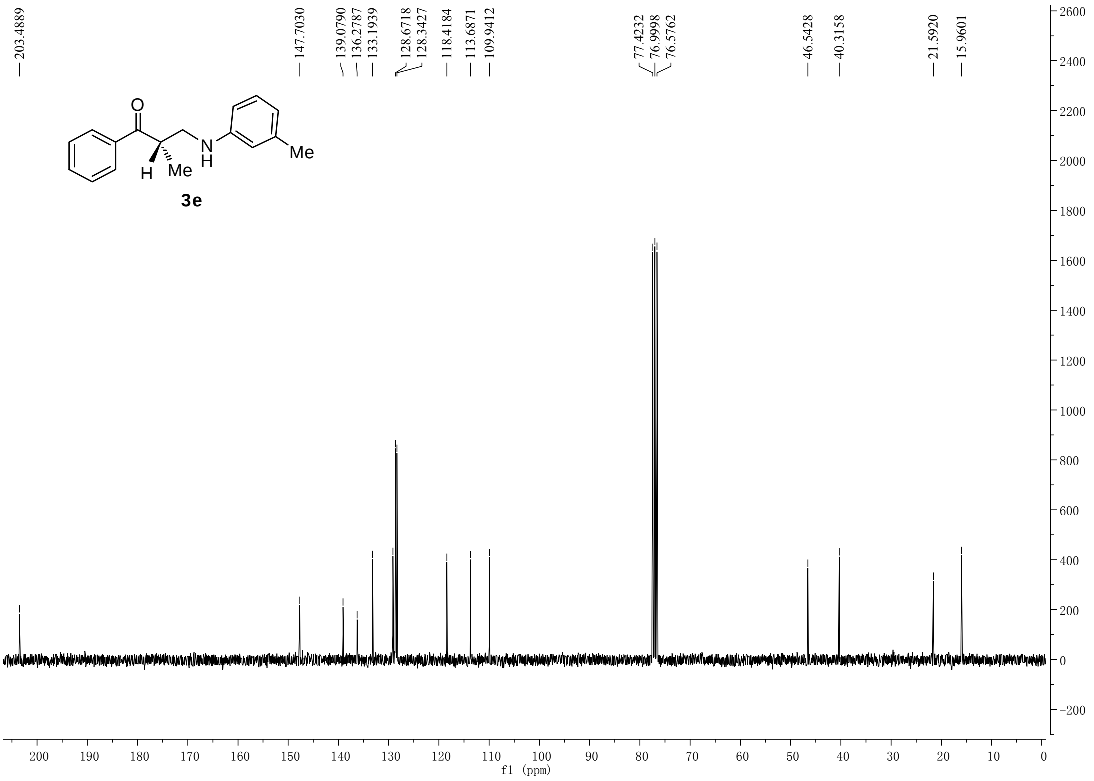

Supplementary Figure 5. ^{1}H and ^{13}C NMR spectra for compound **3e**

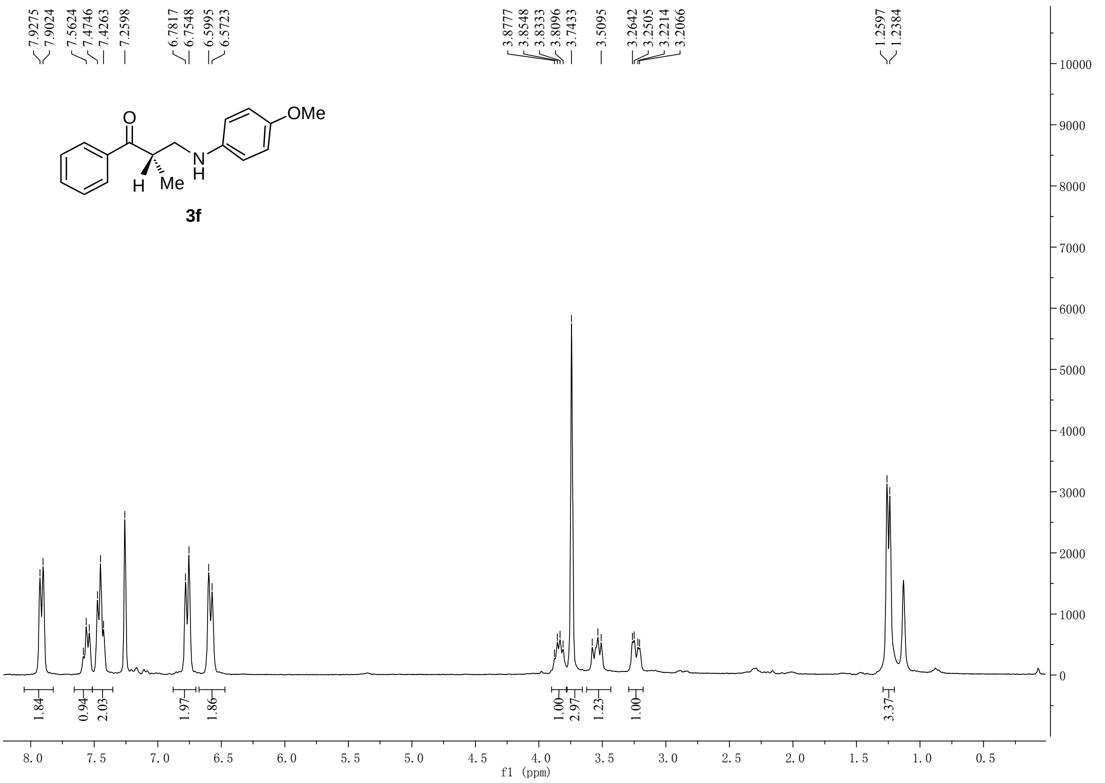

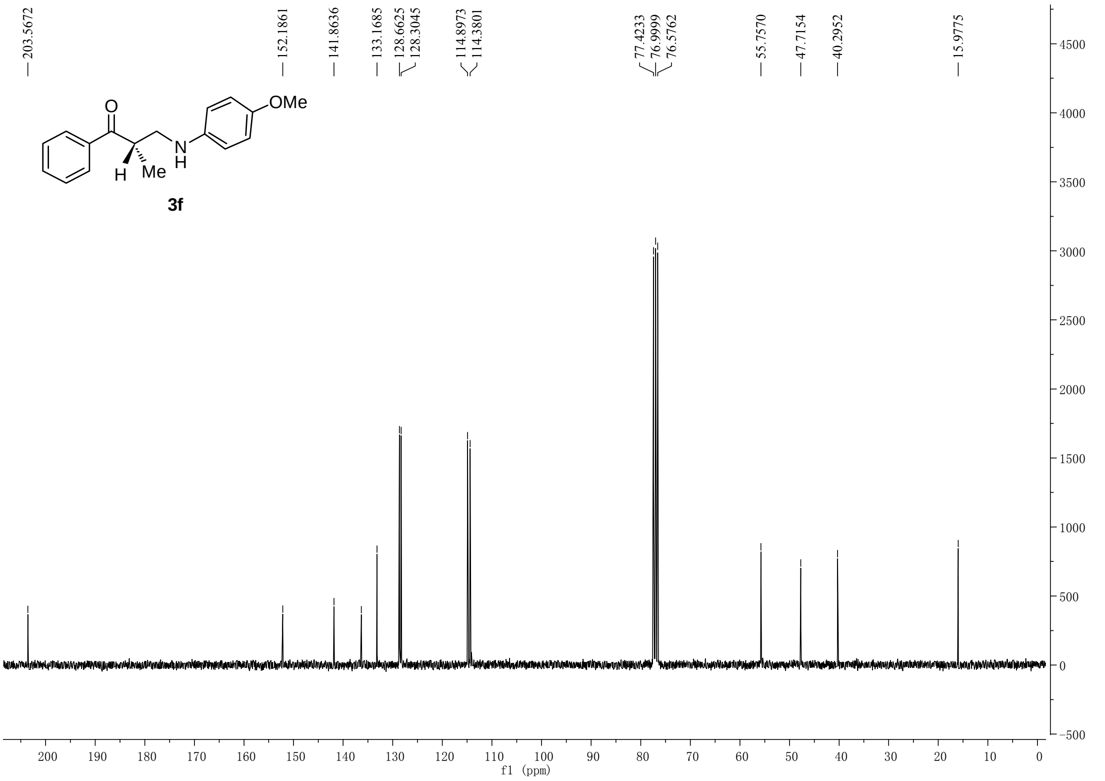

Supplementary Figure 6. ^{1}H and ^{13}C NMR spectra for compound **3f**

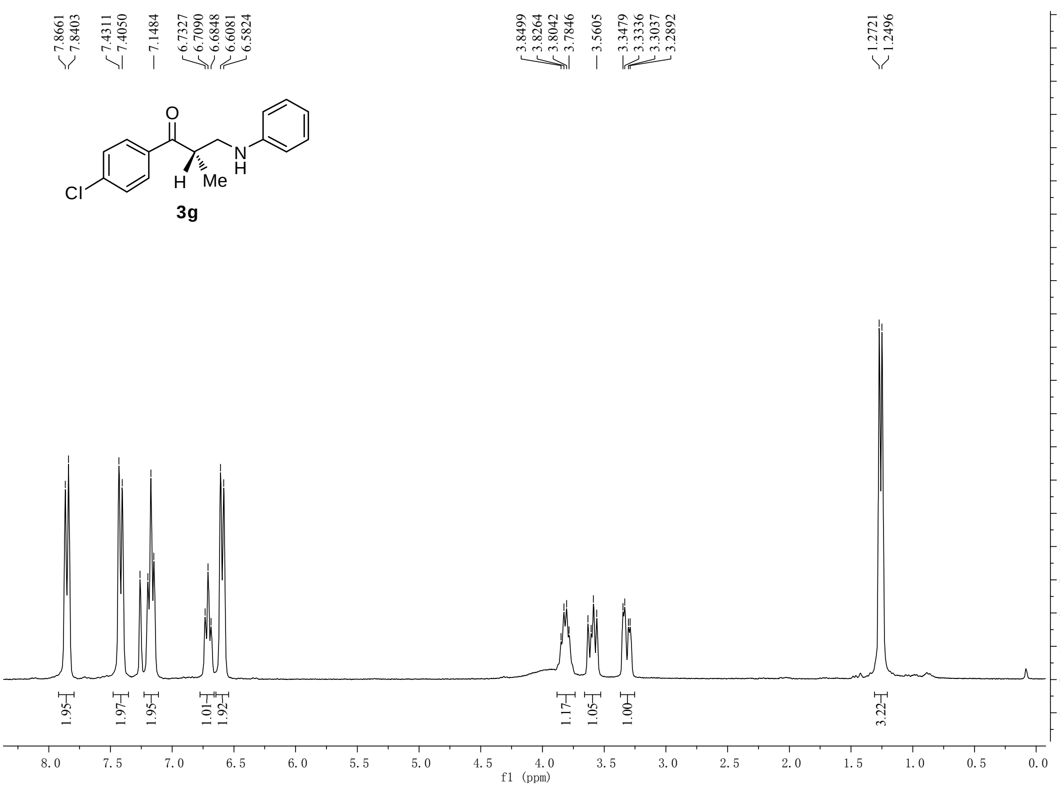

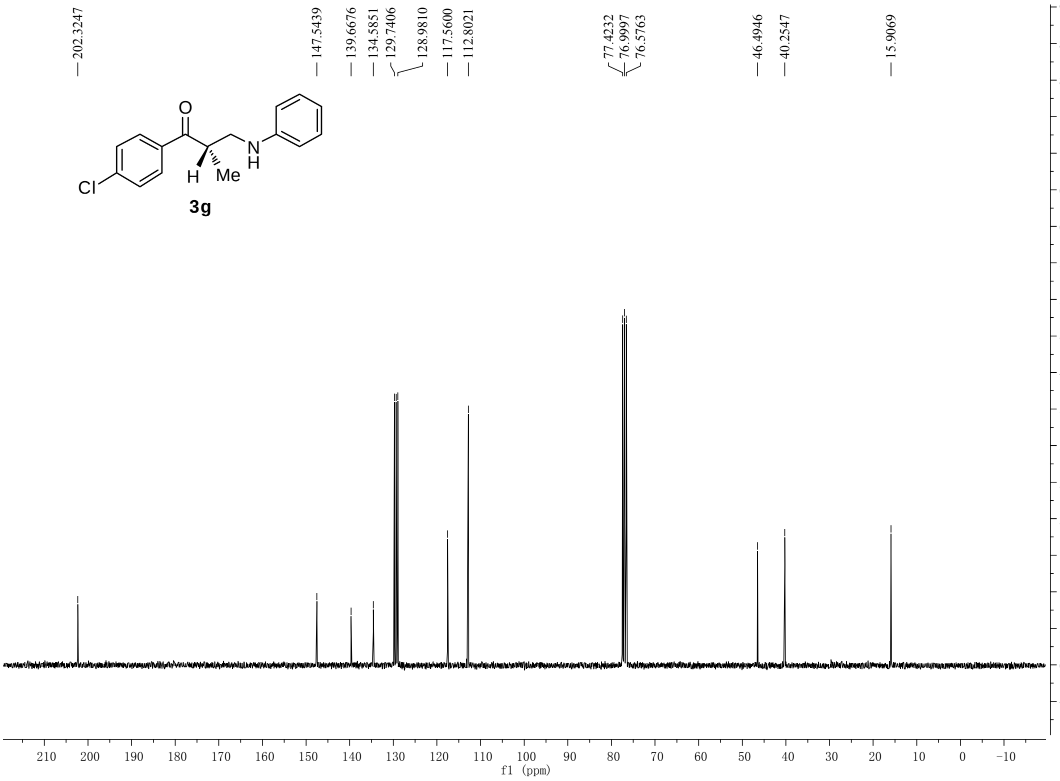

Supplementary Figure 7. ^{1}H and ^{13}C NMR spectra for compound **3g**

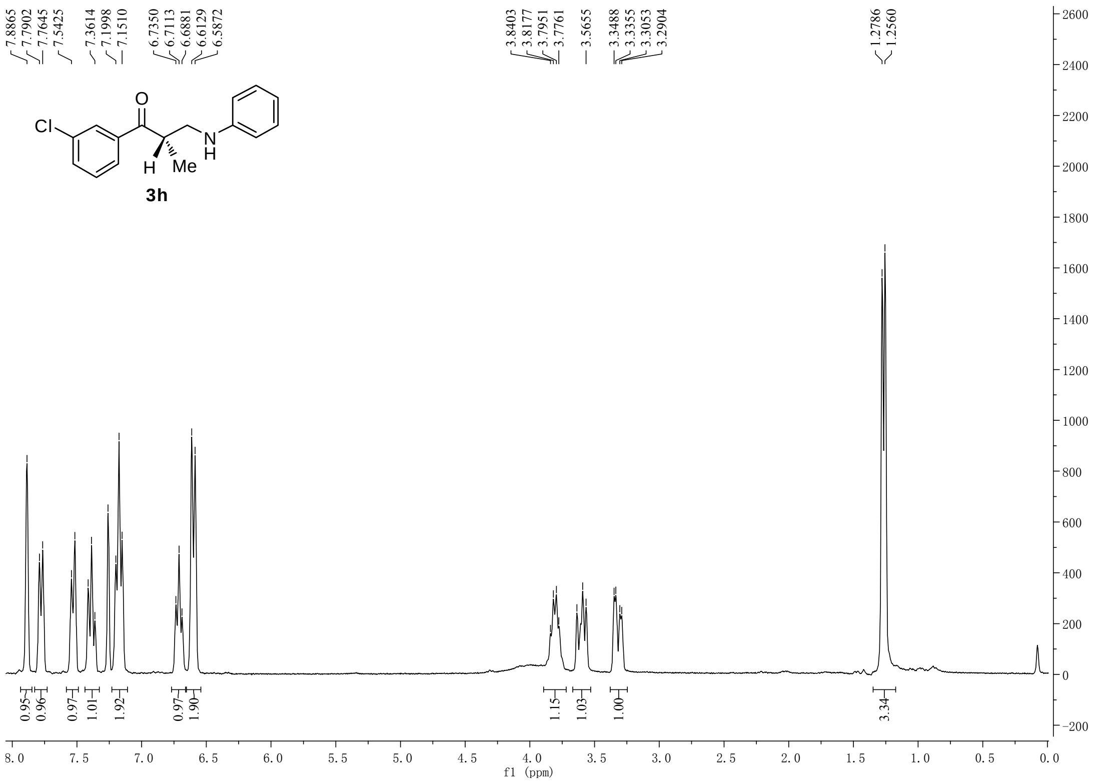

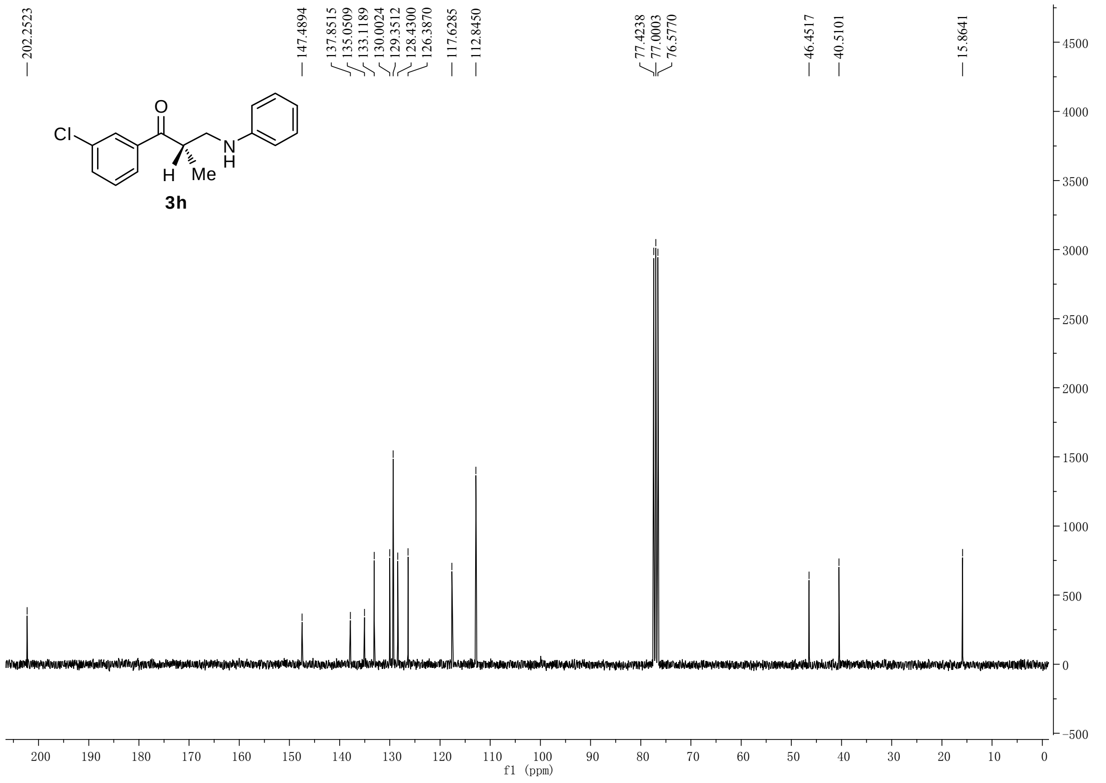

Supplementary Figure 8. ^{1}H and ^{13}C NMR spectra for compound **3h**

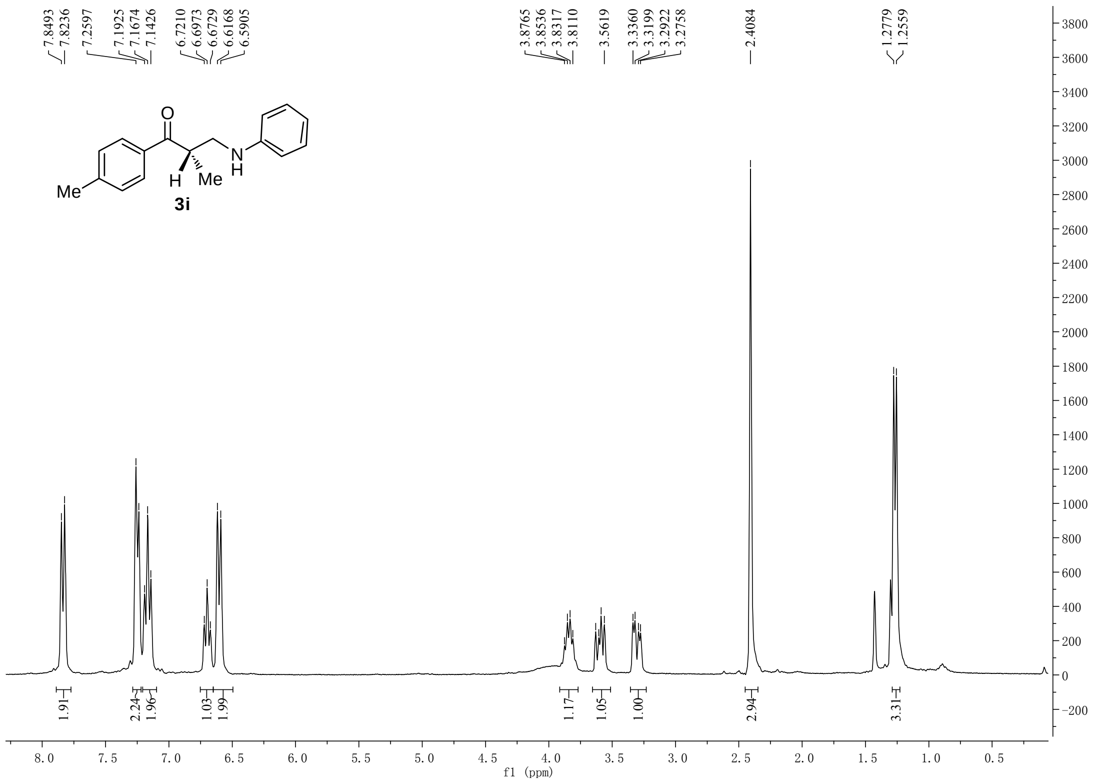

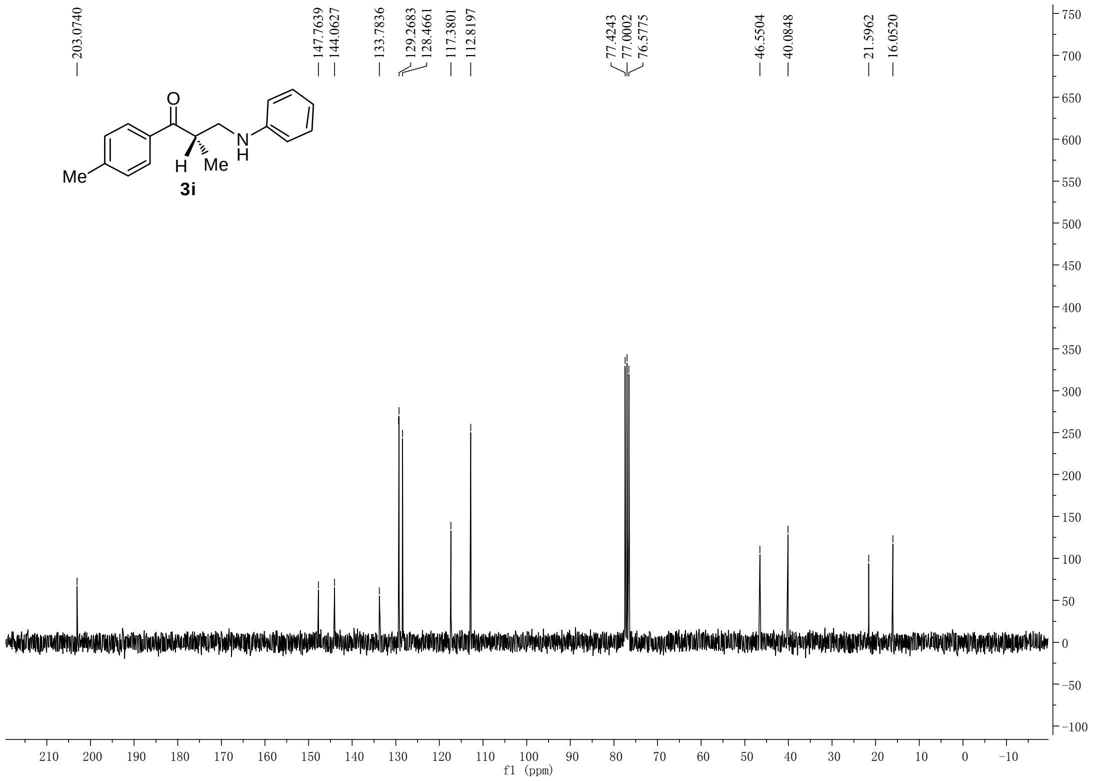

Supplementary Figure 9. ^{1}H and ^{13}C NMR spectra for compound **3i**

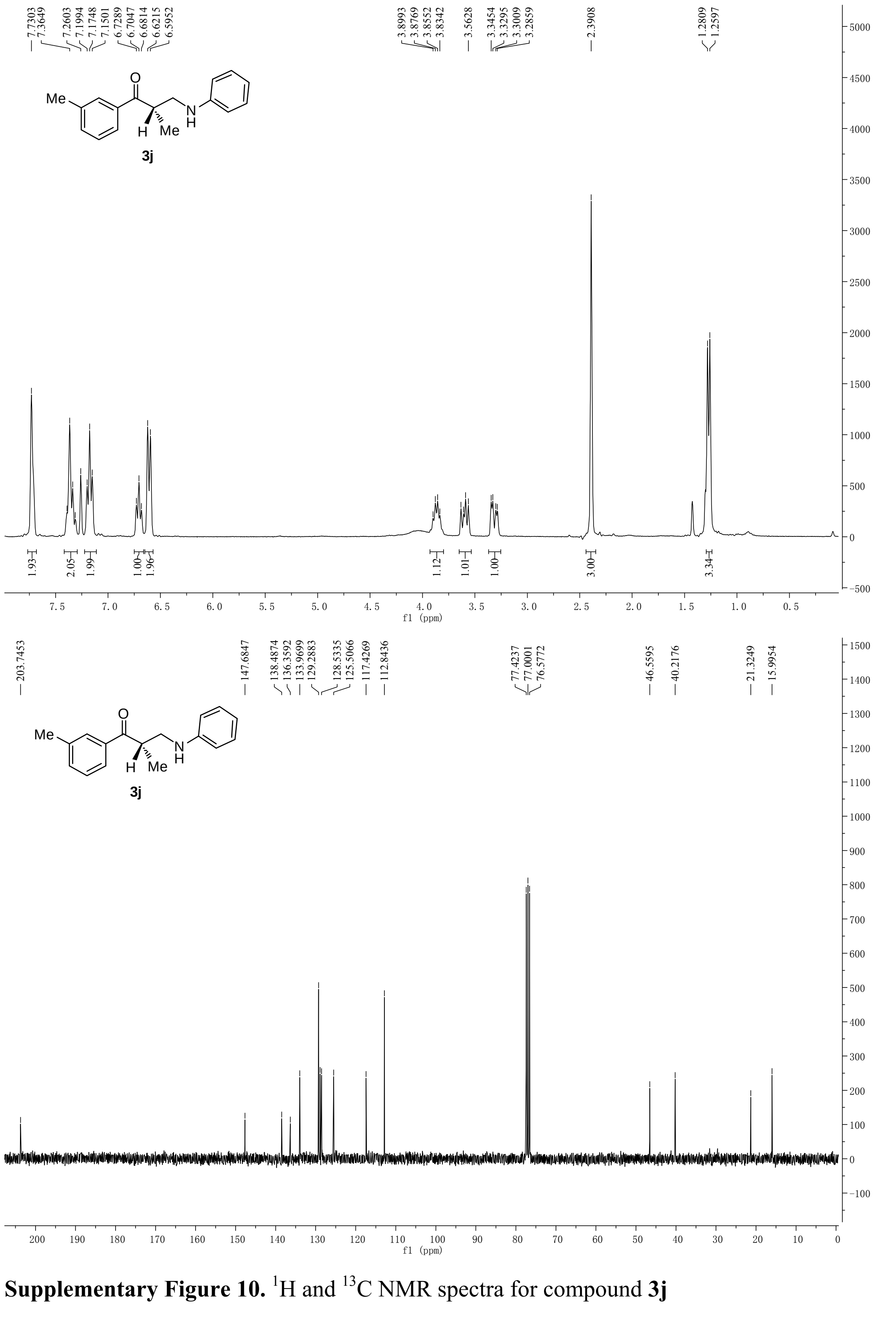

Supplementary Figure 10. ^{1}H and ^{13}C NMR spectra for compound **3j**

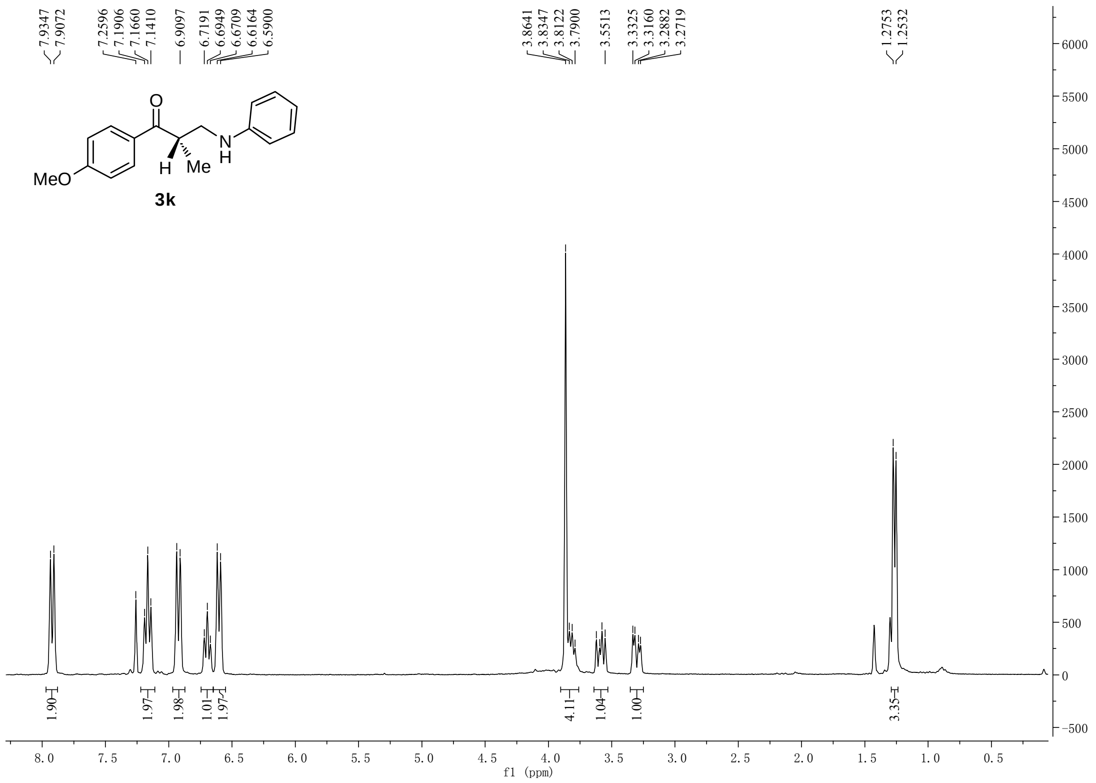

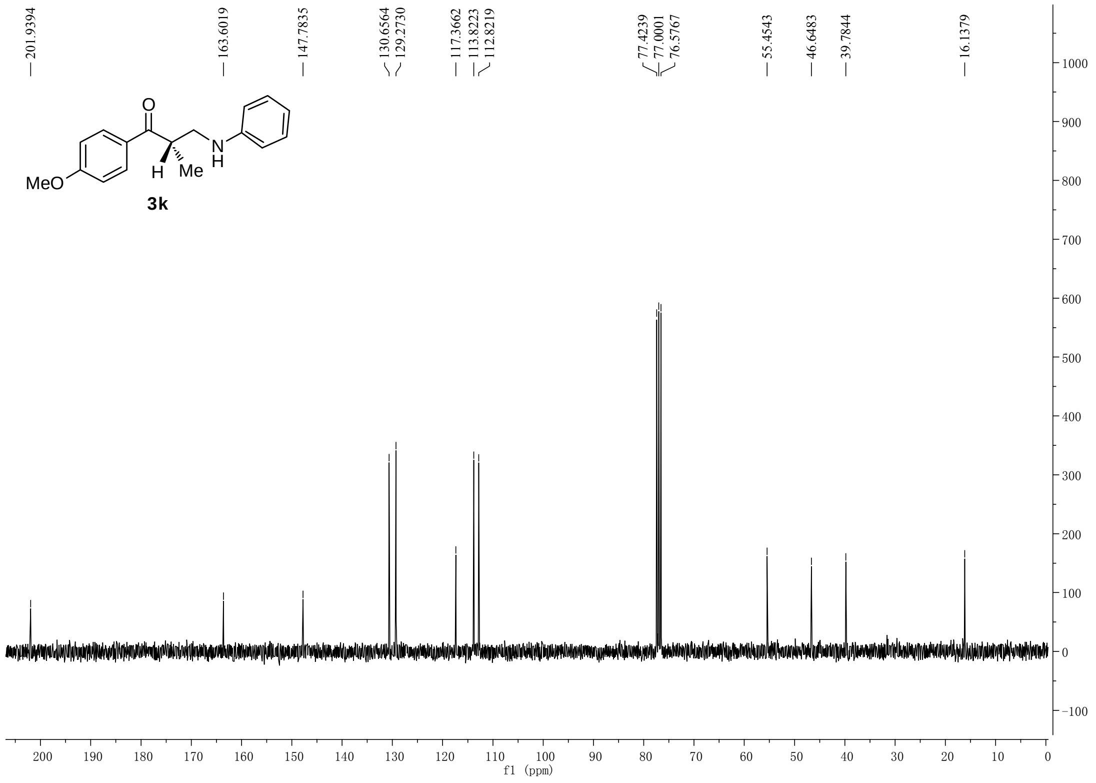

Supplementary Figure 11. ^{1}H and ^{13}C NMR spectra for compound **3k**

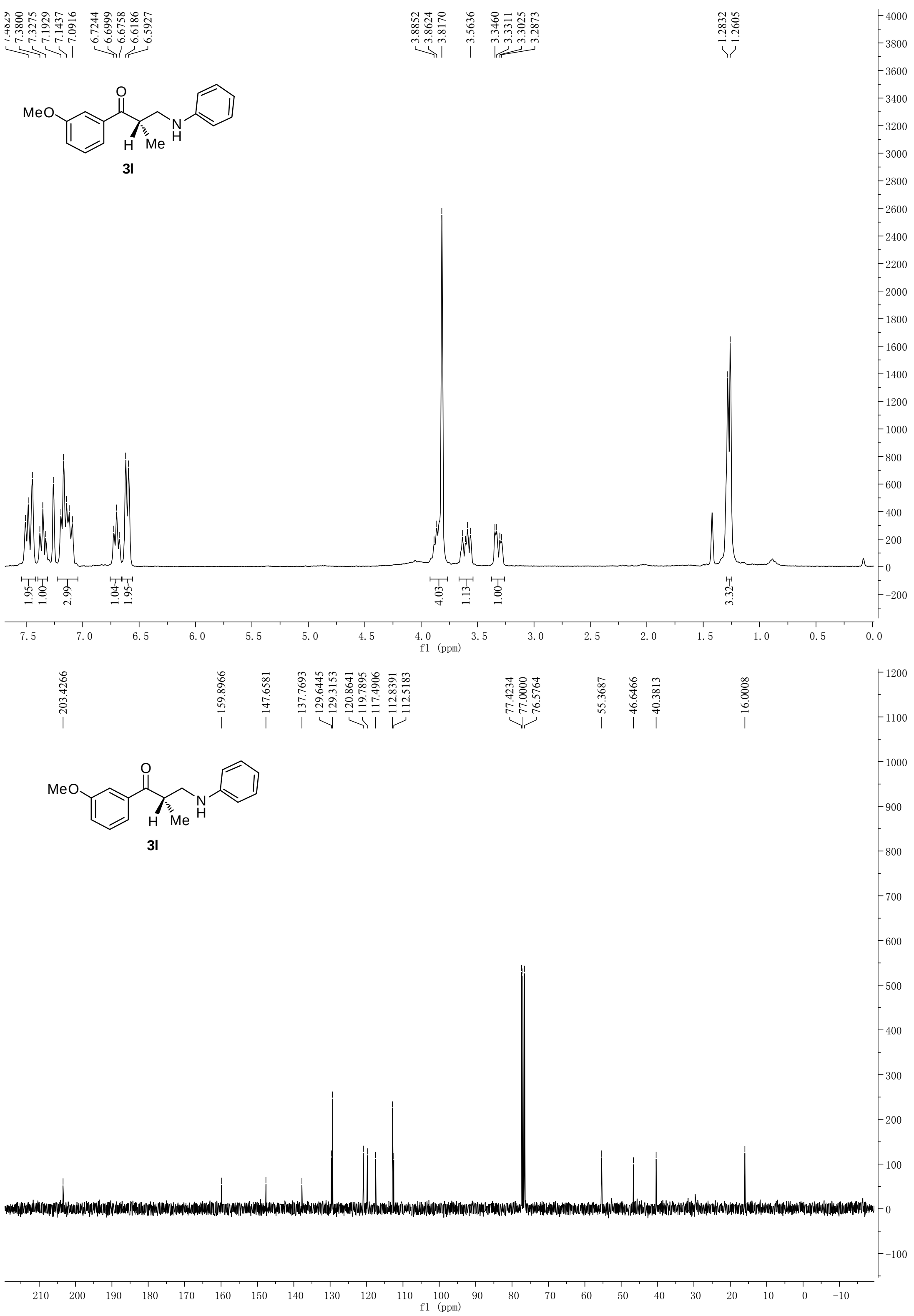

Supplementary Figure 12. ^{1}H and ^{13}C NMR spectra for compound **3l**

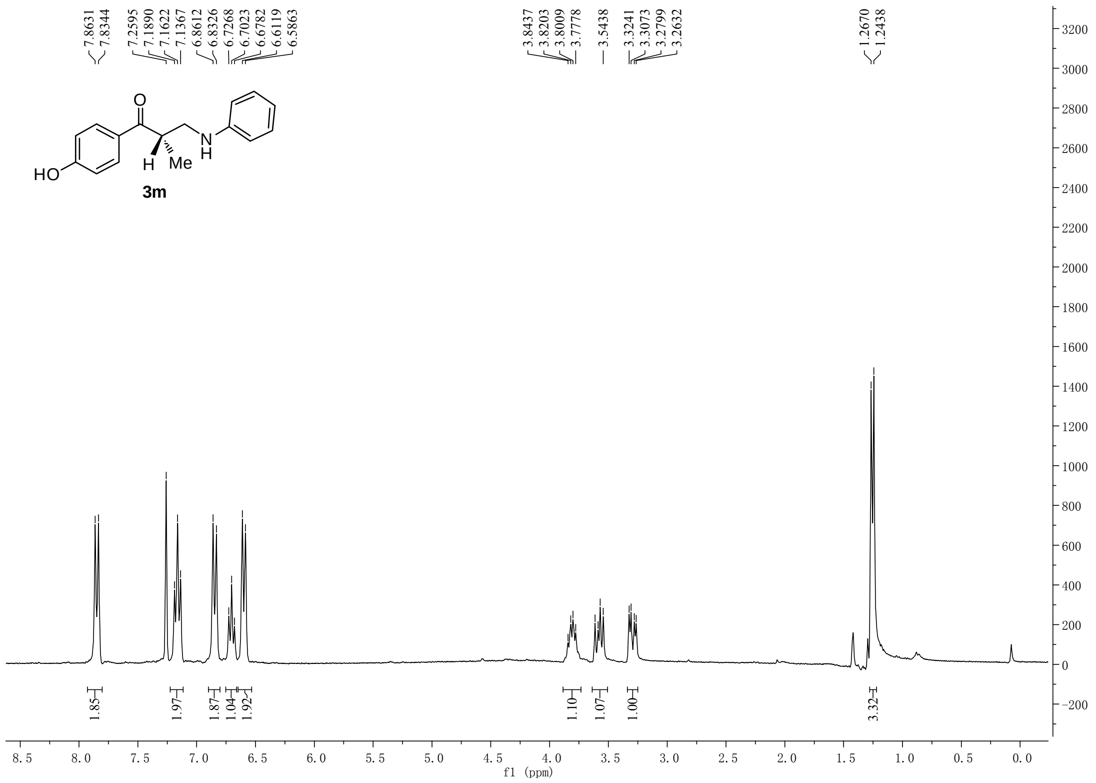

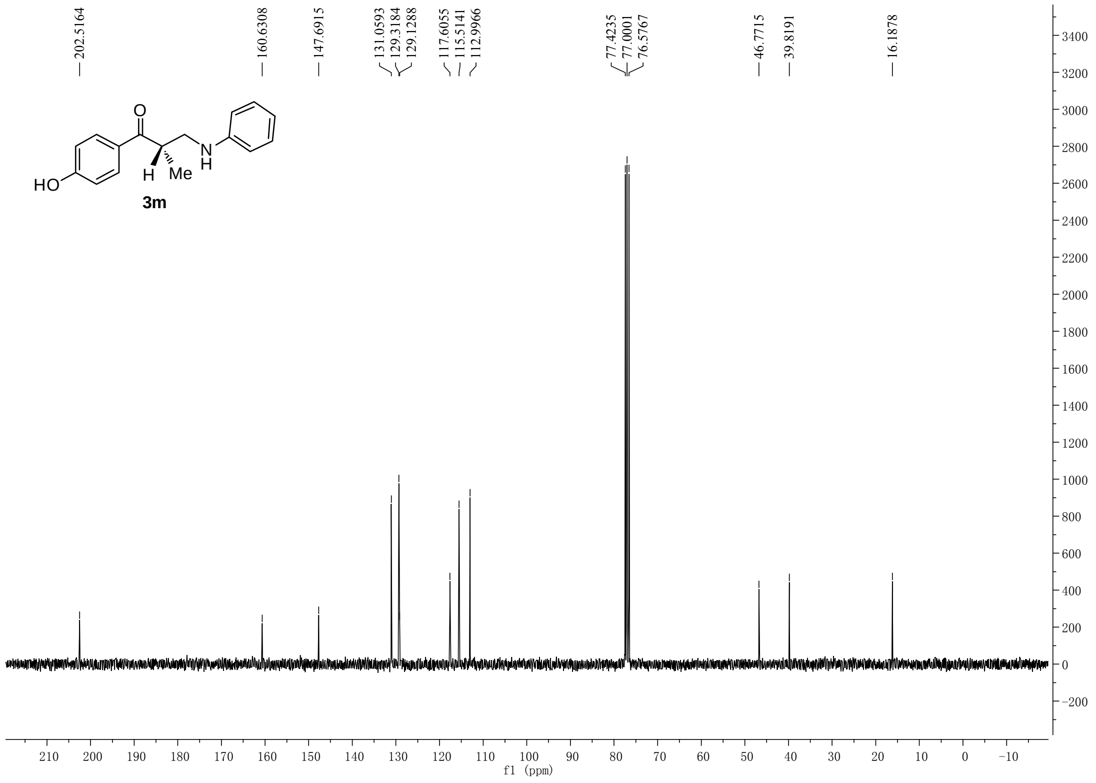

Supplementary Figure 13. ^{1}H and ^{13}C NMR spectra for compound **3m**

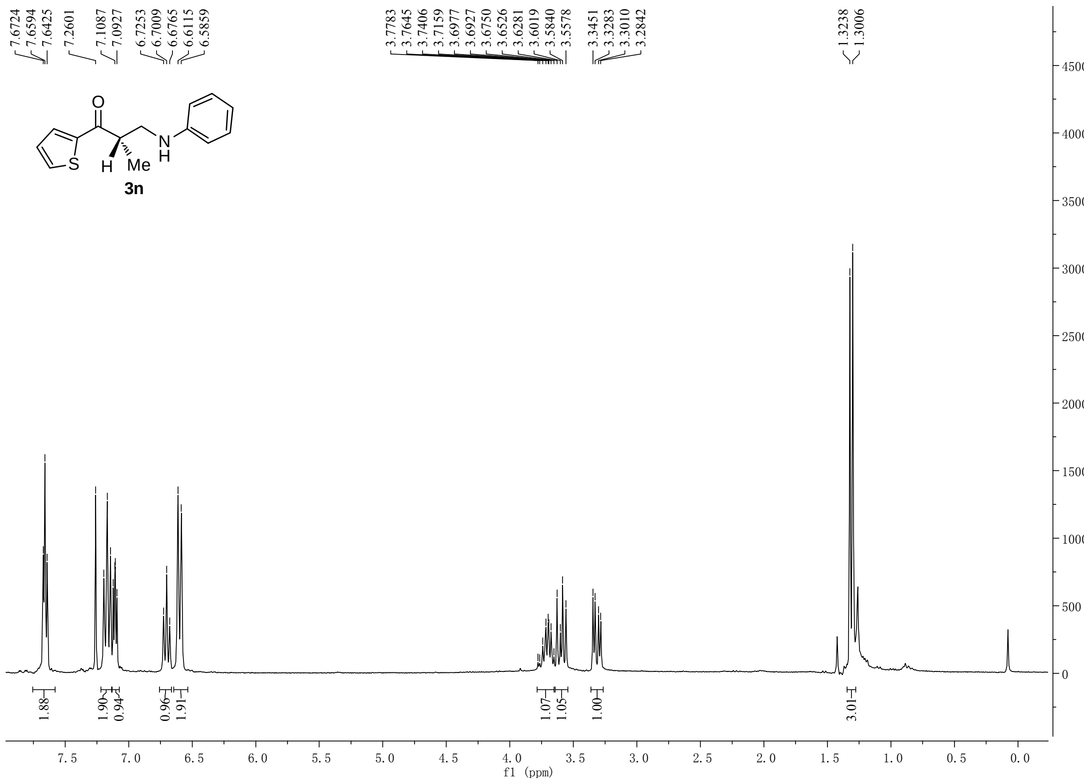

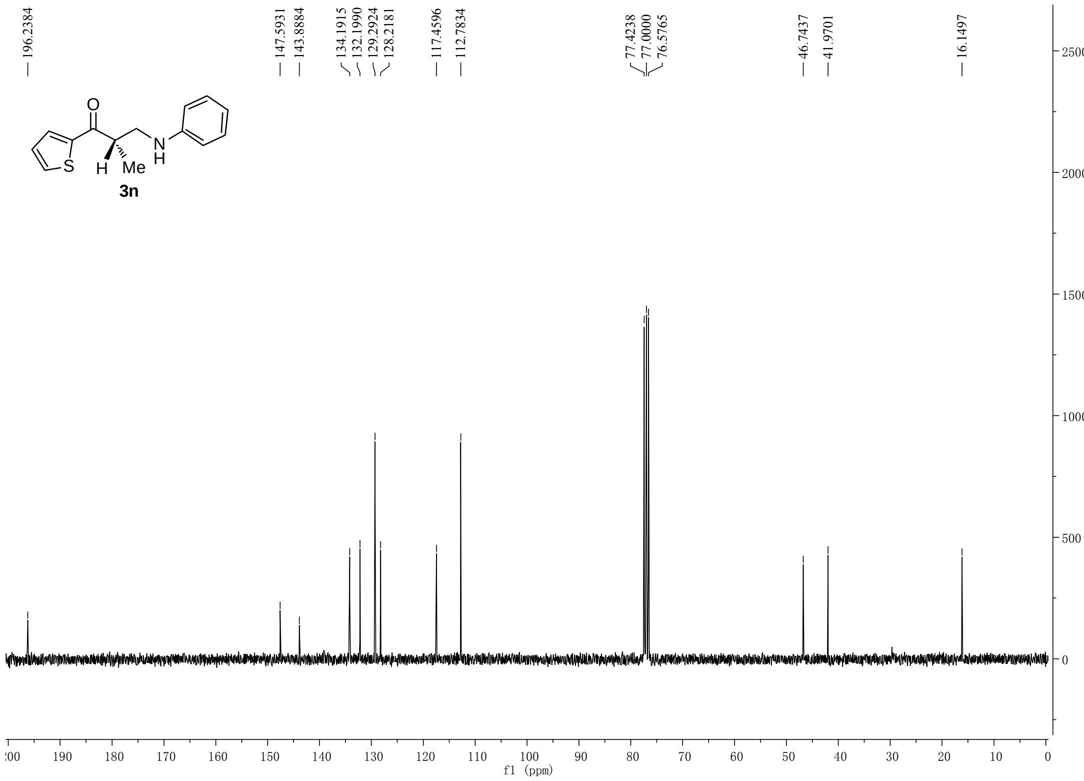

Supplementary Figure 14. ^{1}H and ^{13}C NMR spectra for compound **3n**

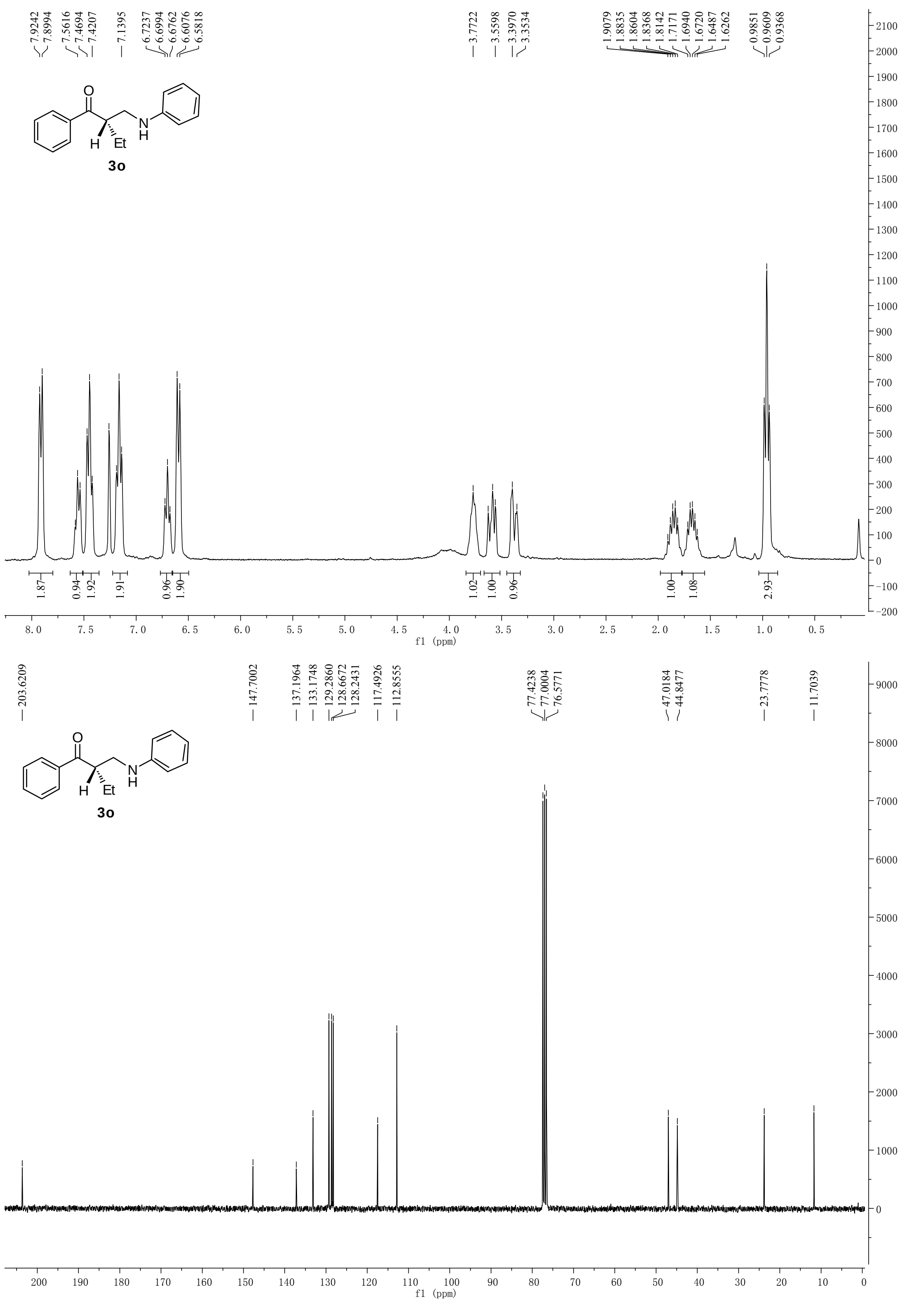

Supplementary Figure 15. ^{1}H and ^{13}C NMR spectra for compound **3o**

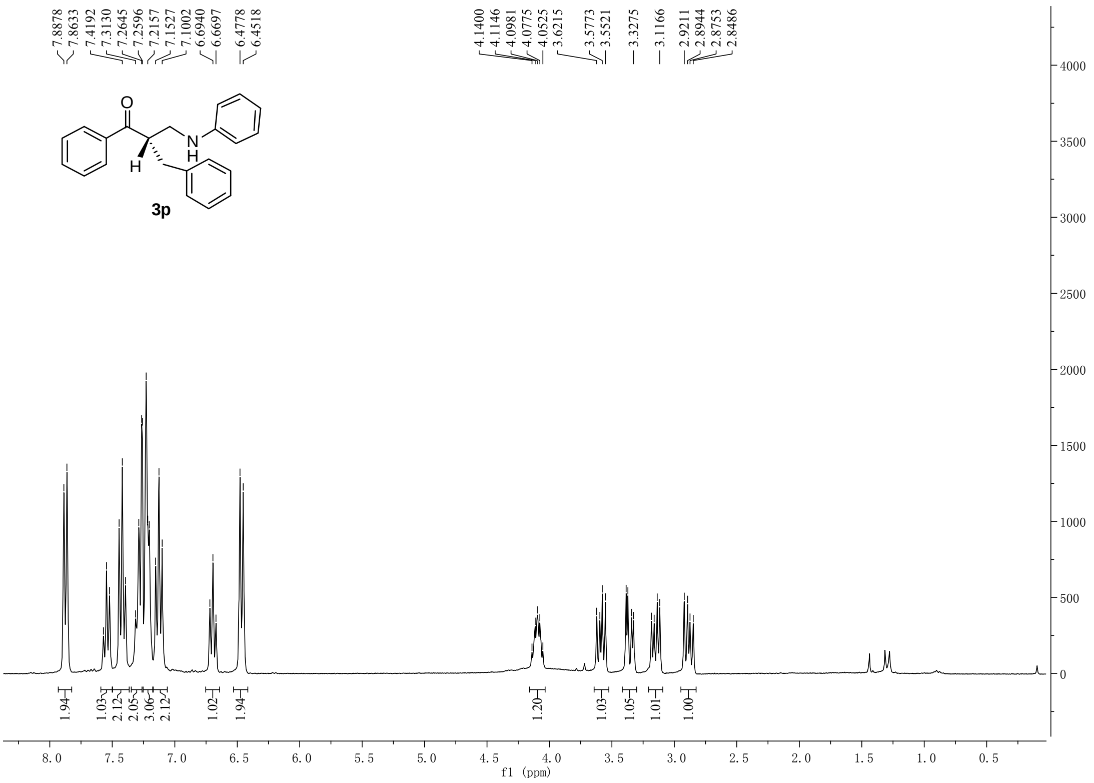

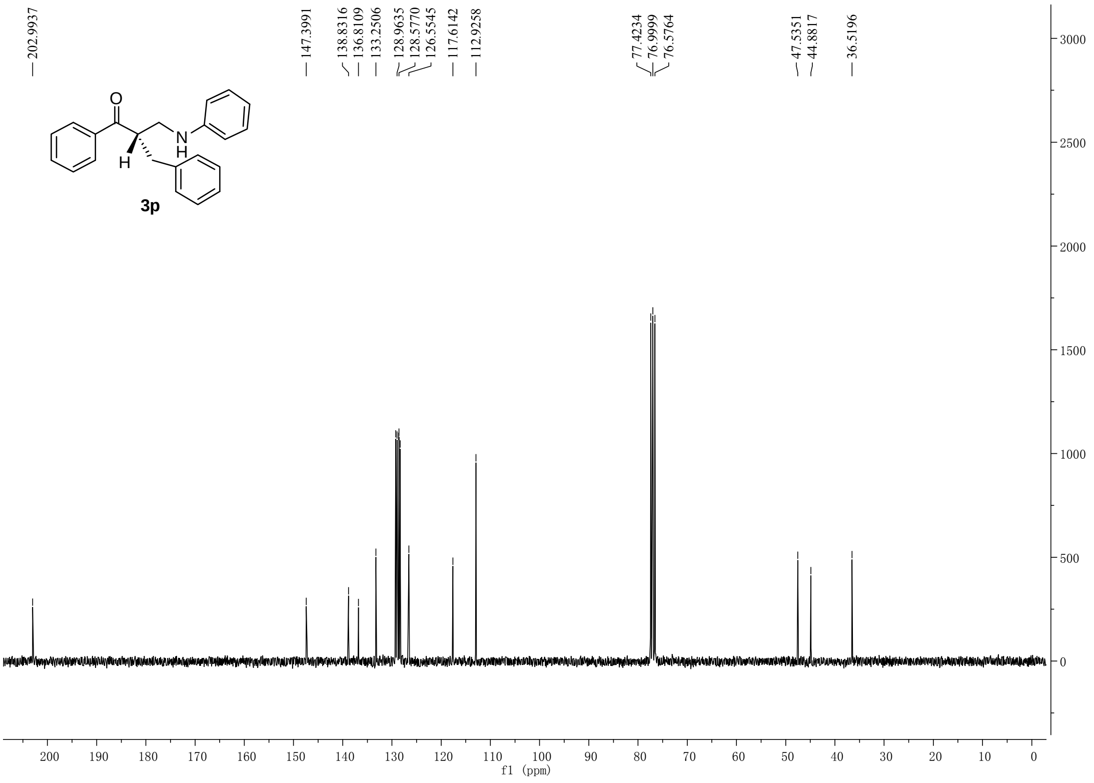

Supplementary Figure 16. ^{1}H and ^{13}C NMR spectra for compound **3p**

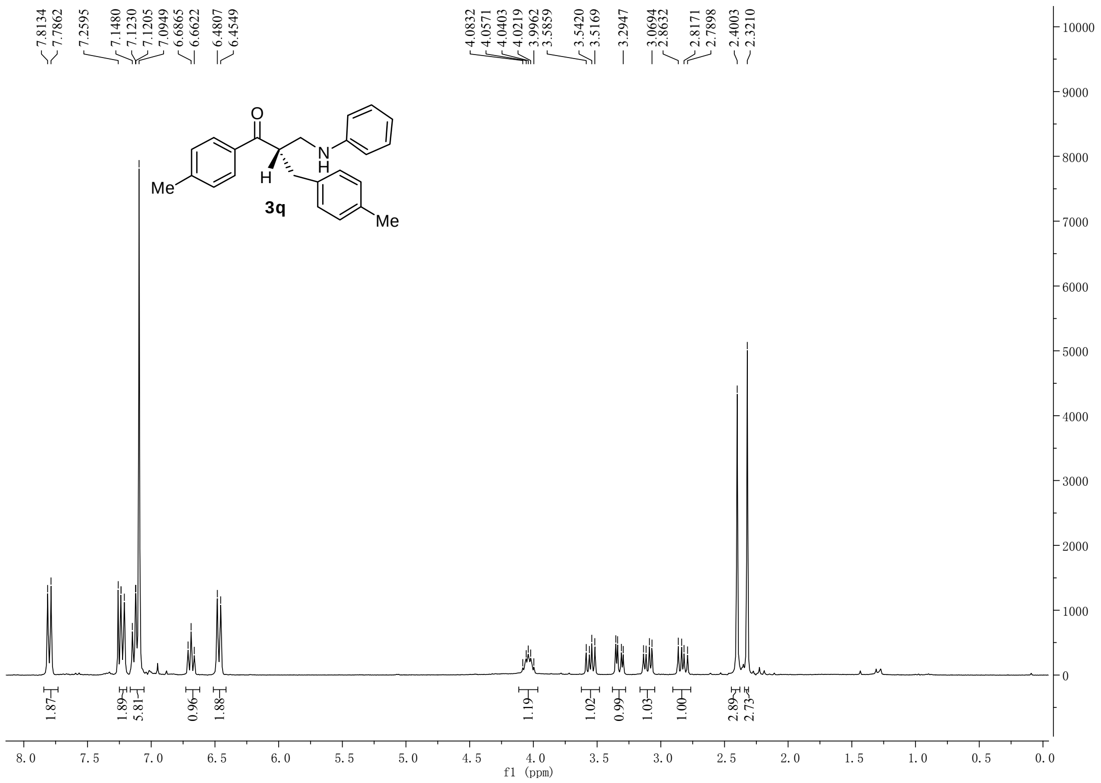

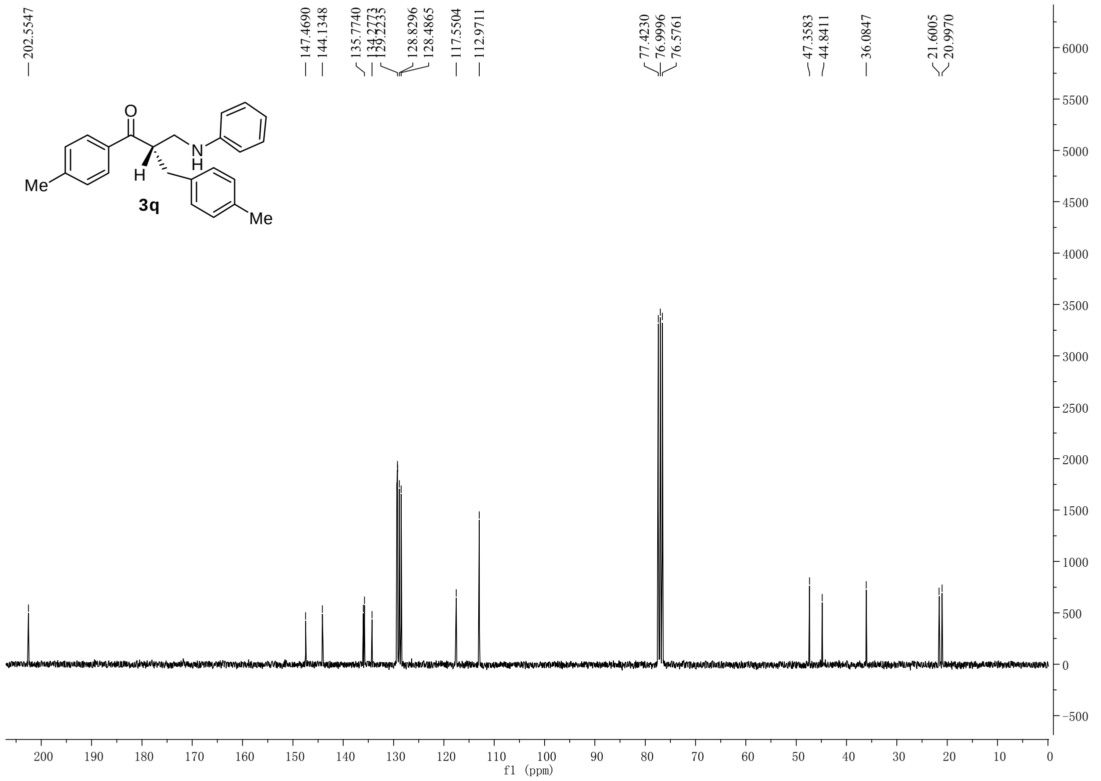

Supplementary Figure 17. ^{1}H and ^{13}C NMR spectra for compound **3q**

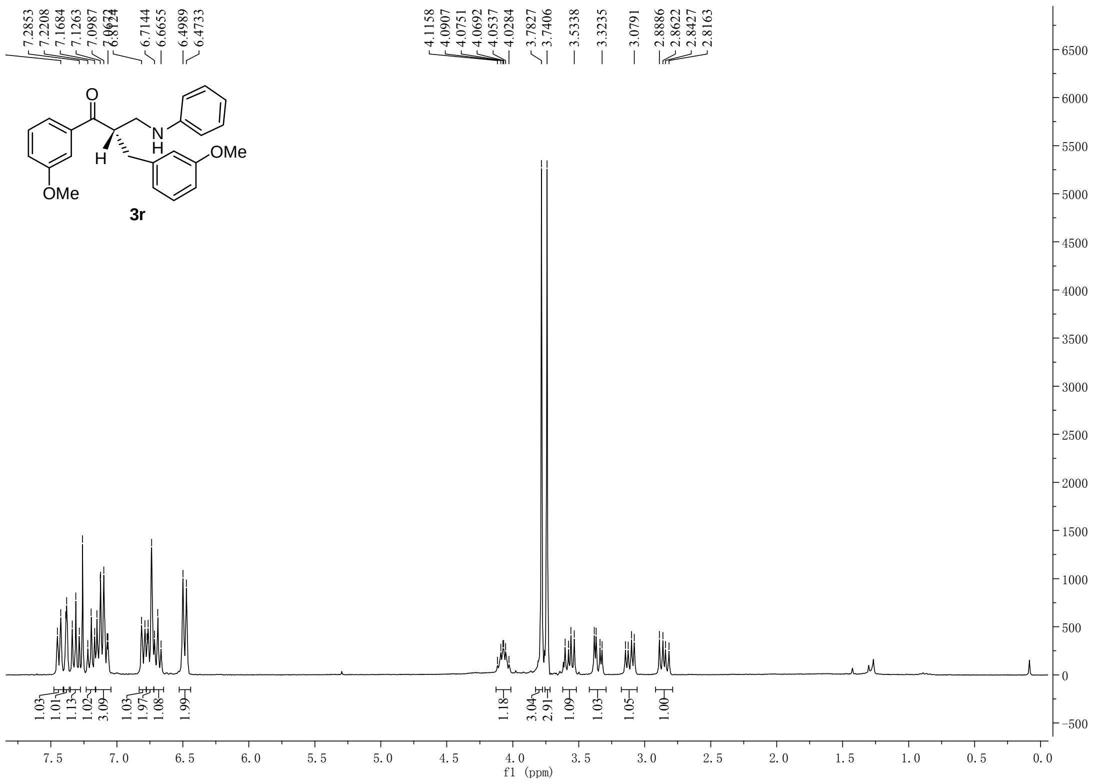

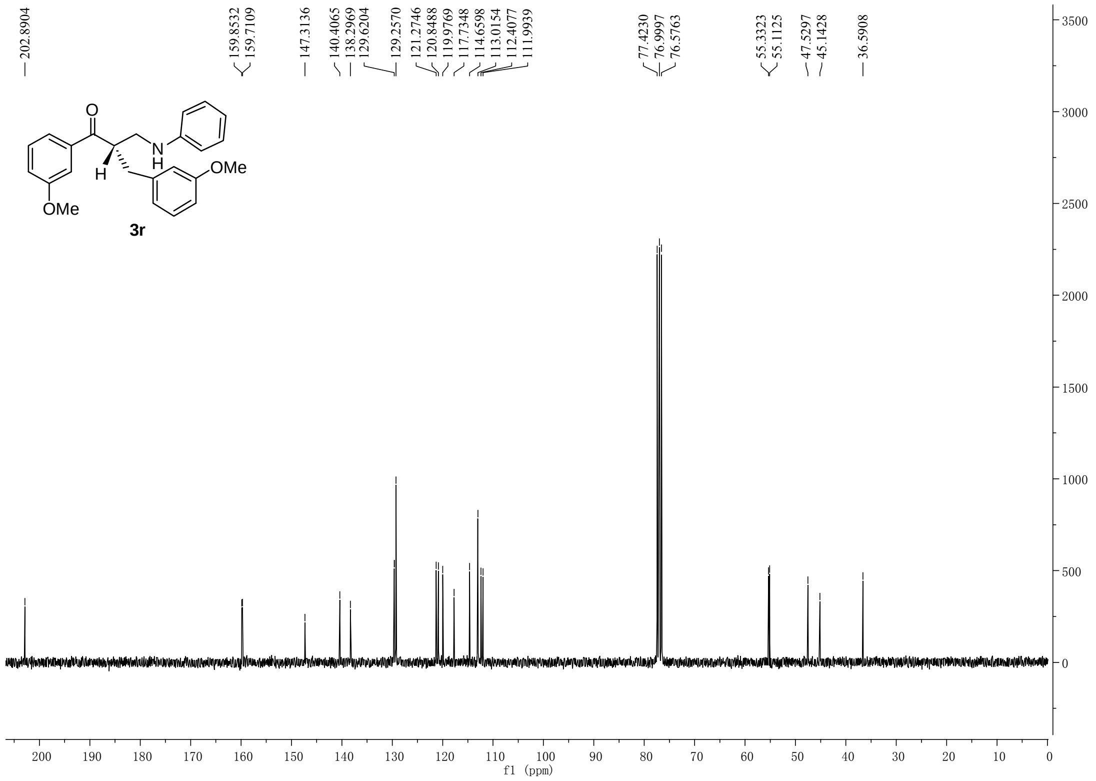

Supplementary Figure 18. ^{1}H and ^{13}C NMR spectra for compound **3r**

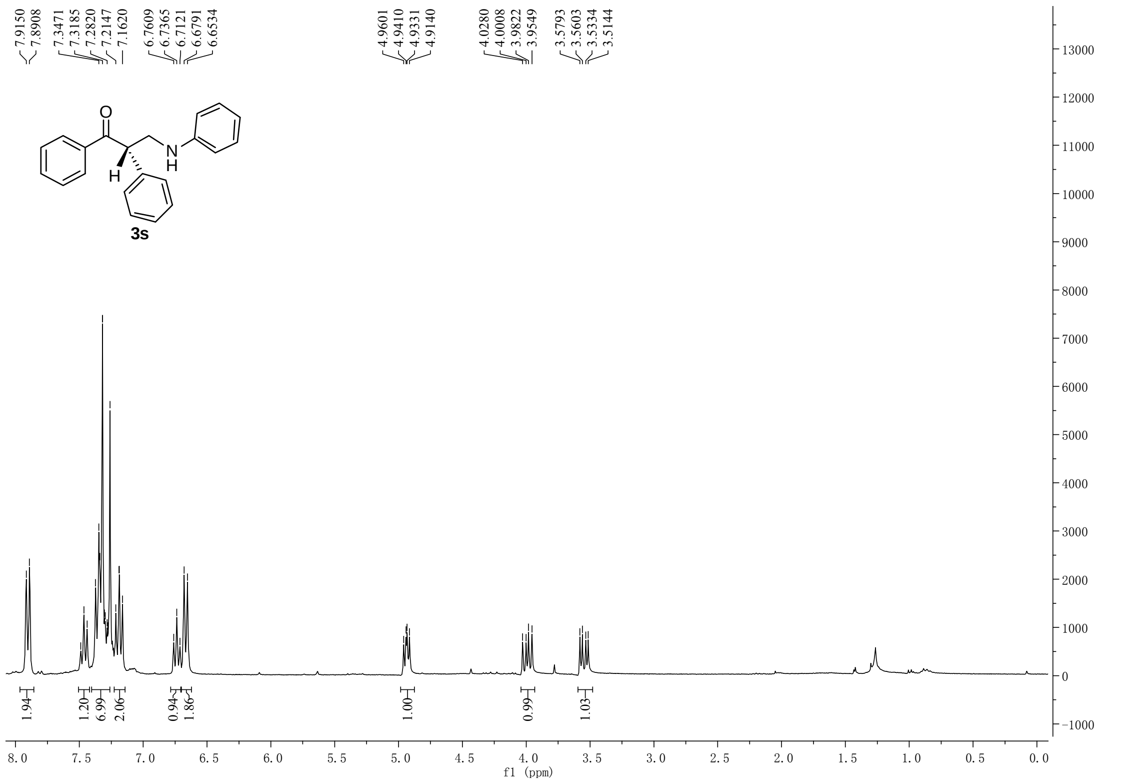

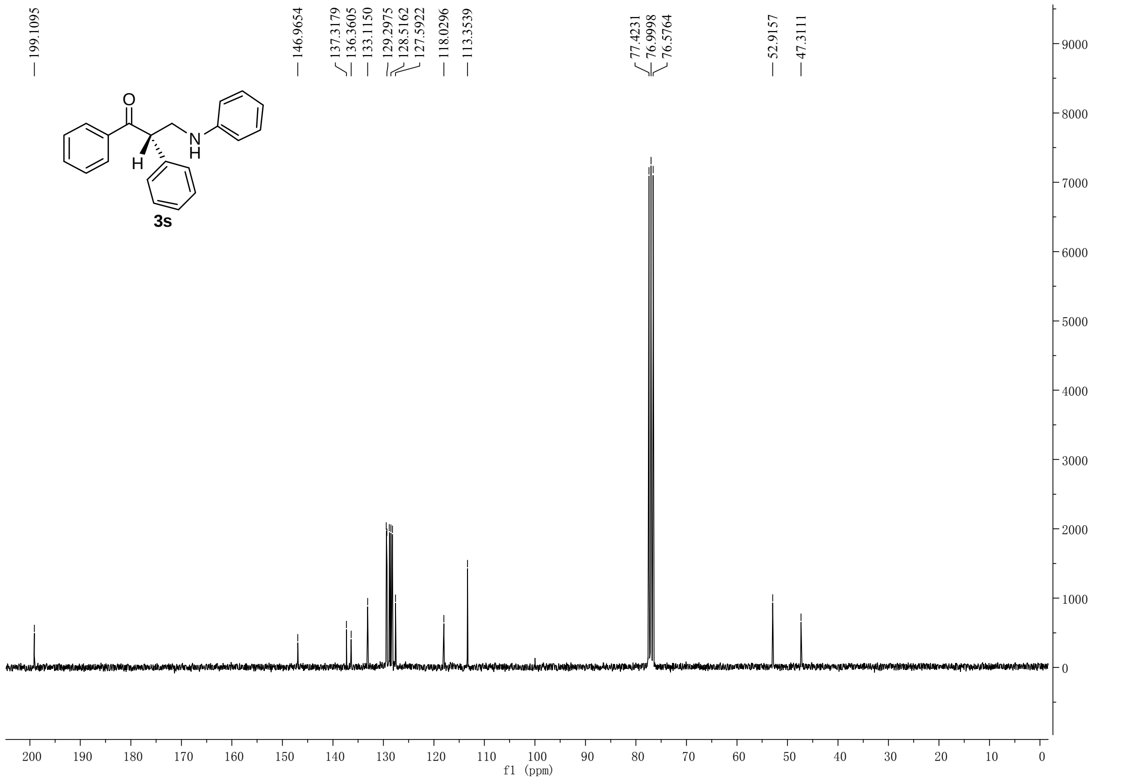

Supplementary Figure 19. ^{1}H and ^{13}C NMR spectra for compound **3s**

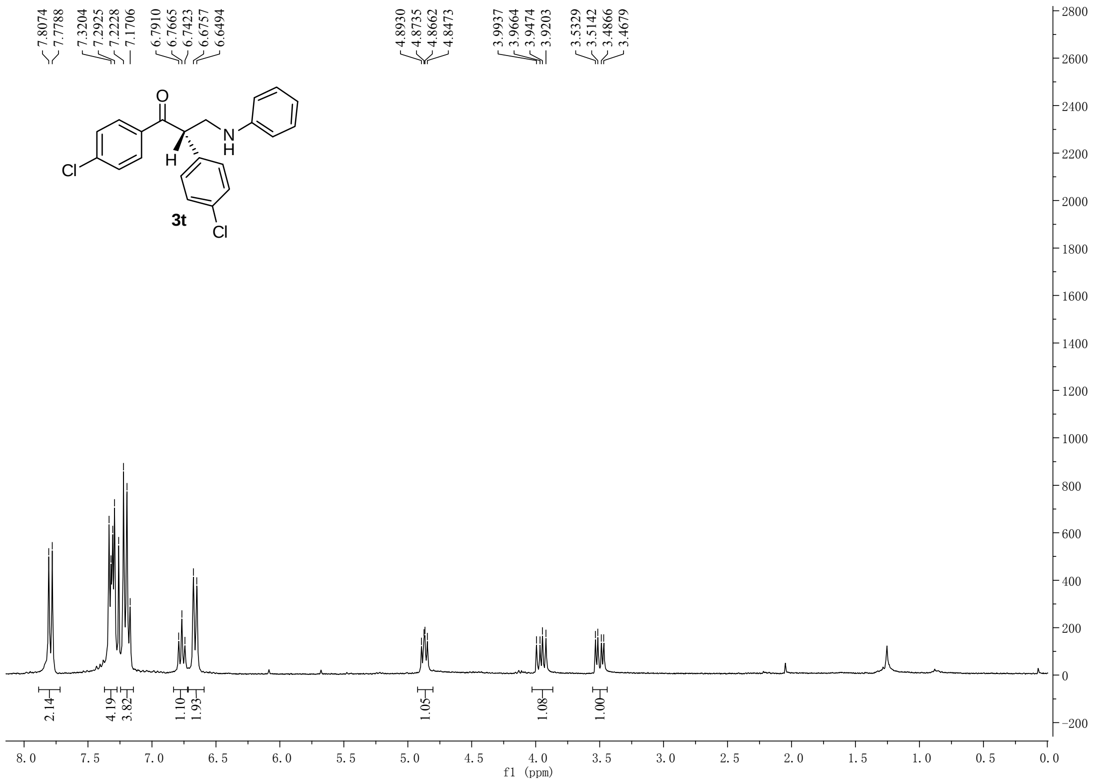

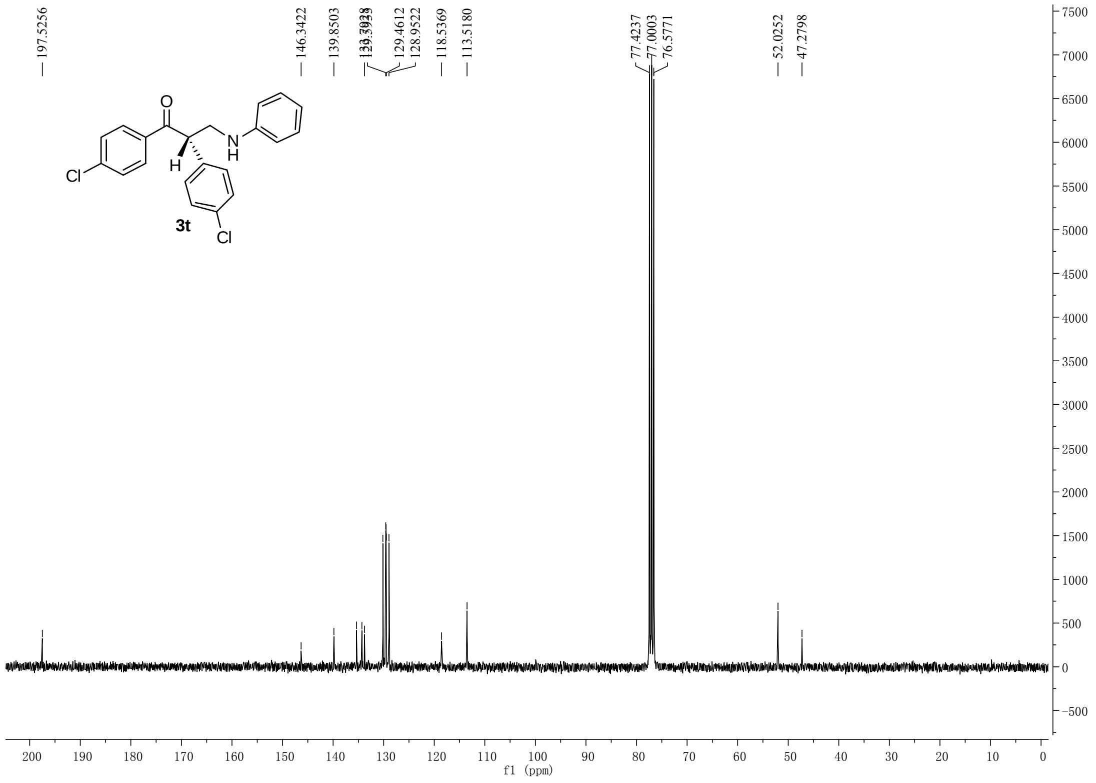

Supplementary Figure 20. ^{1}H and ^{13}C NMR spectra for compound **3t**

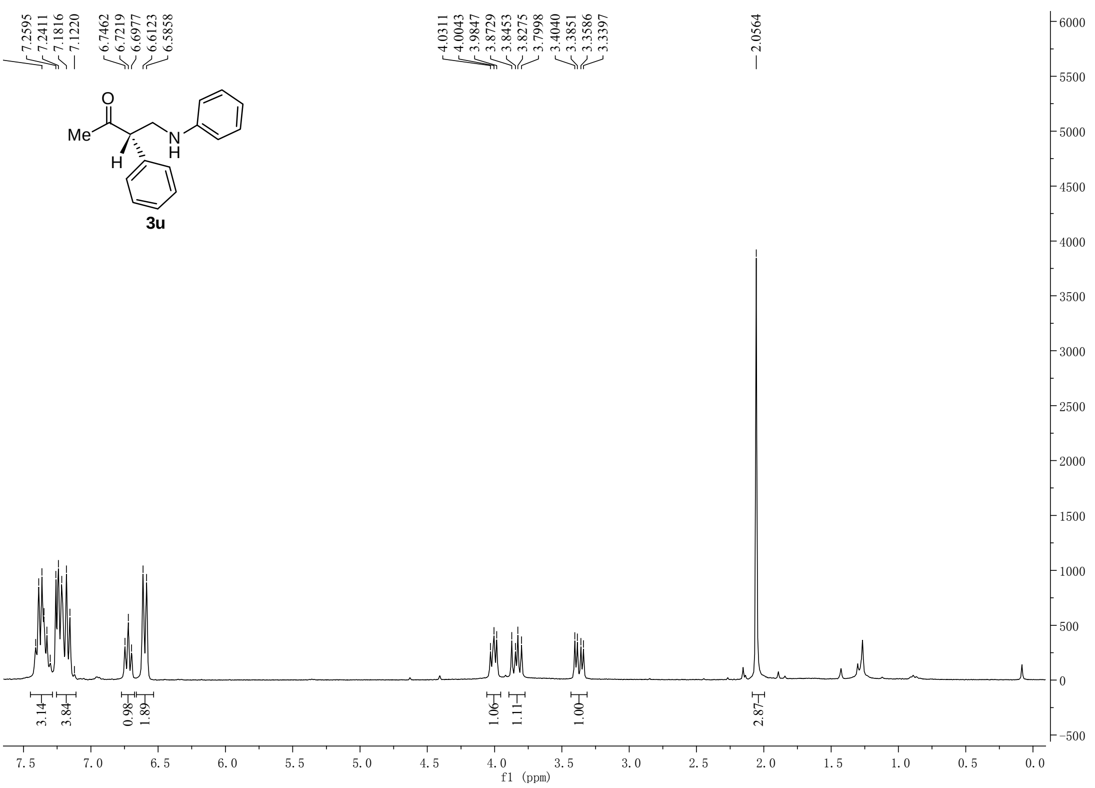

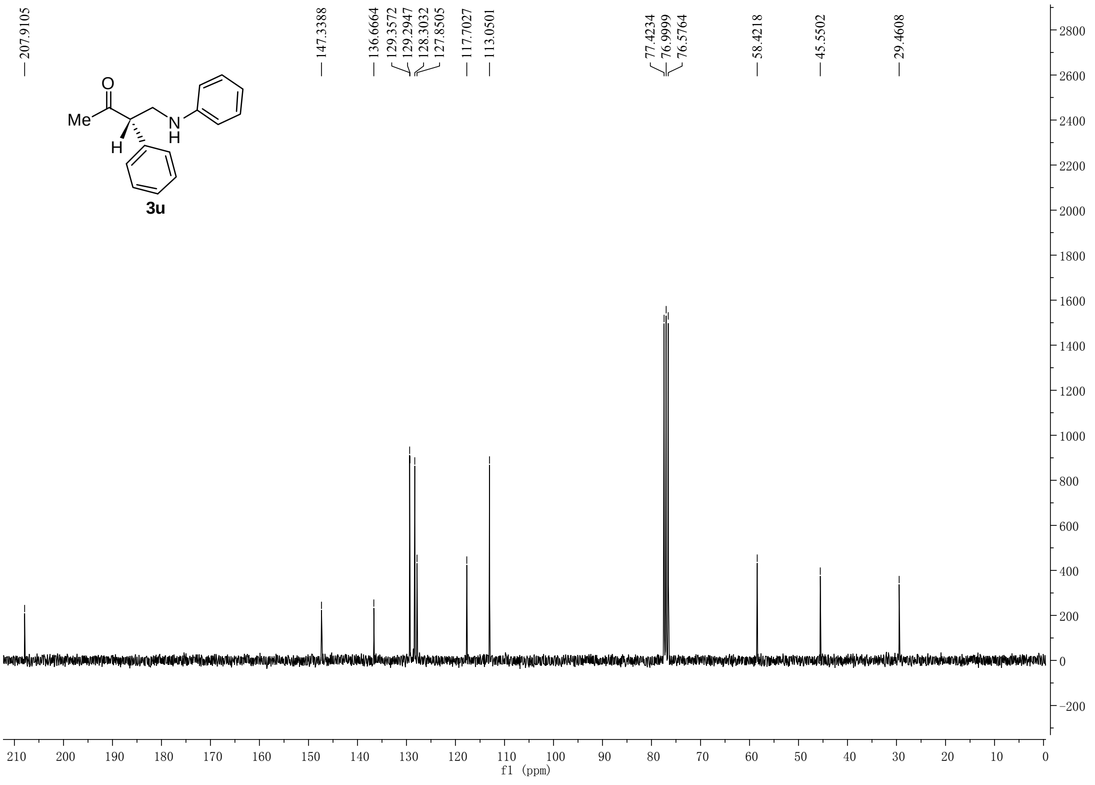

Supplementary Figure 21. ^{1}H and ^{13}C NMR spectra for compound **3u**

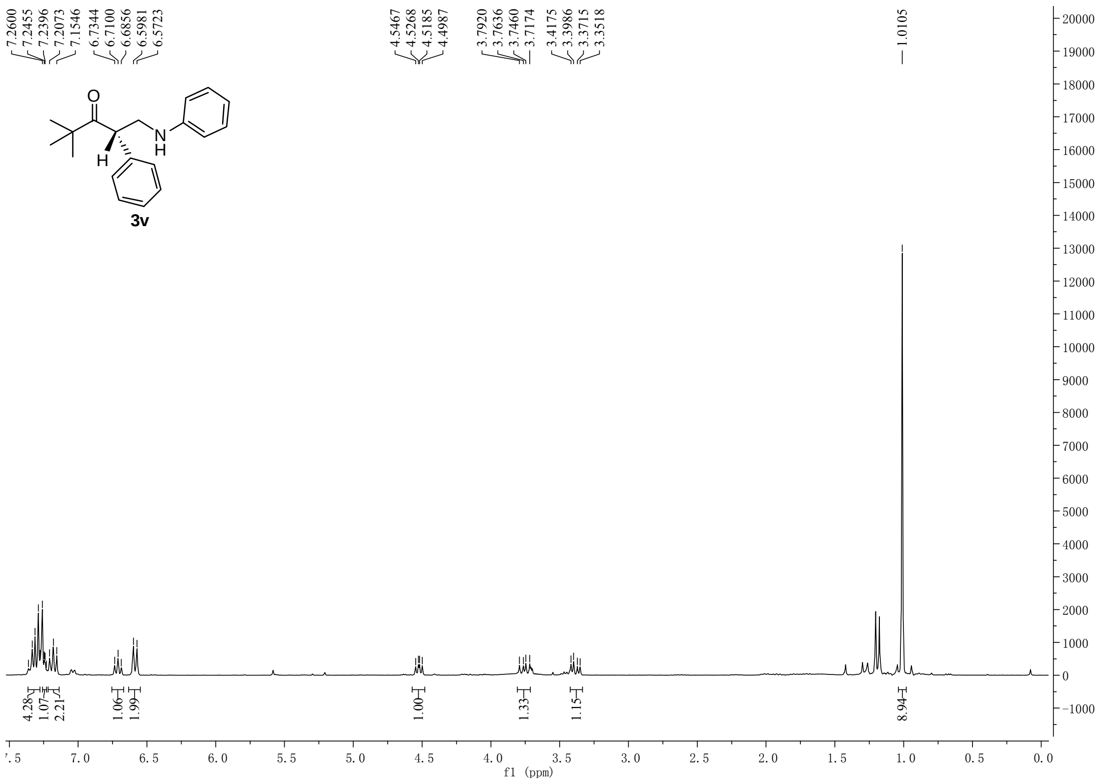

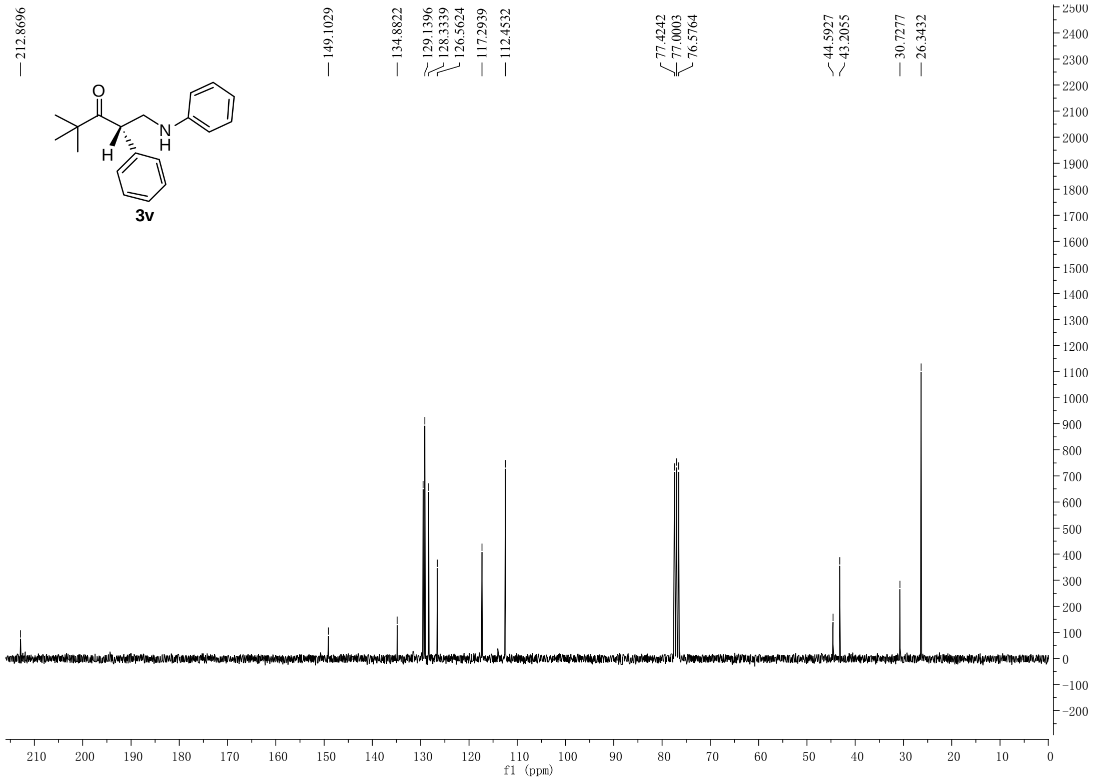

Supplementary Figure 22. ^{1}H and ^{13}C NMR spectra for compound **3v**

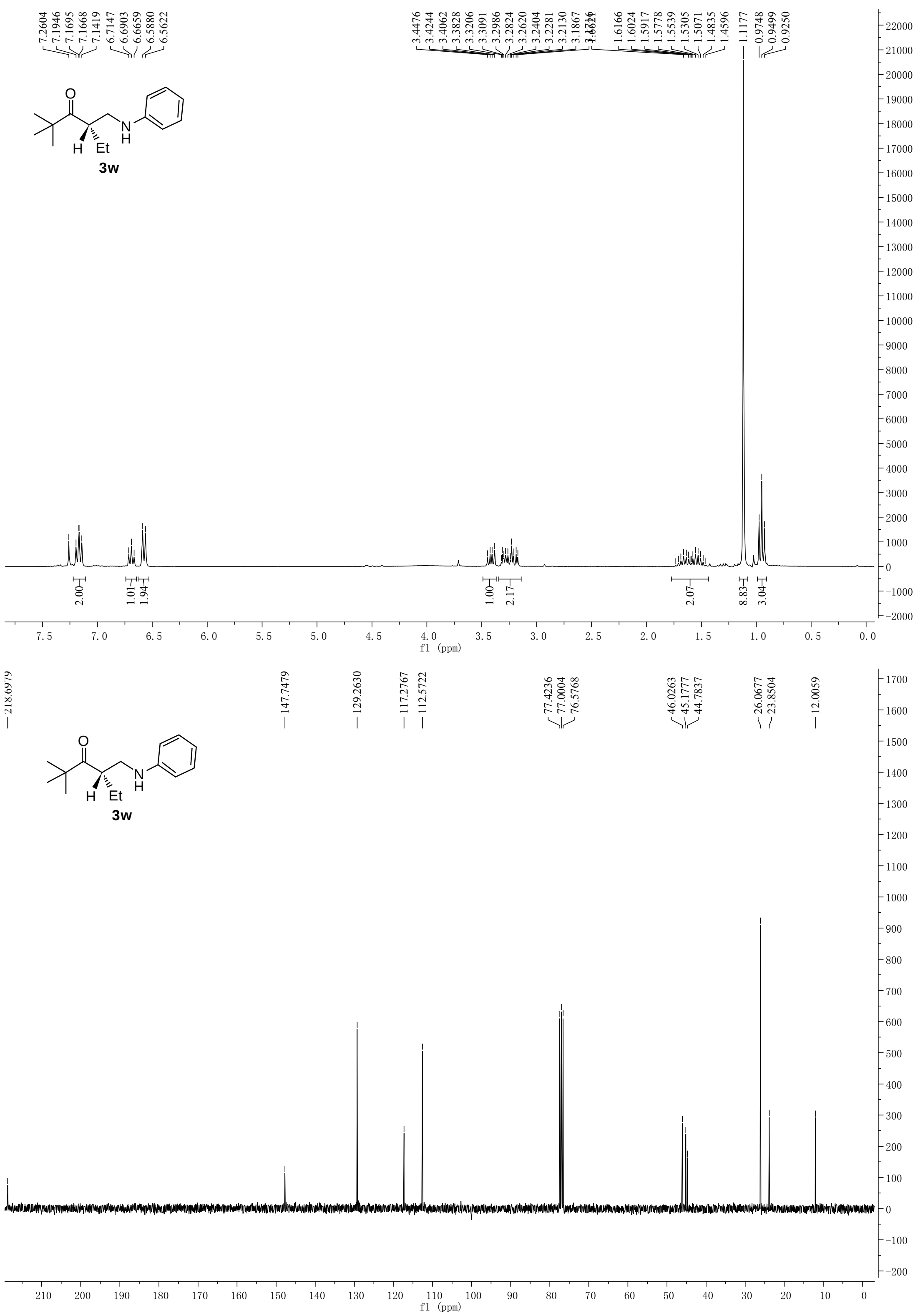

Supplementary Figure 23. ^{1}H and ^{13}C NMR spectra for compound **3w**

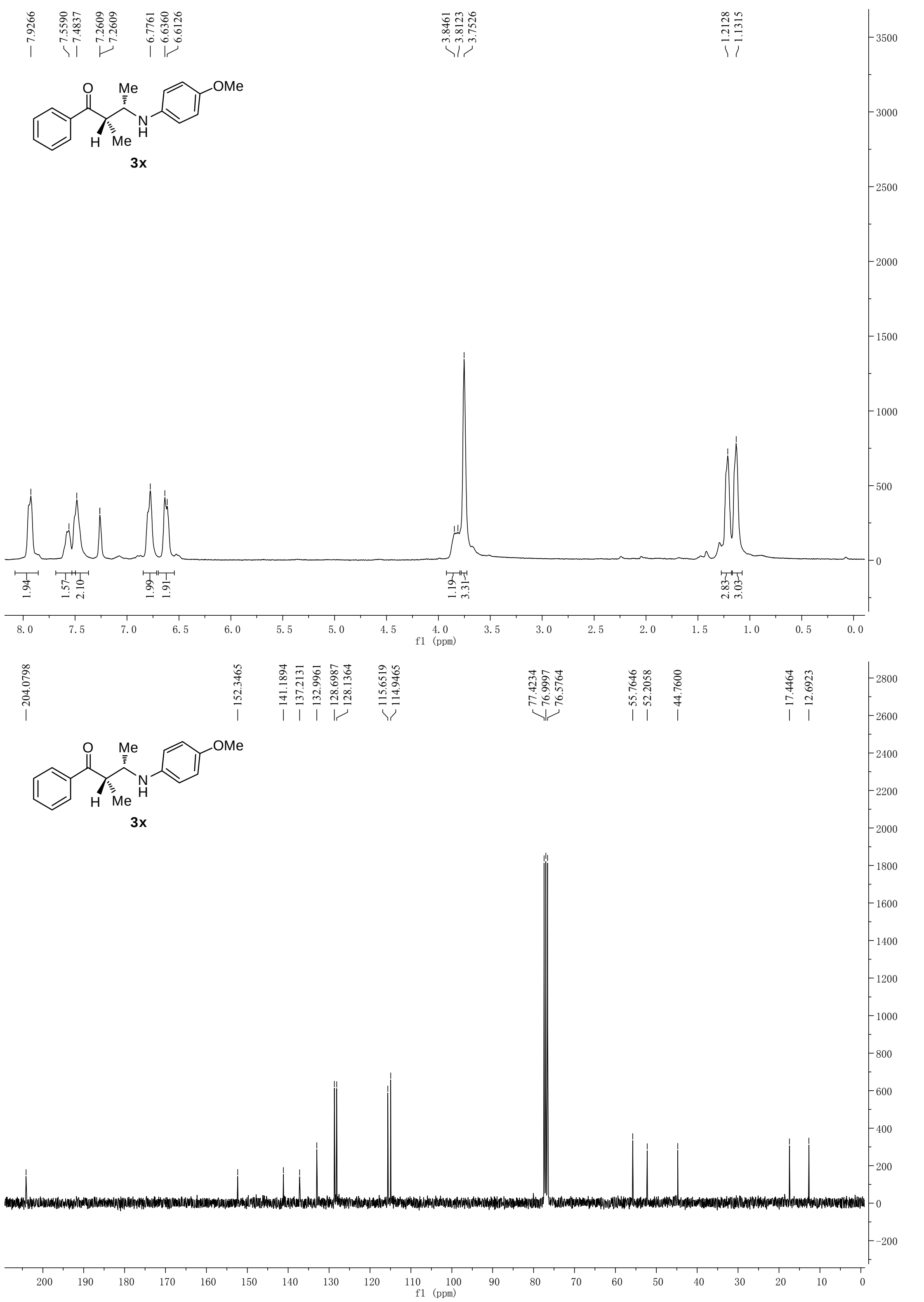

Supplementary Figure 24. ^{1}H and ^{13}C NMR spectra for compound **3x**

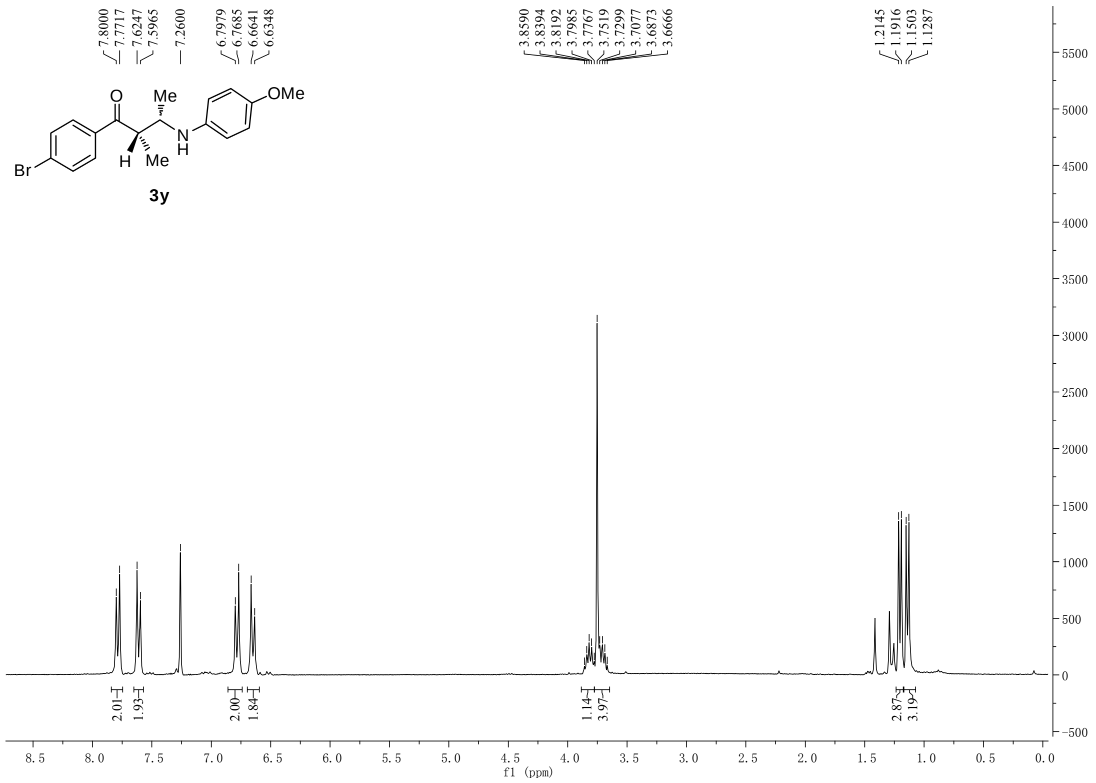

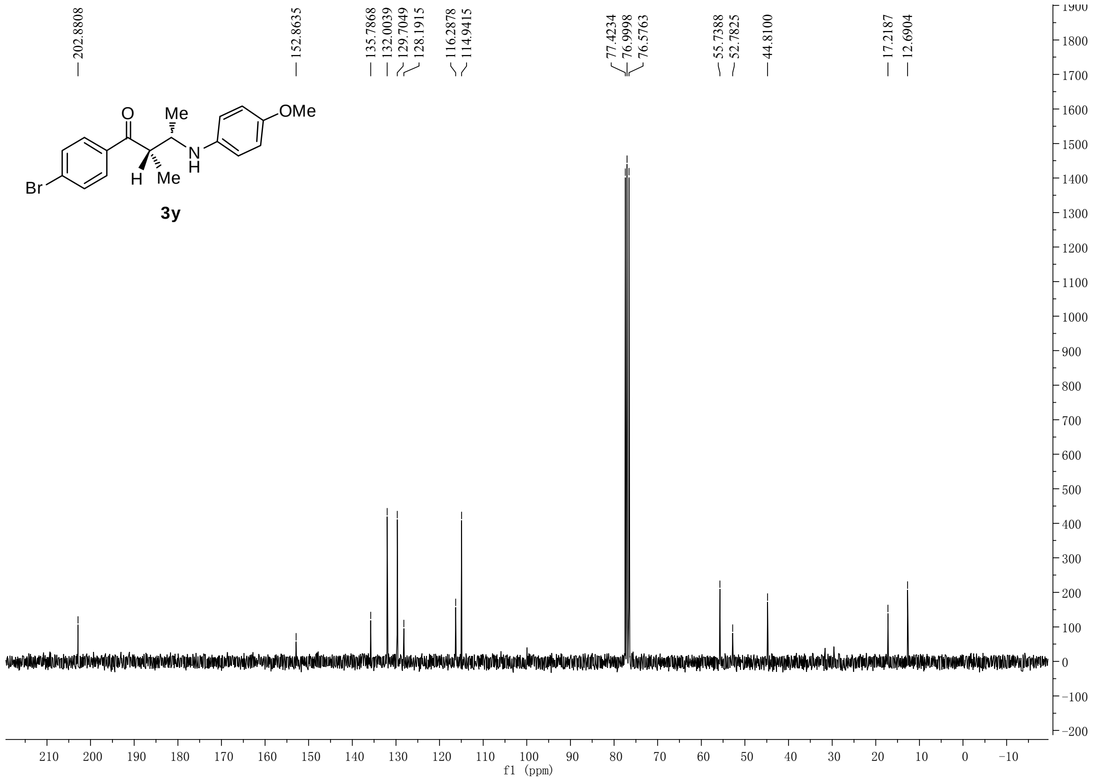

Supplementary Figure 25. ^{1}H and ^{13}C NMR spectra for compound **3y**

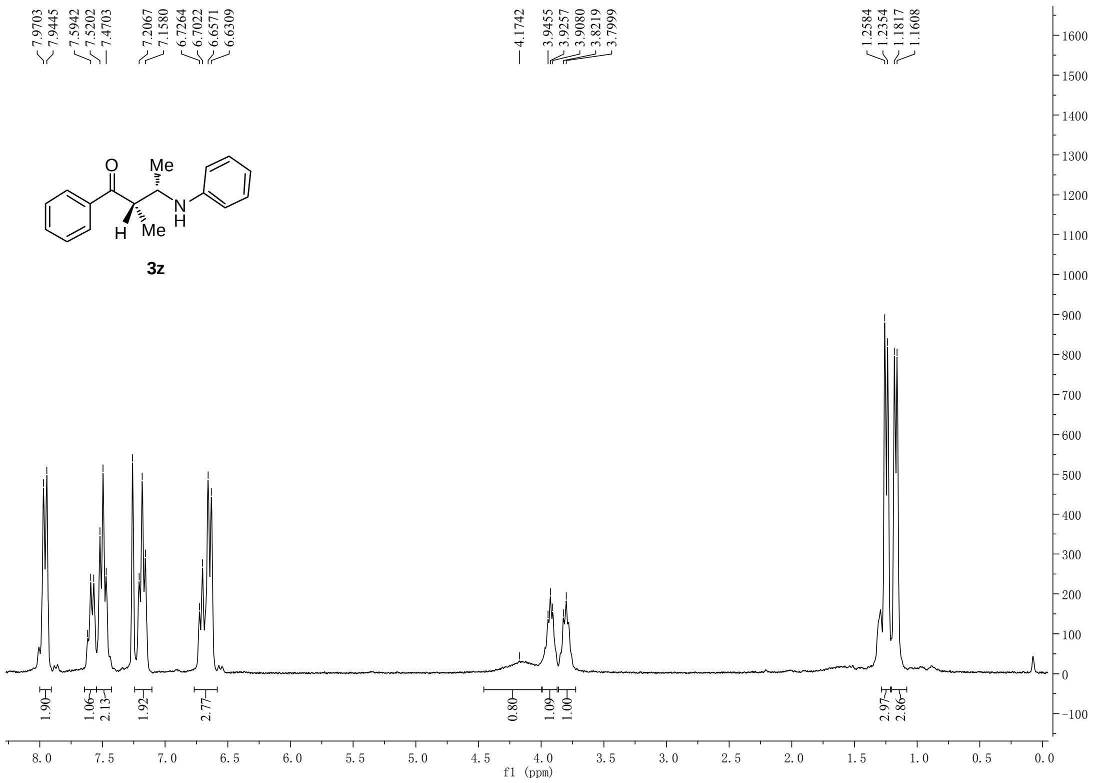

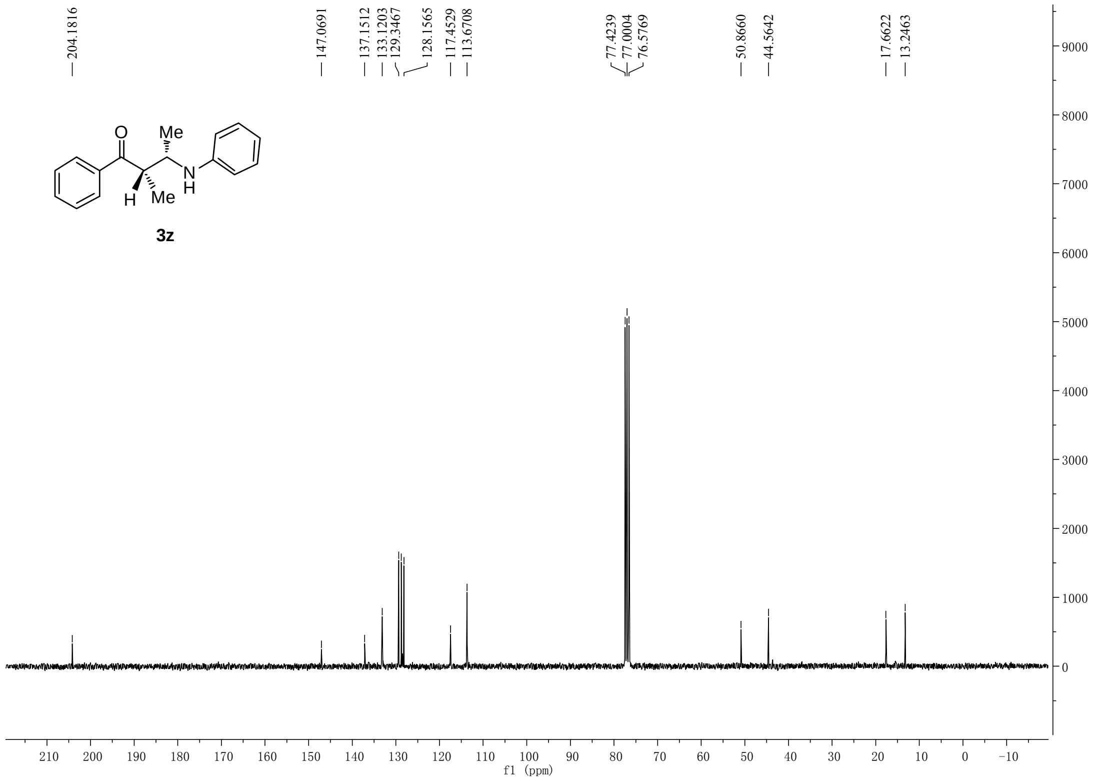

Supplementary Figure 26. ^{1}H and ^{13}C NMR spectra for compound **3z**

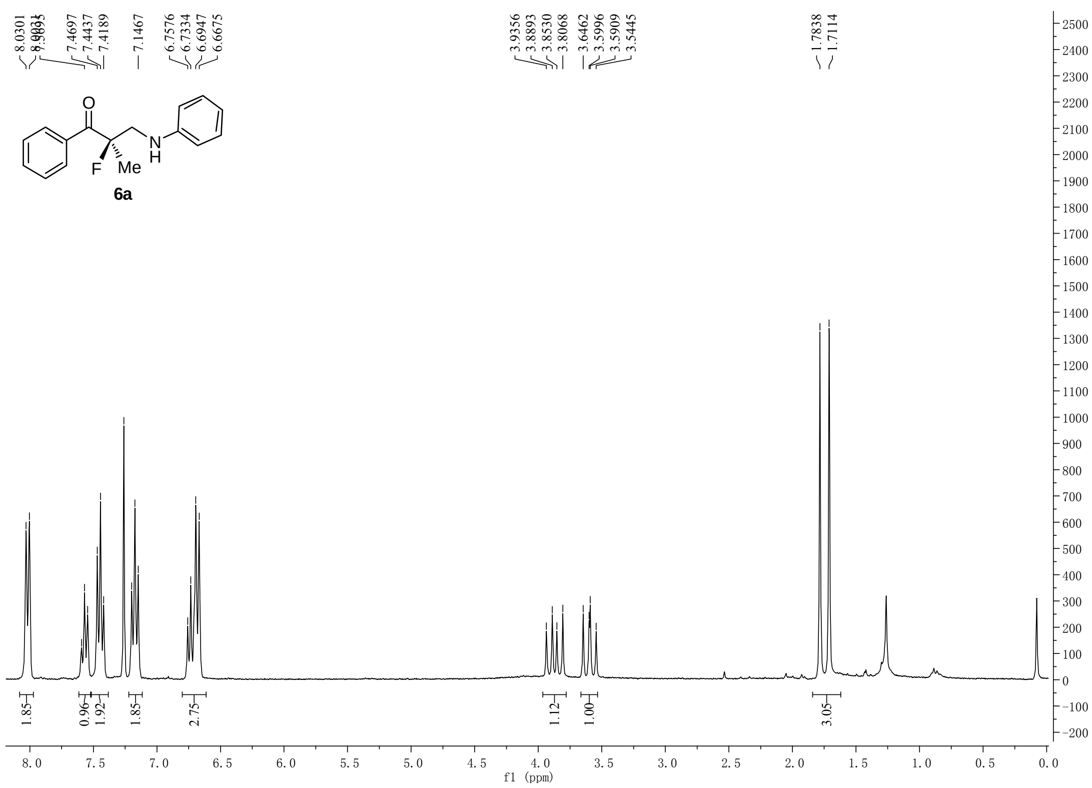

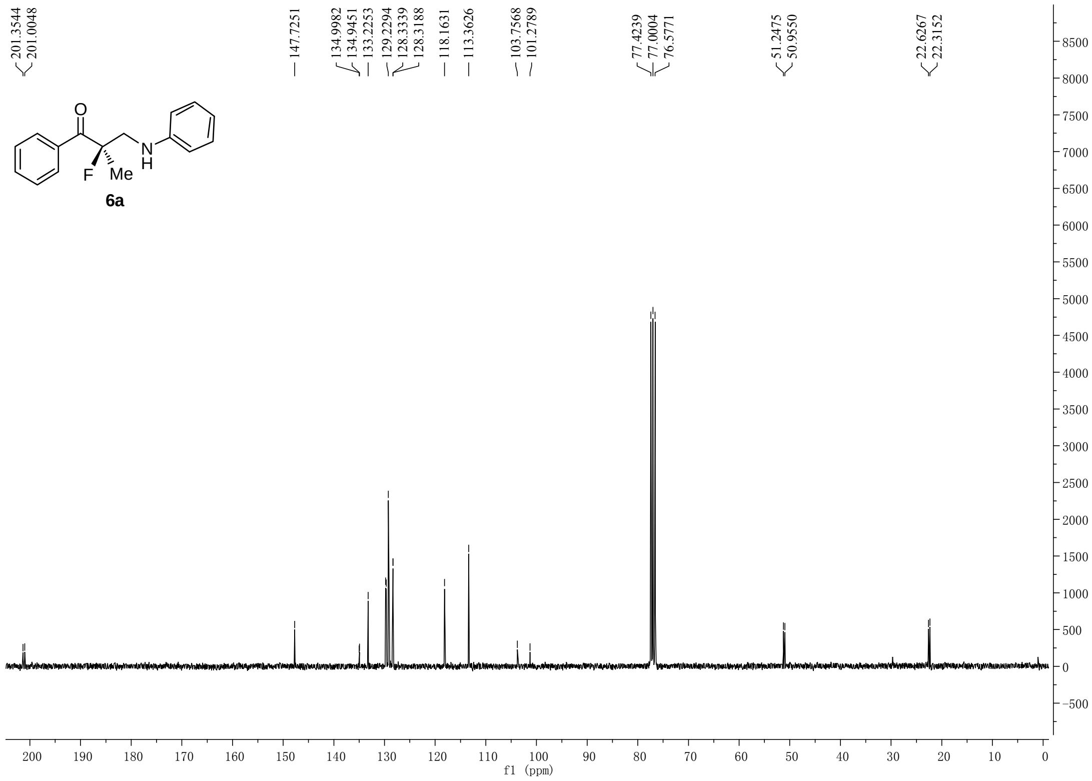

Supplementary Figure 27. ^{1}H and ^{13}C NMR spectra for compound **6a**

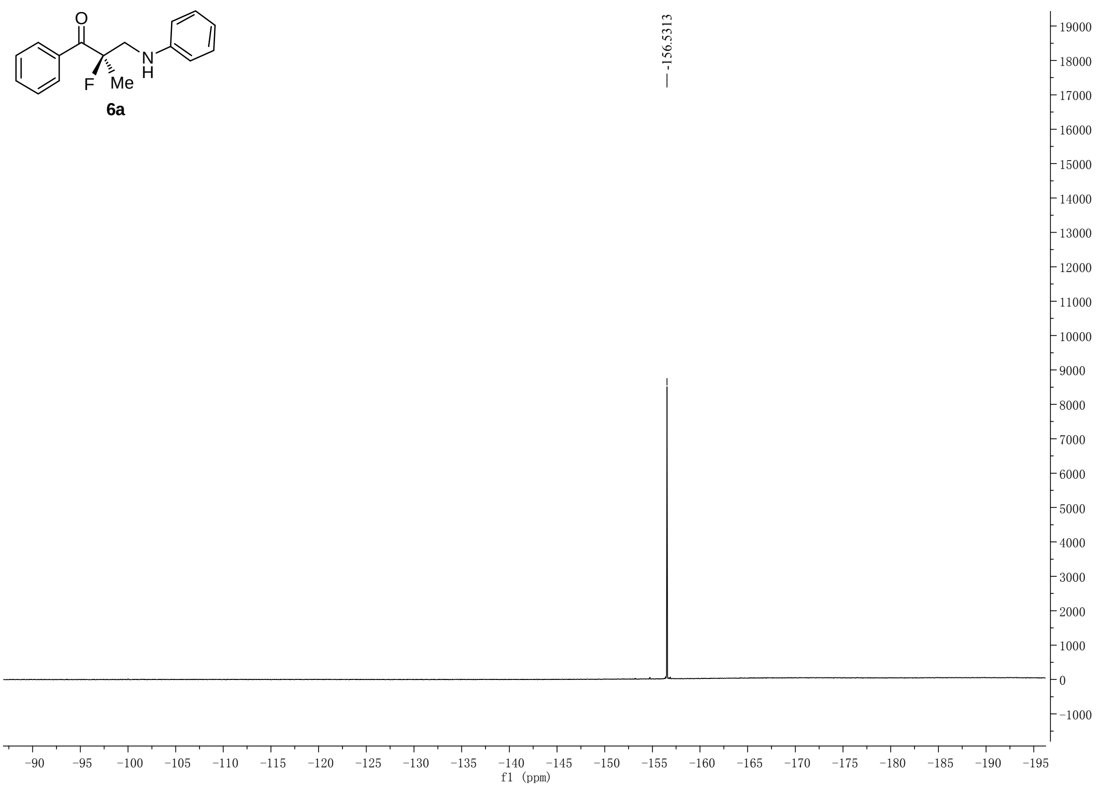

Supplementary Figure 28. ^{19}F NMR spectra for compound **6a**

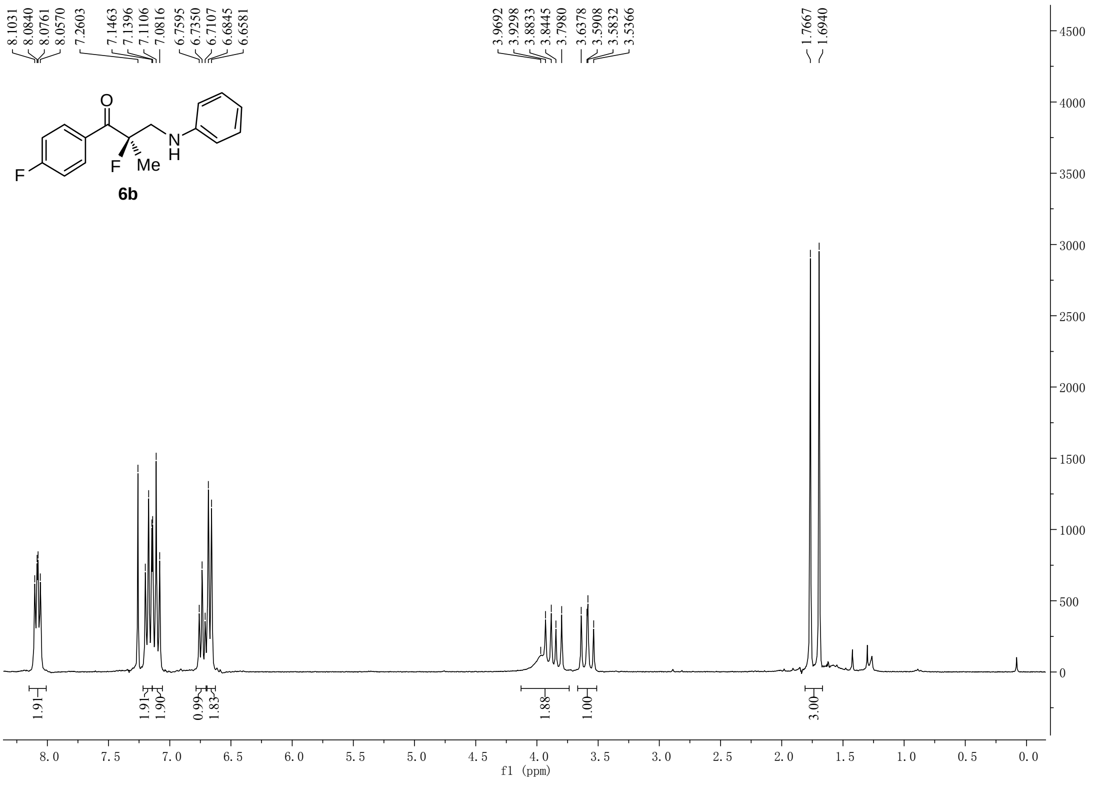

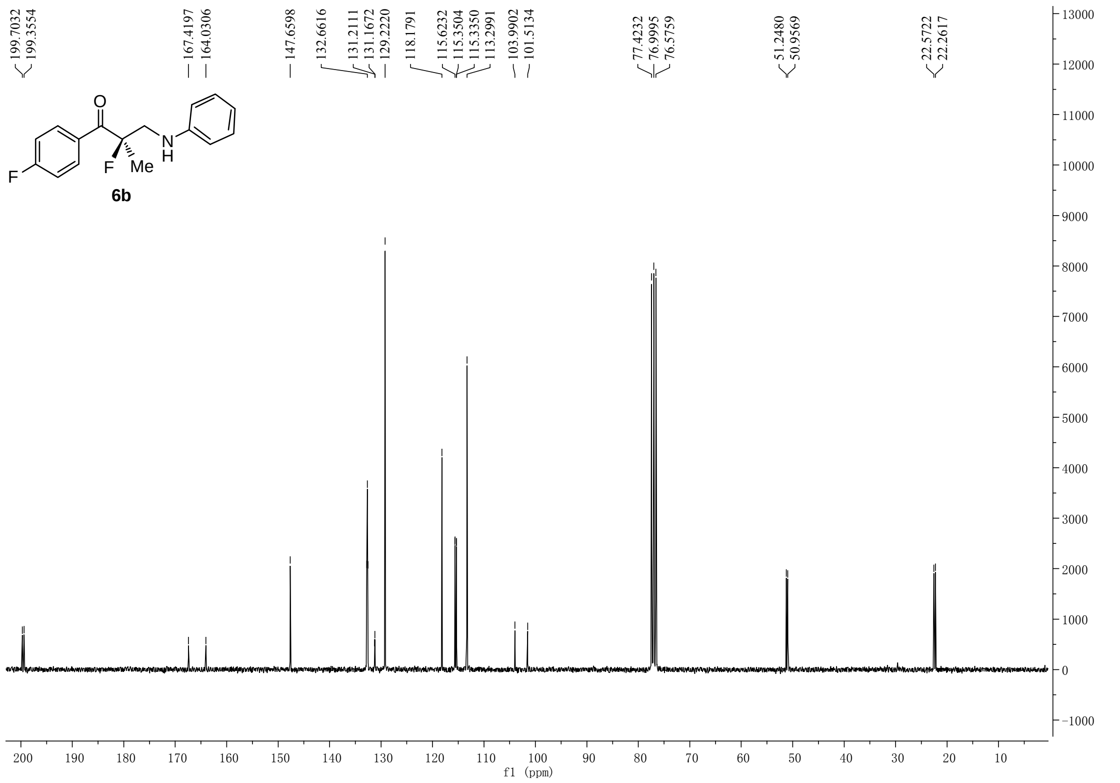

Supplementary Figure 29. ^{1}H and ^{13}C NMR spectra for compound **6b**

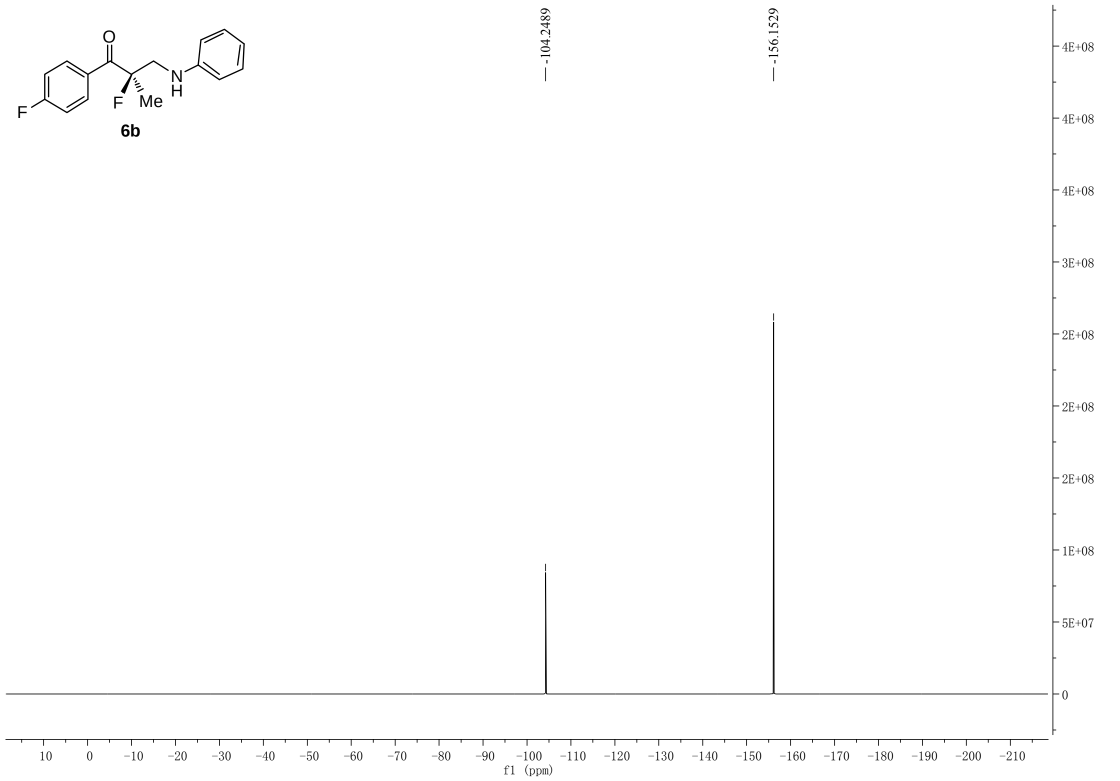

Supplementary Figure 30. ^{19}F NMR spectra for compound **6b**

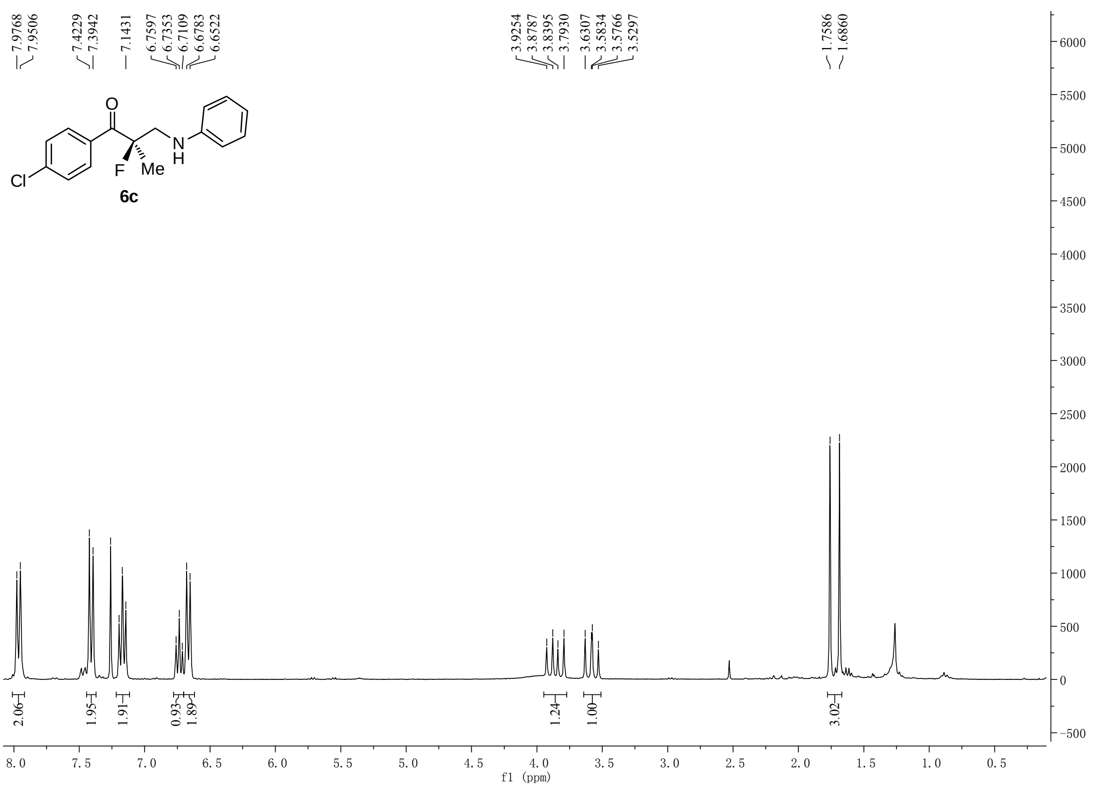

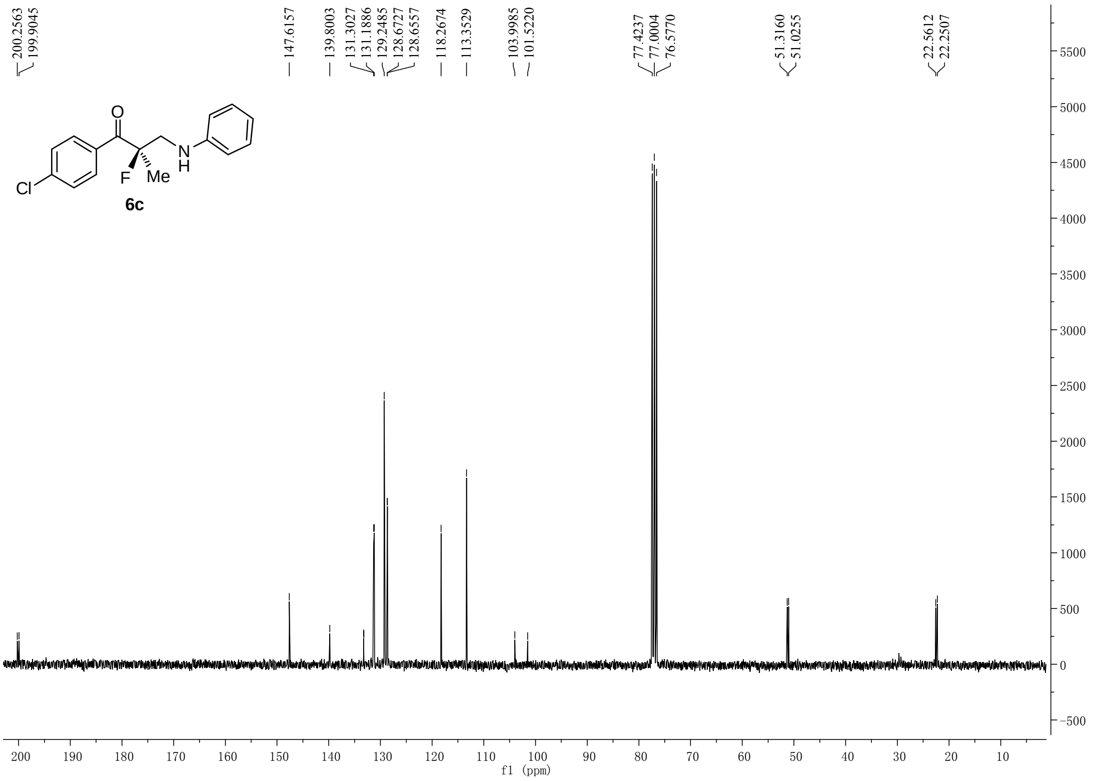

Supplementary Figure 31. ^{1}H and ^{13}C NMR spectra for compound **6c**

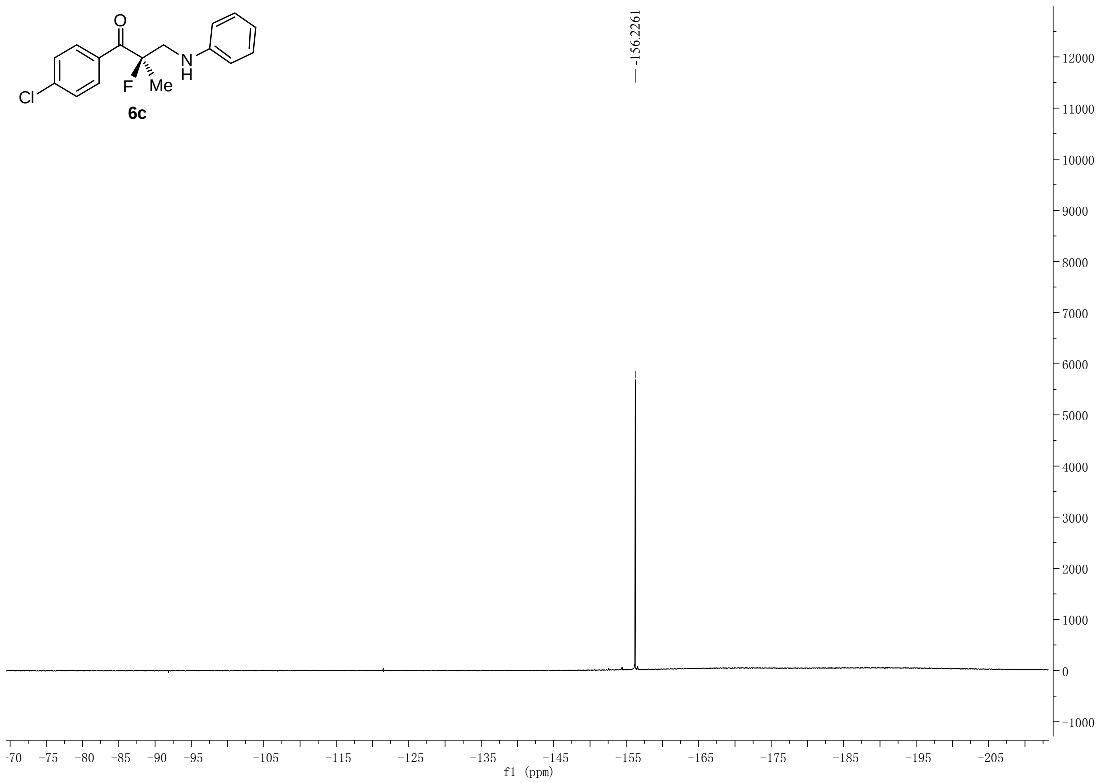

Supplementary Figure 32. ^{19}F NMR spectra for compound **6c**

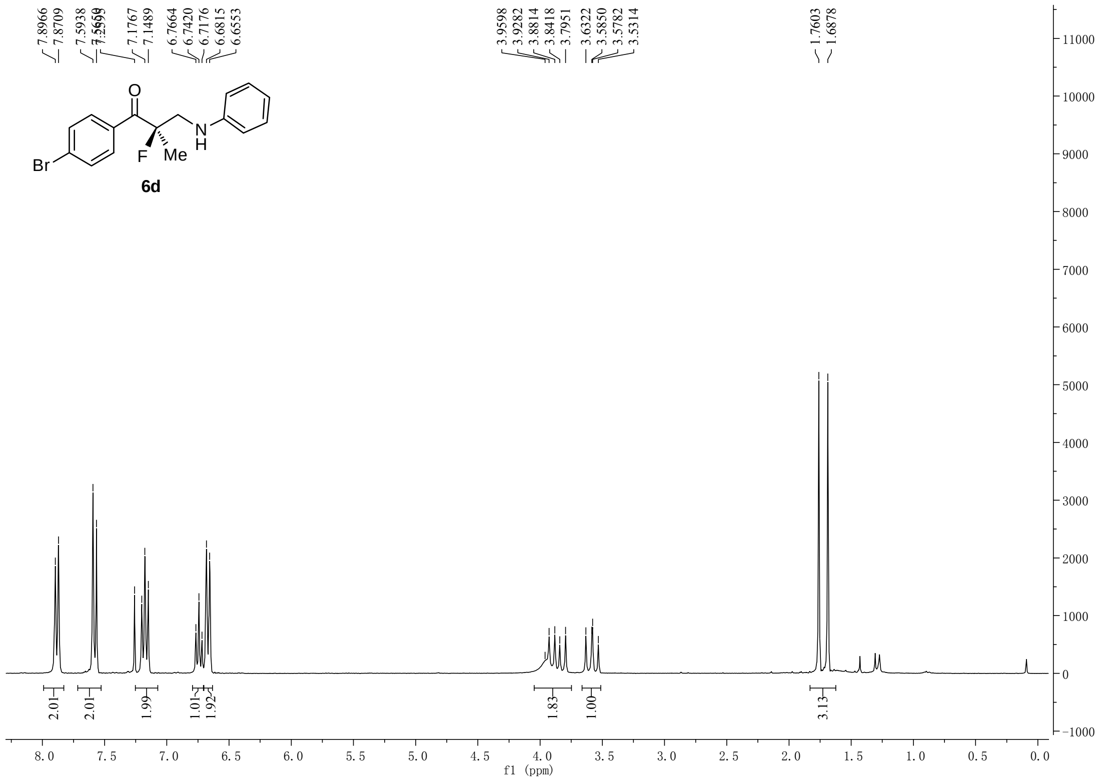

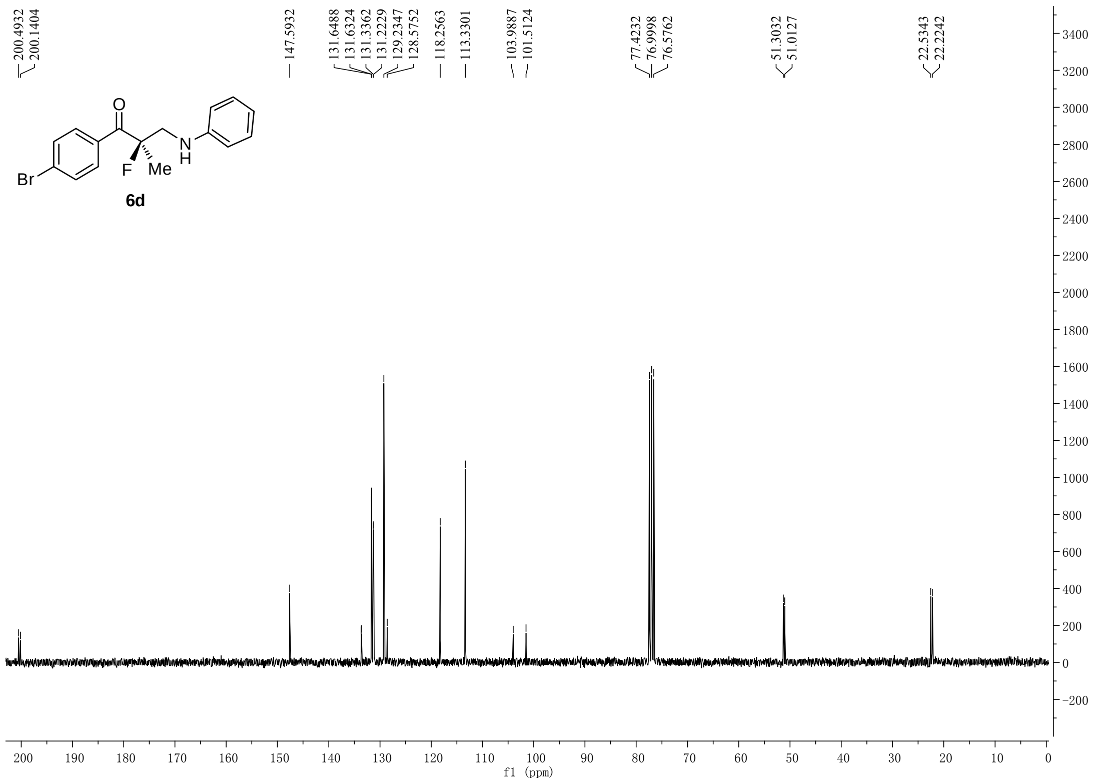

Supplementary Figure 33. ^{1}H and ^{13}C NMR spectra for compound **6d**

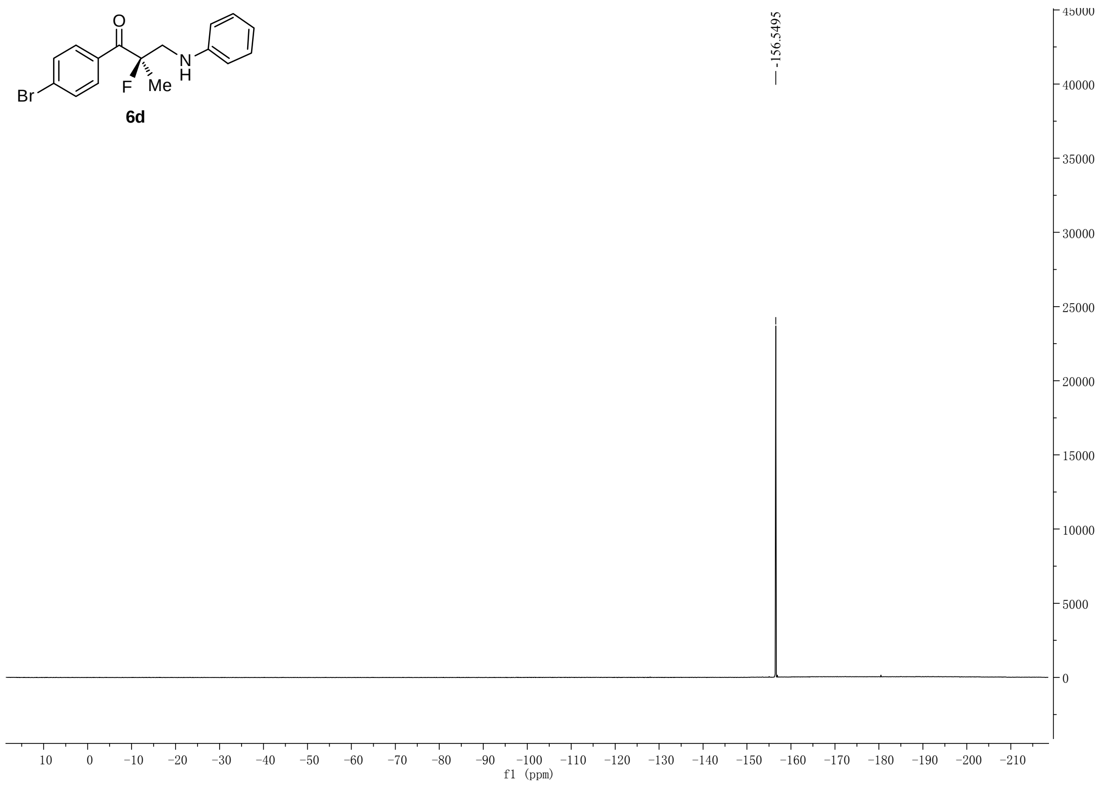

Supplementary Figure 34. ^{19}F NMR spectra for compound **6d**

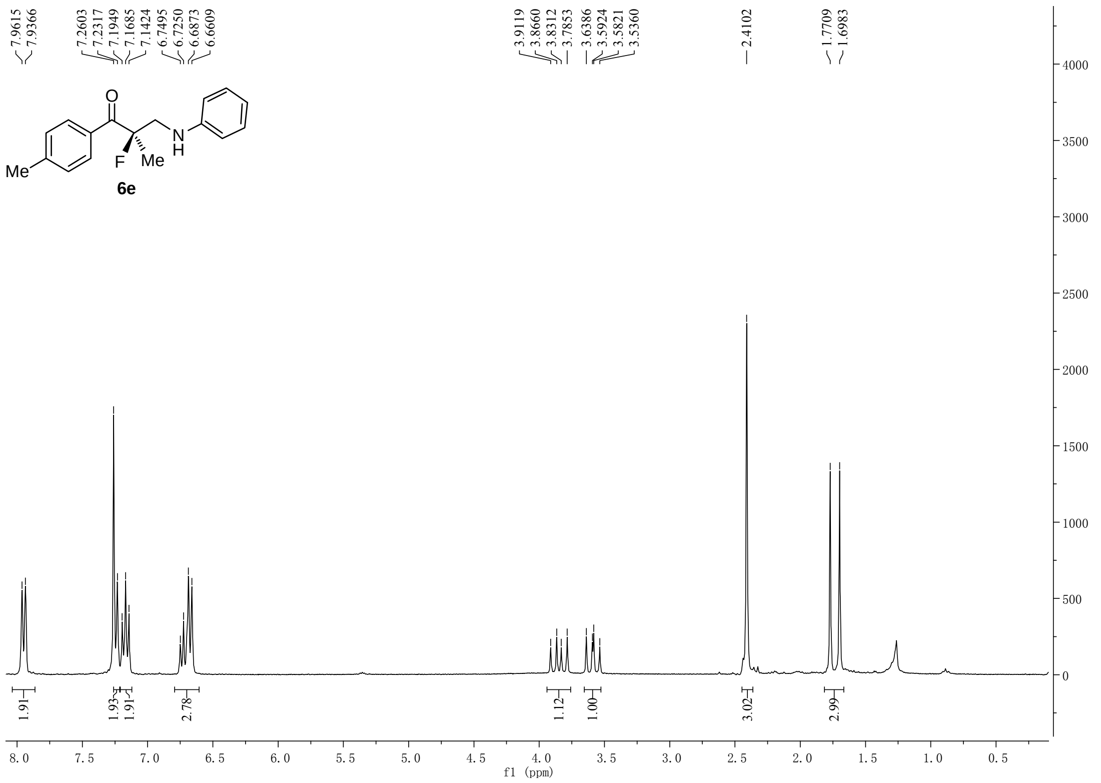

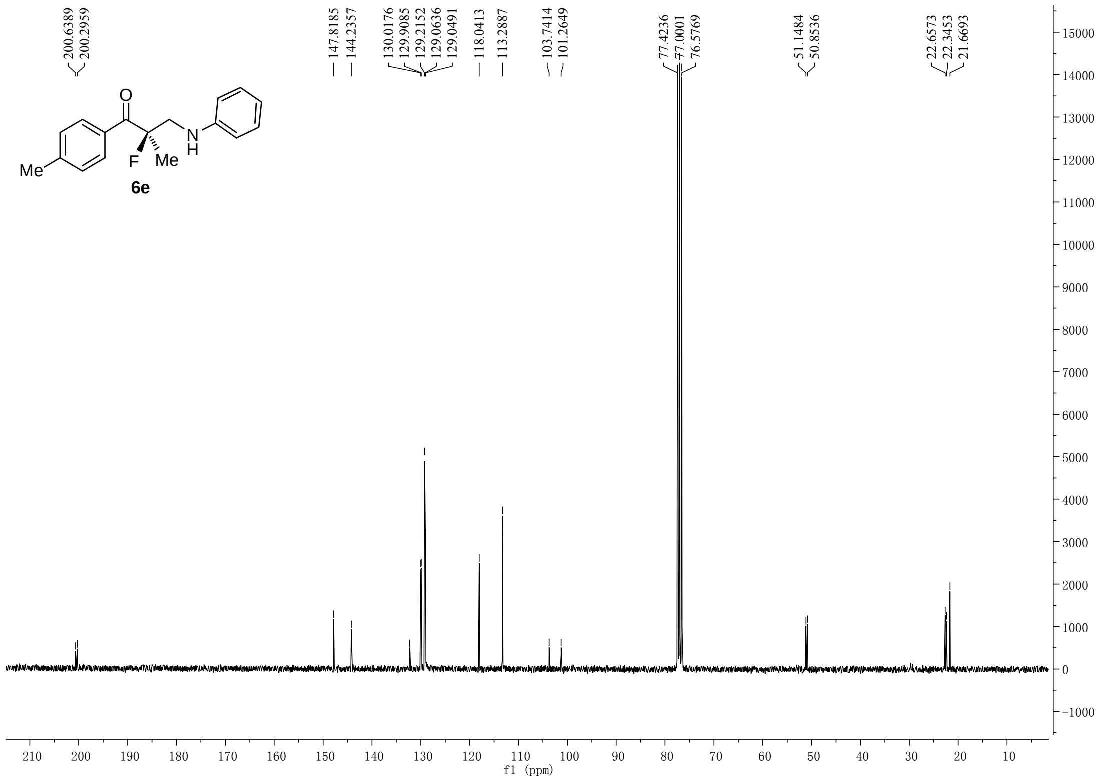

Supplementary Figure 35. ^{1}H and ^{13}C NMR spectra for compound **6e**

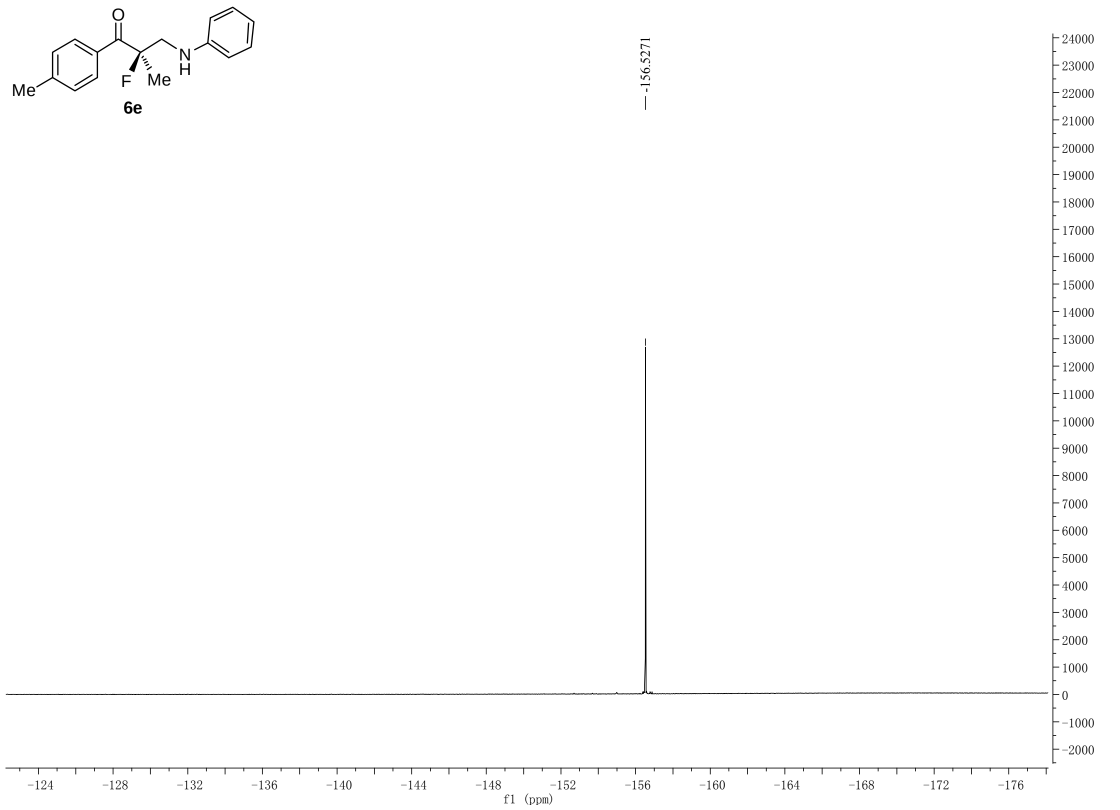

Supplementary Figure 36. ^{19}F NMR spectra for compound **6e**

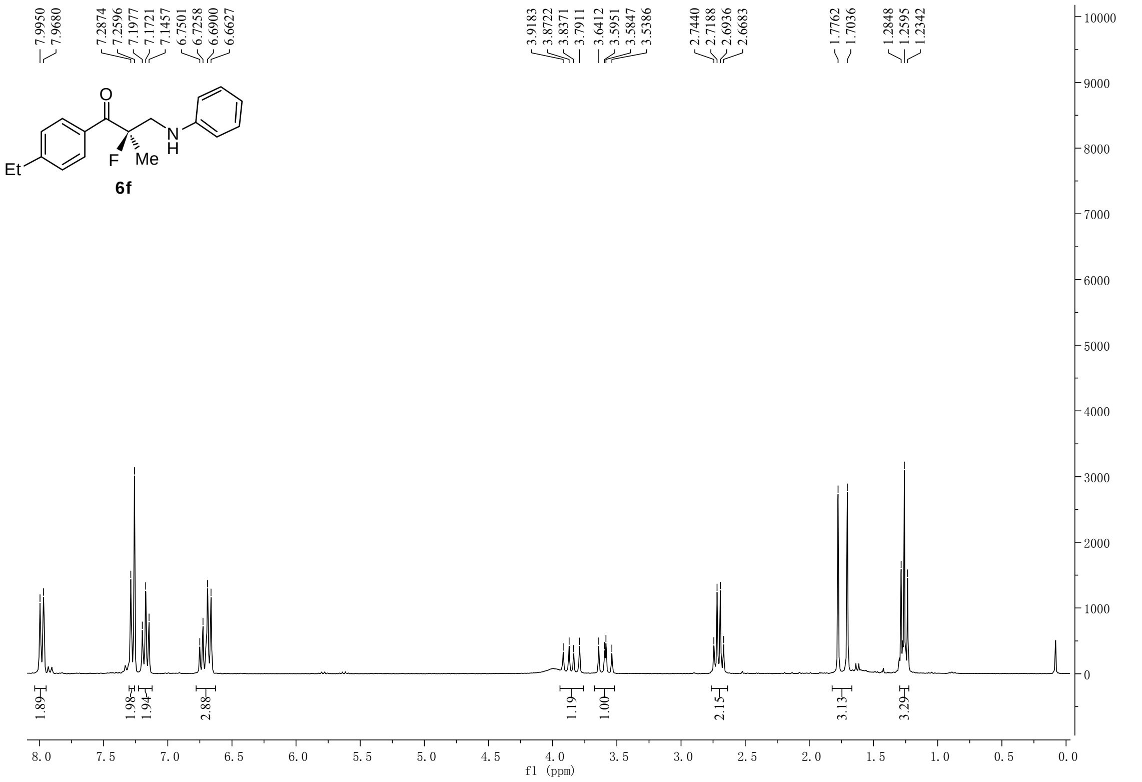

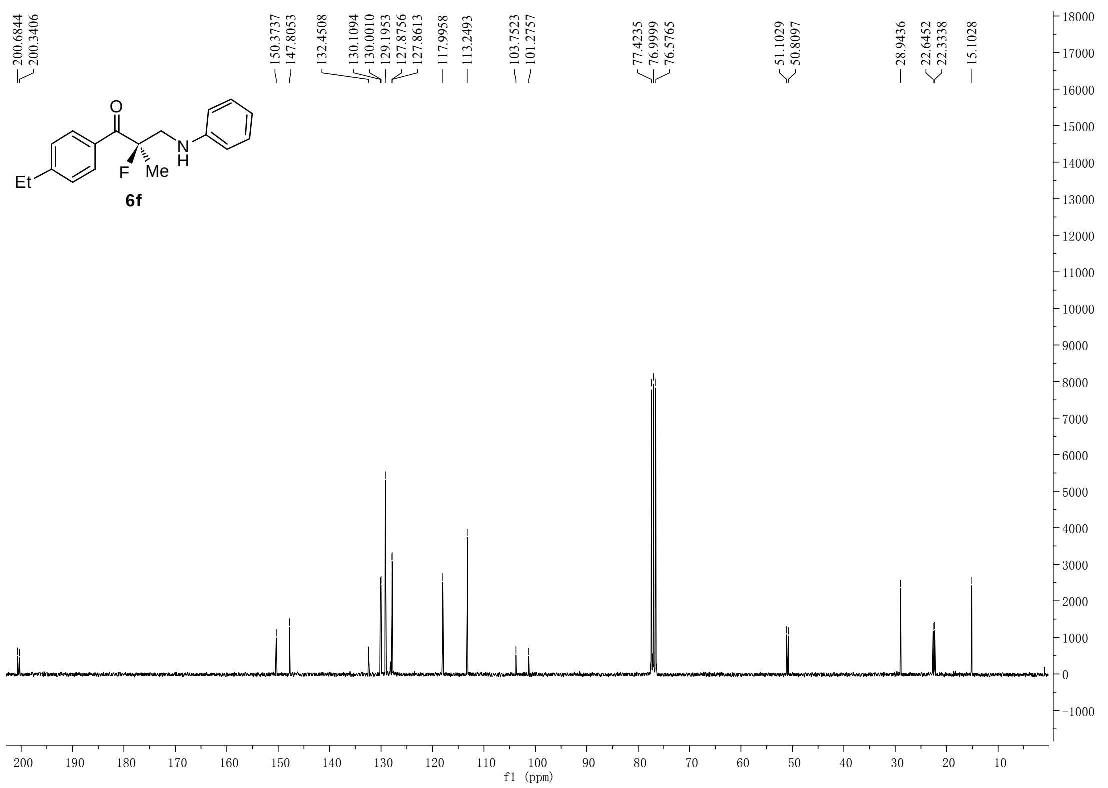

Supplementary Figure 37. ^{1}H and ^{13}C NMR spectra for compound **6f**

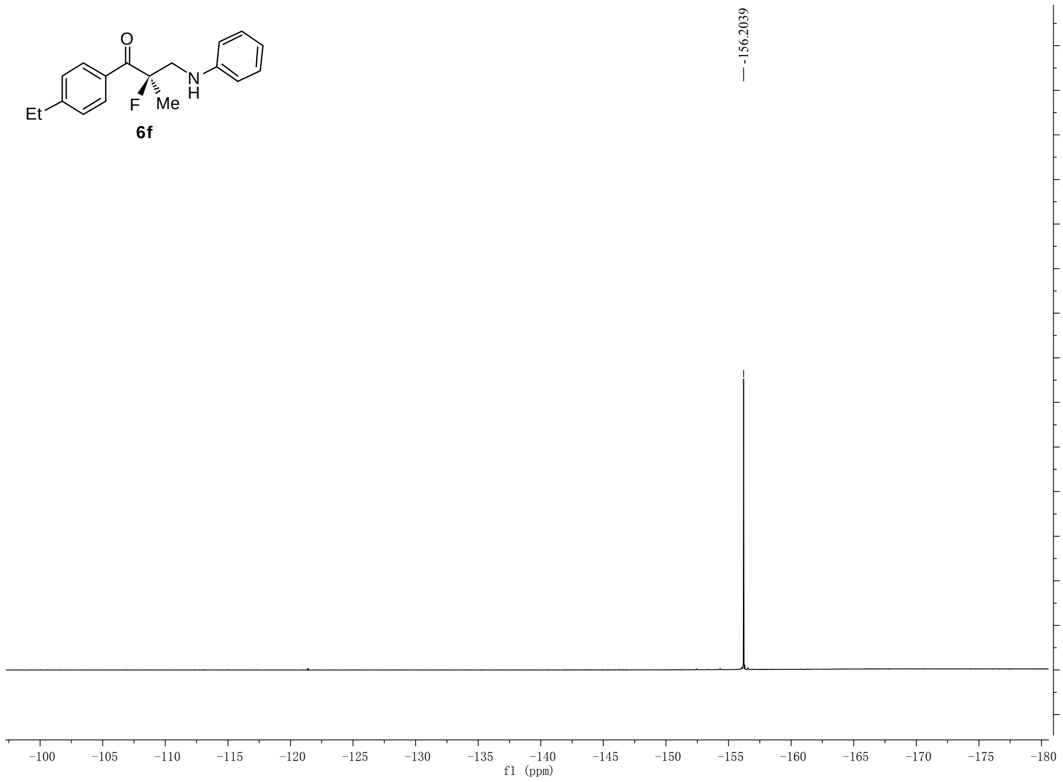

Supplementary Figure 38. ^{19}F NMR spectra for compound **6f**

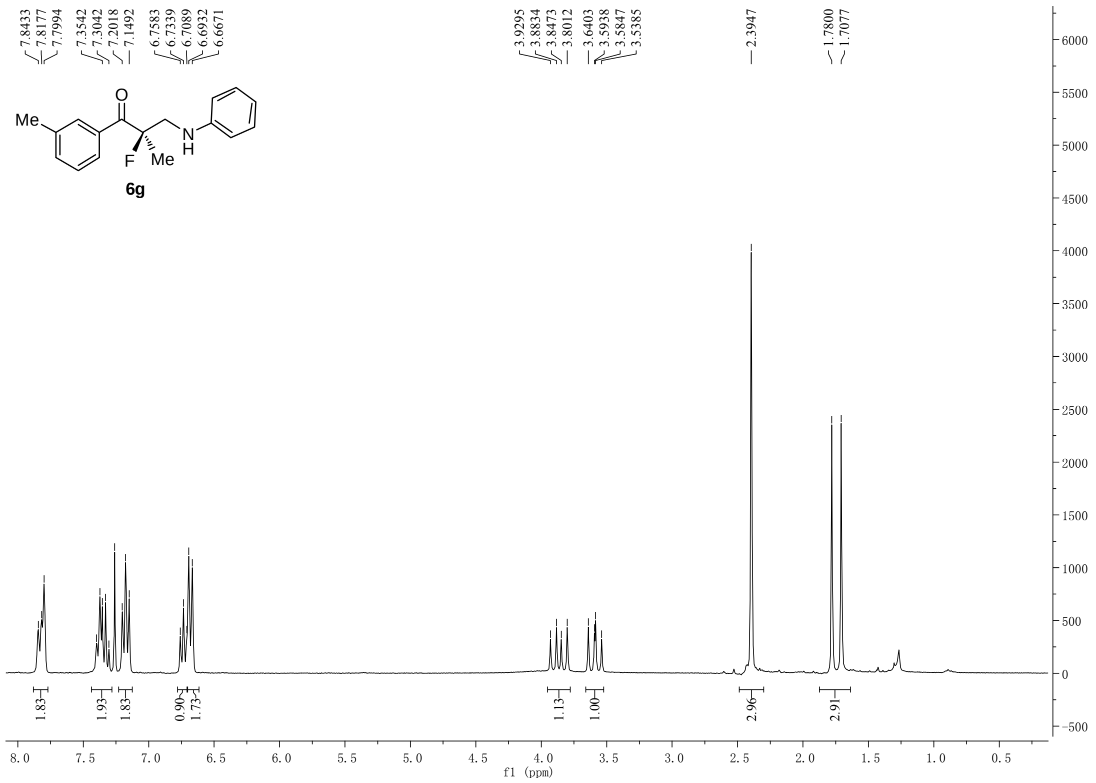

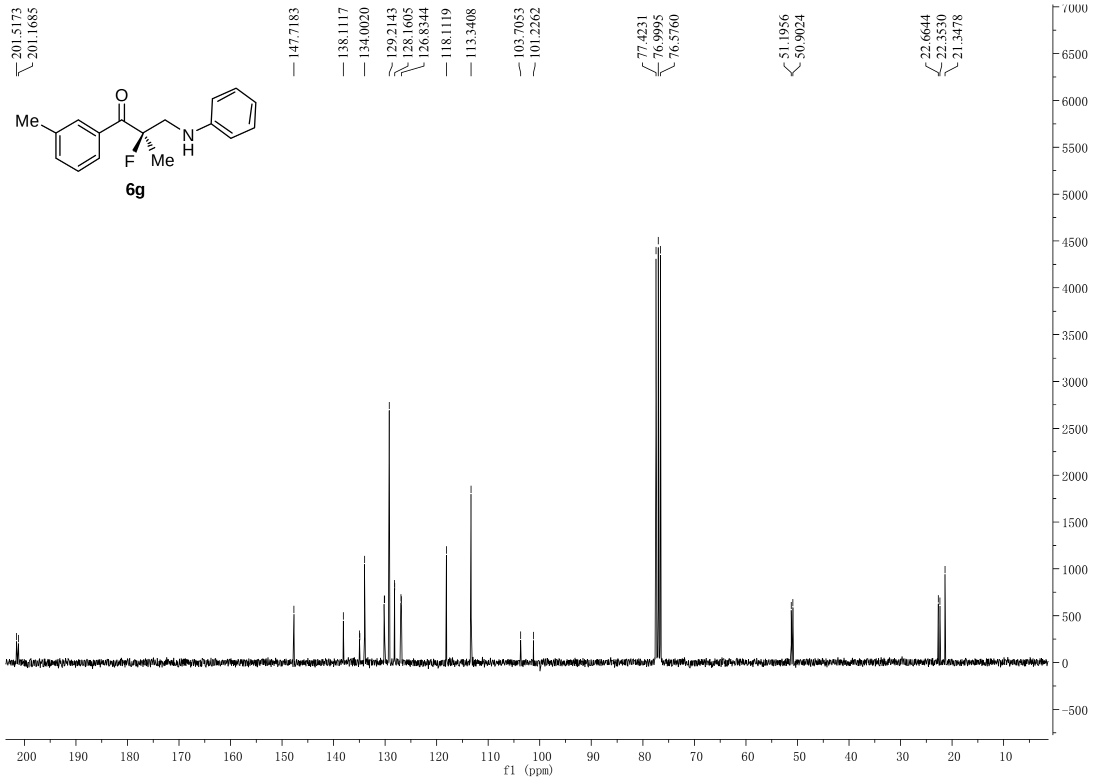

Supplementary Figure 39. ^{1}H and ^{13}C NMR spectra for compound **6g**

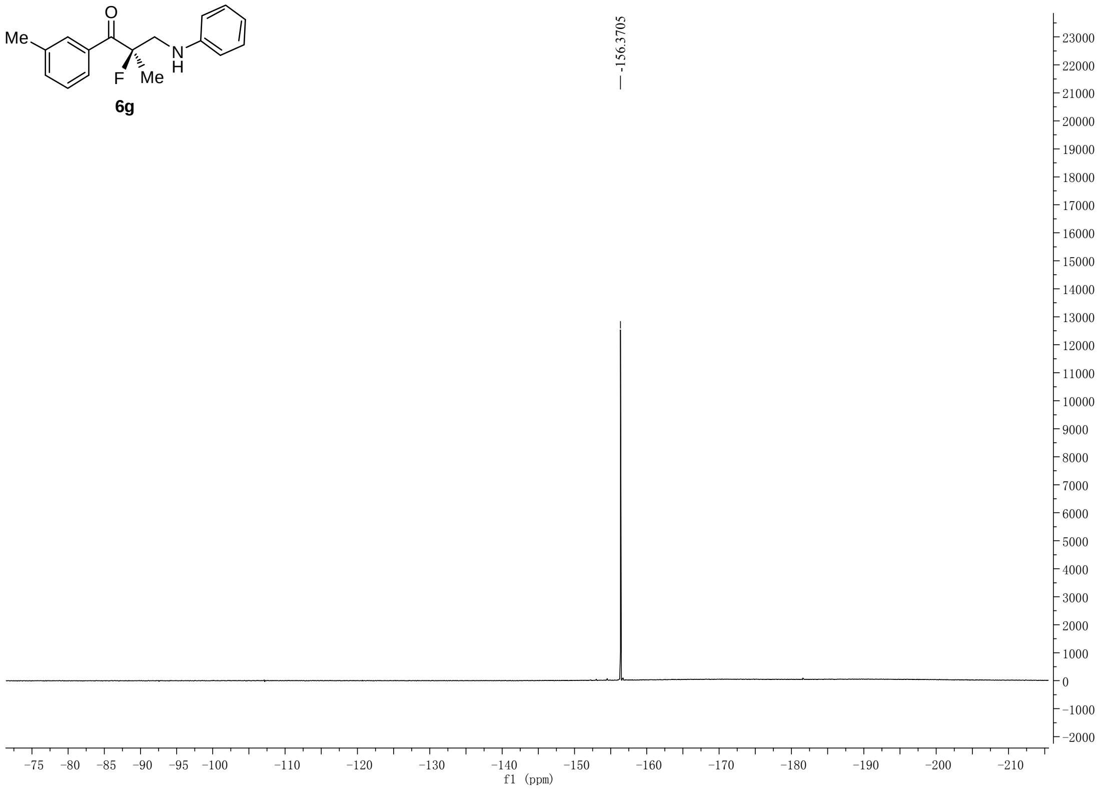

Supplementary Figure 40. ^{19}F NMR spectra for compound **6g**

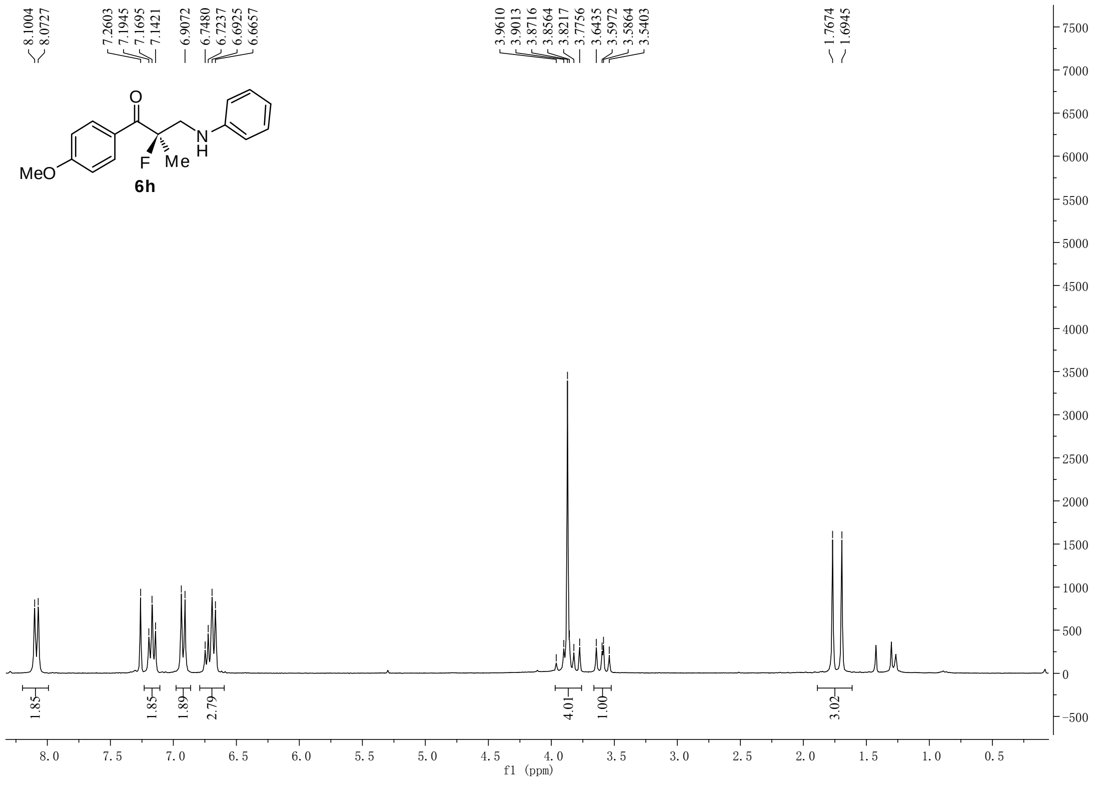

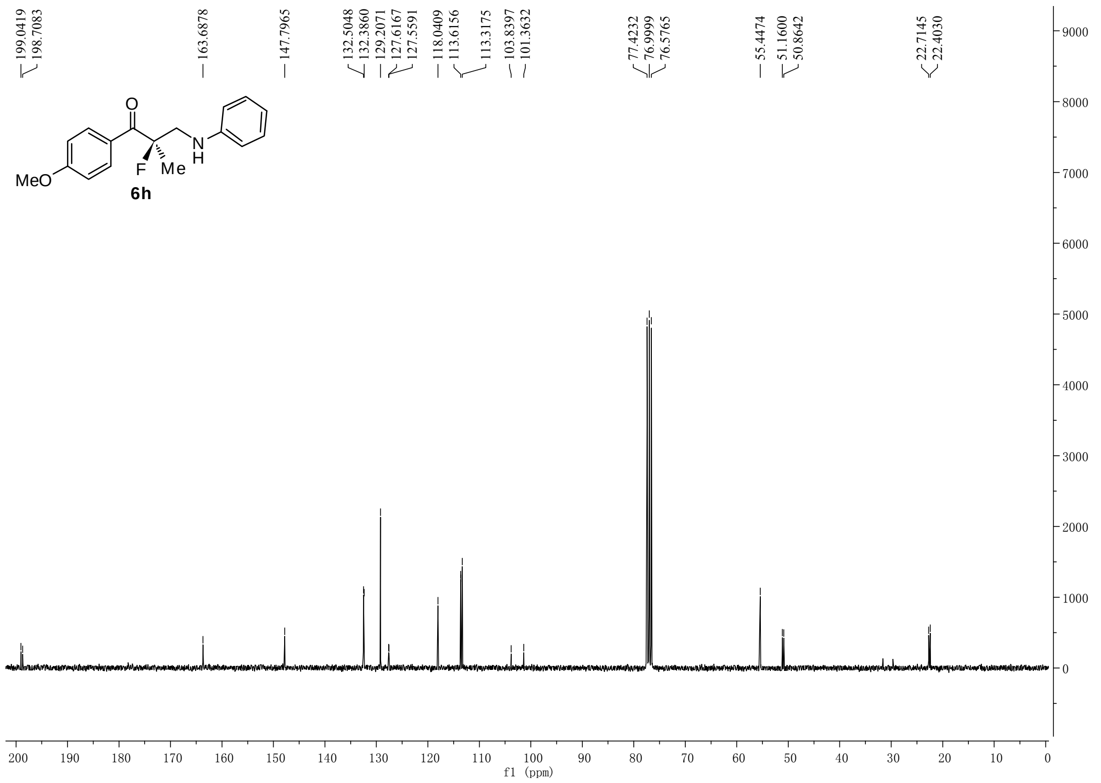

Supplementary Figure 41. ^{1}H and ^{13}C NMR spectra for compound **6h**

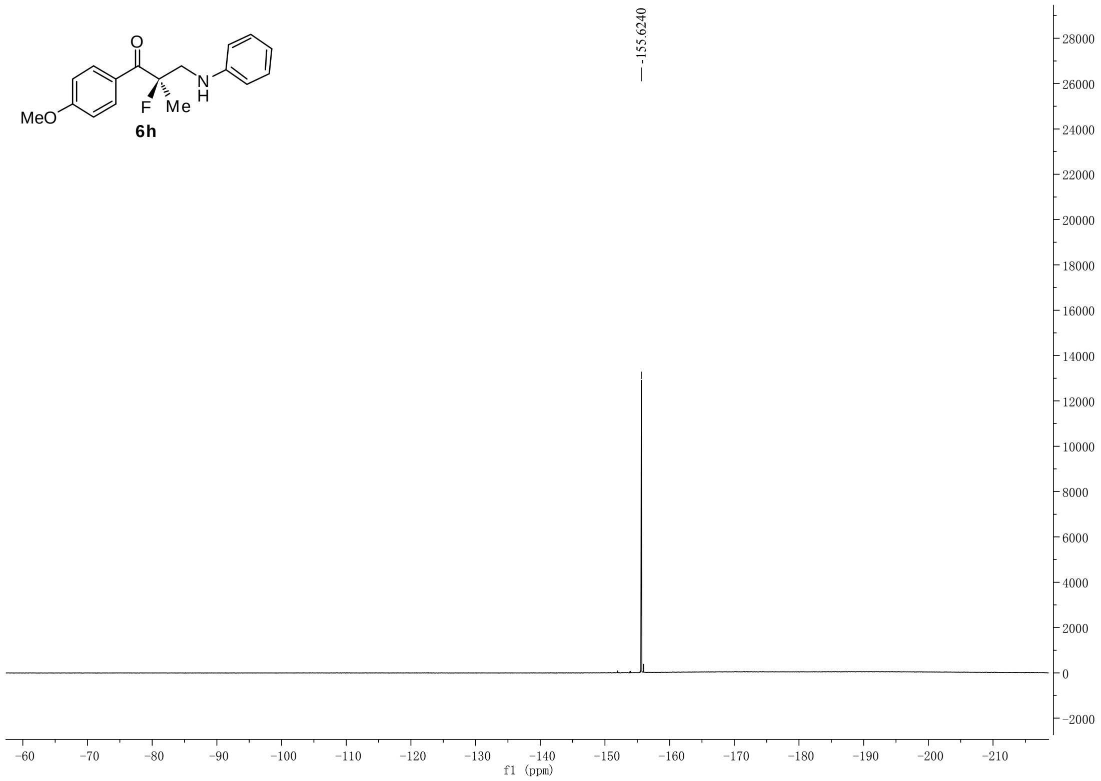

Supplementary Figure 42. ^{19}F NMR spectra for compound **6h**

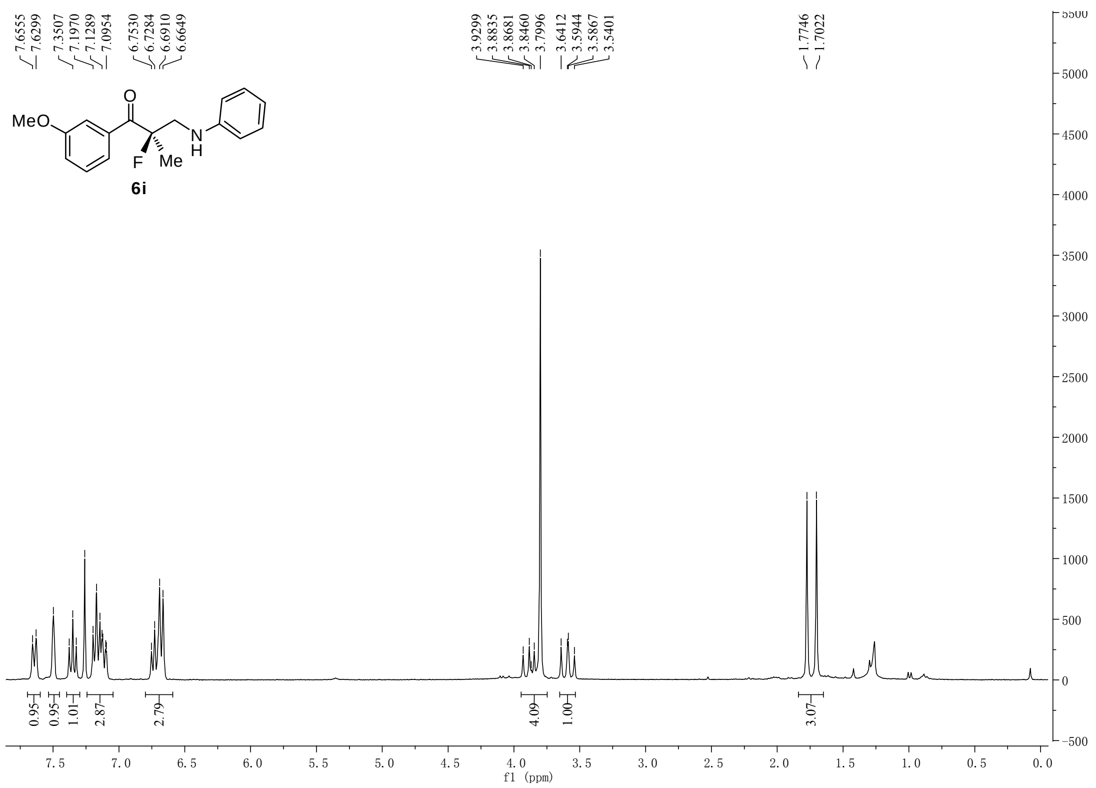

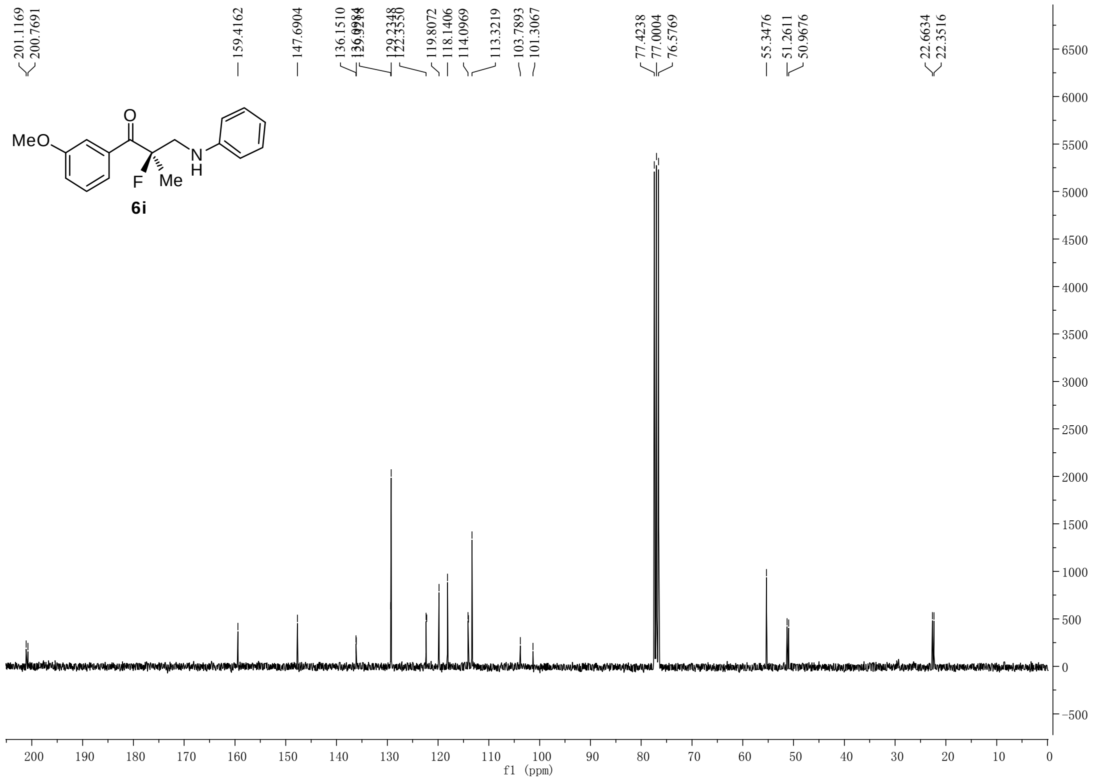

Supplementary Figure 43. ^{1}H and ^{13}C NMR spectra for compound **6i**

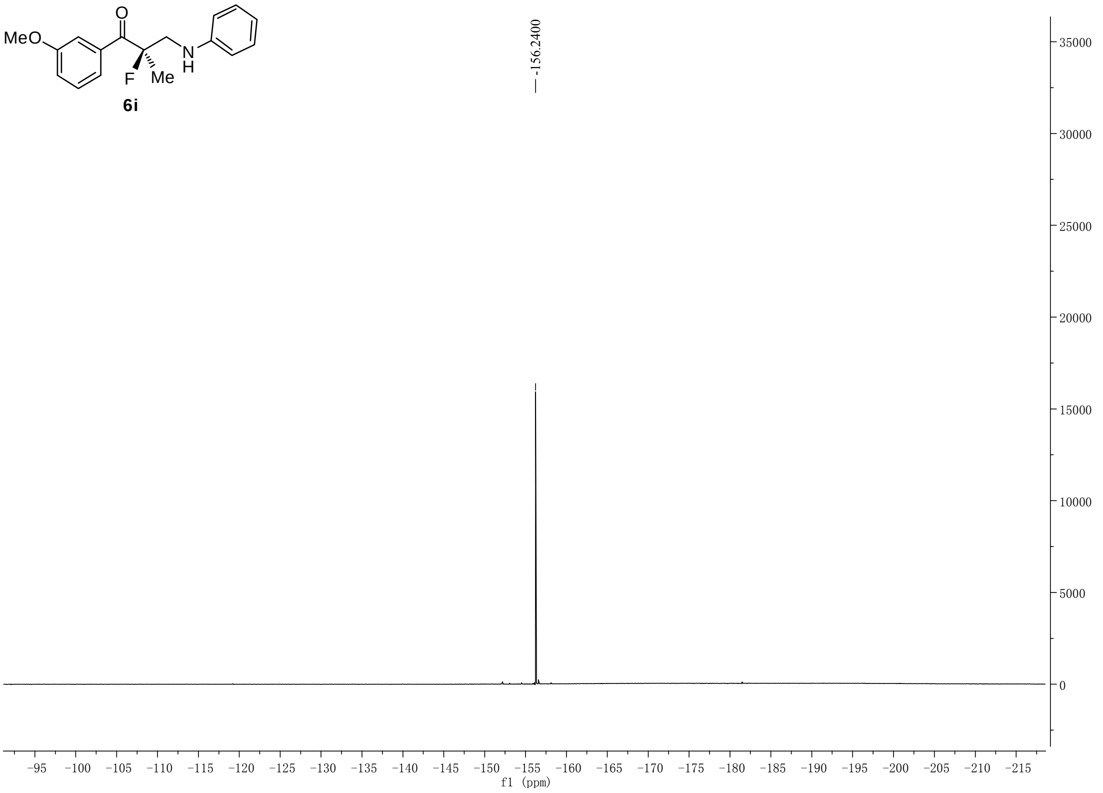

Supplementary Figure 44. ^{19}F NMR spectra for compound **6i**

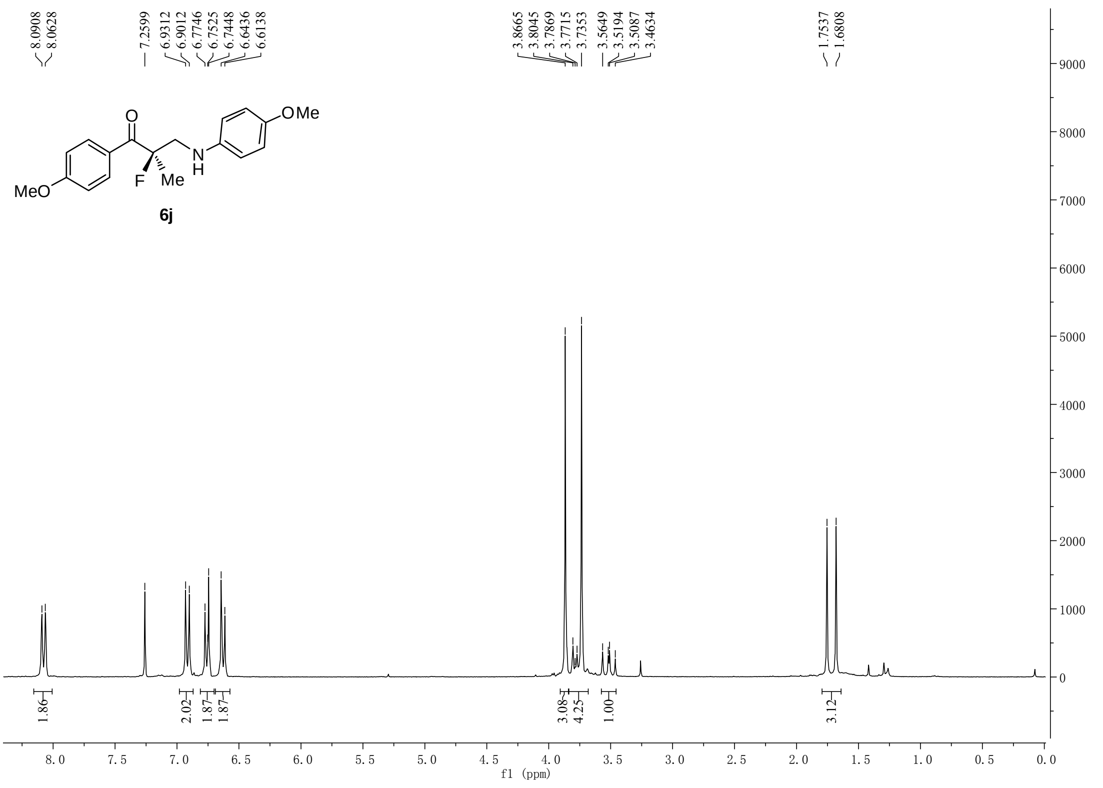

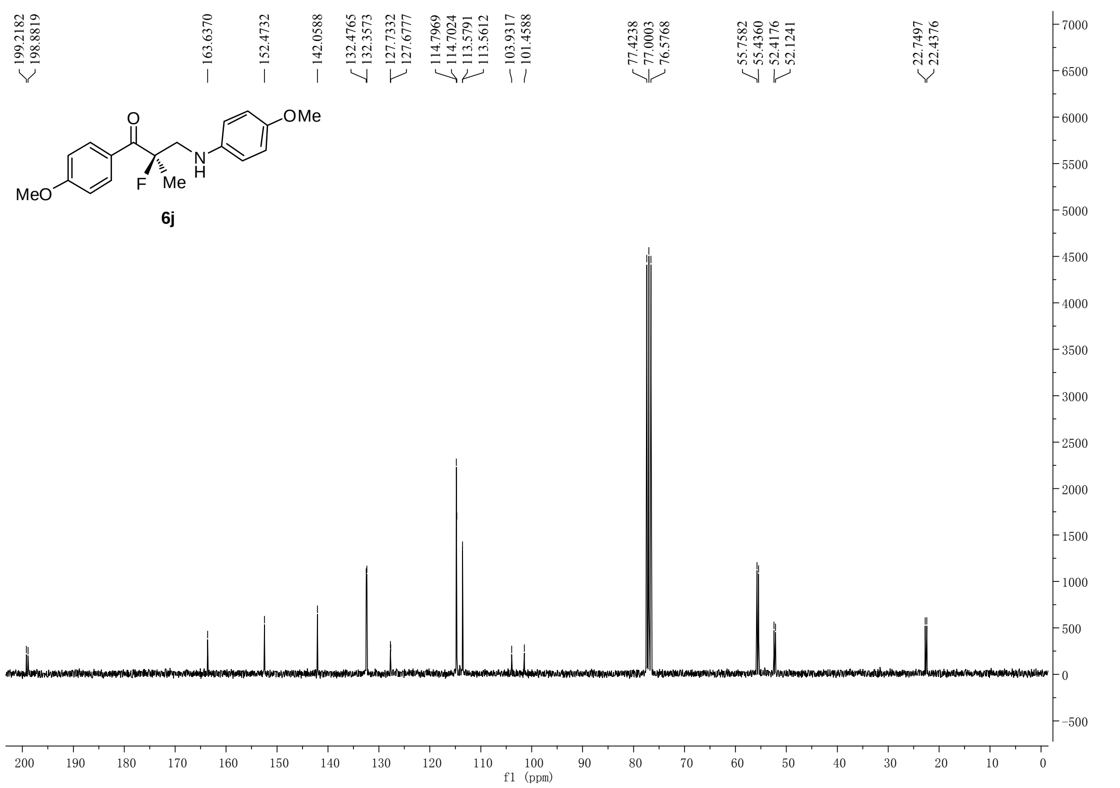

Supplementary Figure 45. ^{1}H and ^{13}C NMR spectra for compound **6j**

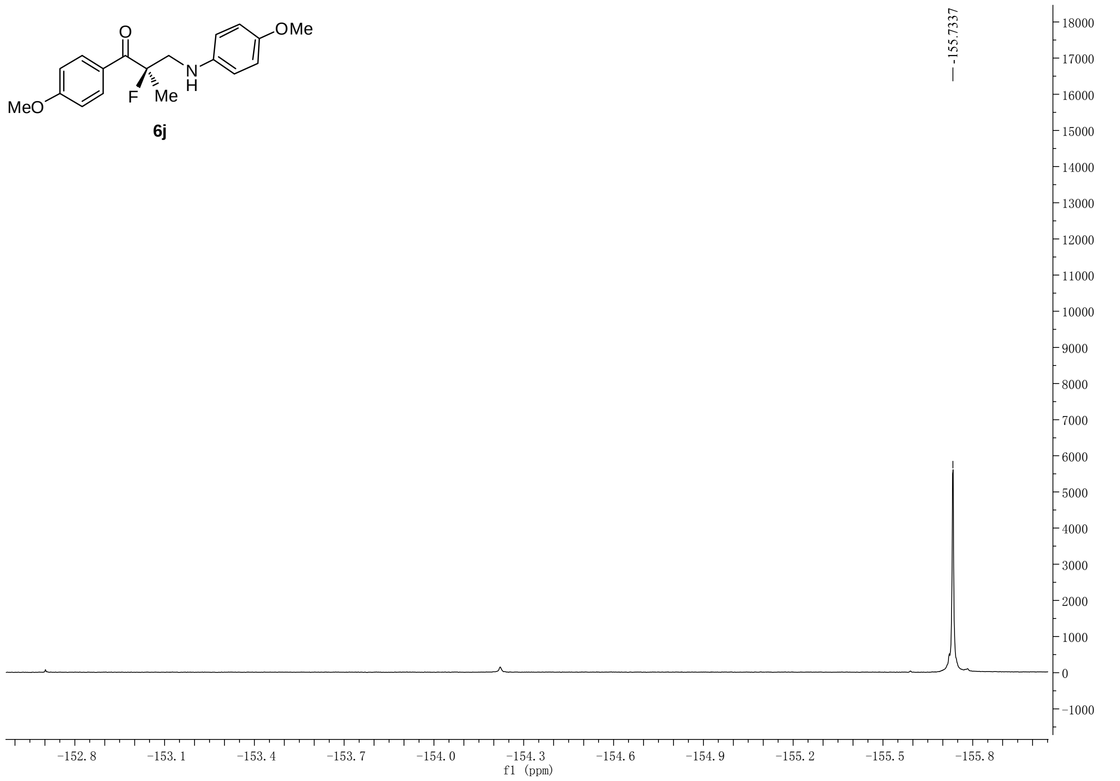

Supplementary Figure 46. ^{19}F NMR spectra for compound **6j**

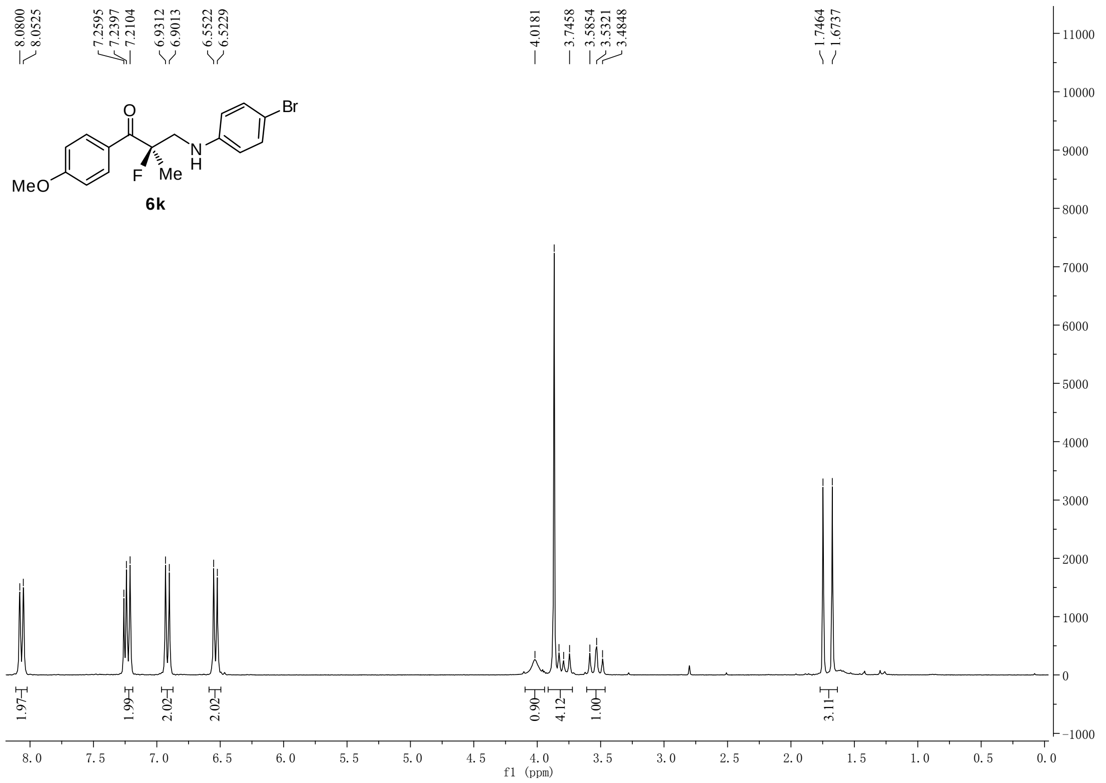

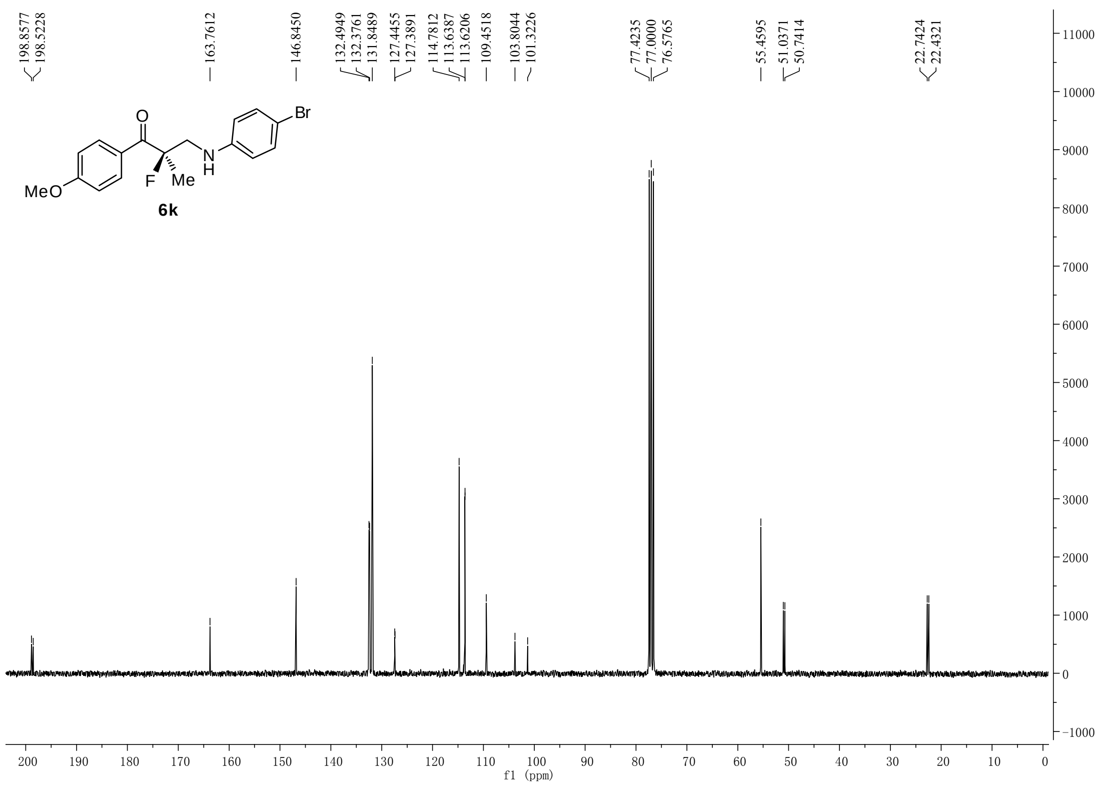

Supplementary Figure 47. ^{1}H and ^{13}C NMR spectra for compound **6k**

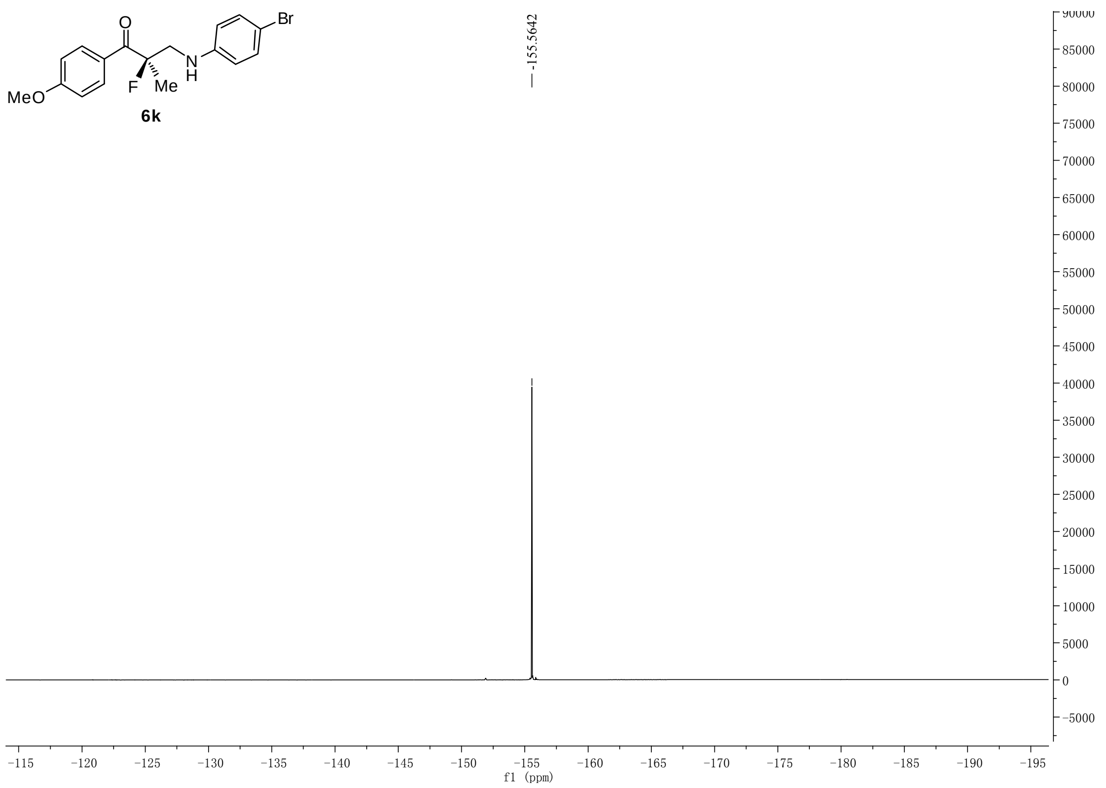

Supplementary Figure 48. ^{19}F NMR spectra for compound **6k**

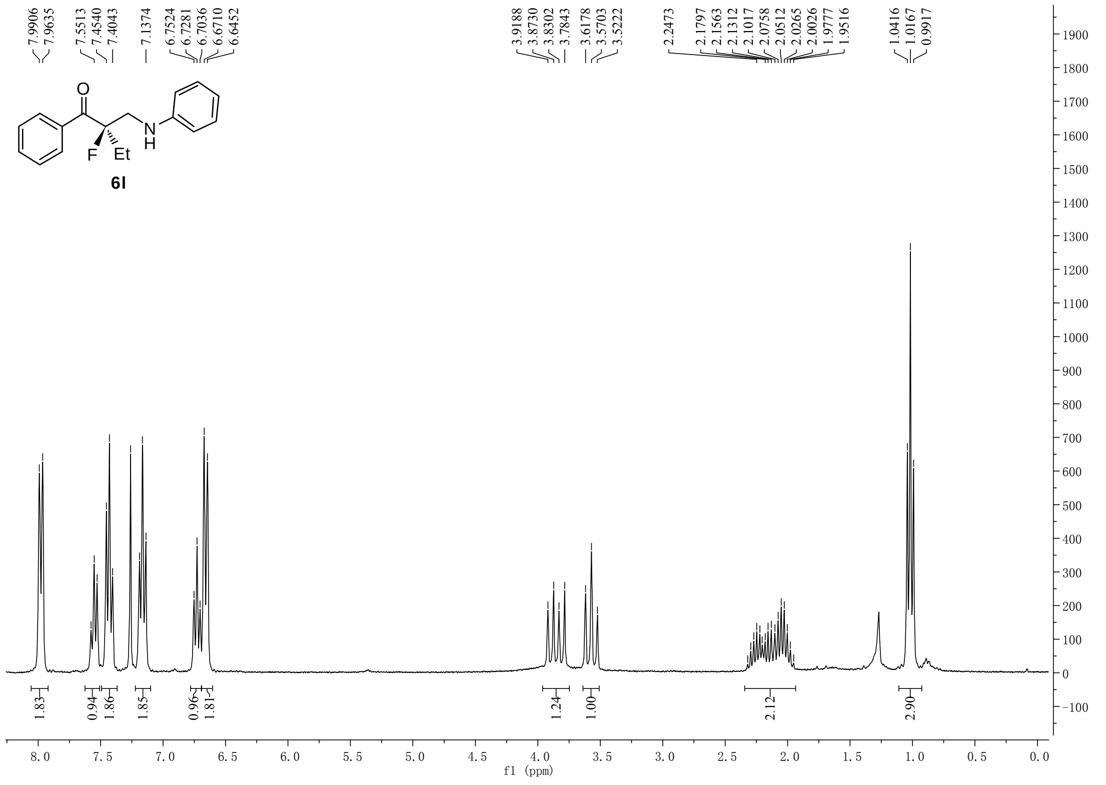

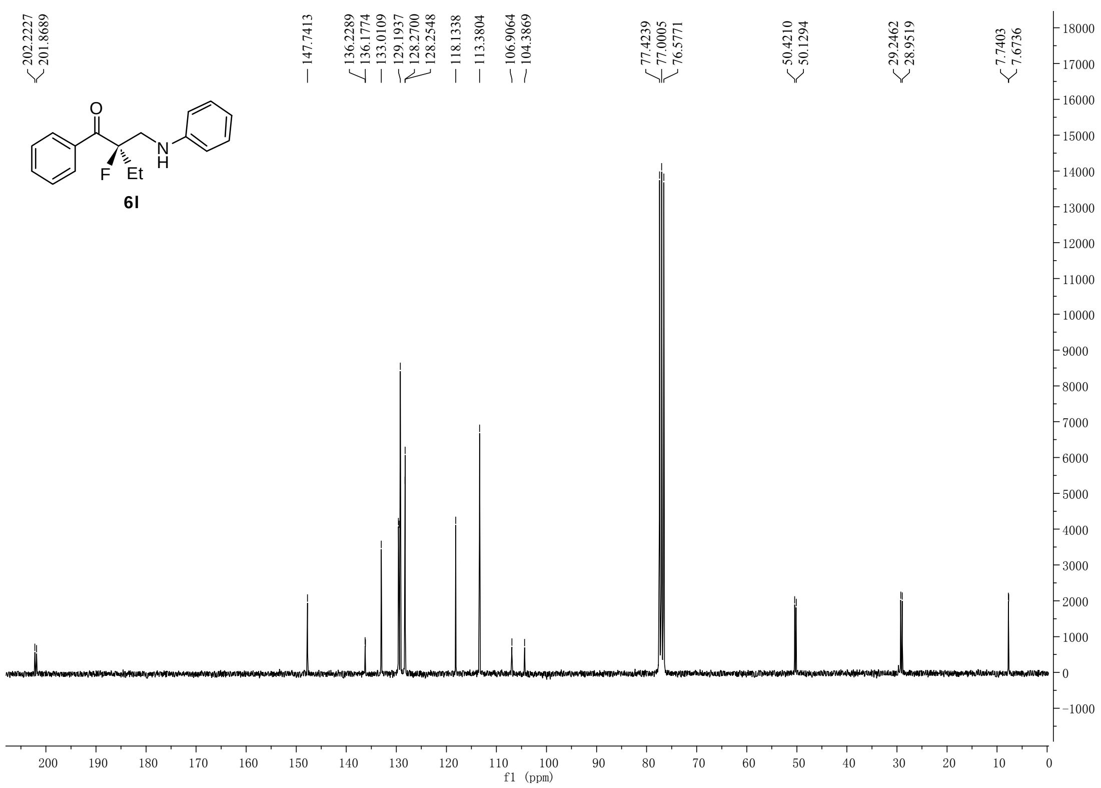

Supplementary Figure 49. ^{1}H and ^{13}C NMR spectra for compound **6l**

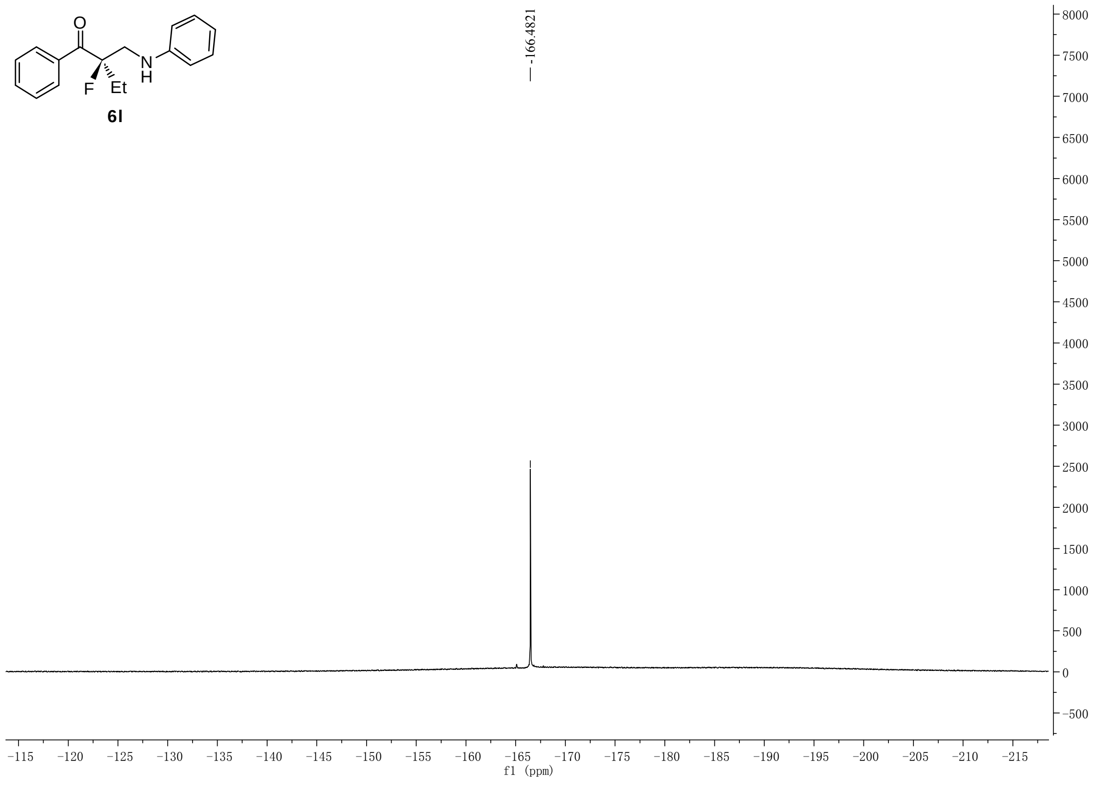

Supplementary Figure 50. ^{19}F NMR spectra for compound **6l**

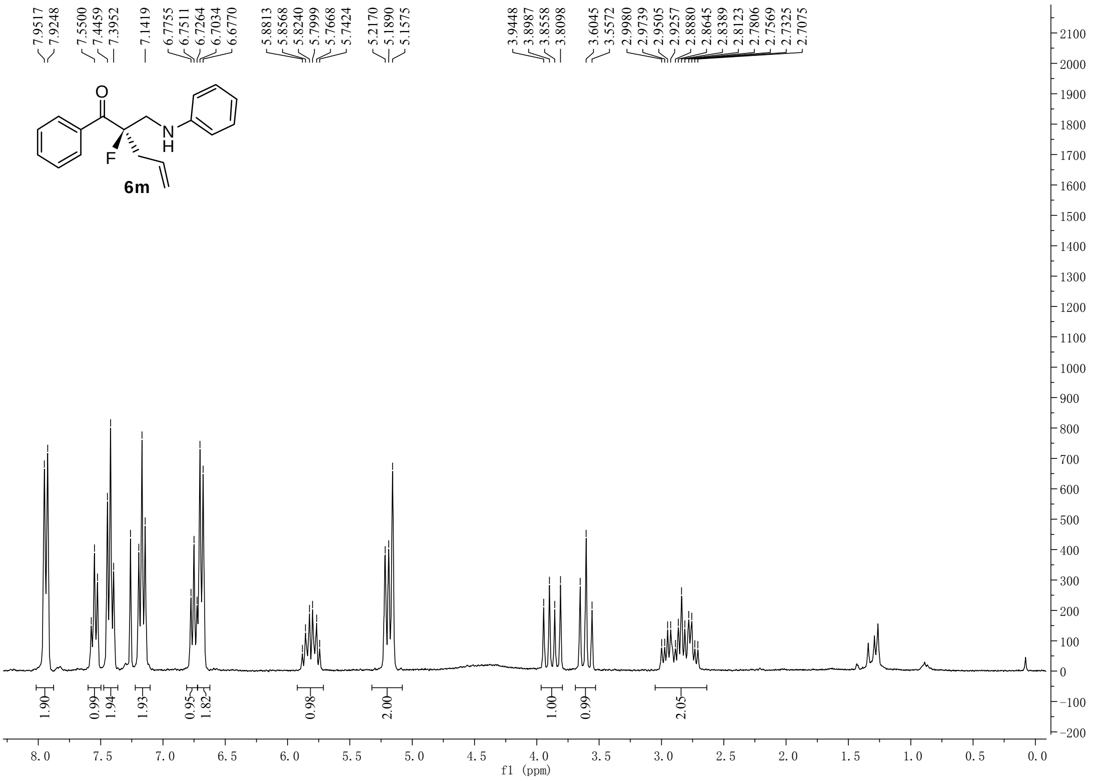

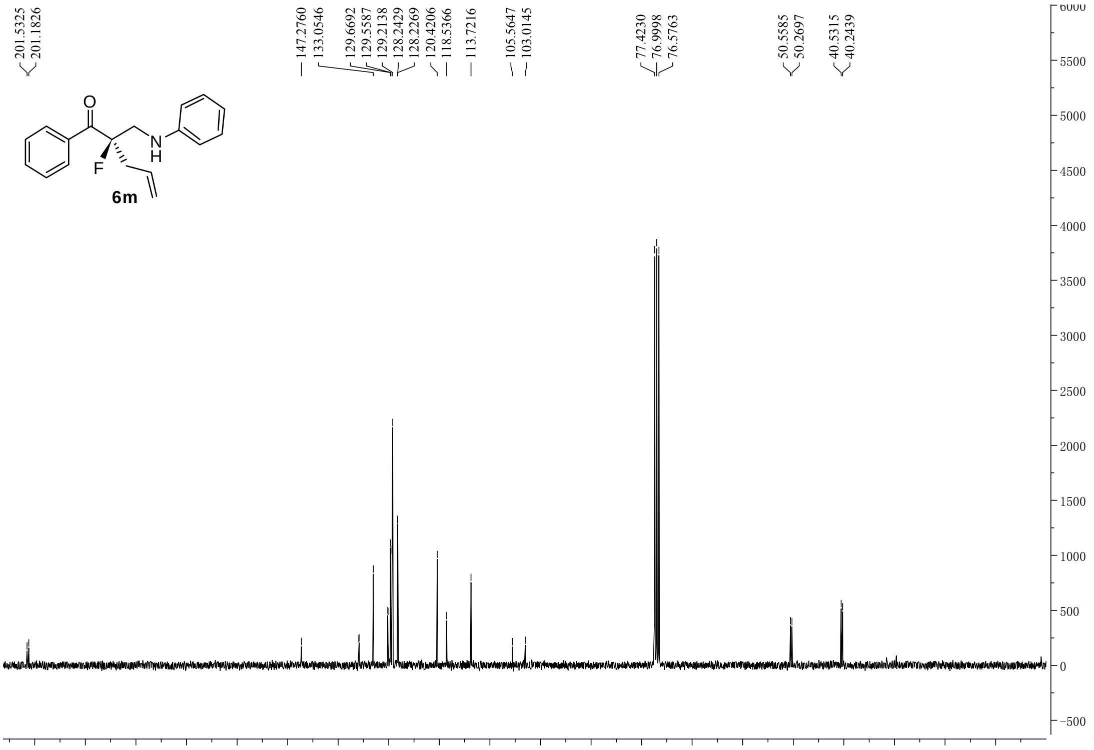

Supplementary Figure 51. ^{1}H and ^{13}C NMR spectra for compound **6m**

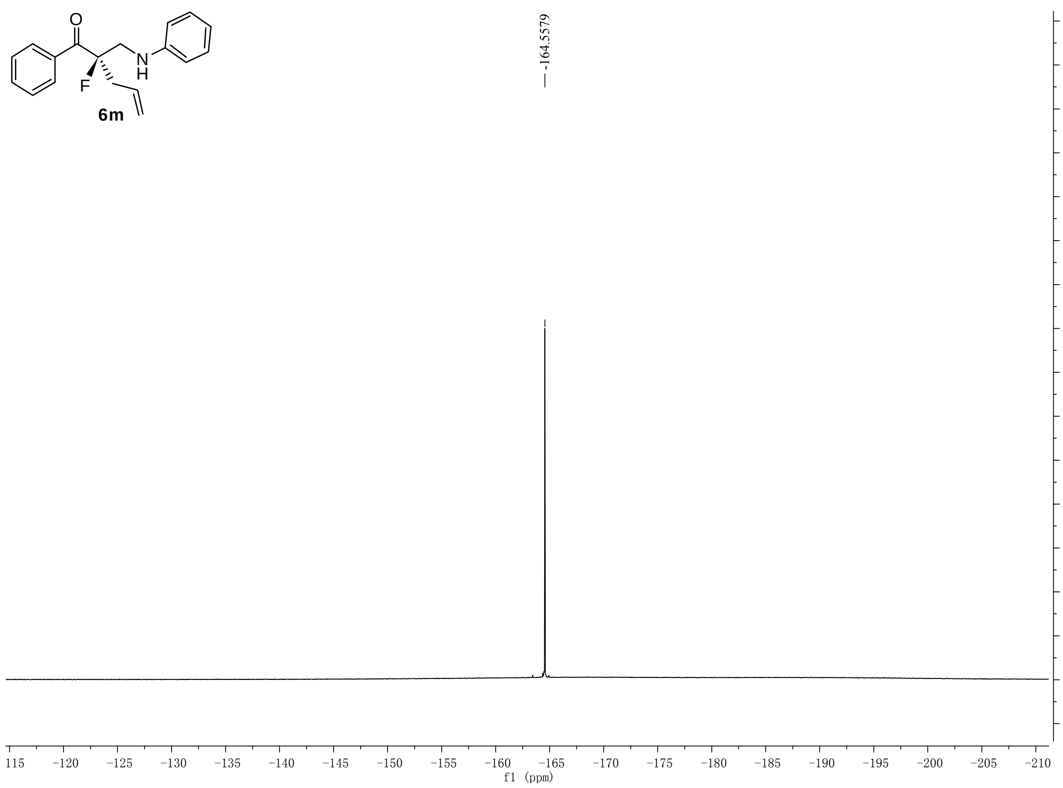

Supplementary Figure 52. ^{19}F NMR spectra for compound **6m**

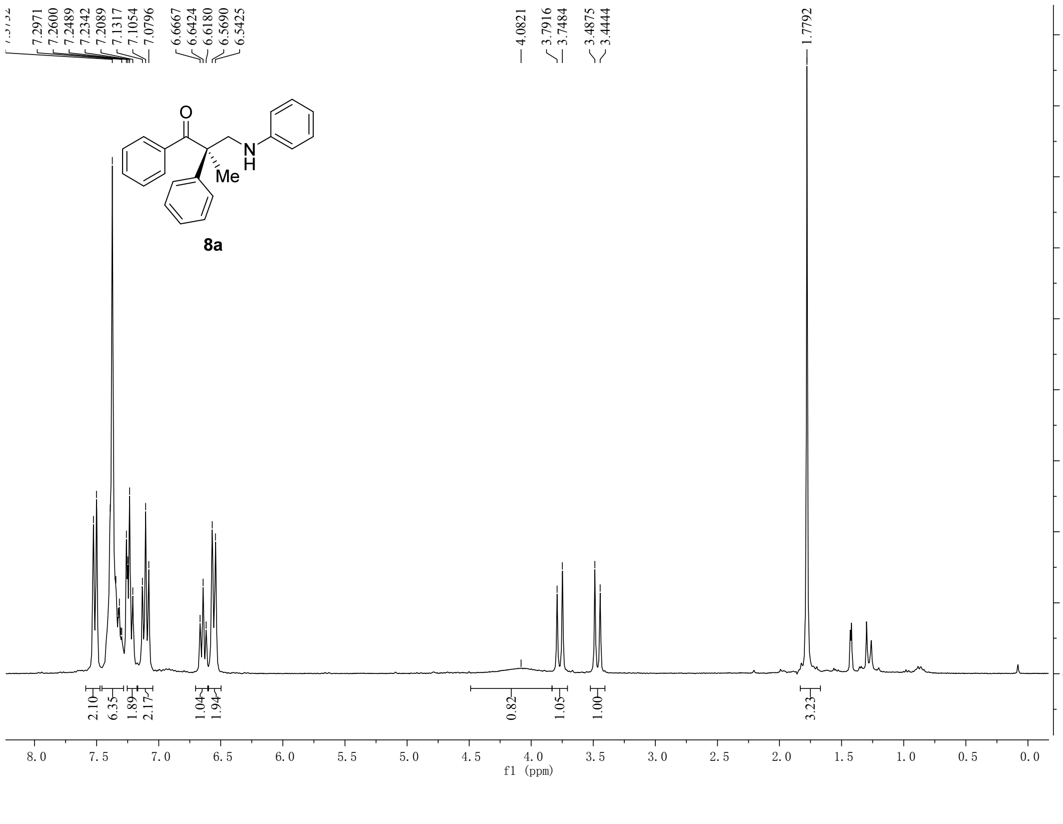

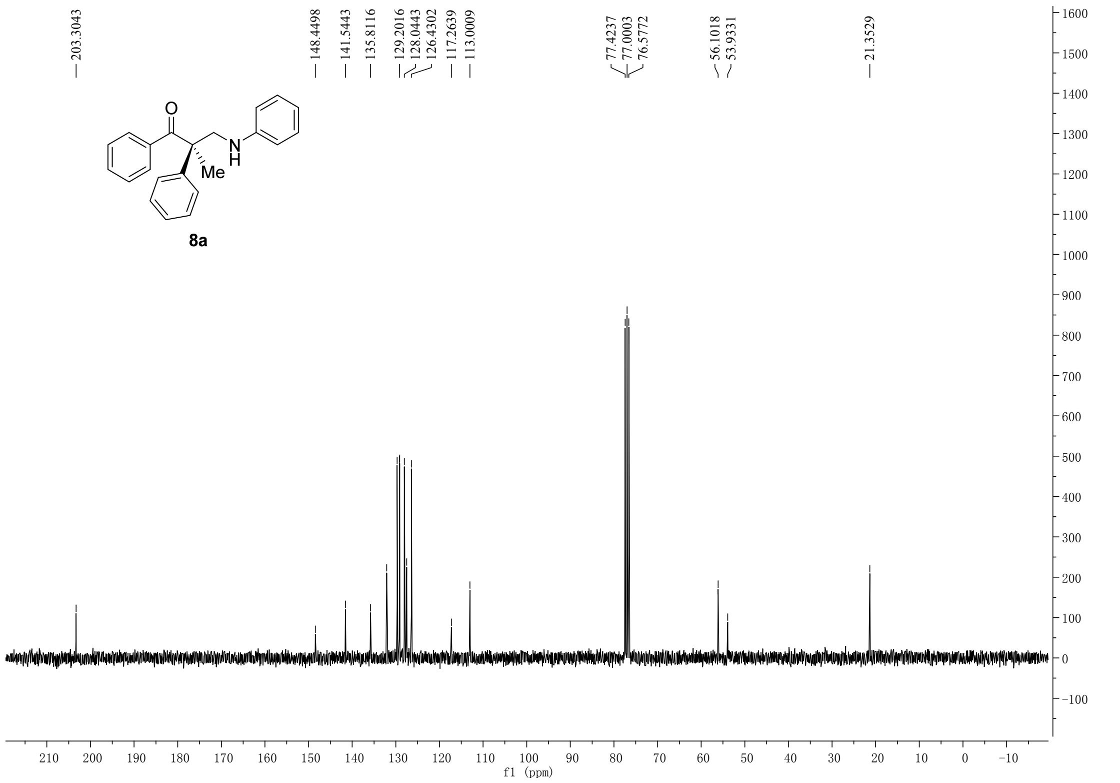

Supplementary Figure 53. ^{1}H and ^{13}C NMR spectra for compound **8a**

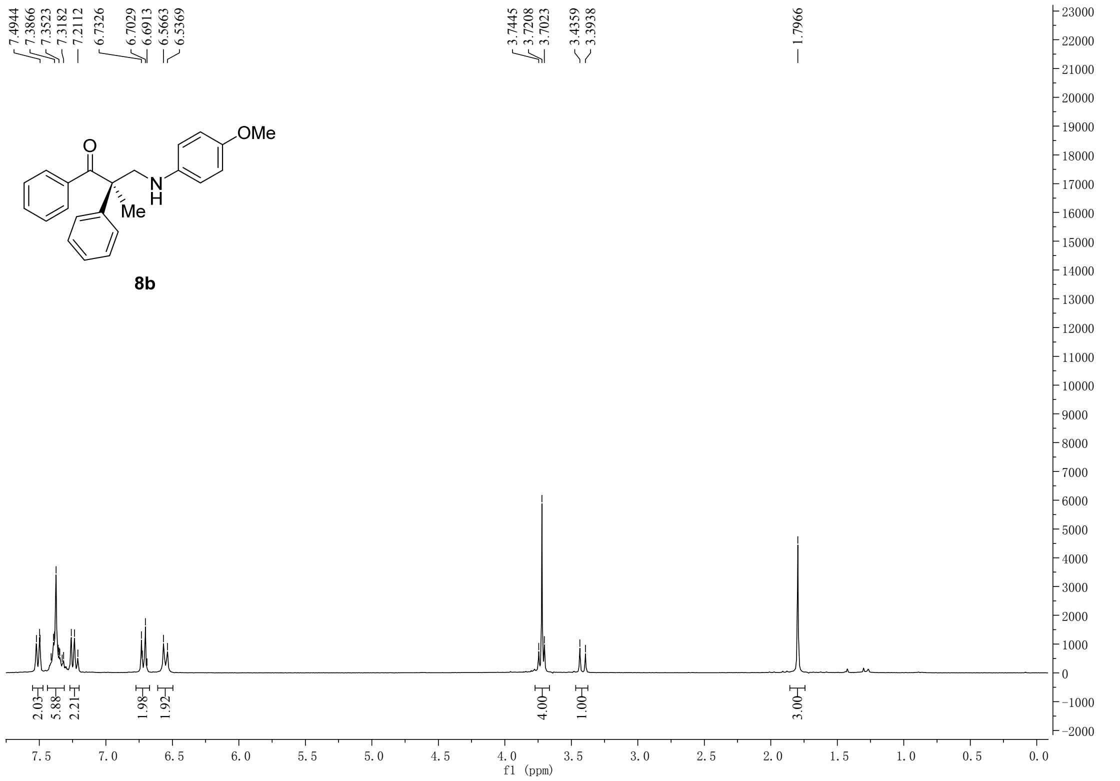

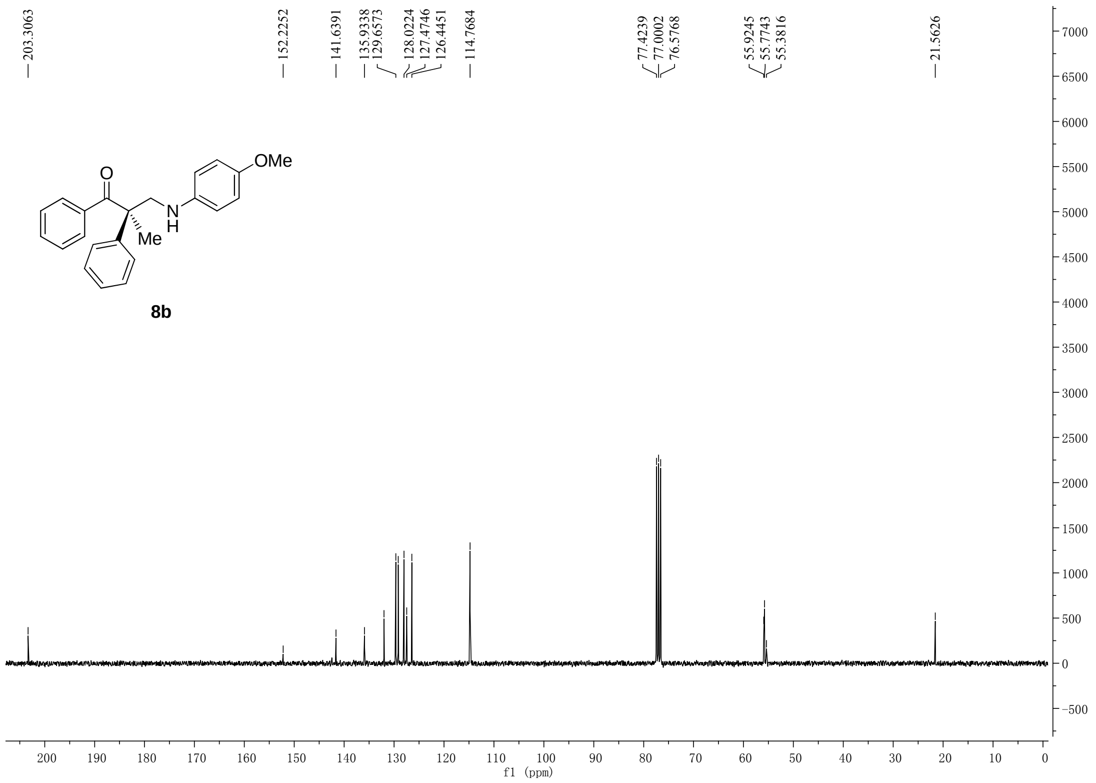

Supplementary Figure 54. ^{1}H and ^{13}C NMR spectra for compound **8b**

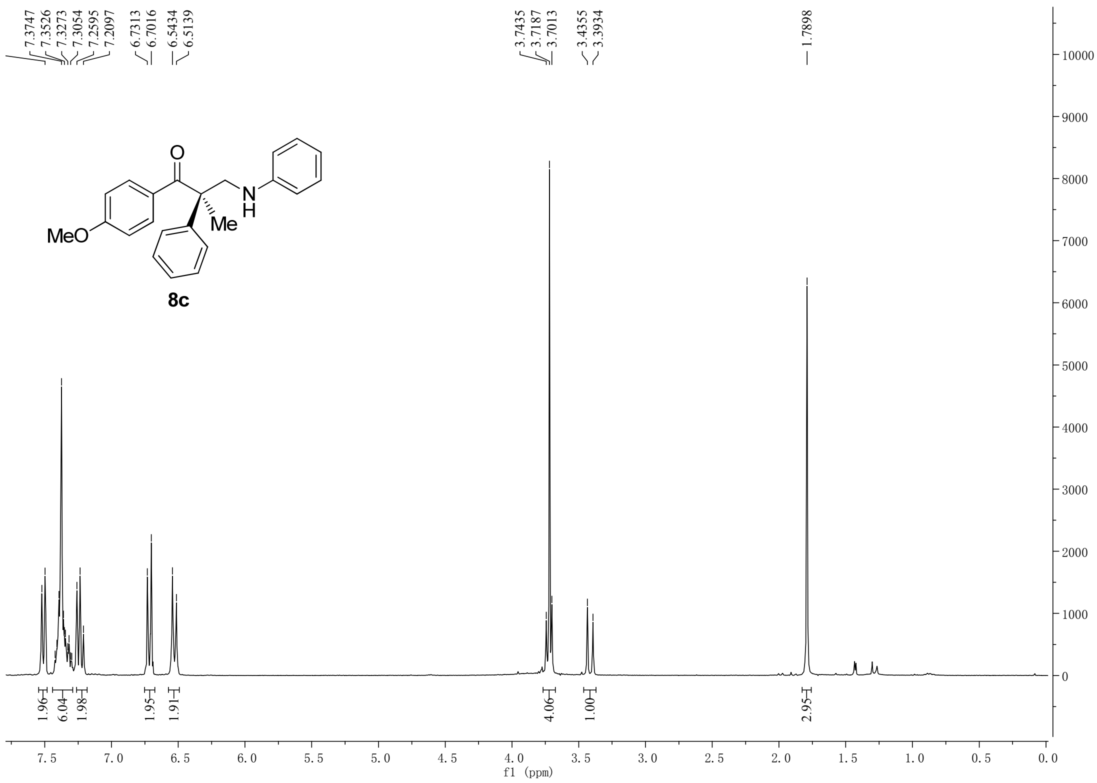

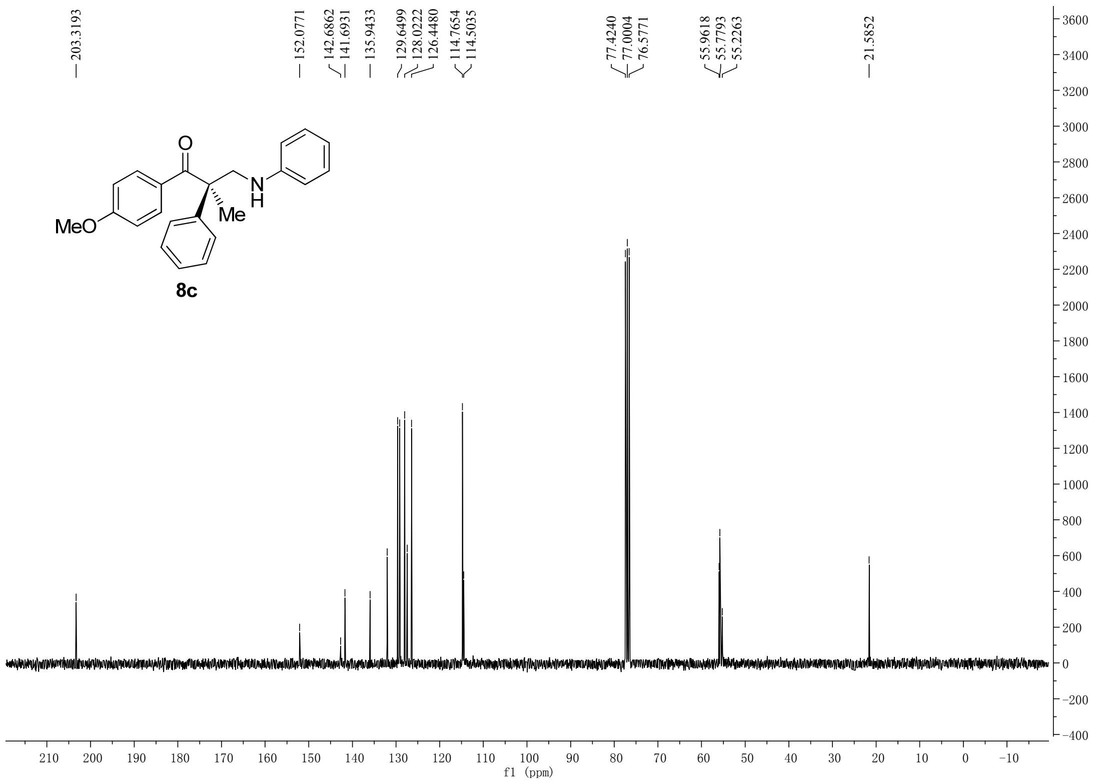

Supplementary Figure 55. ^{1}H and ^{13}C NMR spectra for compound **8c**

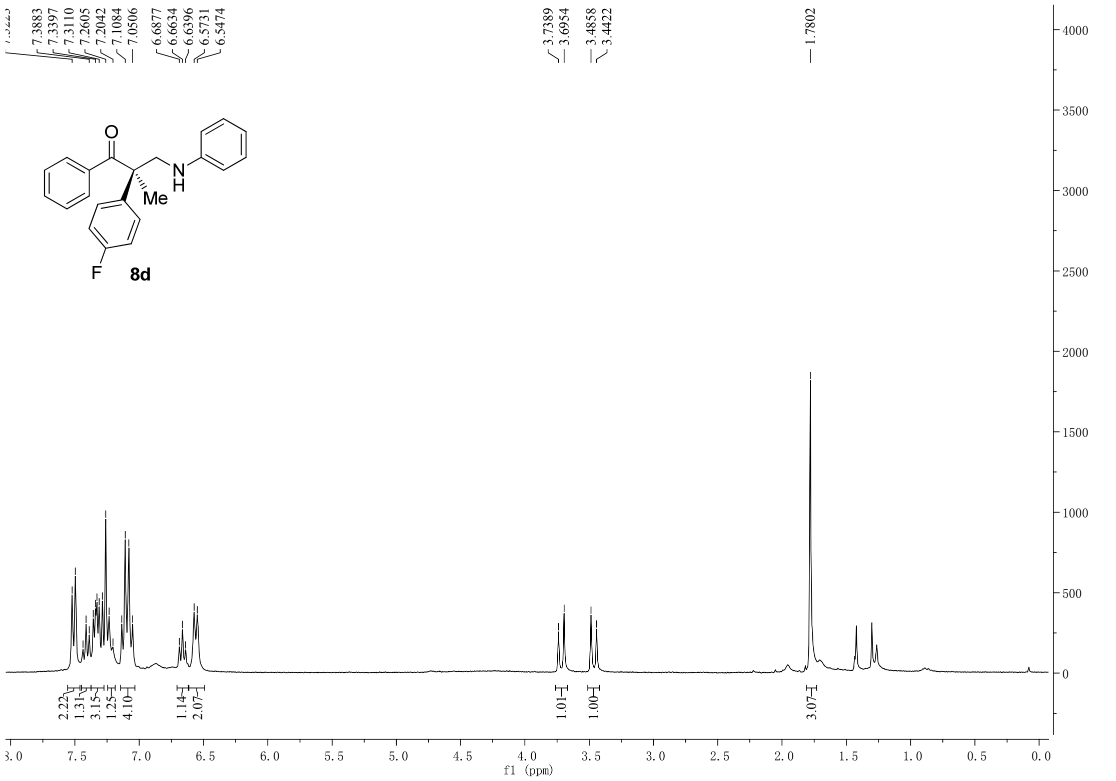

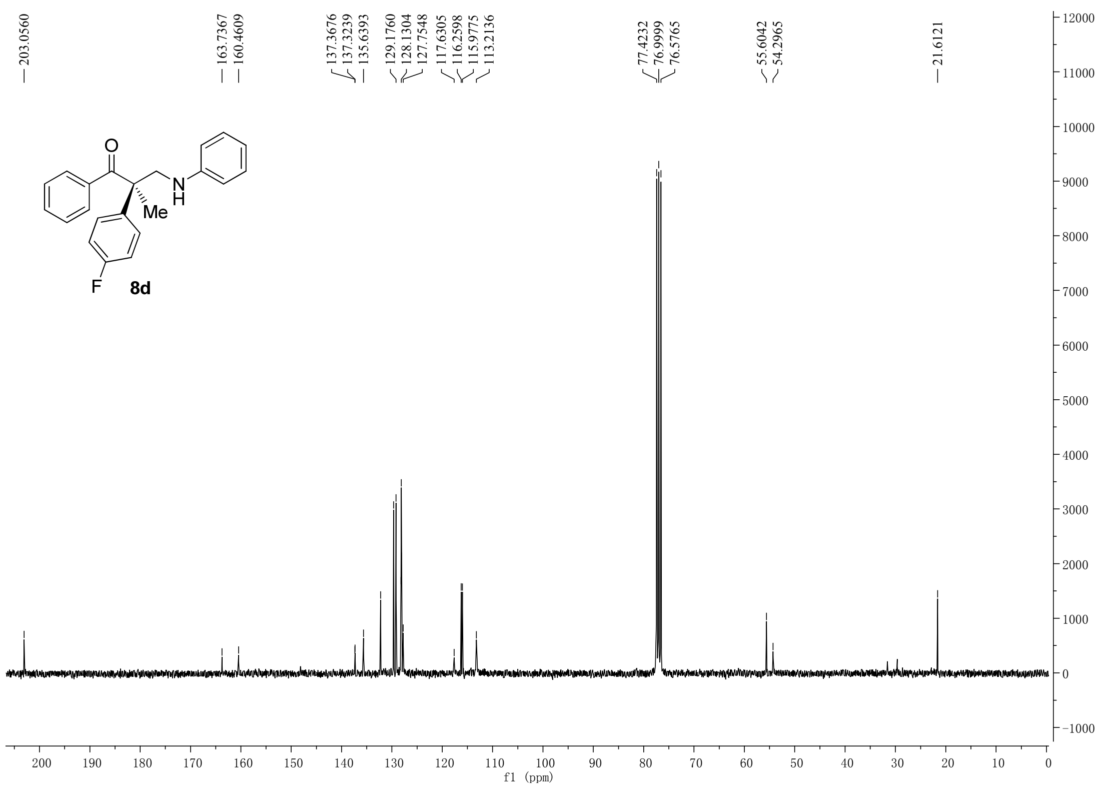

Supplementary Figure 56. ^{1}H and ^{13}C NMR spectra for compound **8d**

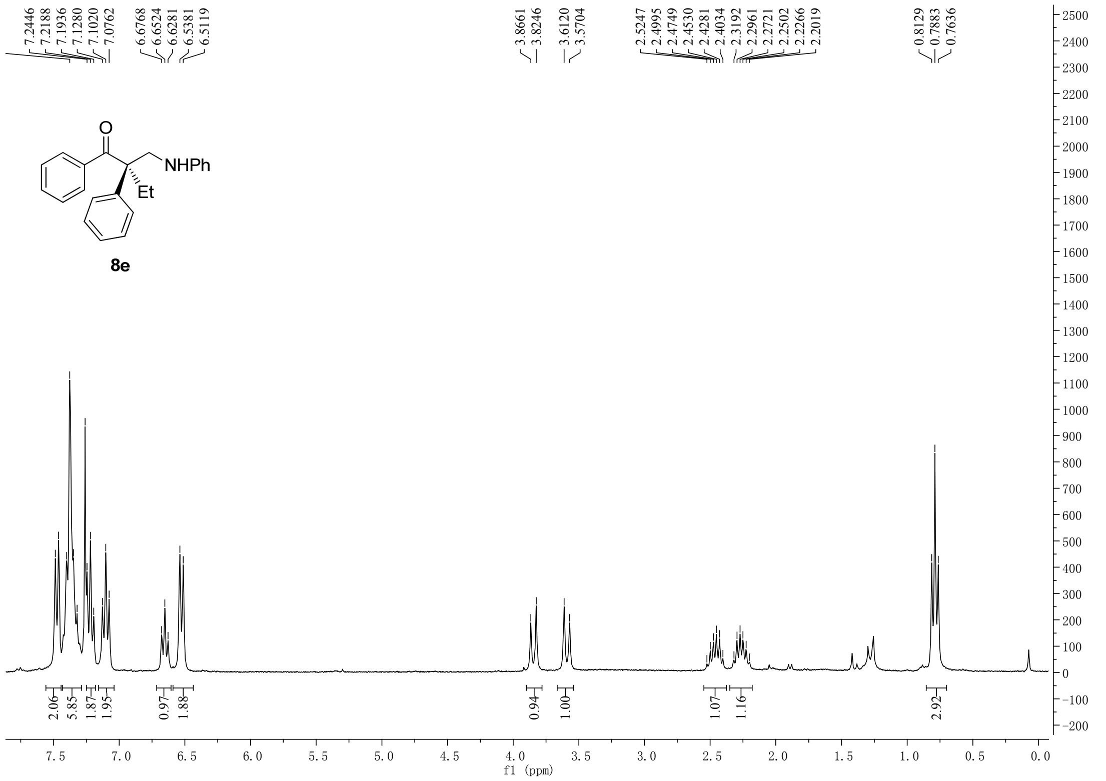

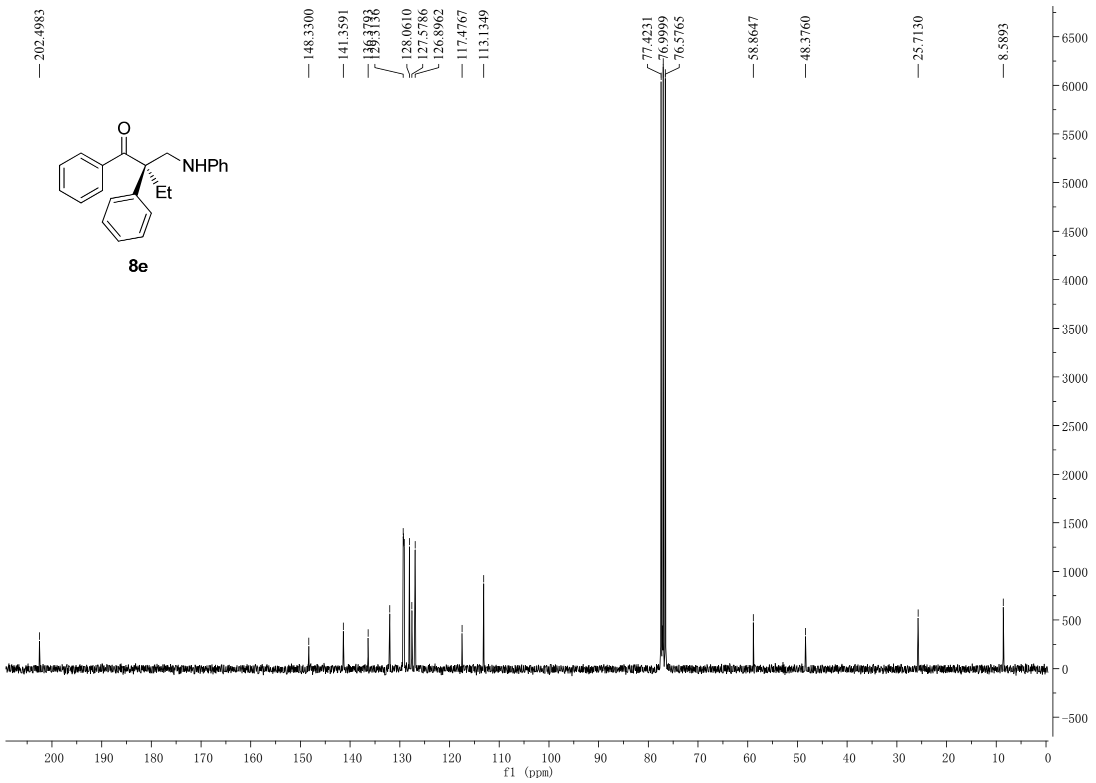

Supplementary Figure 57. ^{1}H and ^{13}C NMR spectra for compound **8e**

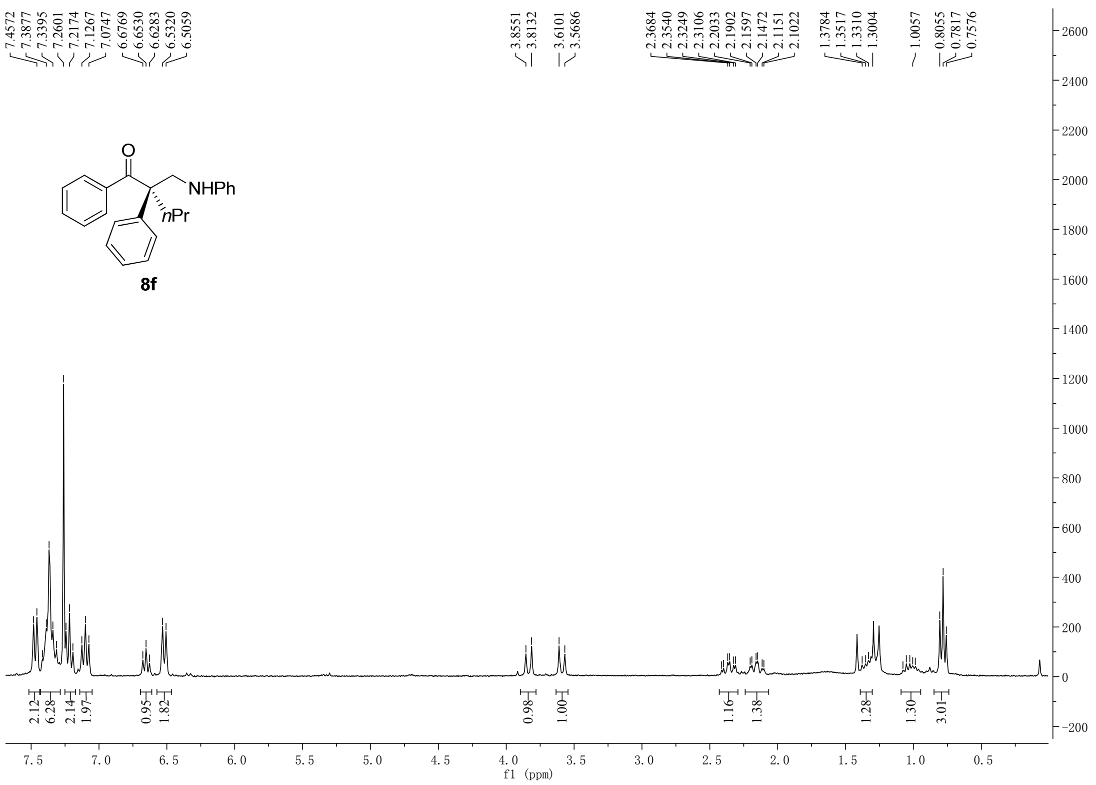

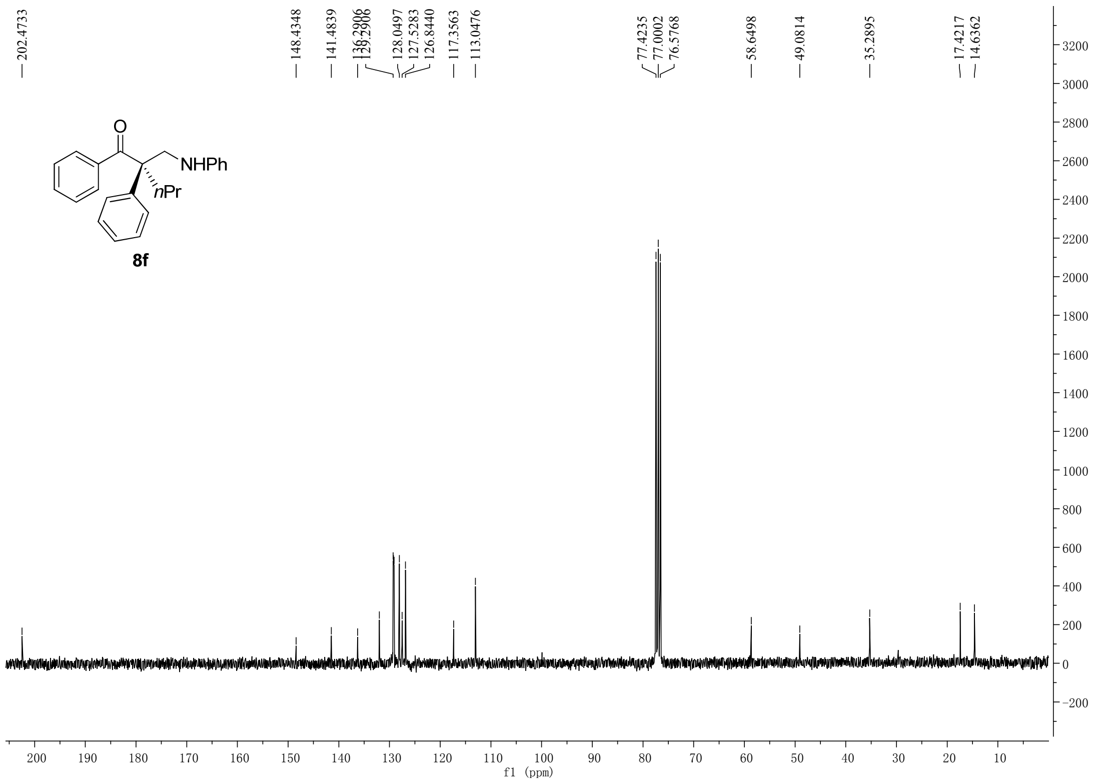

Supplementary Figure 58. ^{1}H and ^{13}C NMR spectra for compound **8f**

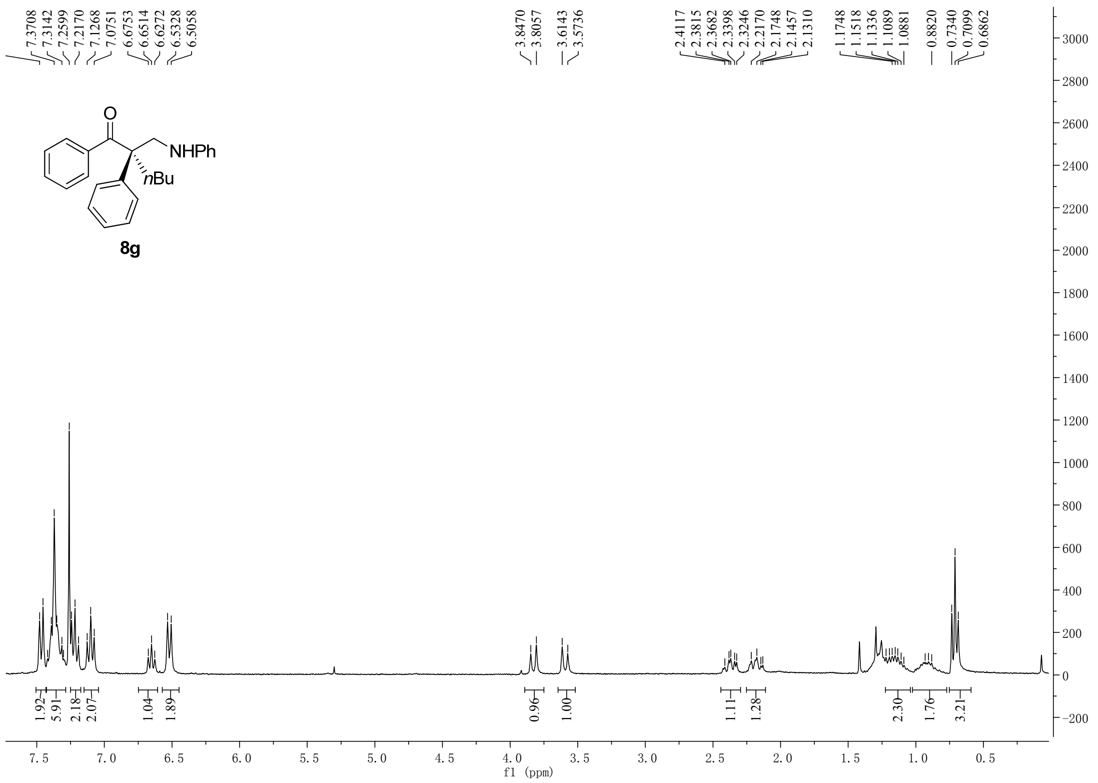

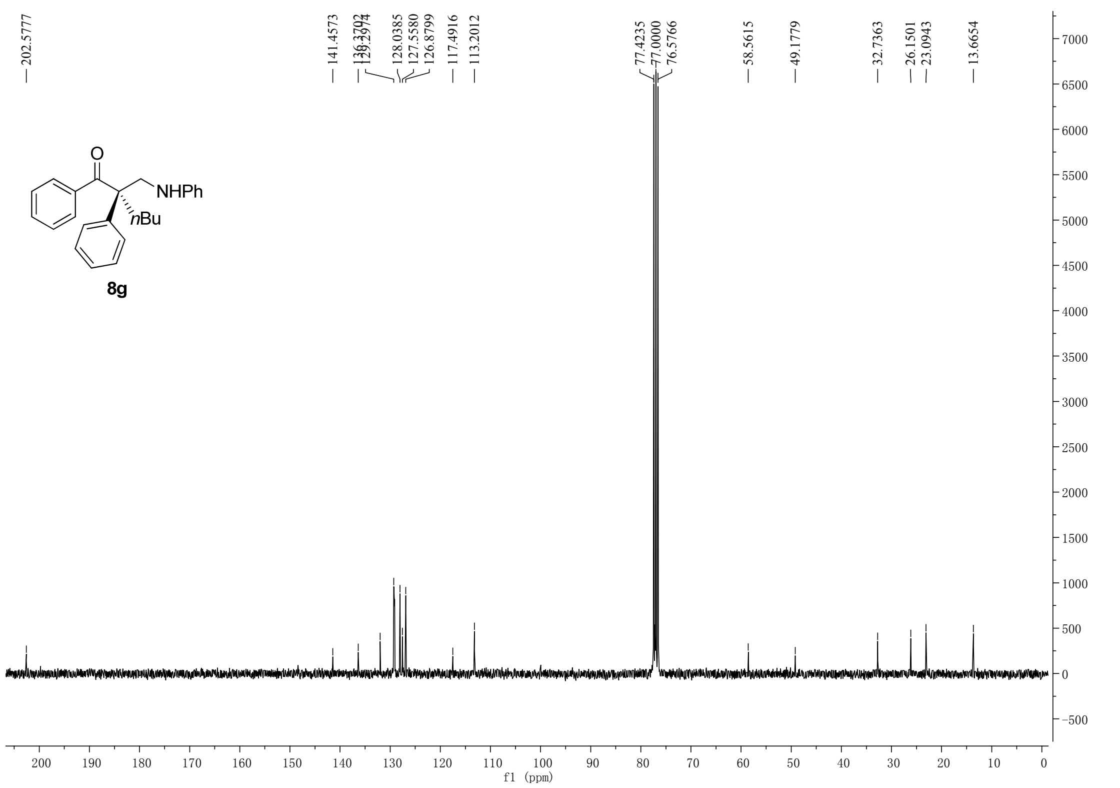

Supplementary Figure 59. ^{1}H and ^{13}C NMR spectra for compound **8g**

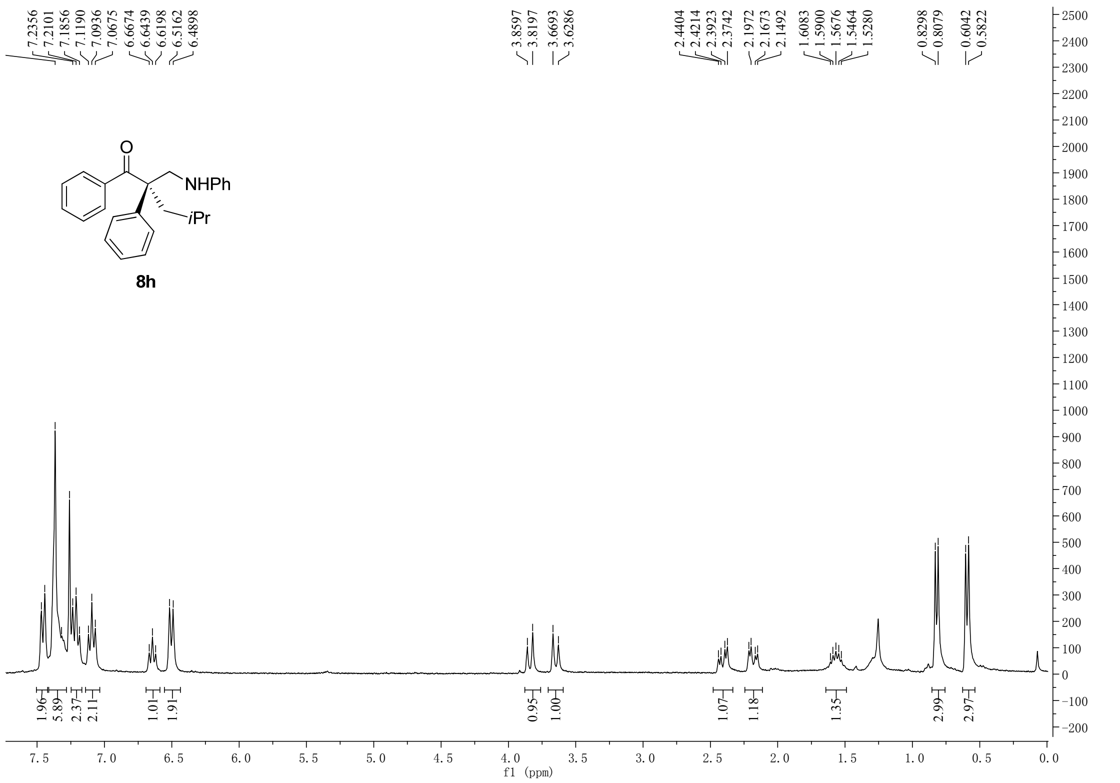

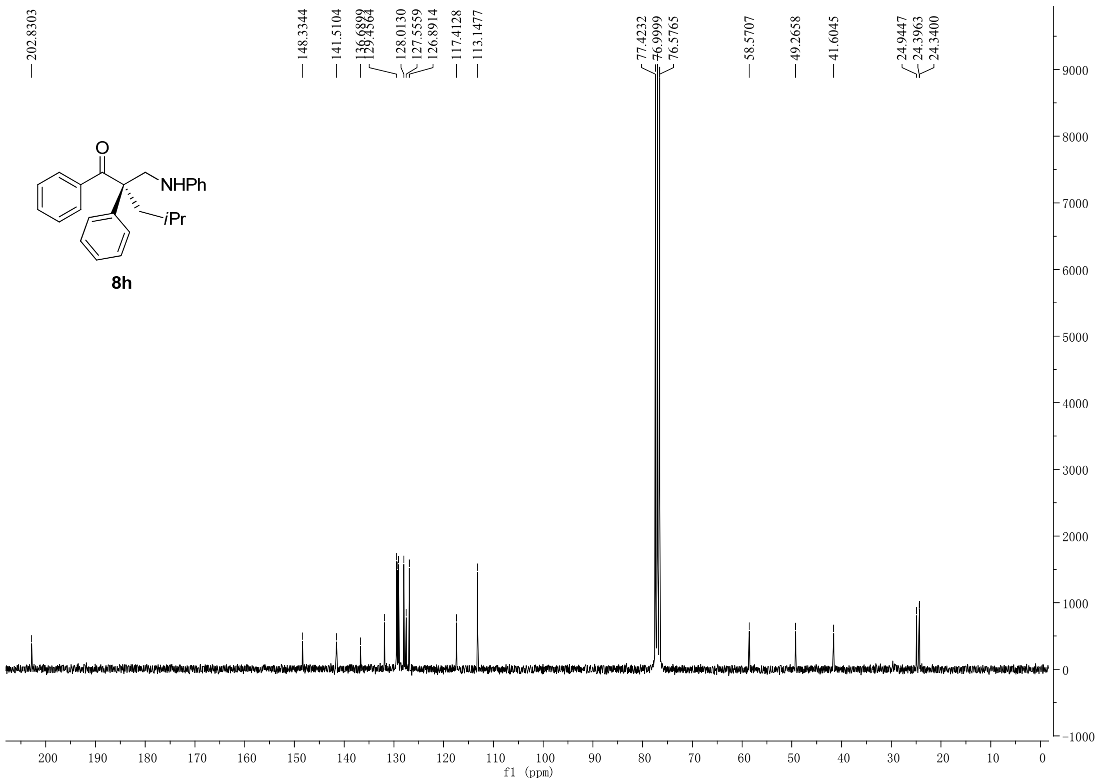

Supplementary Figure 60. ^{1}H and ^{13}C NMR spectra for compound **8h**

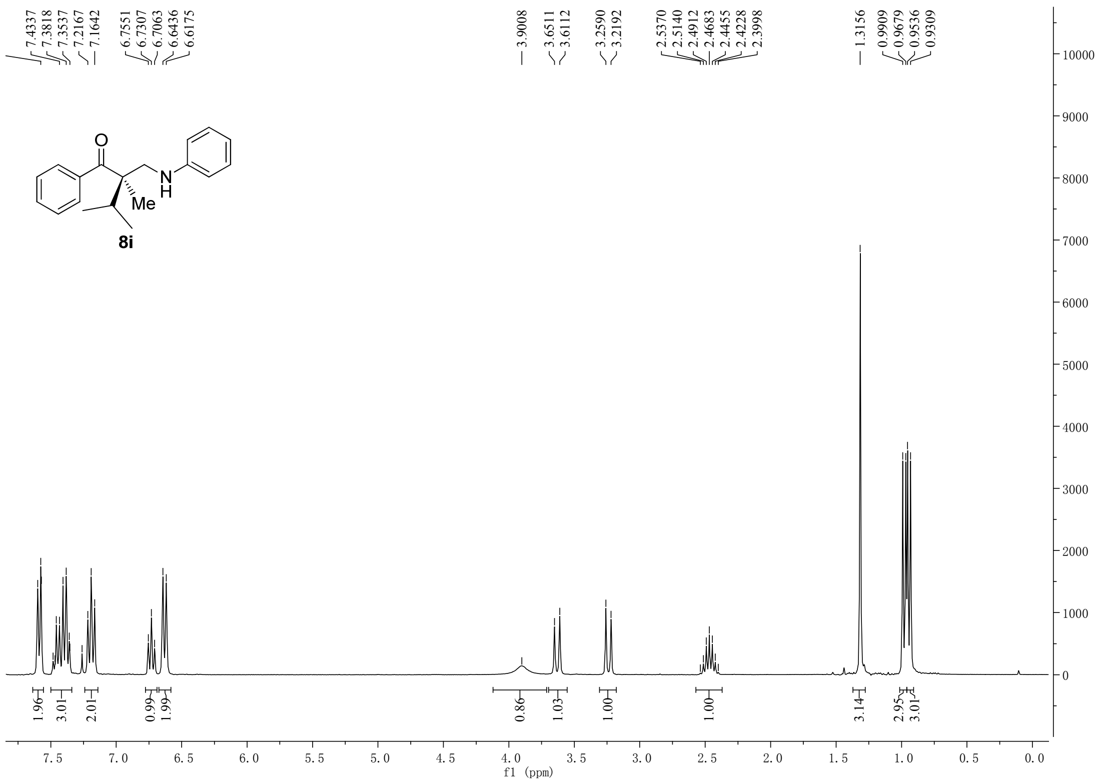

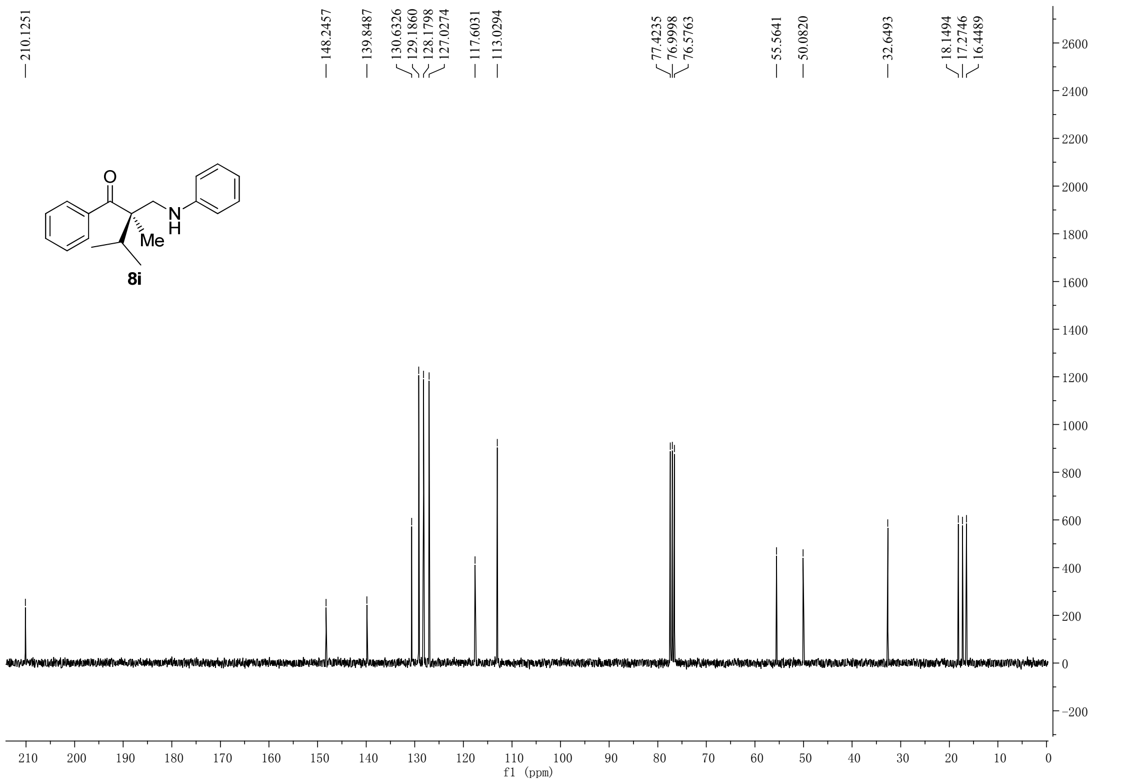

Supplementary Figure 61. ^{1}H and ^{13}C NMR spectra for compound **8i**

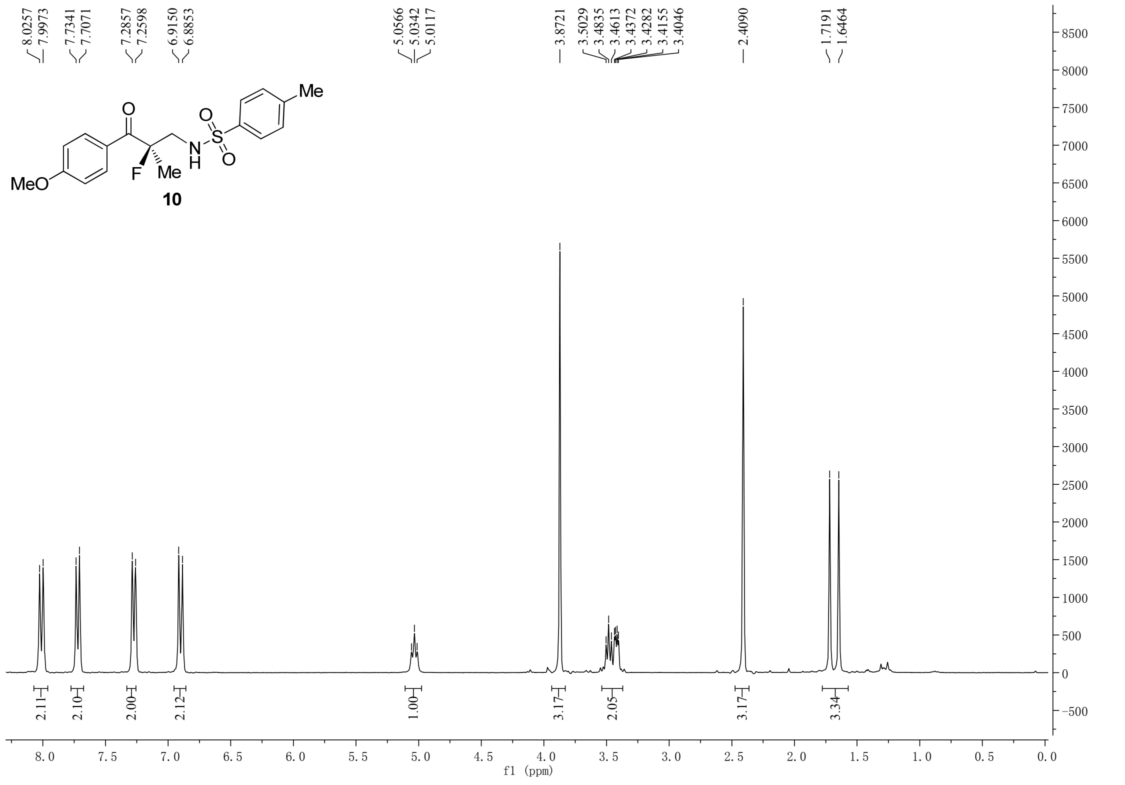

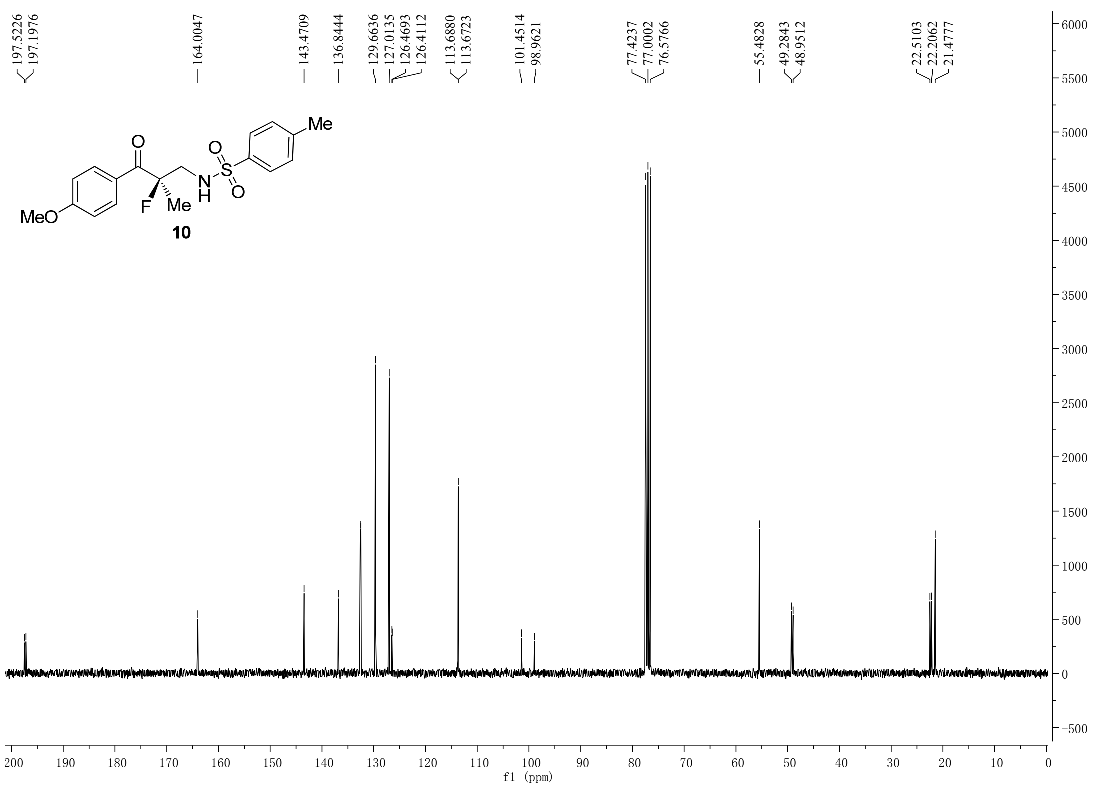

Supplementary Figure 62. ^{1}H and ^{13}C spectra for compound **10**

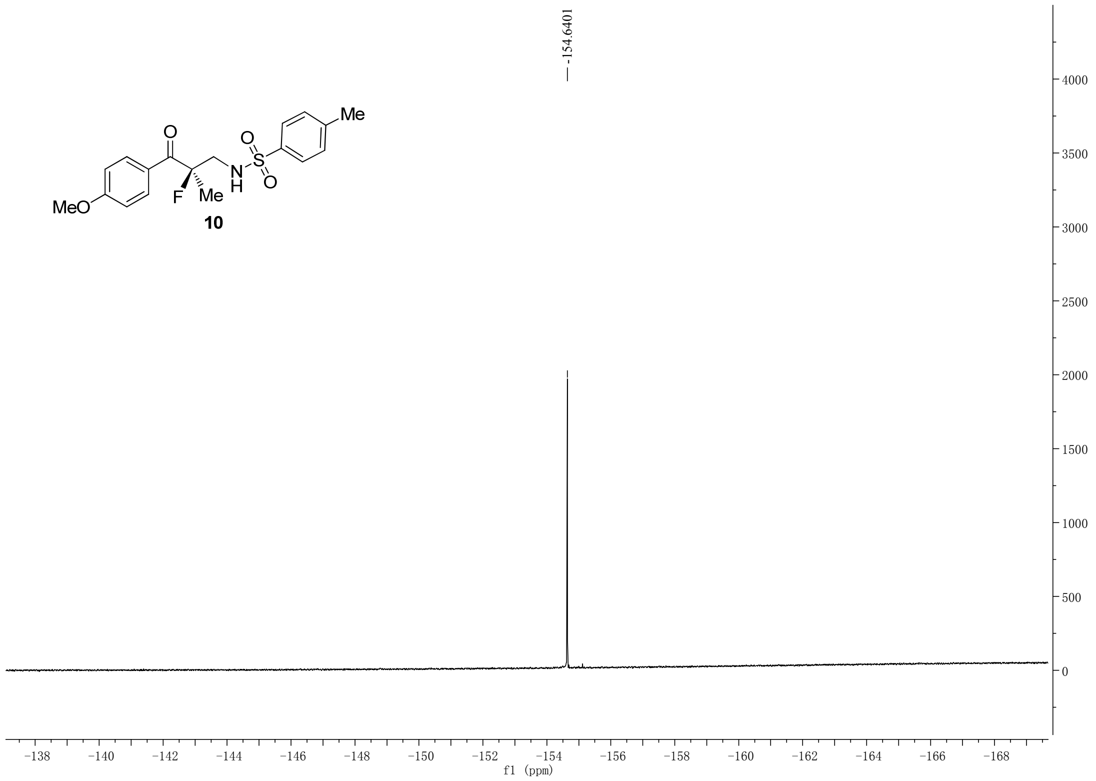

Supplementary Figure 63. ^{19}F NMR spectra for compound **10**

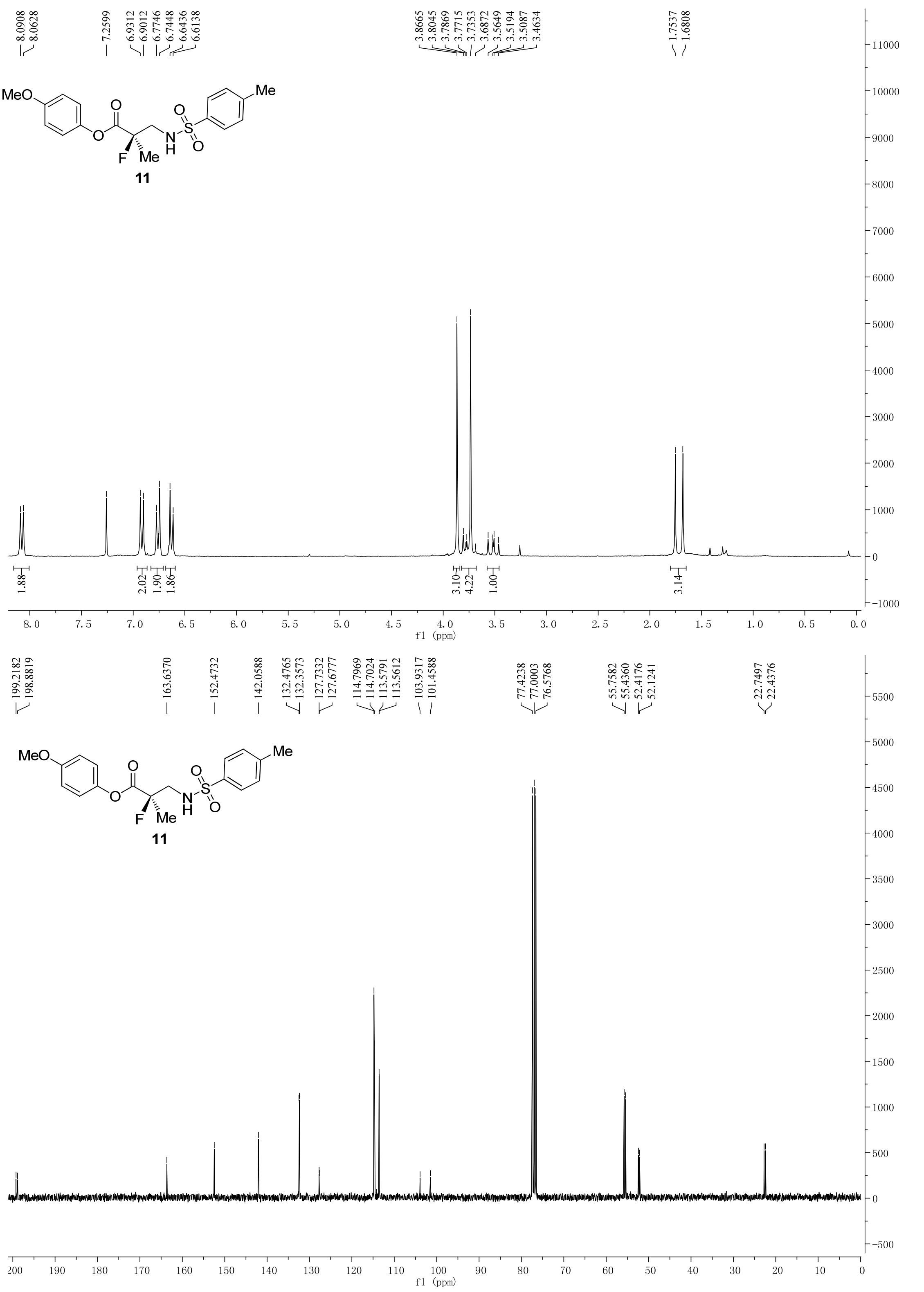

Supplementary Figure 64. ^{1}H and ^{13}C NMR spectra for compound **11**

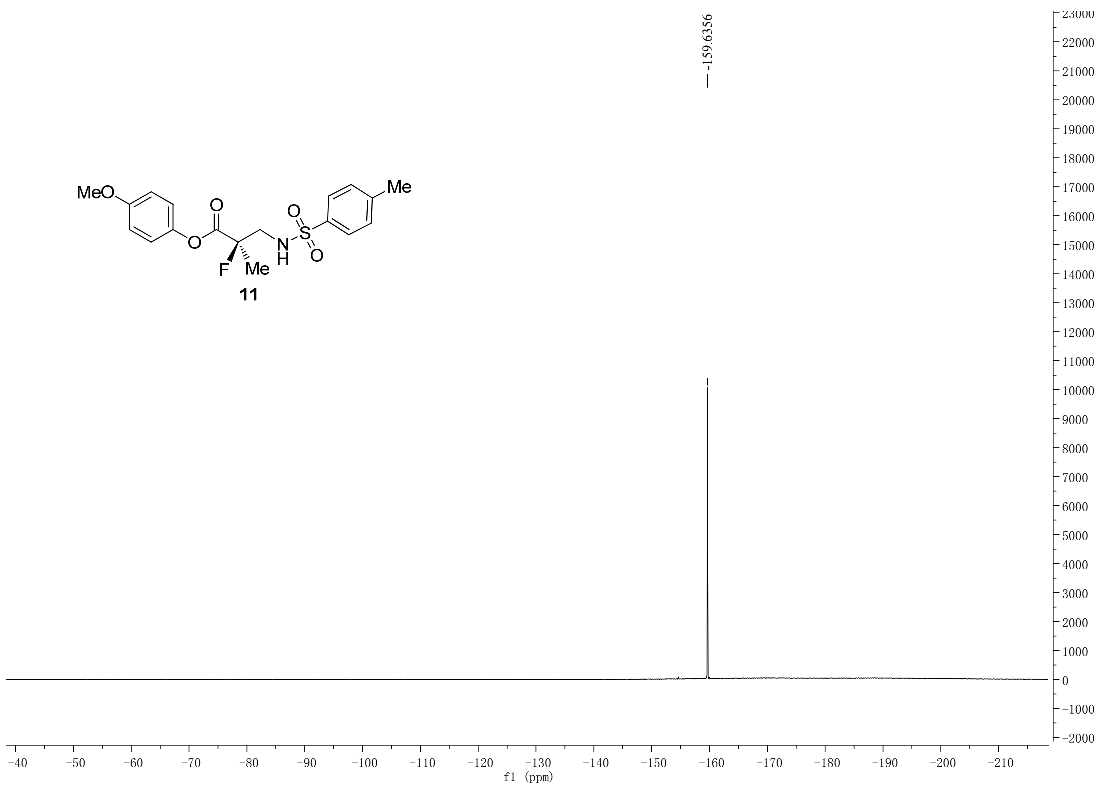

Supplementary Figure 65. ^{19}F NMR spectra for compound **11**

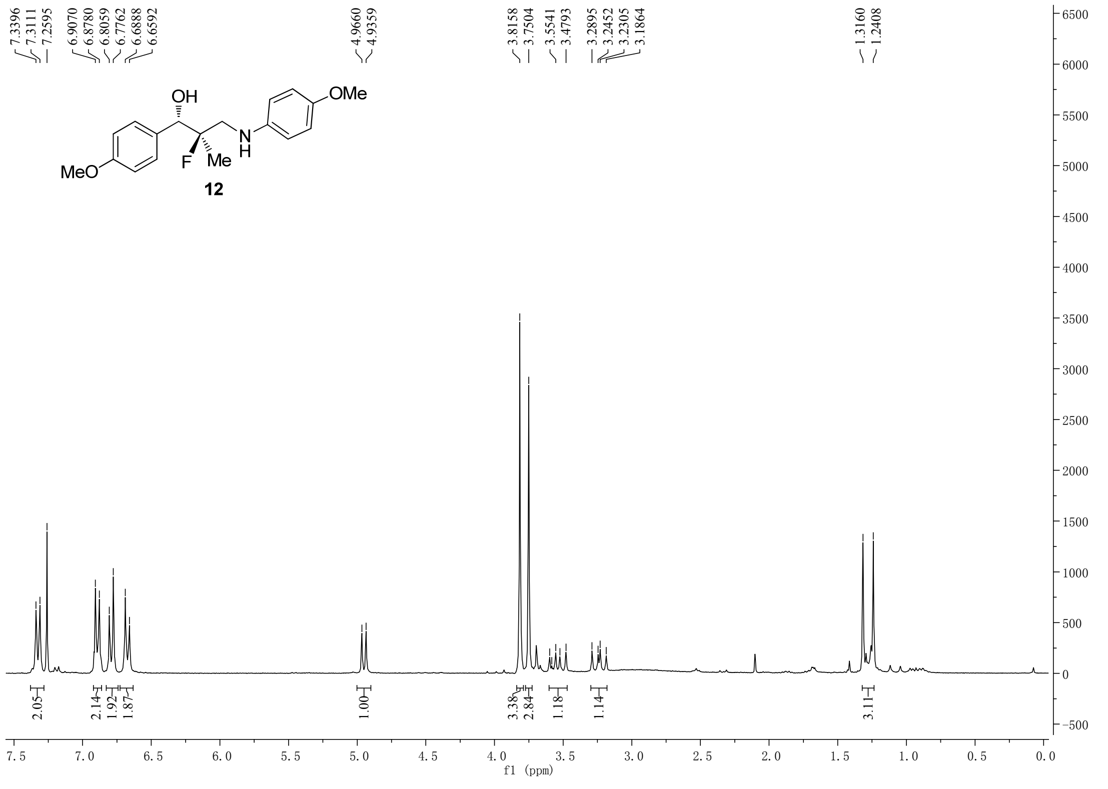

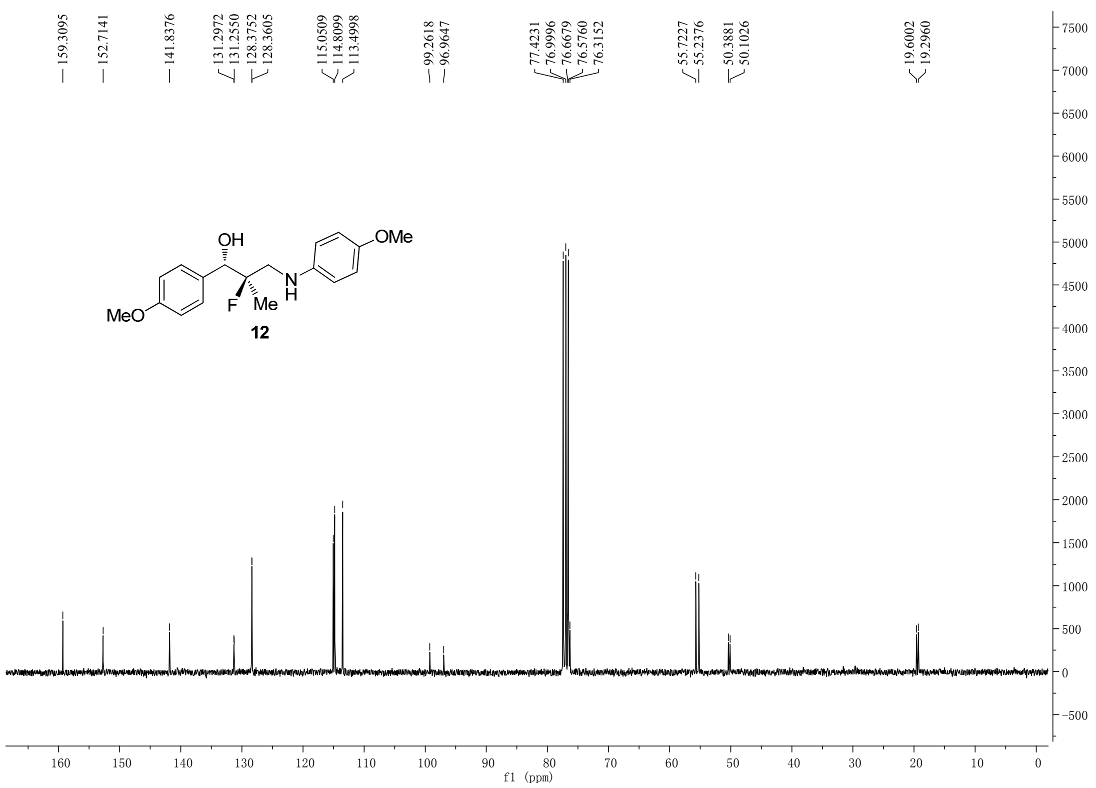

Supplementary Figure 66. ^{1}H and ^{13}C NMR spectra for compound **12**

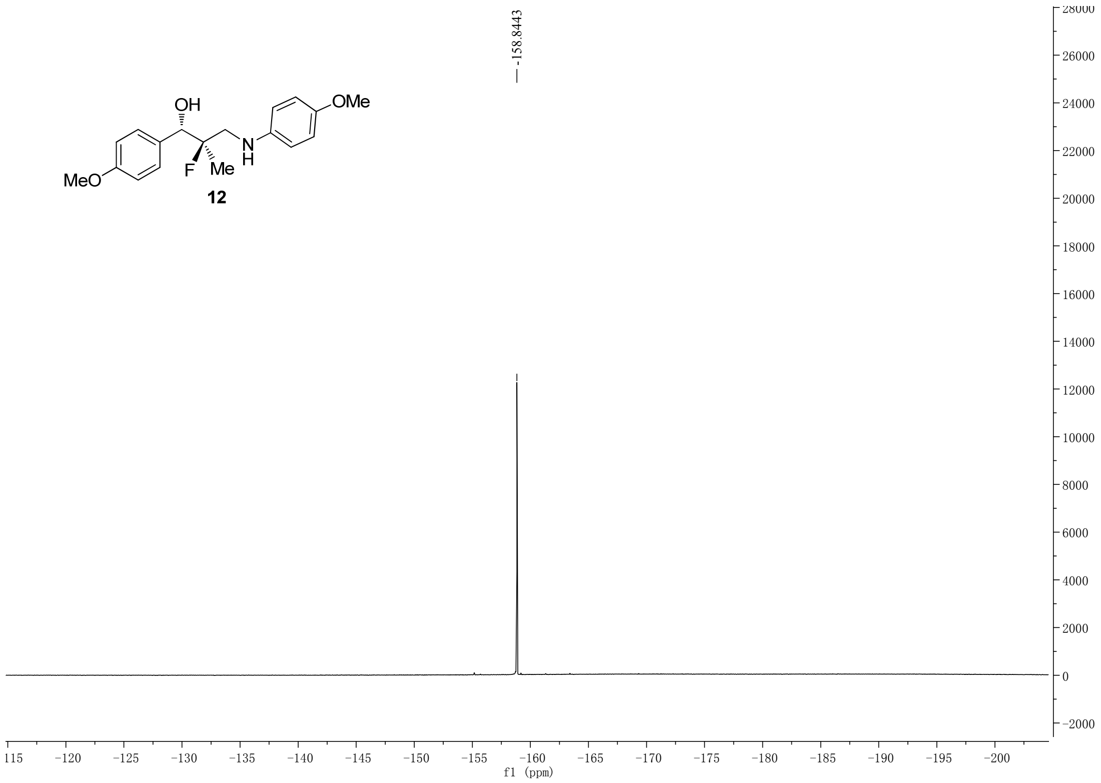

Supplementary Figure 67. ^{19}F NMR spectra for compound **12**

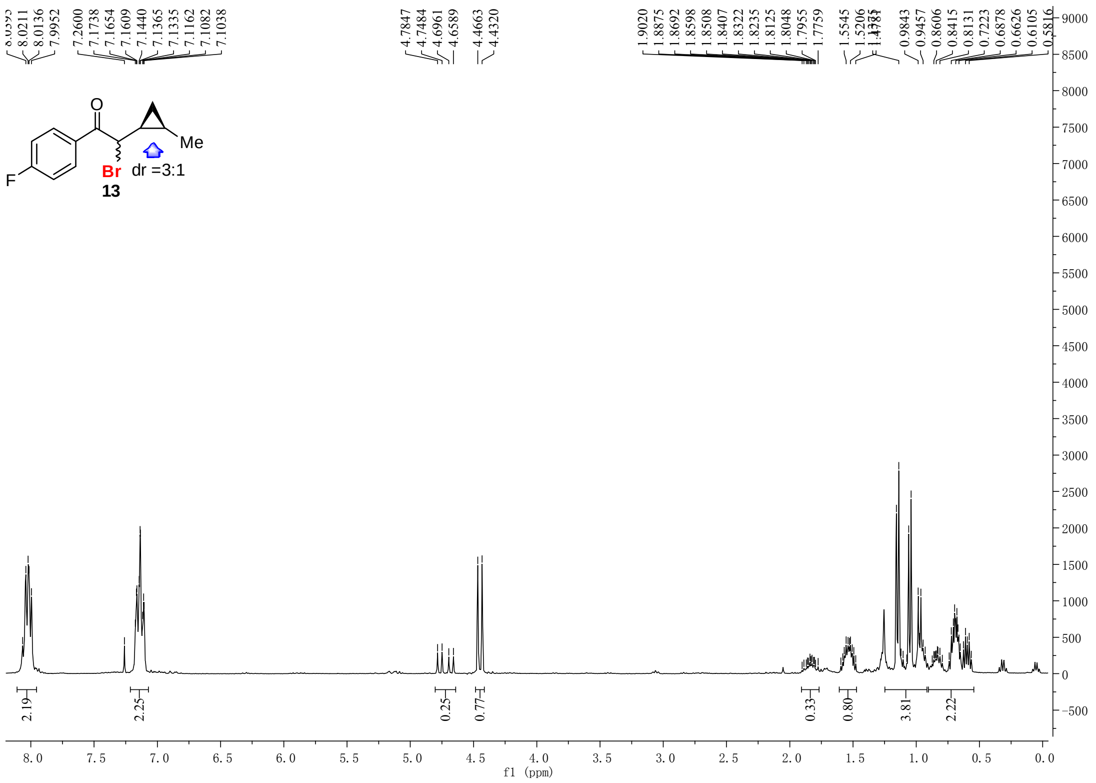

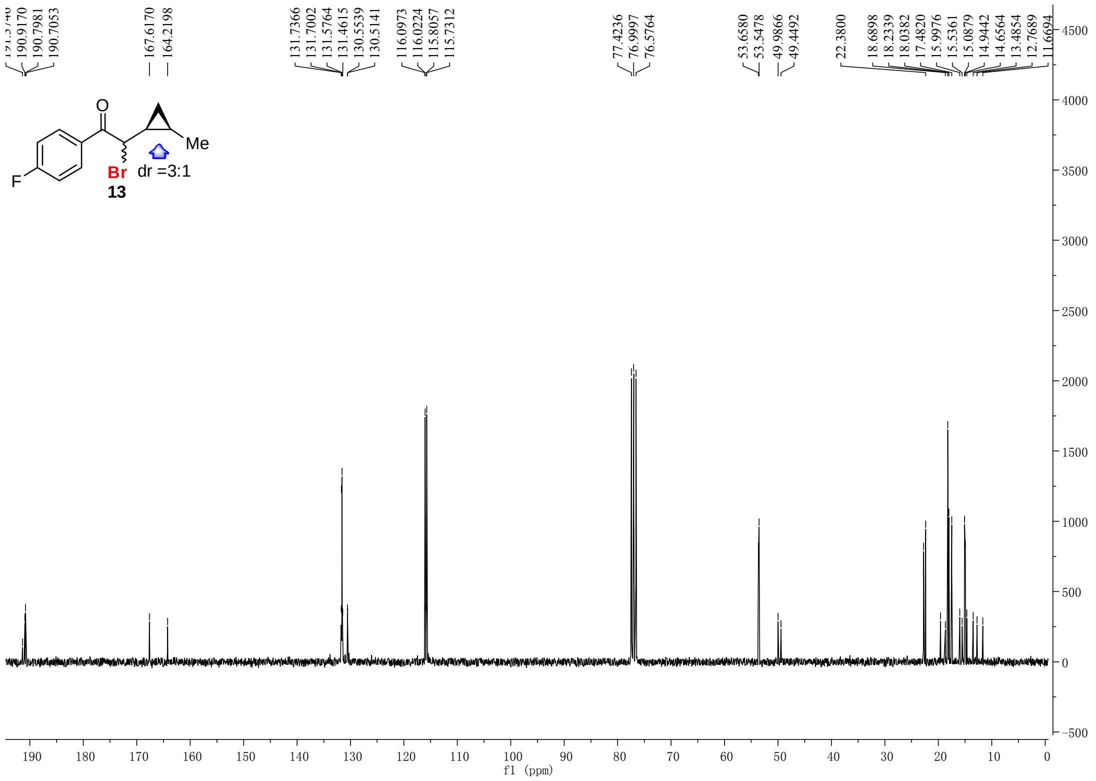

Supplementary Figure 68. ^{1}H and ^{13}C NMR spectra for compound **13**

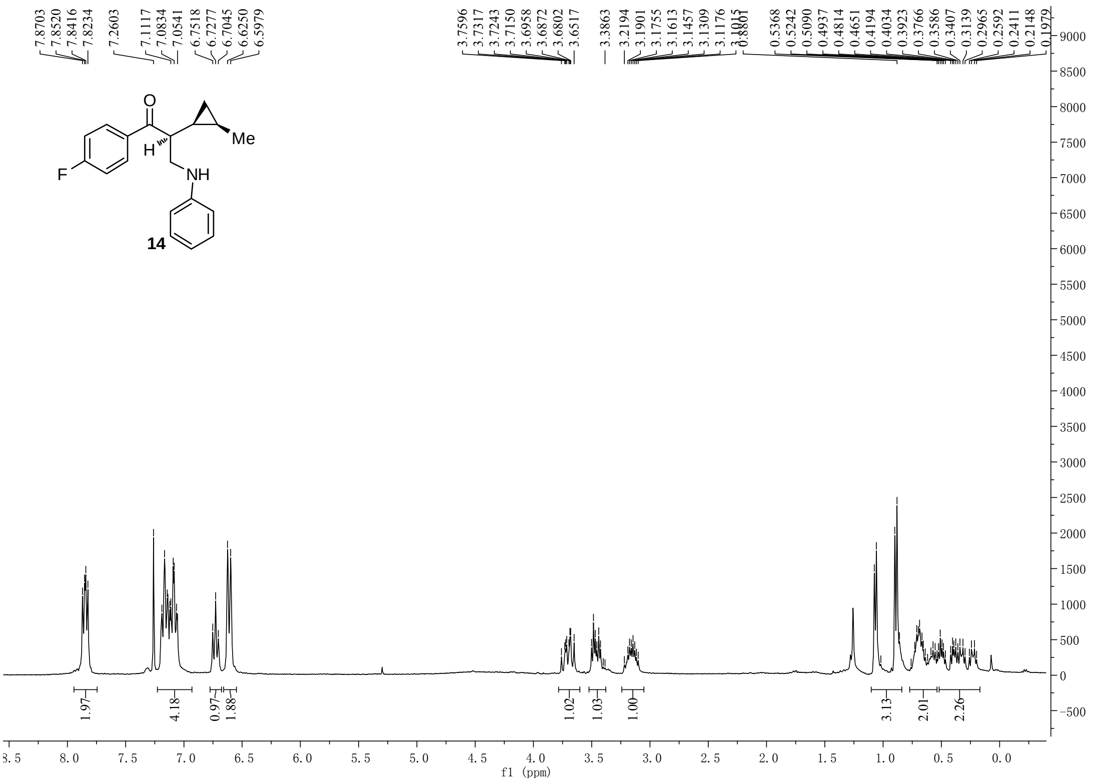

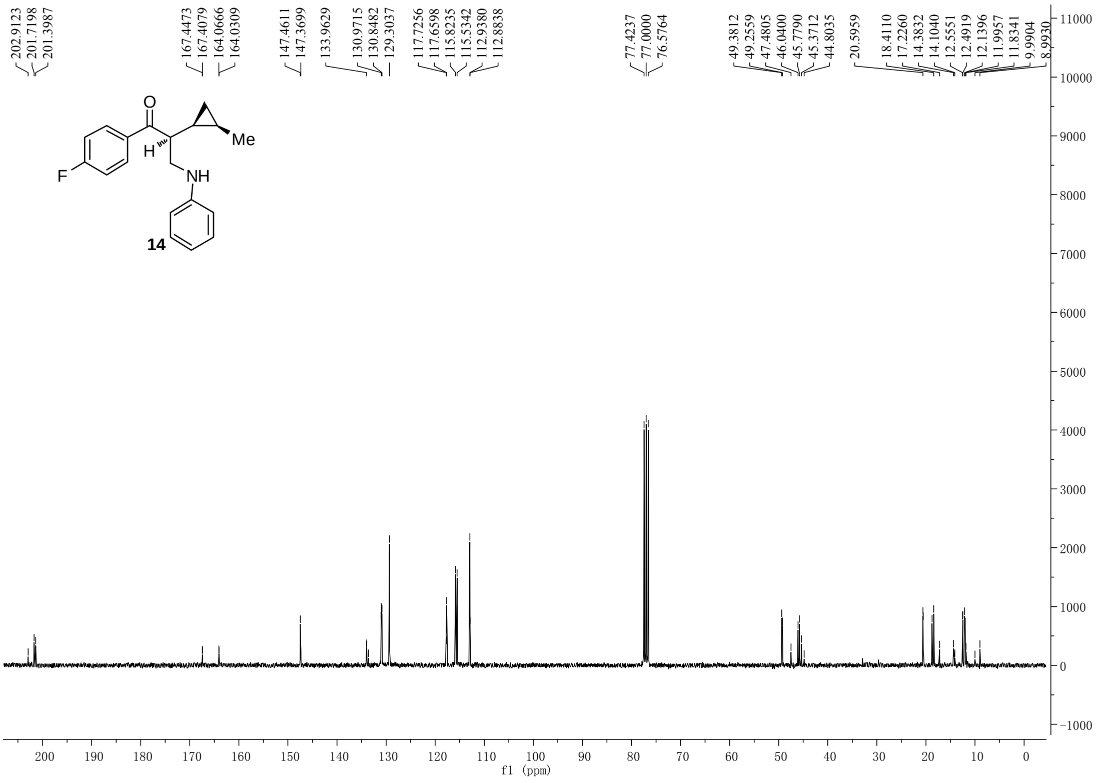

Supplementary Figure 69. ^{1}H and ^{13}C NMR spectra for compound **14**

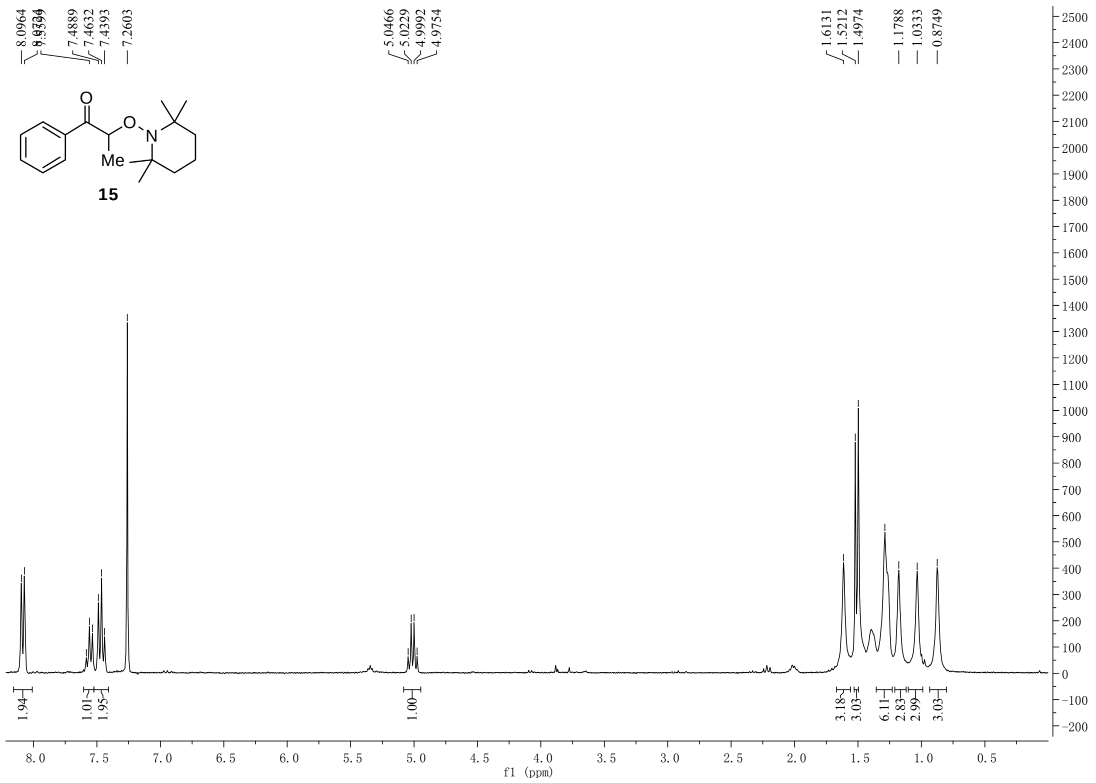

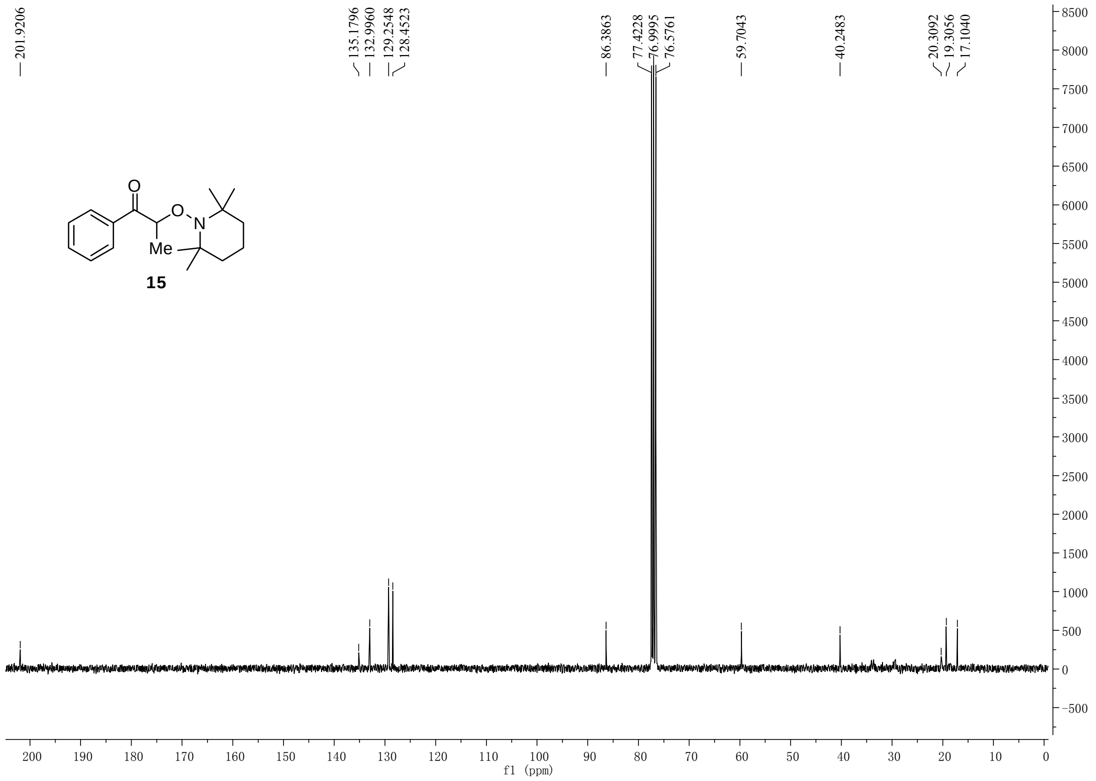

Supplementary Figure 70. ^{1}H and ^{13}C NMR spectra for compound **15**

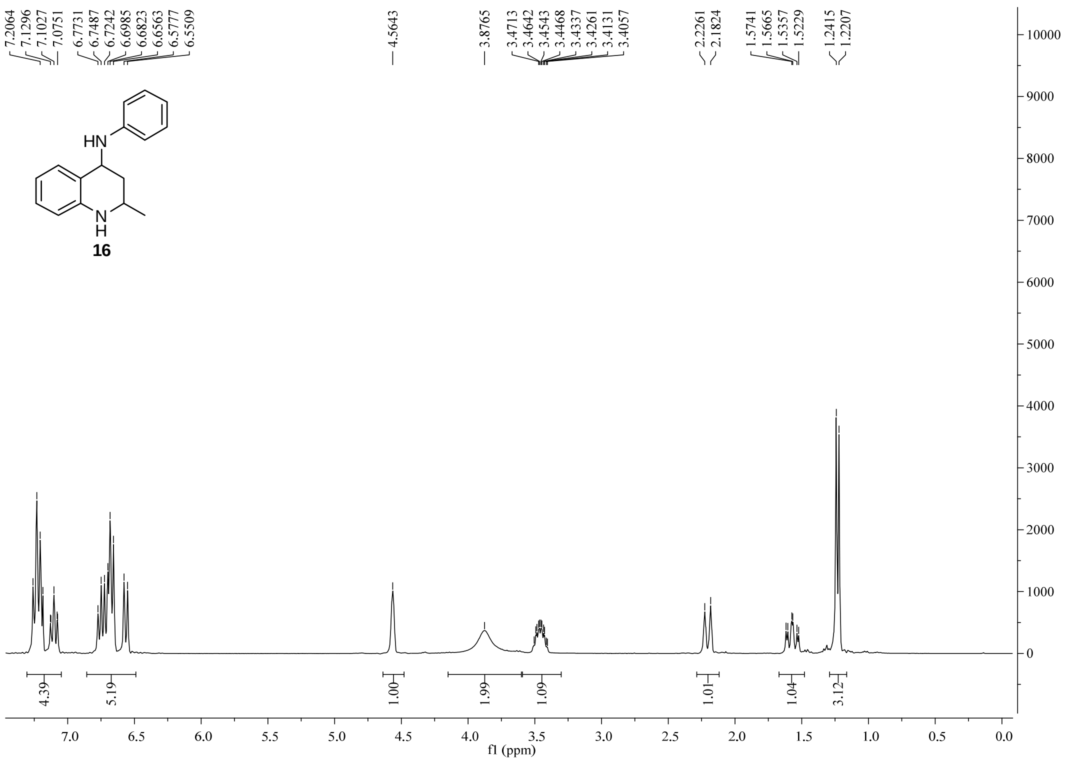

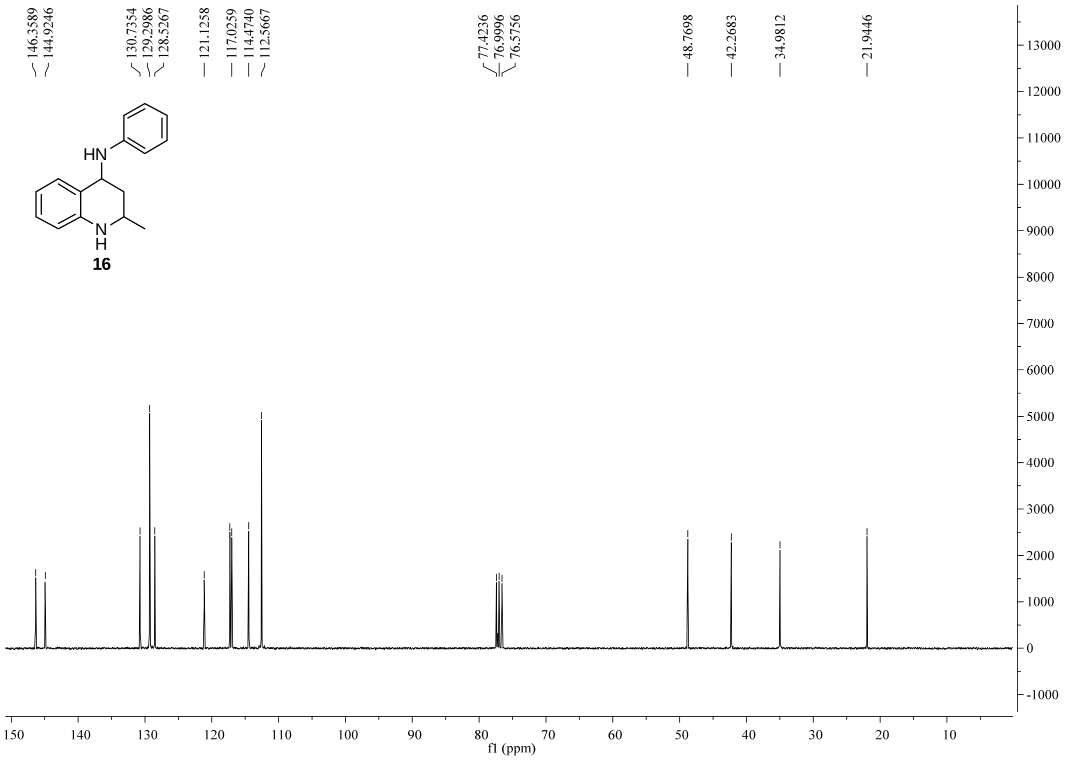

Supplementary Figure 71. ^{1}H and ^{13}C NMR spectra for compound **16**

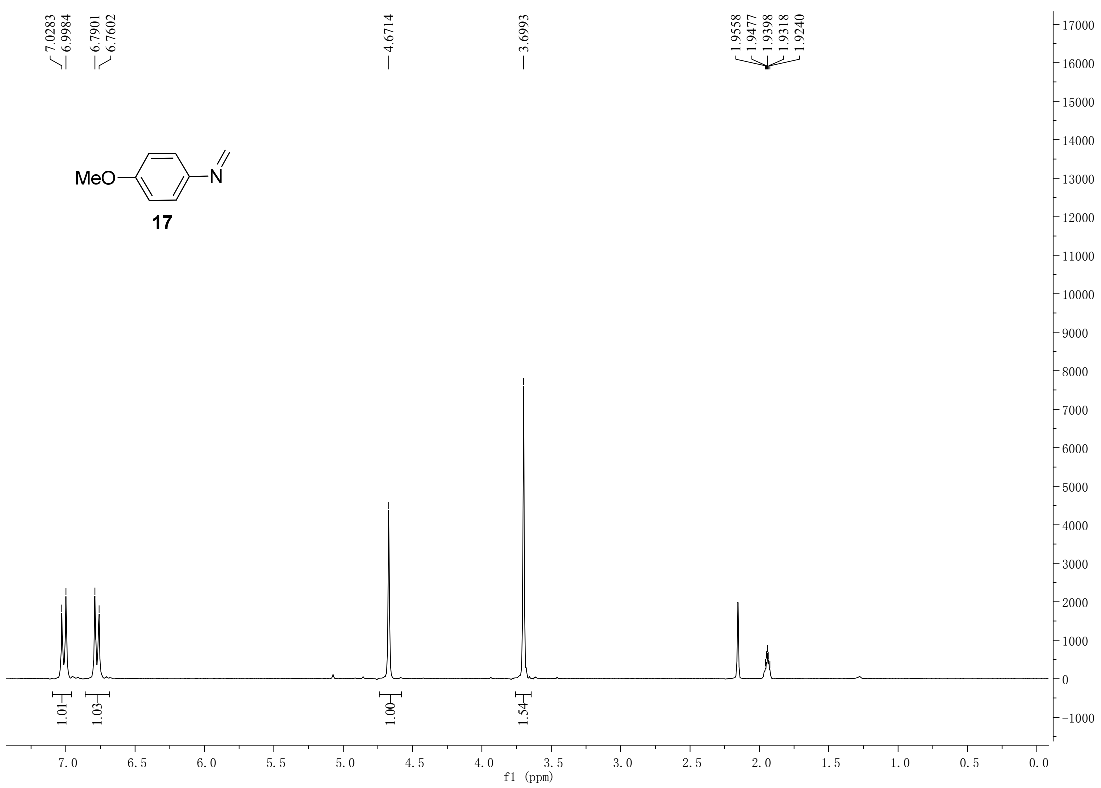

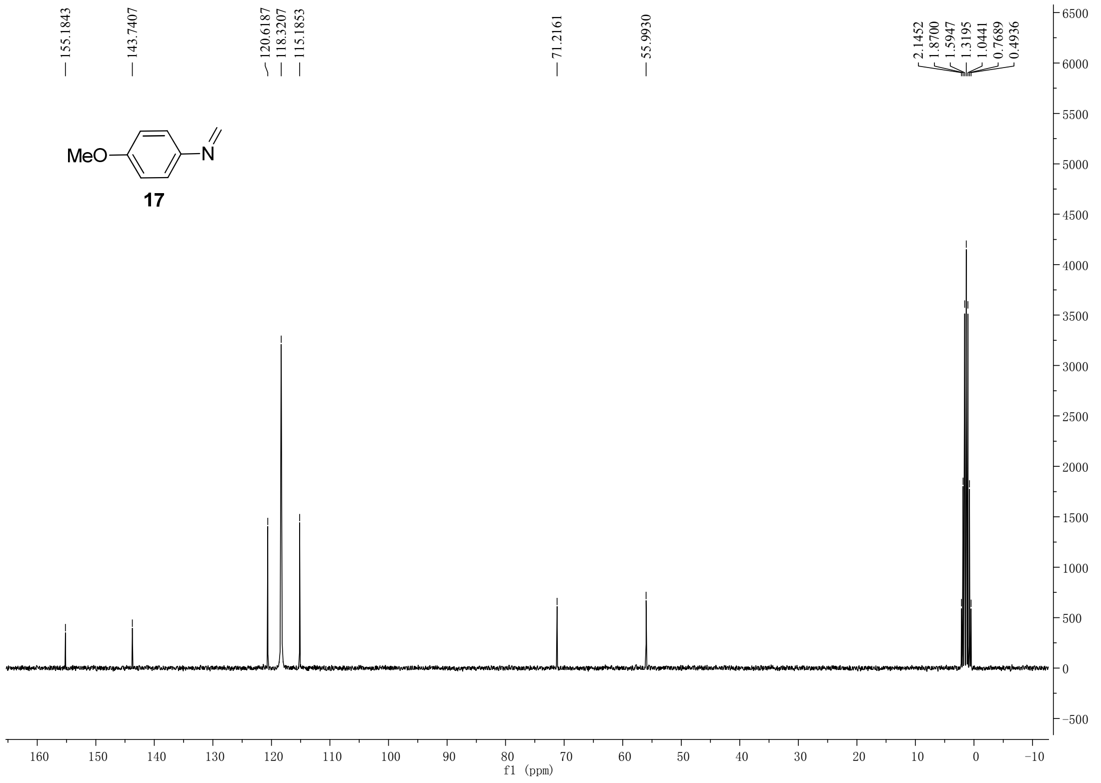

Supplementary Figure 72. ^{1}H and ^{13}C NMR spectra for compound **17**

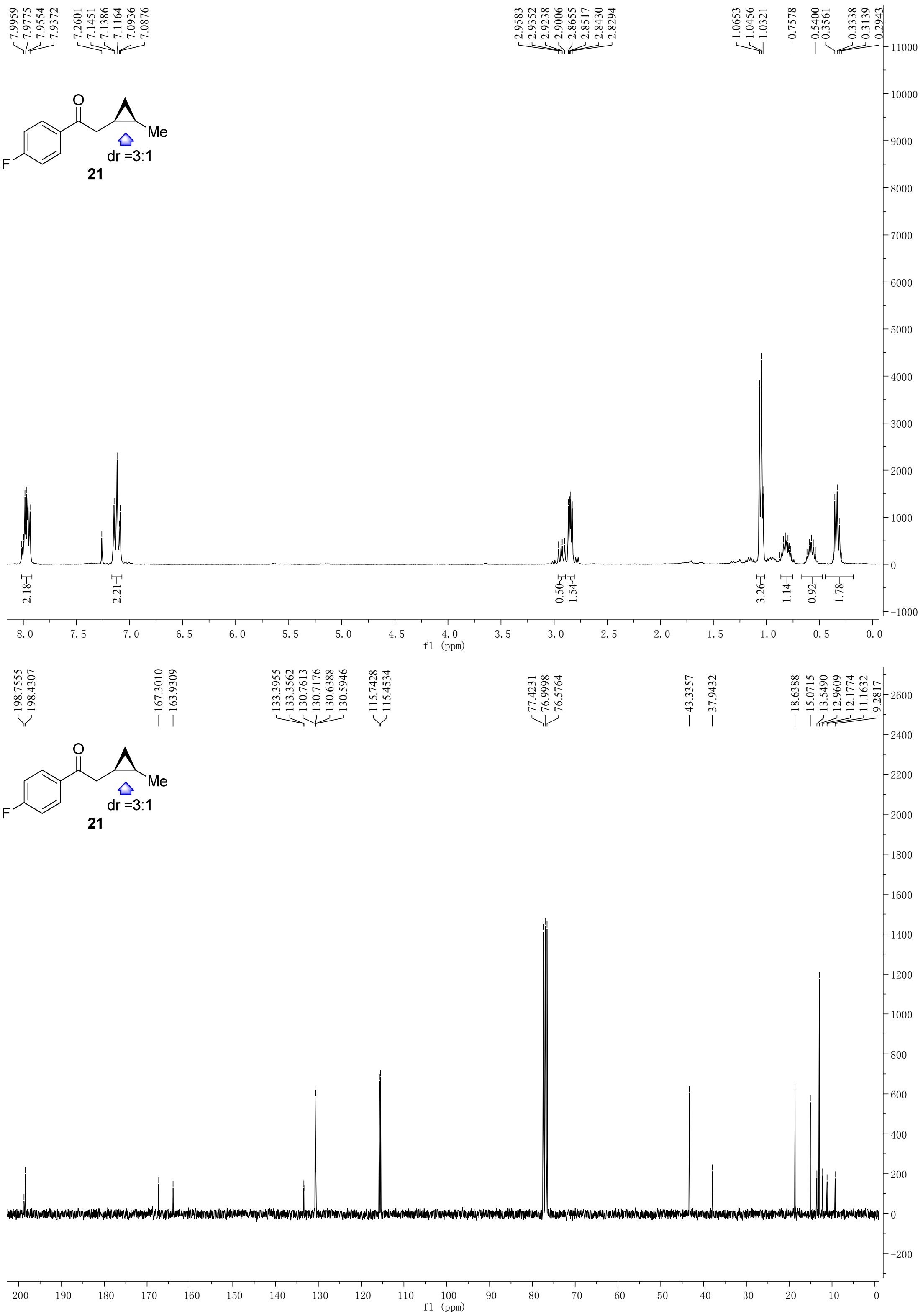

Supplementary Figure 73. ^{1}H and ^{13}C NMR spectra for compound **21**

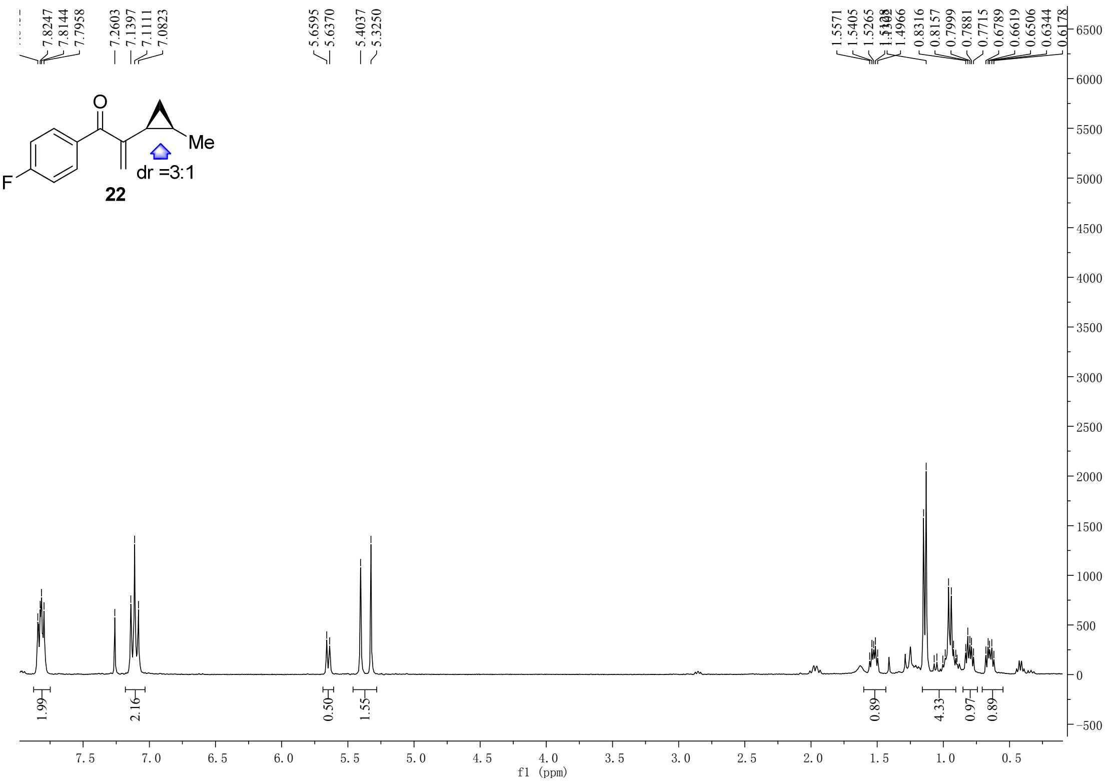

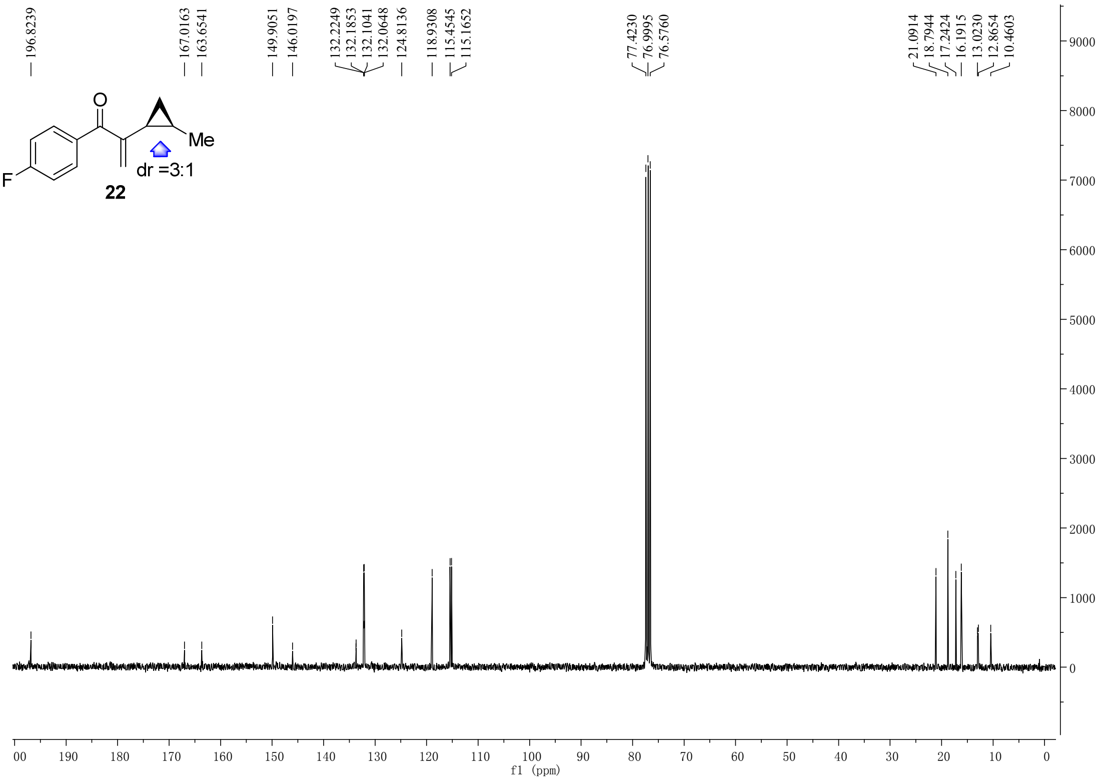

Supplementary Figure 74. ^{1}H and ^{13}C NMR spectra for compound **22**

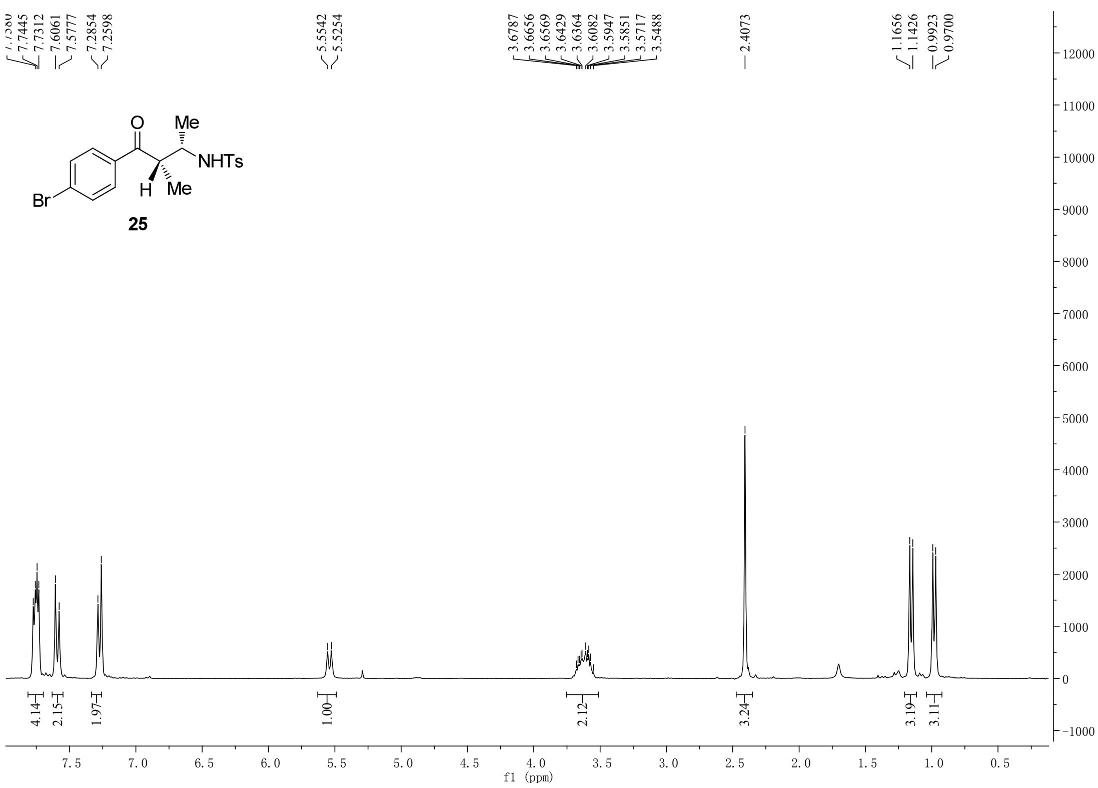

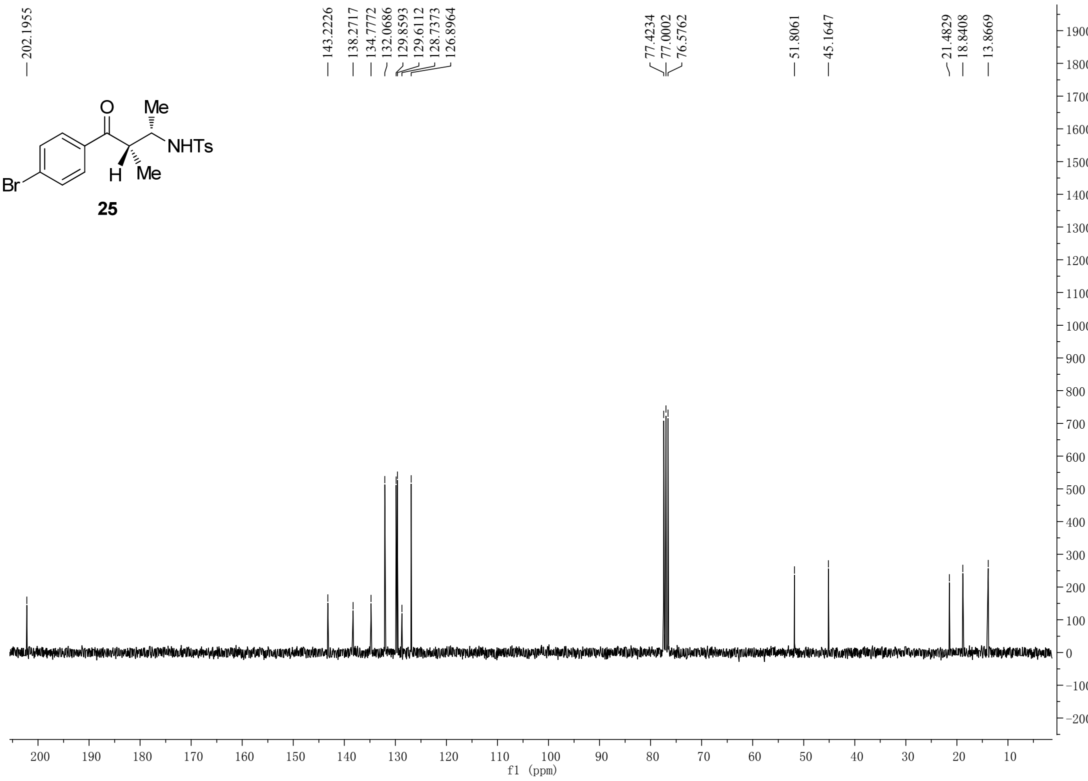

Supplementary Figure 75. ^{1}H and ^{13}C NMR spectra for compound **25**

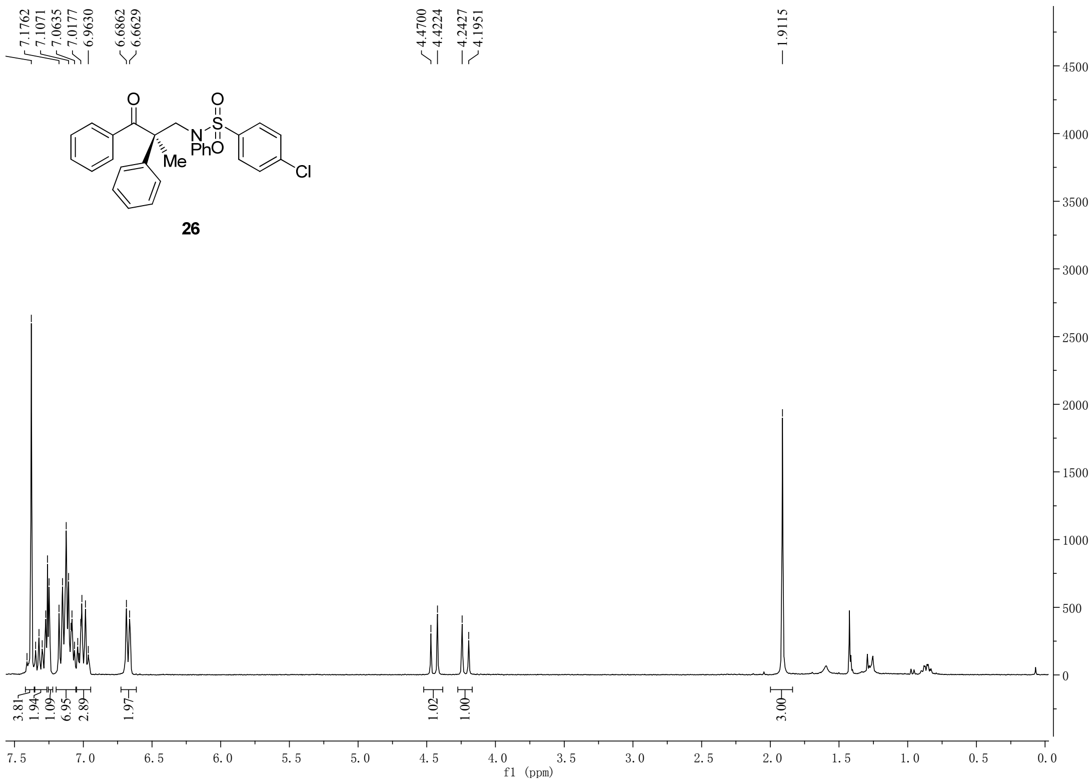

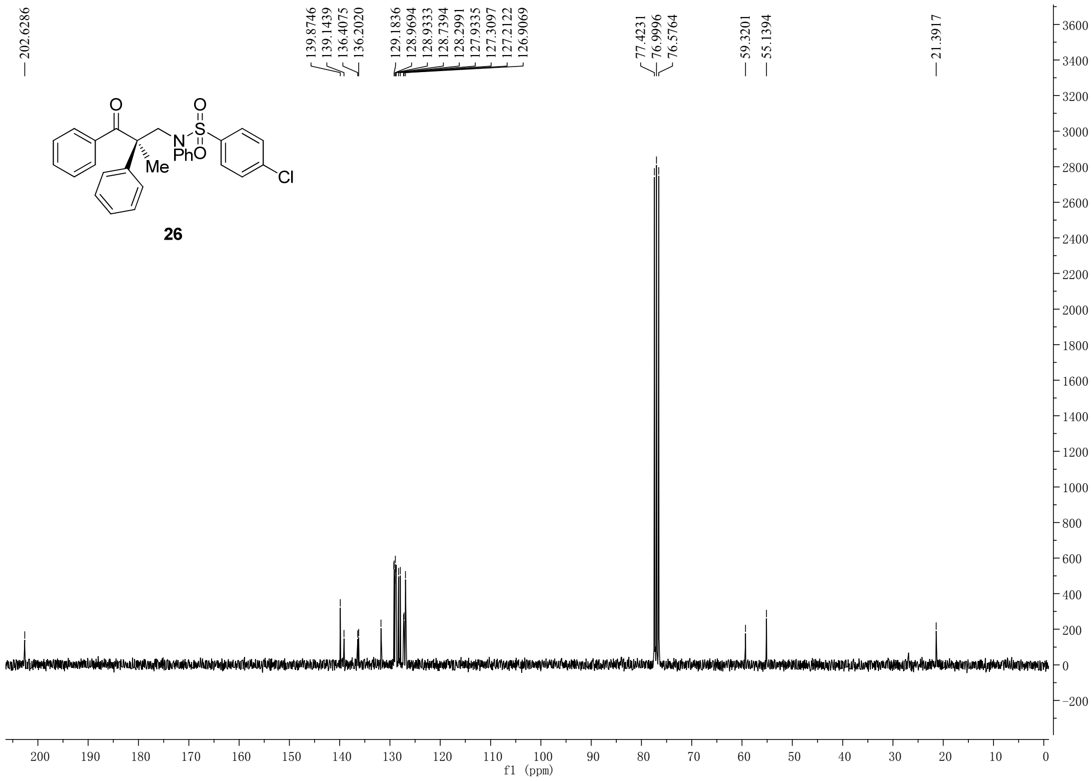

Supplementary Figure 76. ^{1}H and ^{13}C NMR spectra for compound **26**

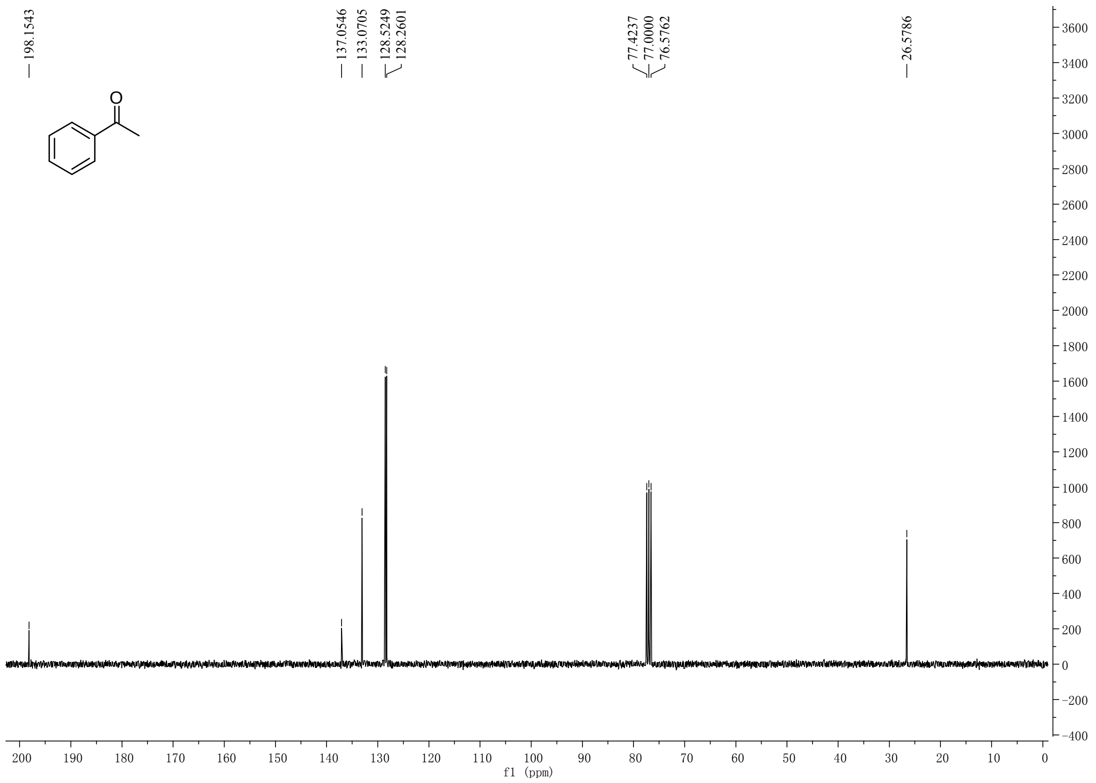

Supplementary Figure 77. ^{13}C NMR spectra for acetophenone

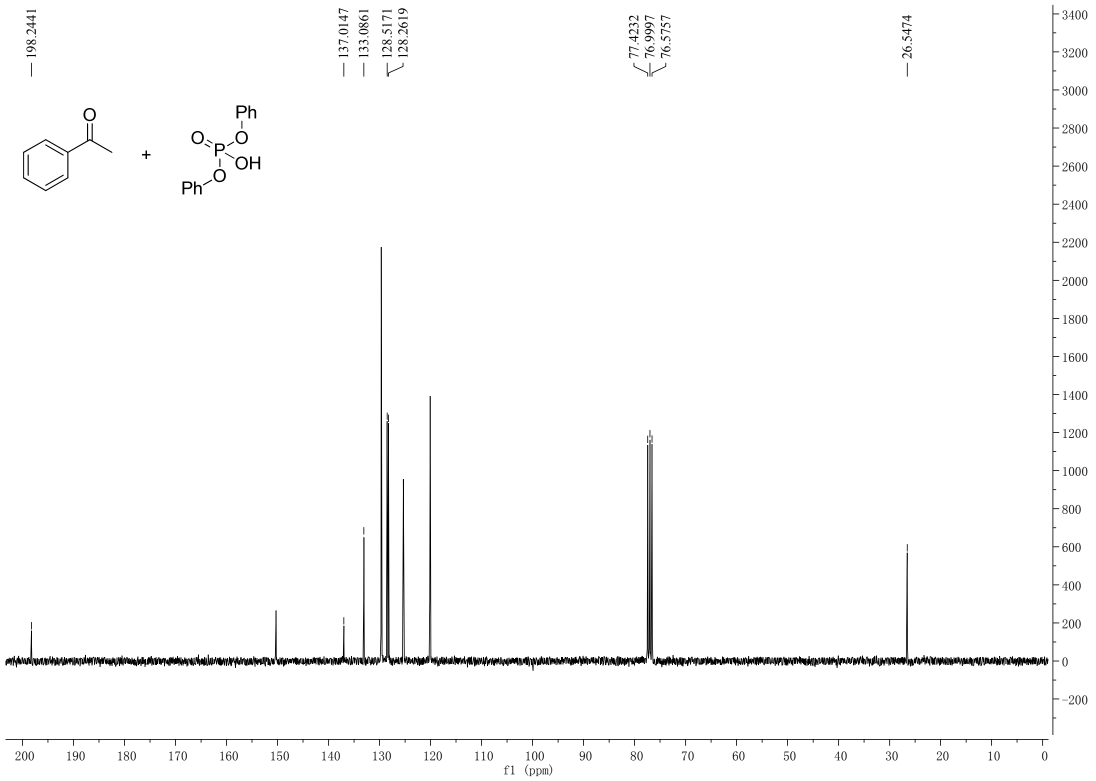

Supplementary Figure 78. ^{13}C NMR spectra for acetophenone with diphenyl phosphate (acetophenone : diphenyl phosphate = 1:1)

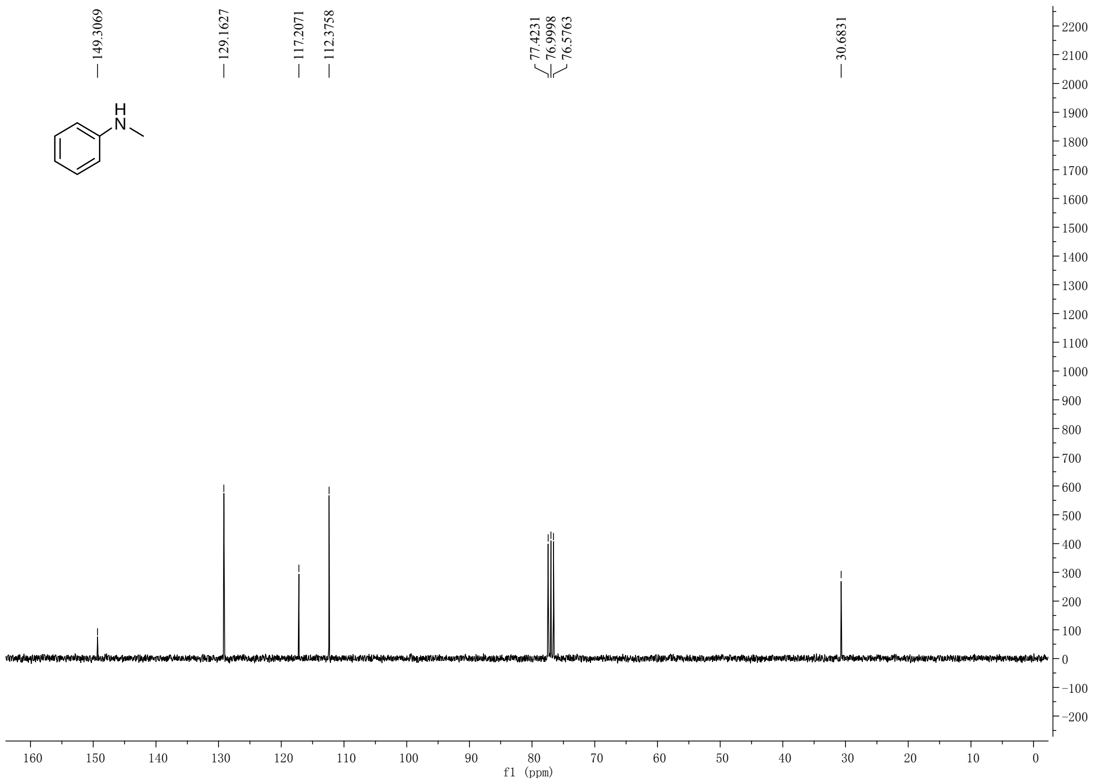

Supplementary Figure 79. ^{13}C NMR spectra for *N*-methylaniline

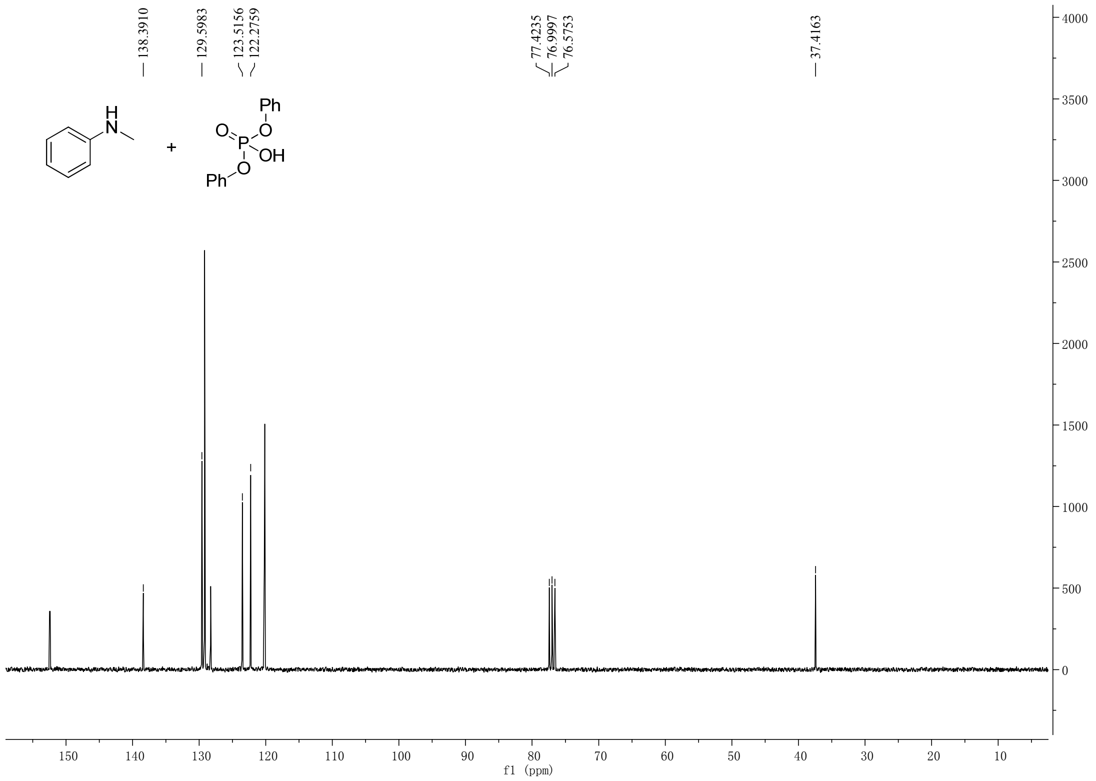

Supplementary Figure 80. ^{13}C NMR spectra for *N*-methylaniline with diphenyl phosphate (*N*-methylaniline : diphenyl phosphate = 1:1)

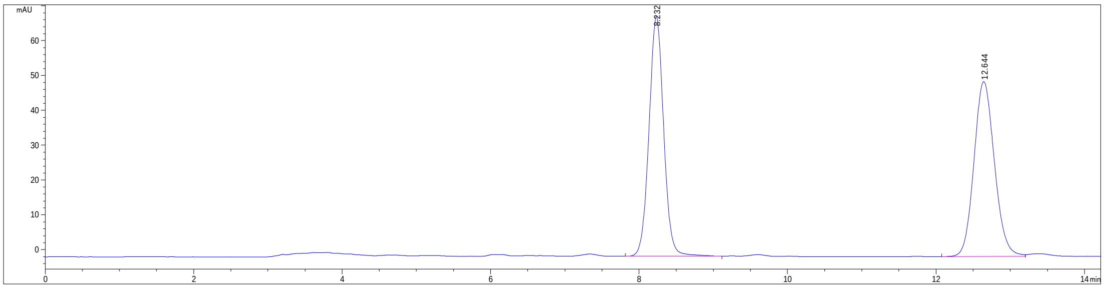

Entry	Retention Time	Area	Height	%Area
1	8.232	943.2	68.6	49.505
2	12.644	962.1	50.2	50.495

Racemic **3a**

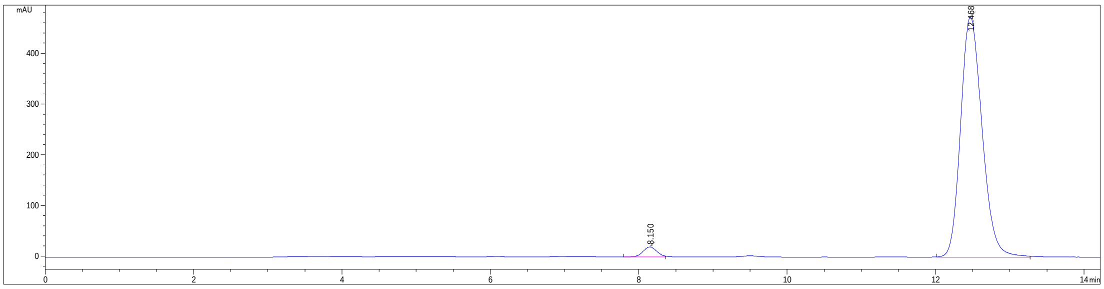

Entry	Retention Time	Area	Height	%Area
1	8.15	257.2	19.8	2.633
2	12.468	9511.2	474	97.367

Enantiomerically enriched **3a**

Supplementary Figure 81. HPLC spectra for compound **3a**

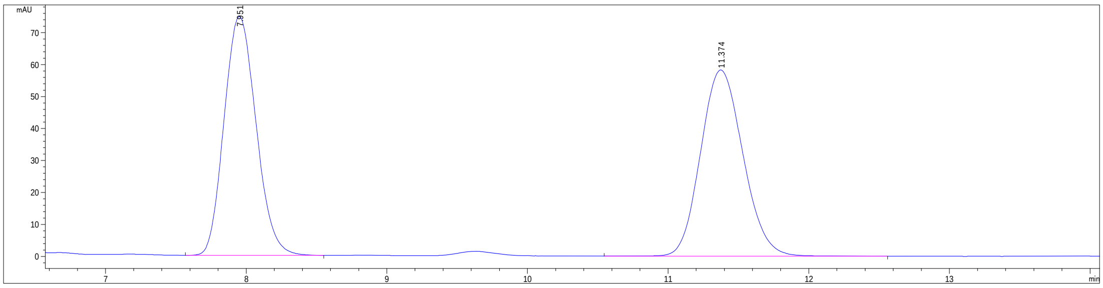

Entry	Retention Time	Area	Height	%Area
1	7.951	1181.8	74.5	49.245
2	11.374	1218.1	58.1	50.755

Racemic **3a**

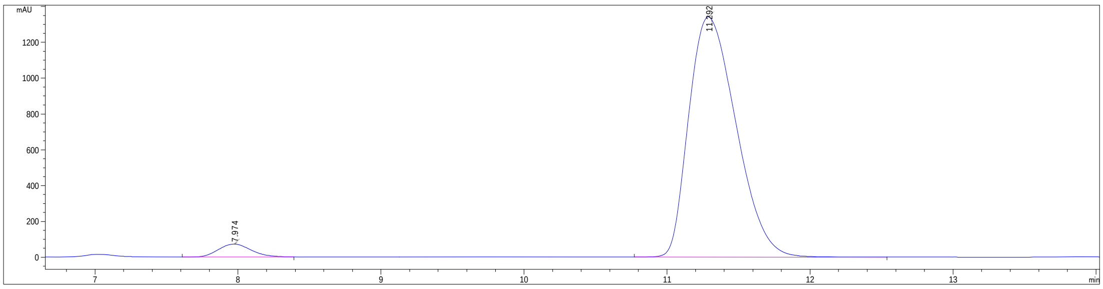

Entry	Retention Time	Area	Height	%Area

1	7.974	1013.1	68.7	3.185
2	11.292	30800.2	1342.3	96.815

Enantiomerically enriched **3a**

Supplementary Figure 82. HPLC spectra for compound **3a** (The synthesis of **3a** in a 1.0 mmol scale).

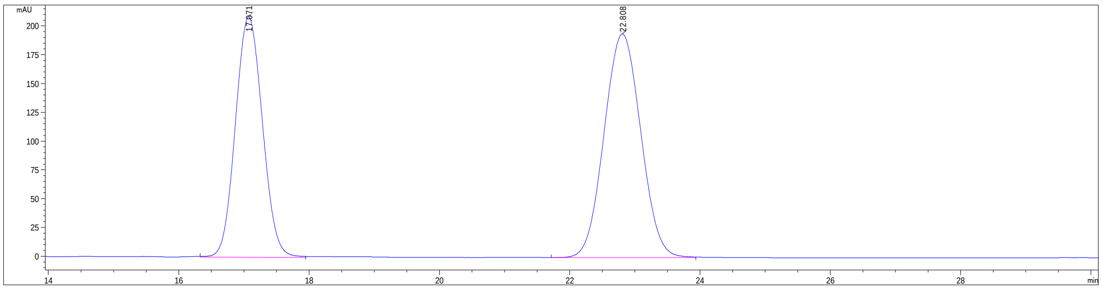

Entry	Retention Time	Area	Height	%Area
1	17.071	6002.2	208.5	49.041
2	22.808	6236.9	177.5	50.959

Racemic **3b**

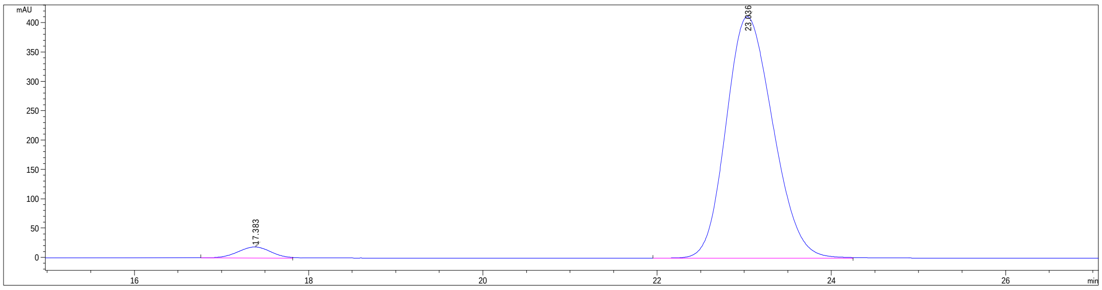

Entry	Retention Time	Area	Height	%Area
1	17.383	501.8	18.8	3.169
2	23.036	15334.7	411.3	96.831

Enantiomerically enriched **3b**

Supplementary Figure 83. HPLC spectra for compound **3b**

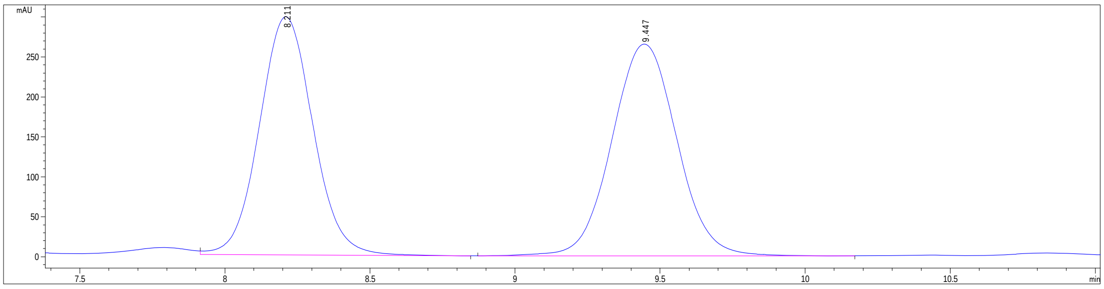

Entry	Retention Time	Area	Height	%Area
1	8.211	3832.3	297.2	49.374
2	9.447	3929.6	265.6	50.626

Racemic **3c**

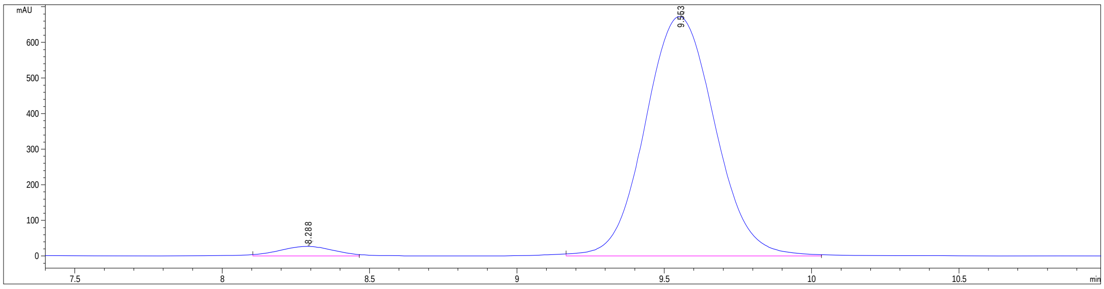

Entry	Retention Time	Area	Height	%Area
1	8.288	340.9	26.7	2.994
2	9.553	11044	673.1	97.006

Enantiomerically enriched **3c**

Supplementary Figure 84. HPLC spectra for compound **3c**

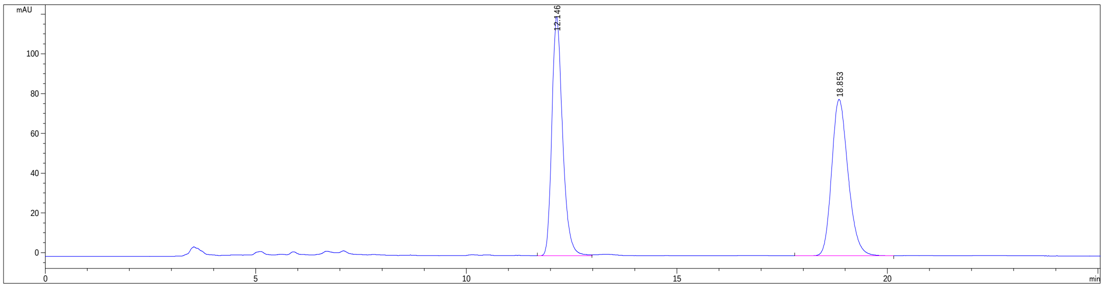

Entry	Retention Time	Area	Height	%Area
1	12.146	2134.3	120.3	50.006
2	18.853	2133.8	78.6	49.994

Racemic **3d**

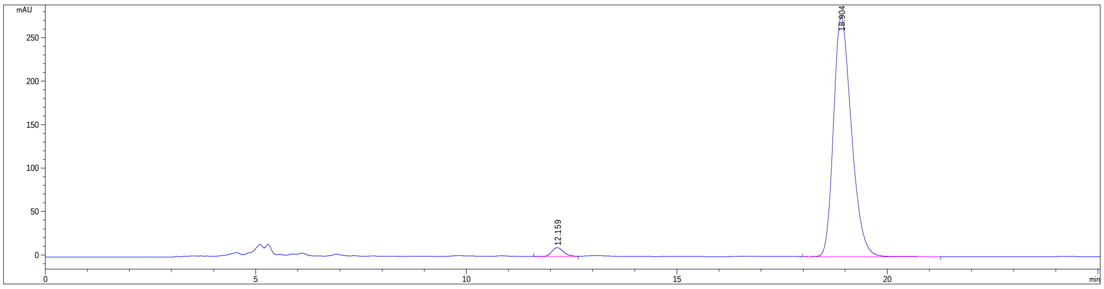

Entry	Retention Time	Area	Height	%Area
1	12.159	201.2	10.2	2.407
2	18.902	8158.6	276.3	97.593

Enantiomerically enriched **3d**

Supplementary Figure 85. HPLC spectra for compound **3d**

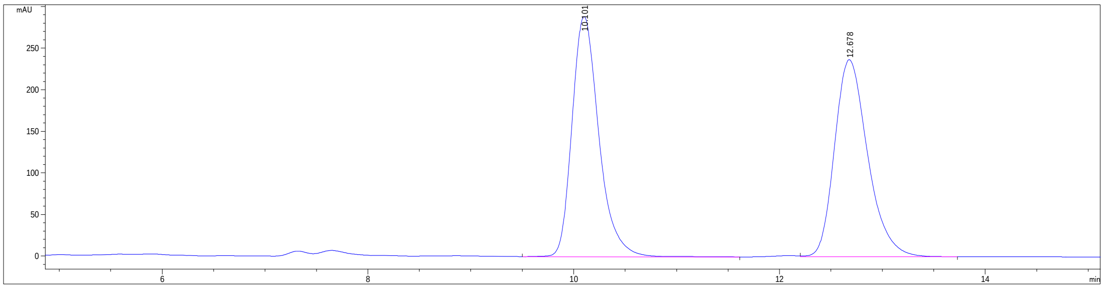

Entry	Retention Time	Area	Height	%Area
1	10.101	5229.5	288.3	49.665
2	12.678	5300.2	236.6	50.335

Racemic **3e**

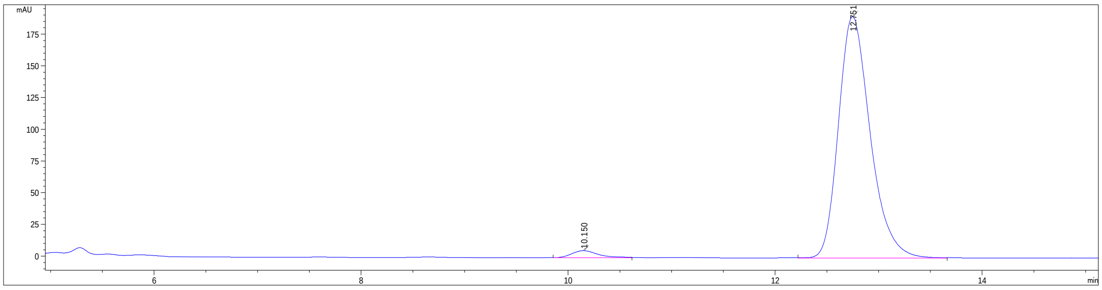

Entry	Retention Time	Area	Height	%Area
1	10.15	103.6	5.6	2.491
2	12.751	4054.3	190.4	97.509

Enantiomerically enriched **3e**

Supplementary Figure 86. HPLC spectra for compound **3e**

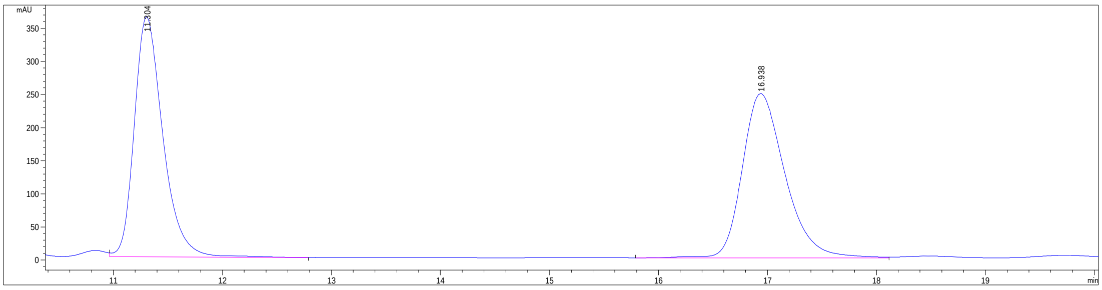

Entry	Retention Time	Area	Height	%Area
1	11.304	6620.7	362.7	49.473
2	16.938	6761.7	248.2	50.527

Racemic **3f**

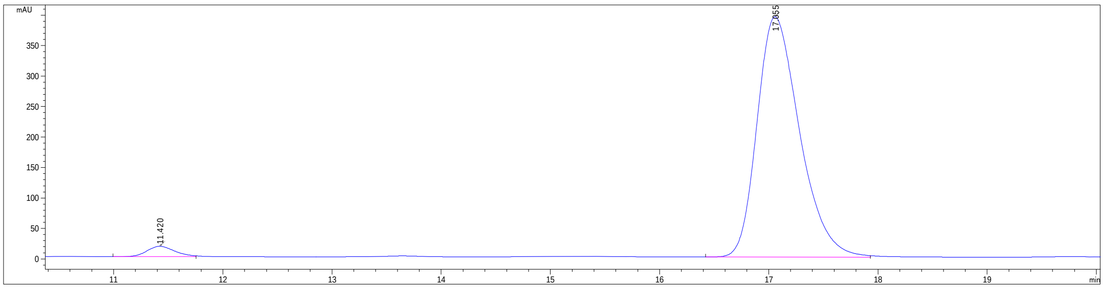

Entry	Retention Time	Area	Height	%Area
1	11.42	304	17.1	2.774
2	17.055	10654	394.4	97.226

Enantiomerically enriched **3f**

Supplementary Figure 87. HPLC spectra for compound **3f**

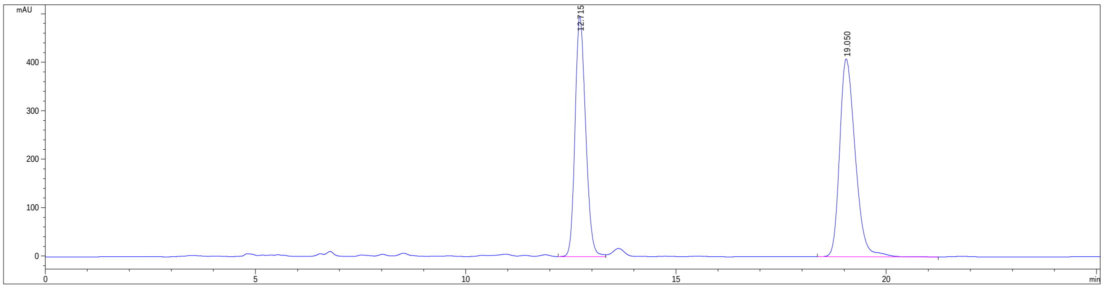

Entry	Retention Time	Area	Height	%Area
1	12.715	8683.1	494	49.276
2	19.05	8938.1	408.7	50.724

Racemic **3g**

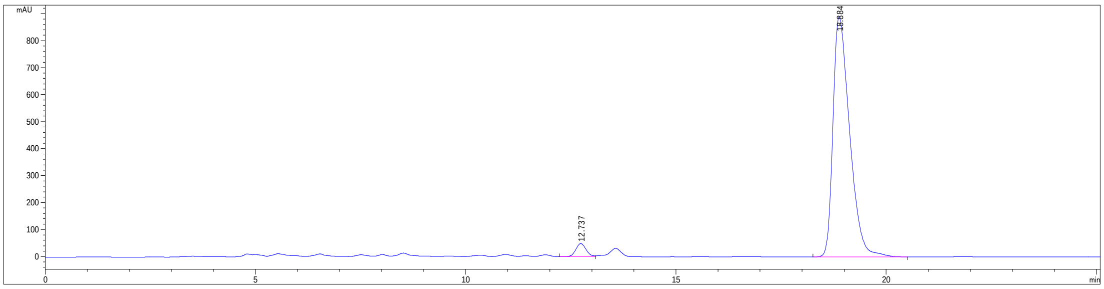

Entry	Retention Time	Area	Height	%Area
1	12.737	839.6	48.6	3.239
2	18.884	25079	894.4	96.761

Enantiomerically enriched **3g**

Supplementary Figure 88. HPLC spectra for compound **3g**

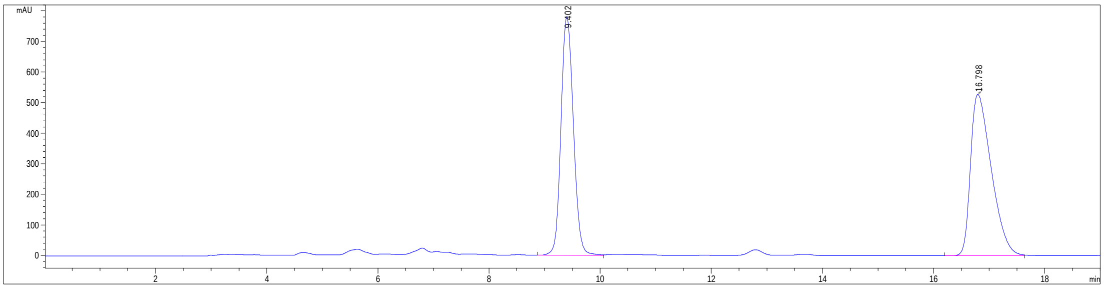

Entry	Retention Time	Area	Height	%Area
1	9.402	12105.3	779	46.856
2	16.798	13730	528.1	53.144

Racemic **3h**

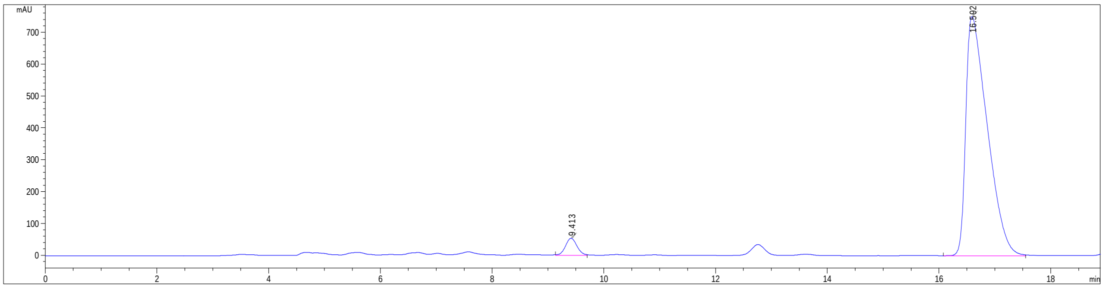

Entry	Retention Time	Area	Height	%Area

1	9.413	766.3	53.7	3.682
2	16.592	20044.9	751.7	96.318

Enantiomerically enriched **3h**

Supplementary Figure 89. HPLC spectra for compound **3h**

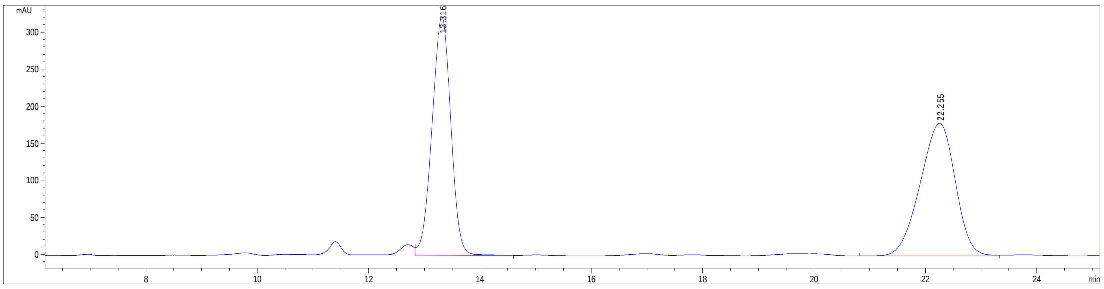

Entry	Retention Time	Area	Height	%Area
1	13.316	7553.3	321.7	49.316
2	22.255	7762.9	179.2	50.684

Racemic **3i**

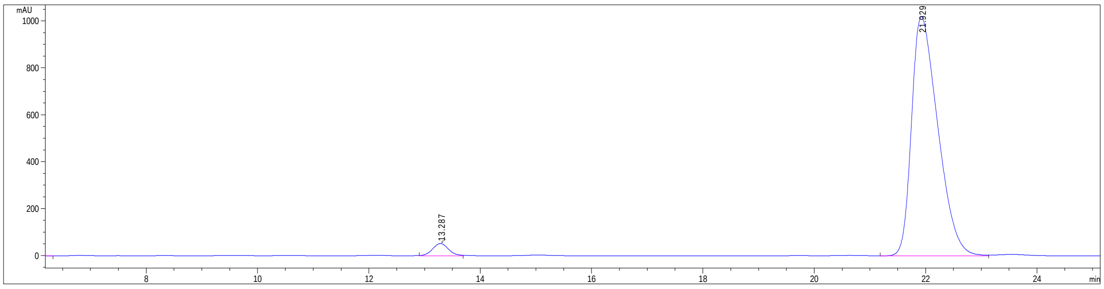

Entry	Retention Time	Area	Height	%Area
1	13.287	1059.4	52.8	3.072
2	21.929	33427.2	1019.7	96.928

Enantiomerically enriched **3i**

Supplementary Figure 90. HPLC spectra for compound **3i**

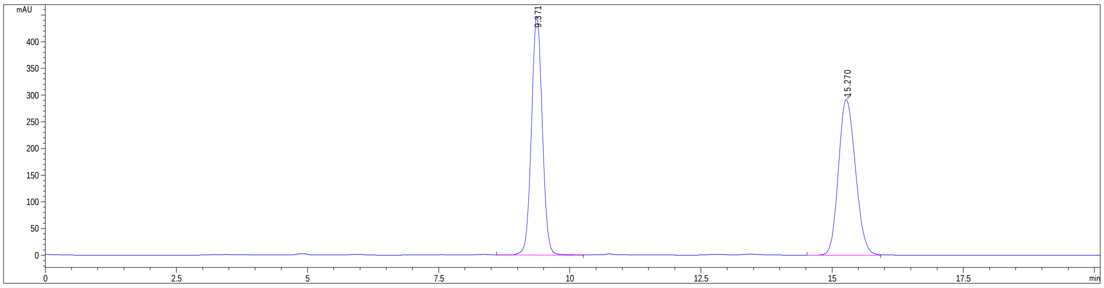

Entry	Retention Time	Area	Height	%Area
1	9.371	6452.2	446.4	49.499
2	15.27	6582.8	292.1	50.501

Racemic **3j**

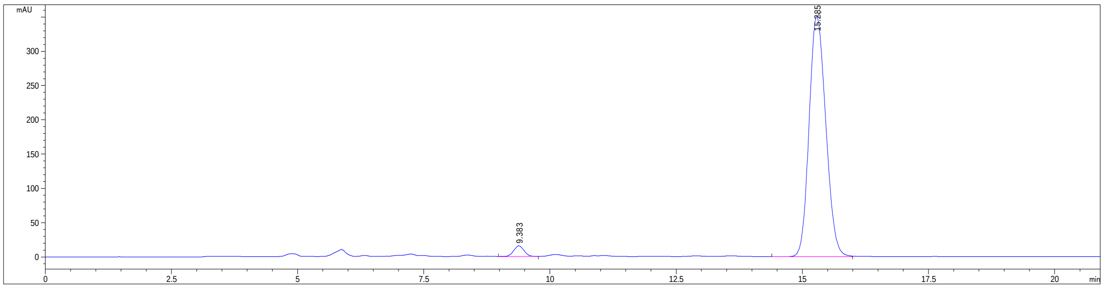

Entry	Retention Time	Area	Height	%Area
1	9.383	218.1	15.3	2.633
2	15.285	8065.3	350.6	97.367

Enantiomerically enriched **3j**

Supplementary Figure 91. HPLC spectra for compound **3j**

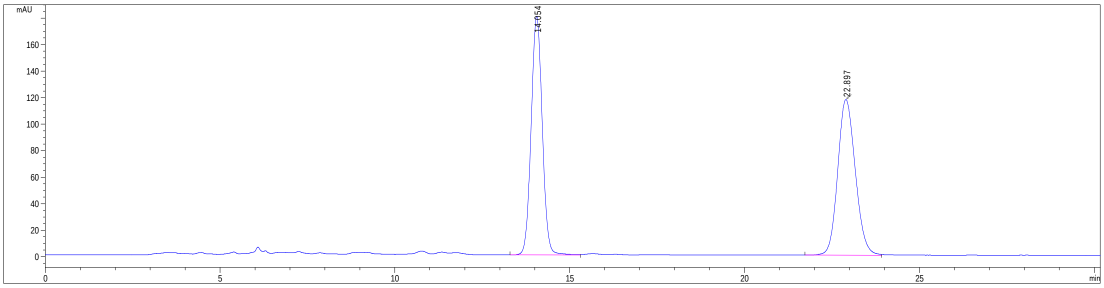

Entry	Retention Time	Area	Height	%Area
1	14.054	3974.1	179.6	49.243
2	22.897	4096.3	117.4	50.757

Racemic **3k**

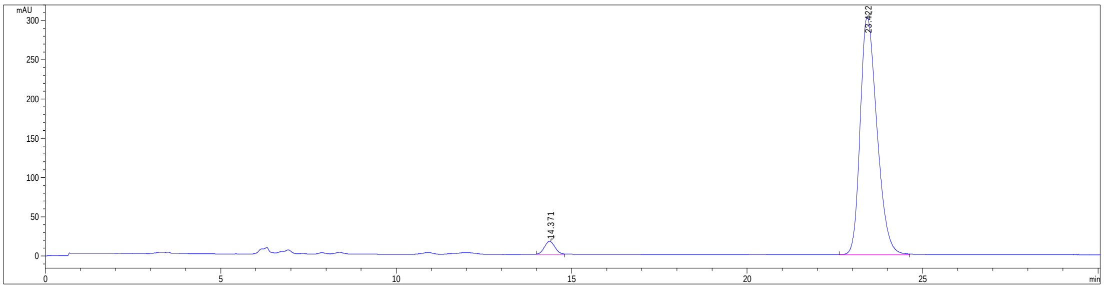

Entry	Retention Time	Area	Height	%Area
1	14.371	353.4	16.7	3.401
2	23.422	10039.7	303.1	96.599

Enantiomerically enriched **3k**

Supplementary Figure 92. HPLC spectra for compound **3k**

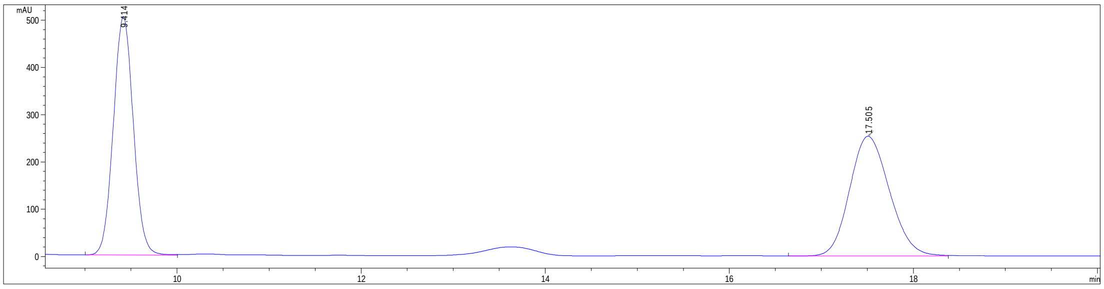

Entry	Retention Time	Area	Height	%Area
1	9.414	7634.6	503.8	50.063
2	17.505	7615.4	253.6	49.937

Racemic **3l**

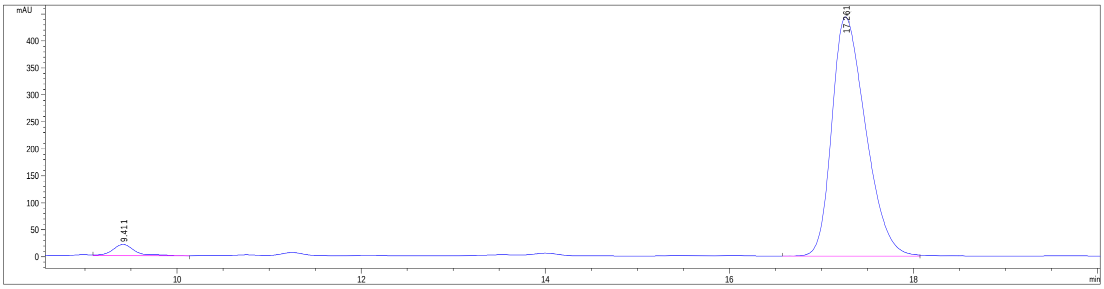

Entry	Retention Time	Area	Height	%Area
1	9.411	349.7	20.9	3.003
2	17.261	11294.1	444.1	96.997

Enantiomerically enriched **3l**

Supplementary Figure 93. HPLC spectra for compound **3l**

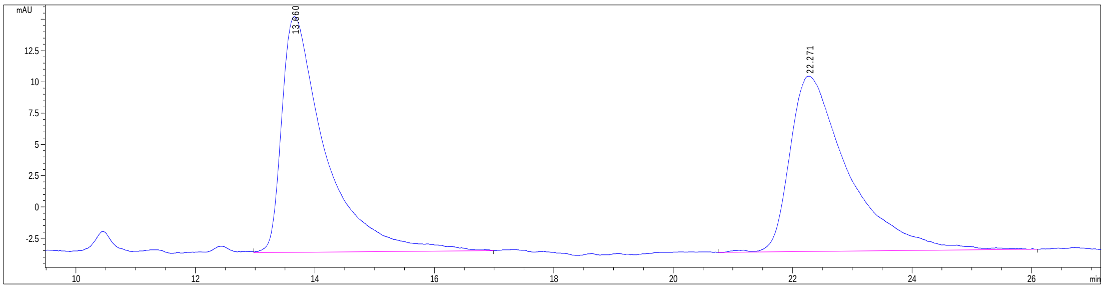

Entry	Retention Time	Area	Height	%Area
1	13.66	955.6	18.8	49.988
2	22.271	956	14	50.012

Racemic **3m**

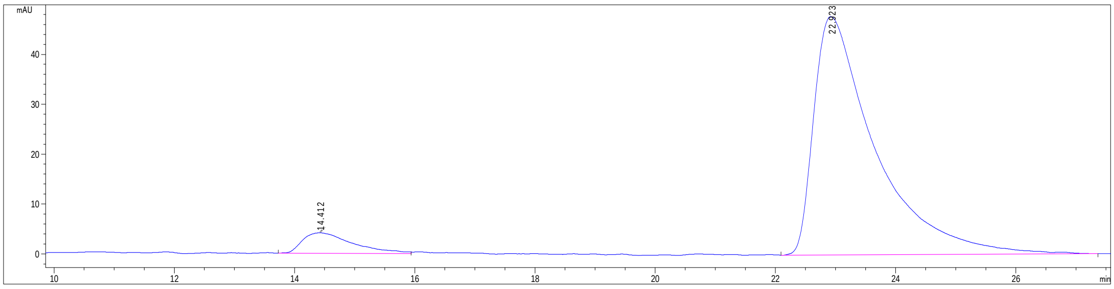

Entry	Retention Time	Area	Height	%Area
1	14.412	222.6	4.1	6.170
2	22.923	3385.7	47.8	93.830

Enantiomerically enriched **3m**

Supplementary Figure 94. HPLC spectra for compound **3m**

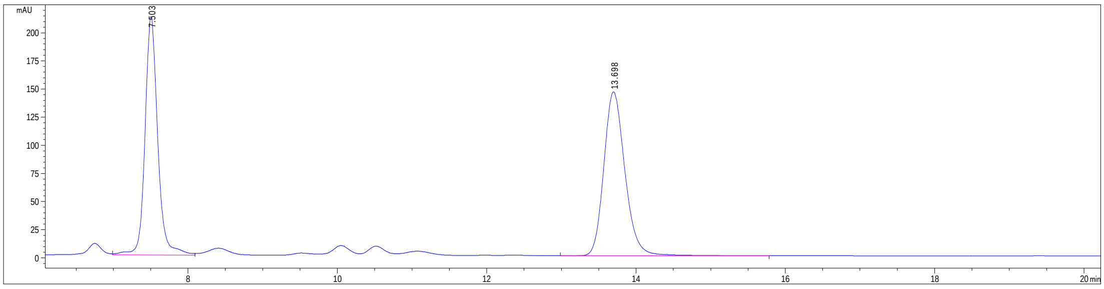

Entry	Retention Time	Area	Height	%Area
1	7.503	2507.7	211.4	49.549
2	13.696	2553.4	140.8	50.451

Racemic **3n**

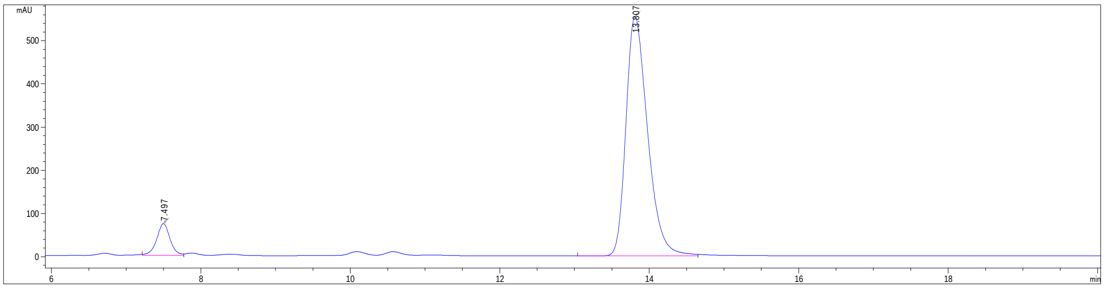

Entry	Retention Time	Area	Height	%Area
1	7.497	869.9	73.6	7.230
2	13.807	11162.7	554.7	92.770

Enantiomerically enriched **3n**

Supplementary Figure 95. HPLC spectra for compound **3n**

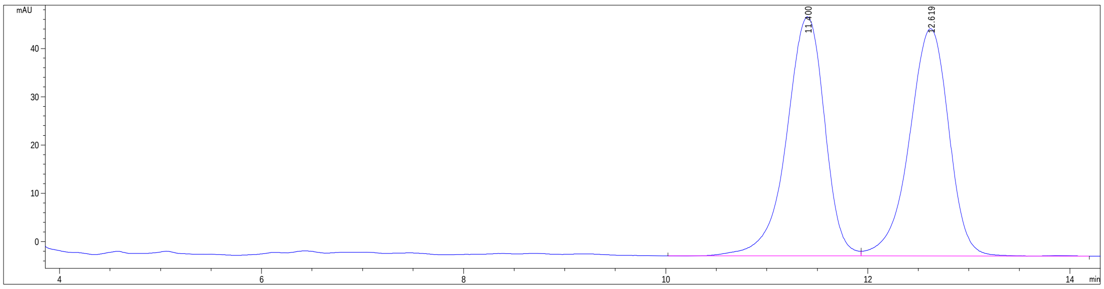

Entry	Retention Time	Area	Height	%Area
1	11.4	1301.3	49.5	50.411
2	12.619	1280	47.1	49.589

Racemic **3o**

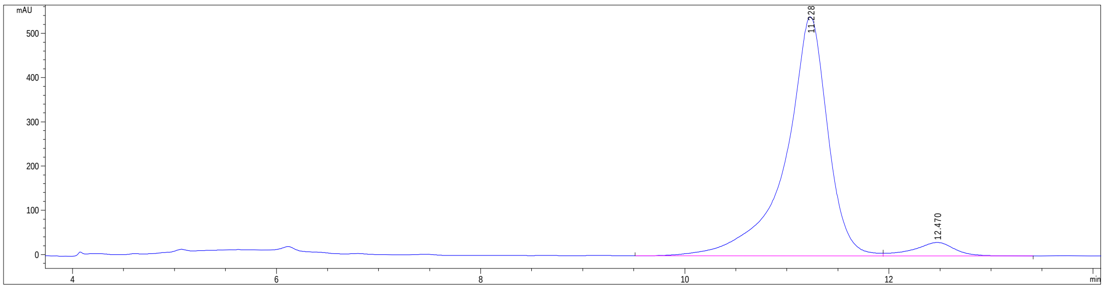

Entry	Retention Time	Area	Height	%Area
1	11.228	15524.2	539.8	94.809

2	12.47	850	30.4	5.191

Enantiomerically enriched **3o**

Supplementary Figure 96. HPLC spectra for compound **3o**

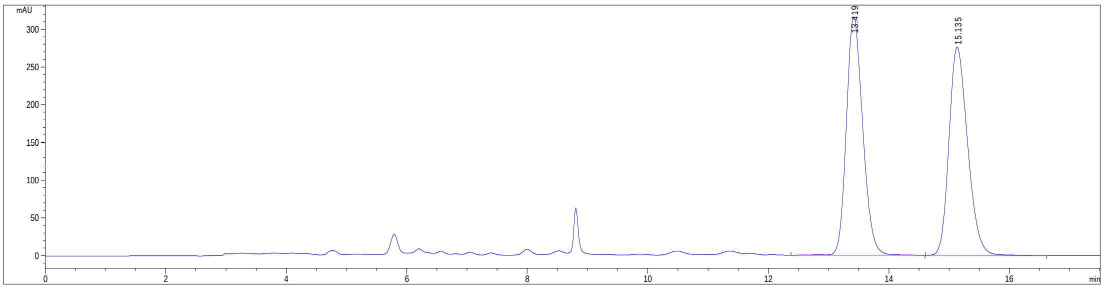

Entry	Retention Time	Area	Height	%Area
1	13.419	5996.9	315.6	50.268
2	15.135	5933	276.1	49.732

Racemic **3p**

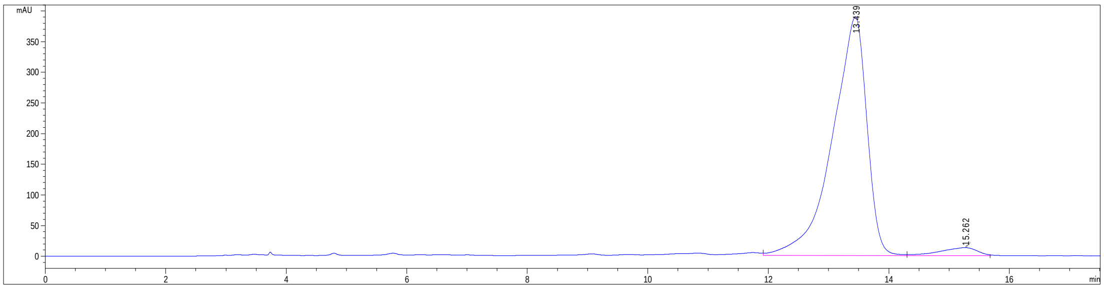

Entry	Retention Time	Area	Height	%Area
1	13.439	15422.6	389.2	96.831
2	15.273	504.8	13	3.169

Enantiomerically enriched **3p**

Supplementary Figure 97. HPLC spectra for compound **3p**

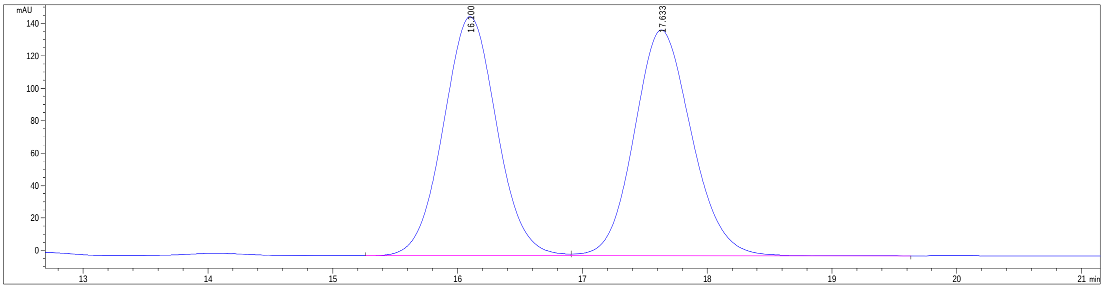

Entry	Retention Time	Area	Height	%Area
1	16.1	4398.9	147.4	49.702
2	17.633	4451.8	139.2	50.298

Racemic **3q**

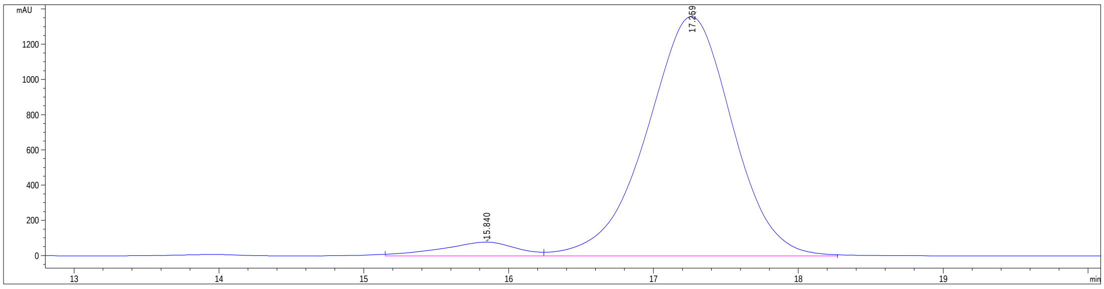

Entry	Retention Time	Area	Height	%Area
1	15.84	2819.4	78.6	4.815
2	17.259	55736.5	1359.8	95.185

Enantiomerically enriched **3q**

Supplementary Figure 98. HPLC spectra for compound **3q**

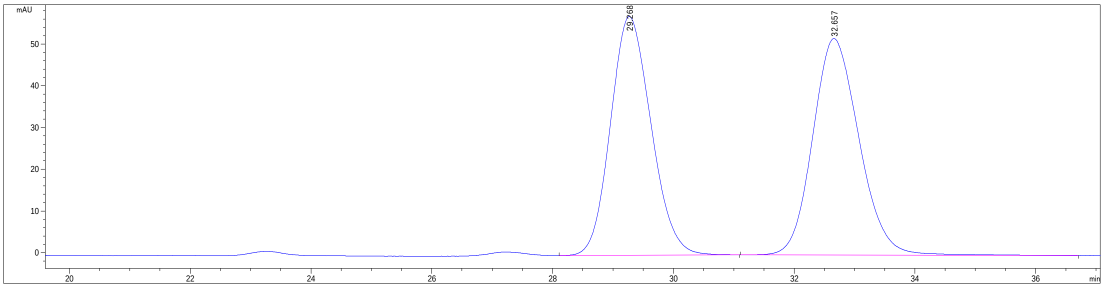

Entry	Retention Time	Area	Height	%Area
1	29.268	2668	57.2	49.174
2	32.657	2757.6	51.9	50.826

Racemic **3r**

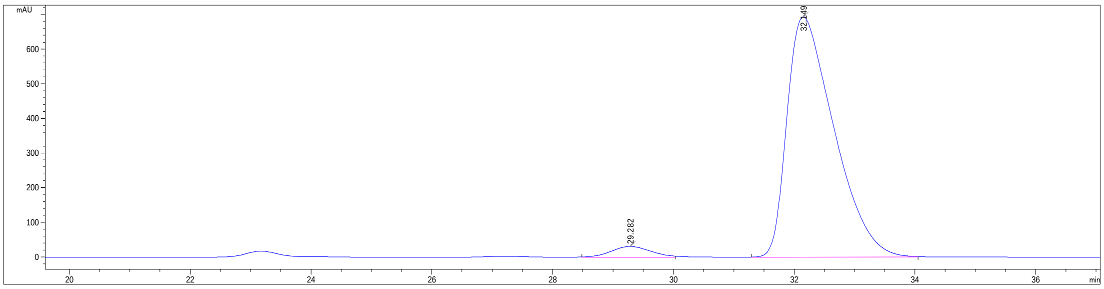

Entry	Retention Time	Area	Height	%Area
1	29.282	1392.2	31.1	3.581
2	32.149	37489.3	693.4	96.419

Enantiomerically enriched **3r**

Supplementary Figure 99. HPLC spectra for compound **3r**

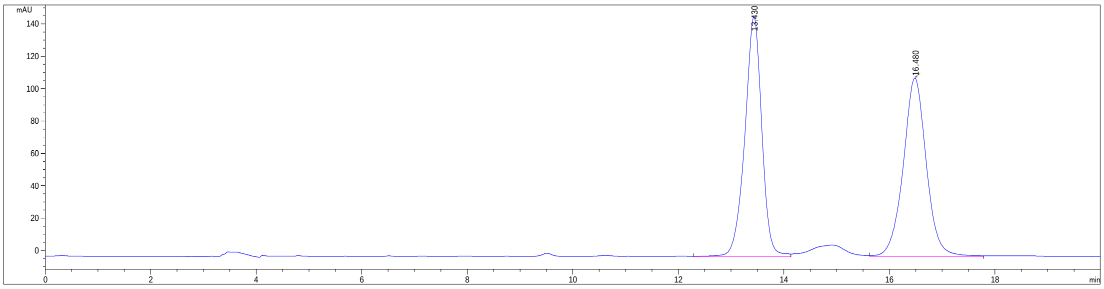

Entry	Retention Time	Area	Height	%Area

1	13.43	3299.4	147.8	49.634
2	16.48	3348	110.3	50.366

Racemic **3s**

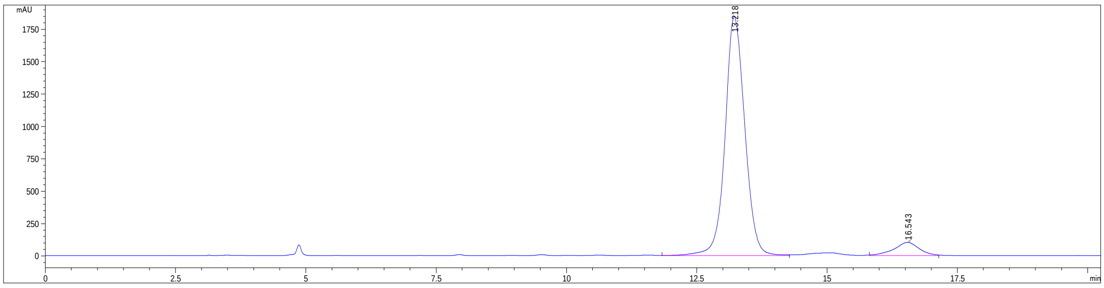

Entry	Retention Time	Area	Height	%Area
1	13.221	48705.9	1841.2	93.316
2	16.543	3488.7	102.8	6.684

Enantiomerically enriched **3s**

Supplementary Figure 100. HPLC spectra for compound **3s**

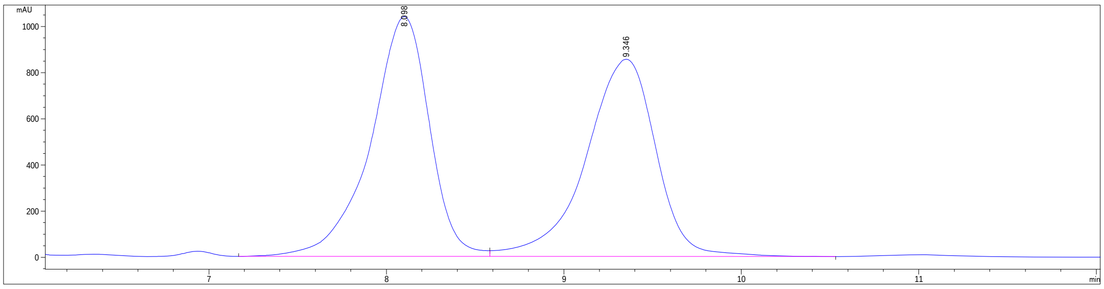

Entry	Retention Time	Area	Height	%Area
1	8.098	23944.3	1037.5	50.537
2	9.346	23435.9	853.6	49.463

Racemic **3t**

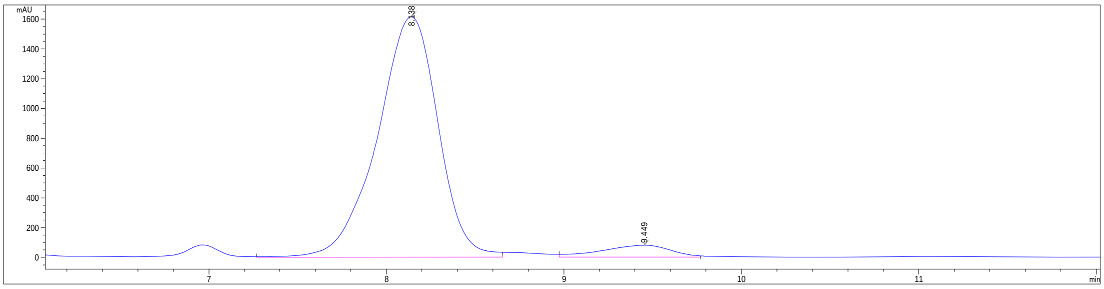

Entry	Retention Time	Area	Height	%Area
1	8.138	38227.2	1615.4	94.924
2	9.449	2044.2	79.2	5.076

Enantiomerically enriched **3t**

Supplementary Figure 101. HPLC spectra for compound **3t**

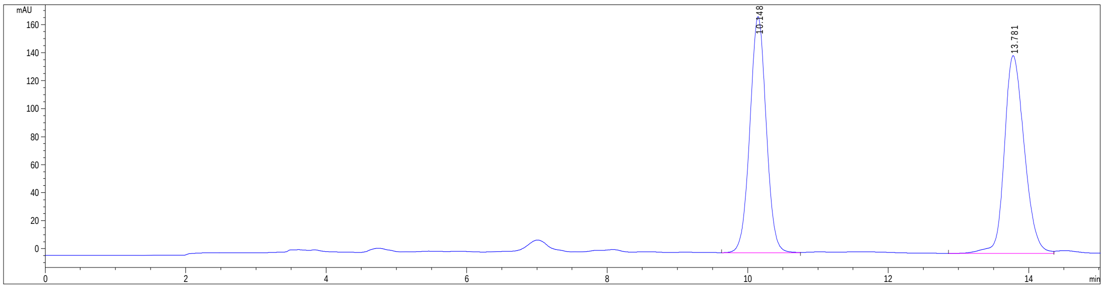

Entry	Retention Time	Area	Height	%Area
1	10.148	2723	168.4	49.418
2	13.781	2787.2	140.8	50.582

Racemic **3u**

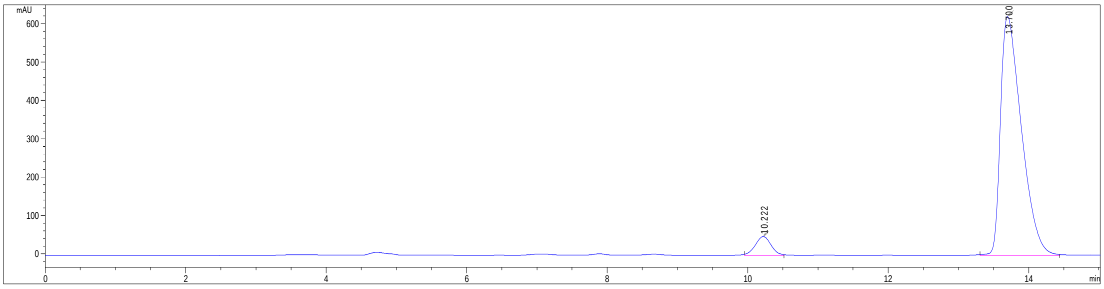

Entry	Retention Time	Area	Height	%Area
1	10.222	651.3	45.6	4.828
2	13.7	12838.9	622.9	95.172

Enantiomerically enriched **3u**

Supplementary Figure 102. HPLC spectra for compound **3u**

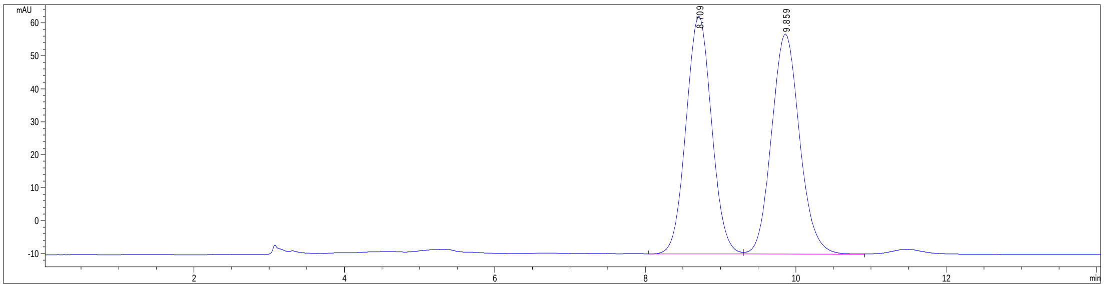

Entry	Retention Time	Area	Height	%Area
1	8.709	1703.8	72	49.644
2	9.859	1728.3	66.7	50.356

Racemic **3v**

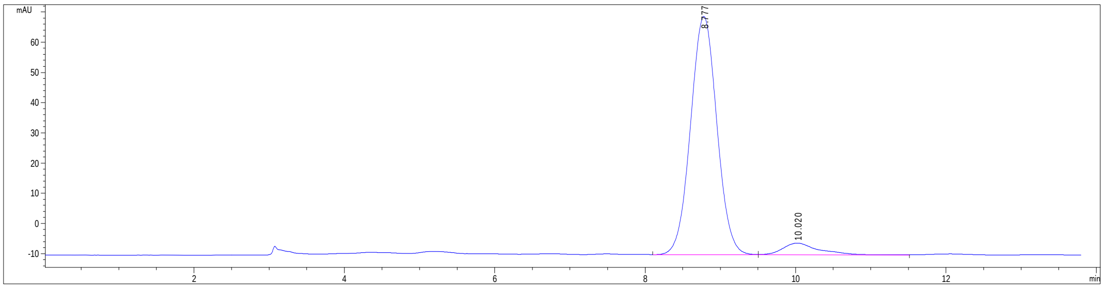

Entry	Retention Time	Area	Height	%Area

1	8.777	1914.3	78.8	93.771
2	10.02	127.2	3.9	6.229

Enantiomerically enriched **3v**

Supplementary Figure 103. HPLC spectra for compound **3v**

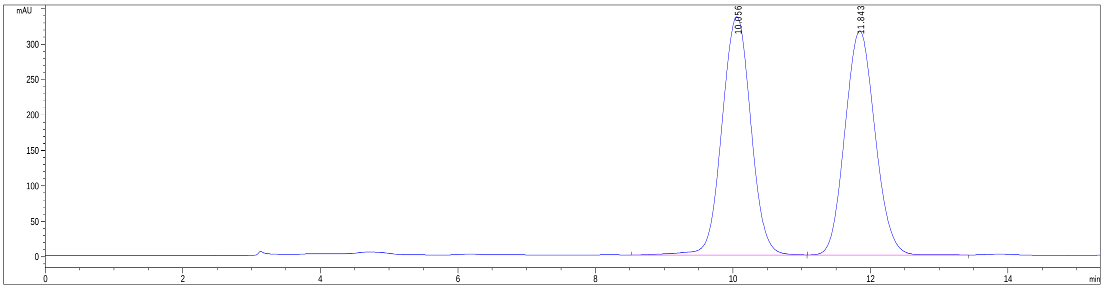

Entry	Retention Time	Area	Height	%Area
1	10.056	9812.2	336	50.517
2	11.843	9611.5	316.3	49.483

Racemic **3w**

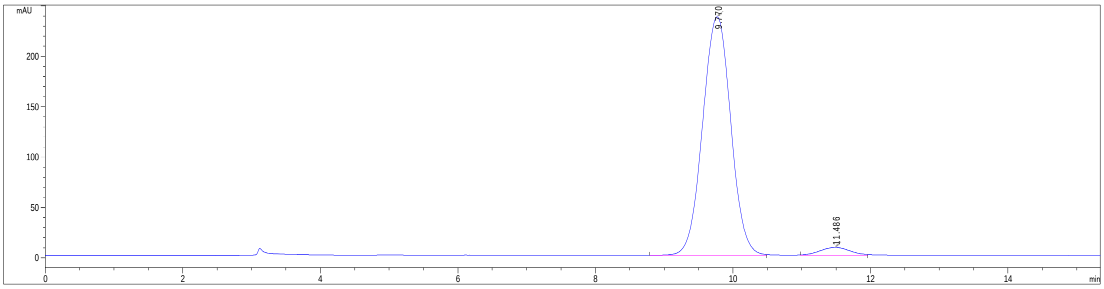

Entry	Retention Time	Area	Height	%Area
1	9.77	6711.3	236.7	96.629
2	11.486	234.1	7.8	3.371

Enantiomerically enriched **3w**

Supplementary Figure 104. HPLC spectra for compound **3w**

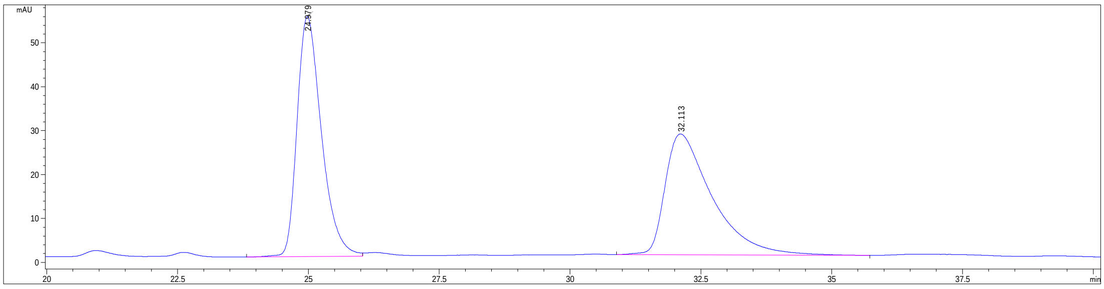

Entry	Retention Time	Area	Height	%Area
1	24.979	1768	54.6	50.690
2	32.113	1719.8	27.5	49.310

Racemic **3x**

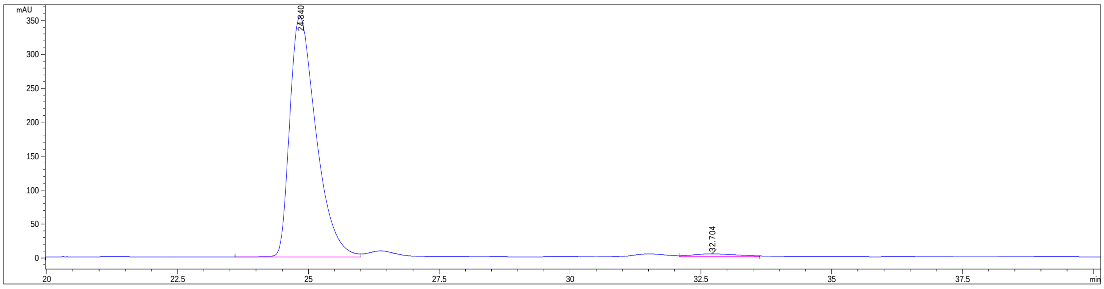

Entry	Retention Time	Area	Height	%Area
1	24.84	12359.2	354.2	97.932
2	32.704	261	4.2	2.068

Enantiomerically enriched **3x**

Supplementary Figure 105. HPLC spectra for compound **3x**

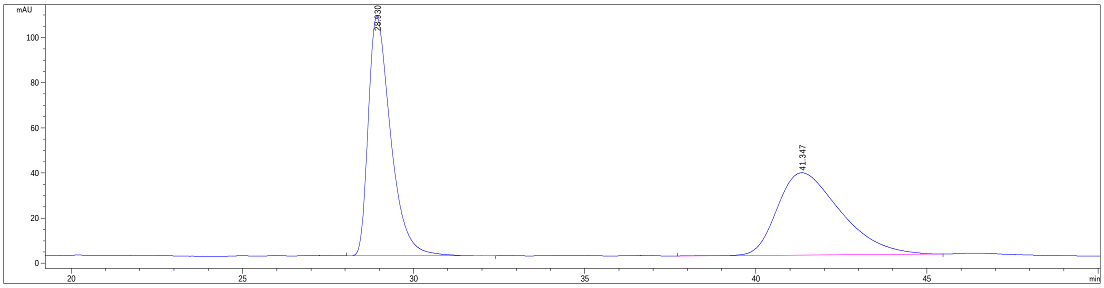

Entry	Retention Time	Area	Height	%Area
1	28.93	4835.3	106	50.762
2	41.347	4690.2	36.5	49.238

Racemic **3y**

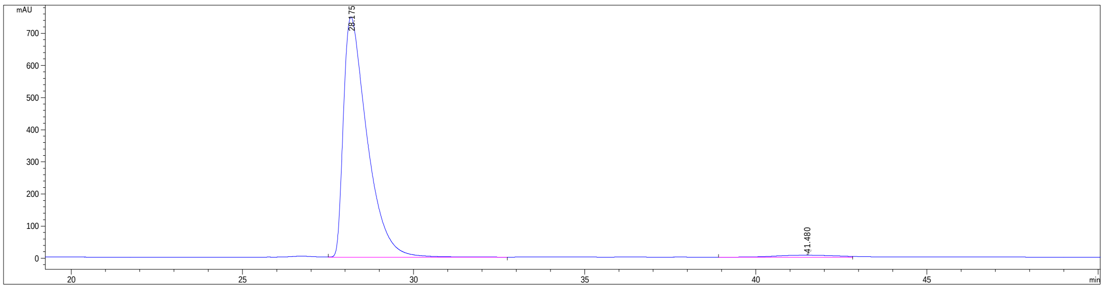

Entry	Retention Time	Area	Height	%Area
1	28.175	35815	747.4	98.097
2	41.48	694.9	5.9	1.903

Enantiomerically enriched **3y**

Supplementary Figure 106. HPLC spectra for compound **3y**

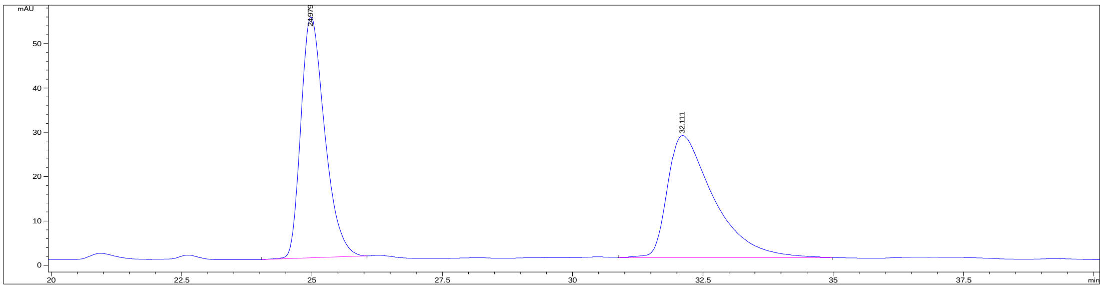

Entry	Retention Time	Area	Height	%Area

1	24.979	1724.9	54.3	50.363
2	32.111	1700.1	27.5	49.637

Racemic **3z**

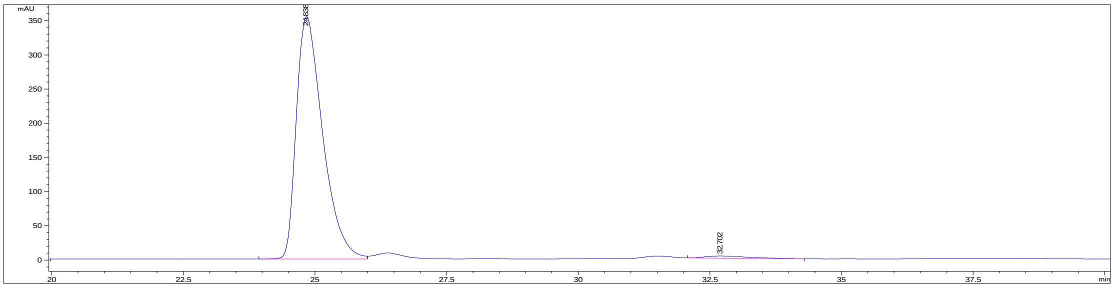

Entry	Retention Time	Area	Height	%Area
1	24.838	12350.8	354.2	98.617
2	32.702	173.3	3.1	1.383

Enantiomerically enriched **3z**

Supplementary Figure 107. HPLC spectra for compound **3z**

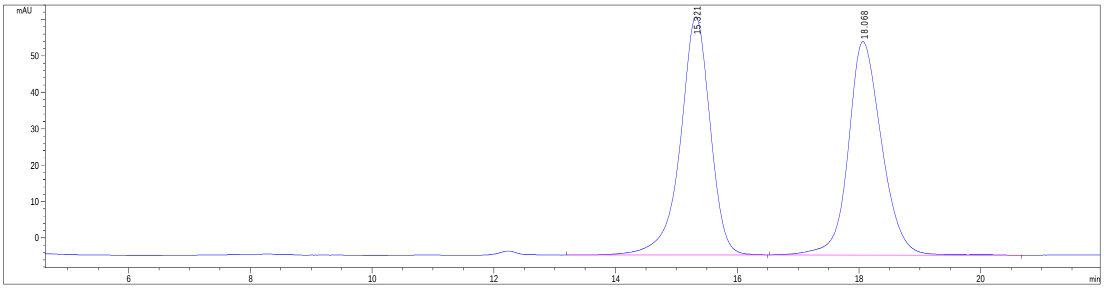

Entry	Retention Time	Area	Height	%Area
1	15.321	2228.2	65.4	50.002
2	18.068	2228	58.6	49.998

Racemic **6a**

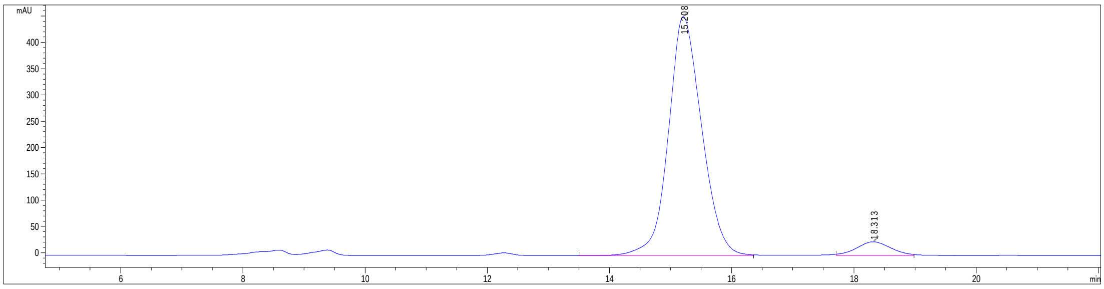

Entry	Retention Time	Area	Height	%Area
1	15.208	17098.8	453.9	94.830
2	18.313	932.2	25	5.170

Enantiomerically enriched **6a**

Supplementary Figure 108. HPLC spectra for compound **6a**

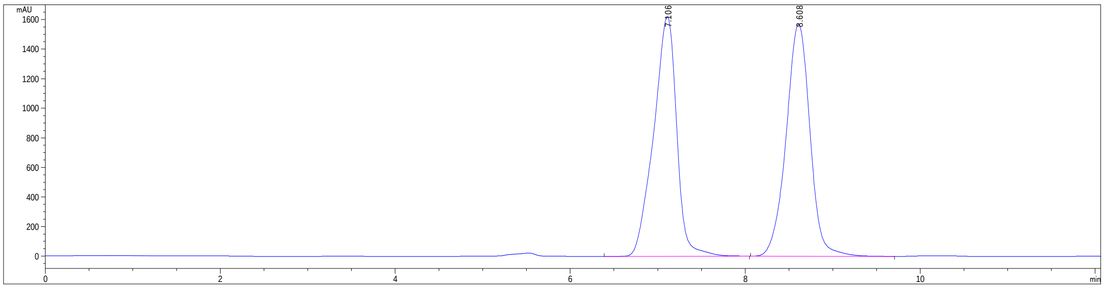

Entry	Retention Time	Area	Height	%Area
1	7.106	29637.6	1620.5	49.900
2	8.608	29756.3	1566.5	50.100

Racemic **6b**

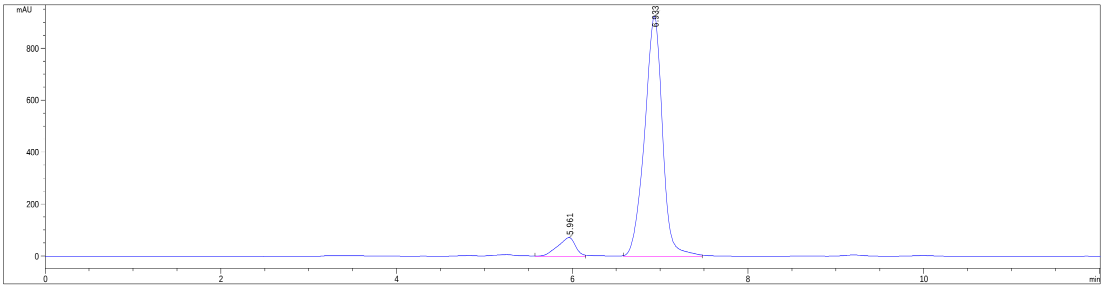

Entry	Retention Time	Area	Height	%Area
1	5.961	1008.7	72.4	7.165
2	6.933	13068.8	925.2	92.835

Enantiomerically enriched **6b**

Supplementary Figure 109. HPLC spectra for compound **6b**

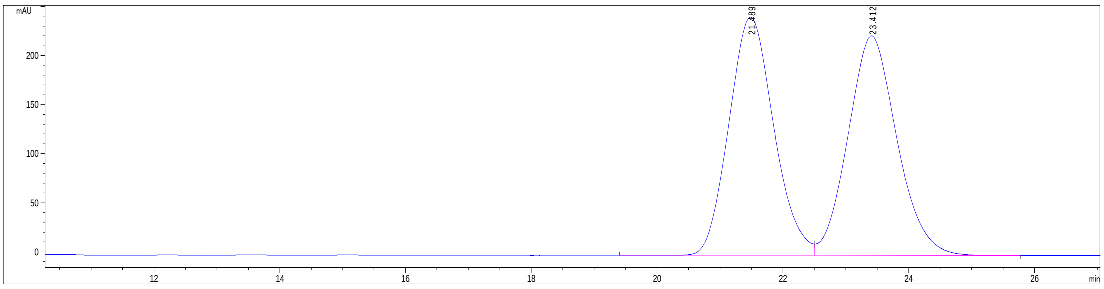

Entry	Retention Time	Area	Height	%Area
1	21.489	12019.9	242.6	49.923
2	23.412	12057.1	223.6	50.077

Racemic **6c**

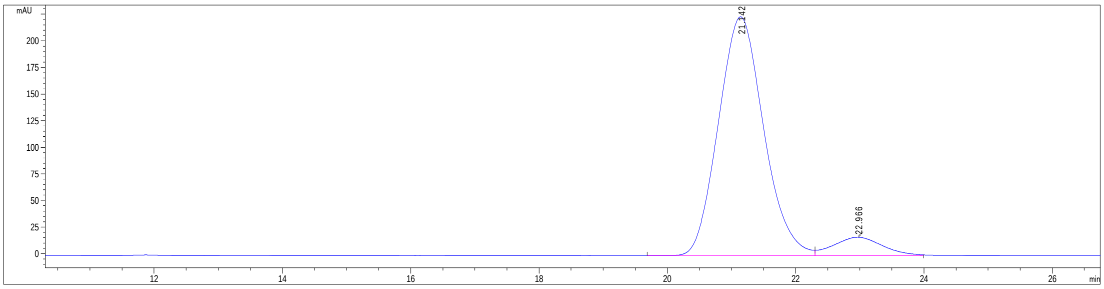

Entry	Retention Time	Area	Height	%Area
1	21.142	10906.8	224.1	92.829

2	22.966	842.5	16.8	7.171

Enantiomerically enriched **6c**

Supplementary Figure 110. HPLC spectra for compound **6c**

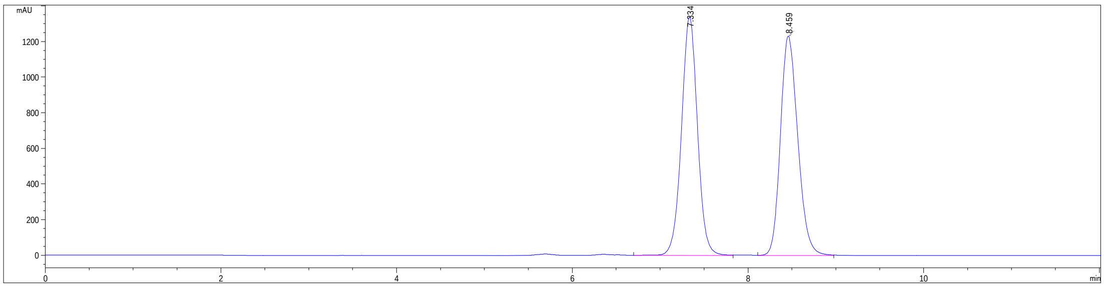

Entry	Retention Time	Area	Height	%Area
1	7.334	16965.3	1339.8	50.013
2	8.459	16956.8	1232.5	49.987

Racemic **6d**

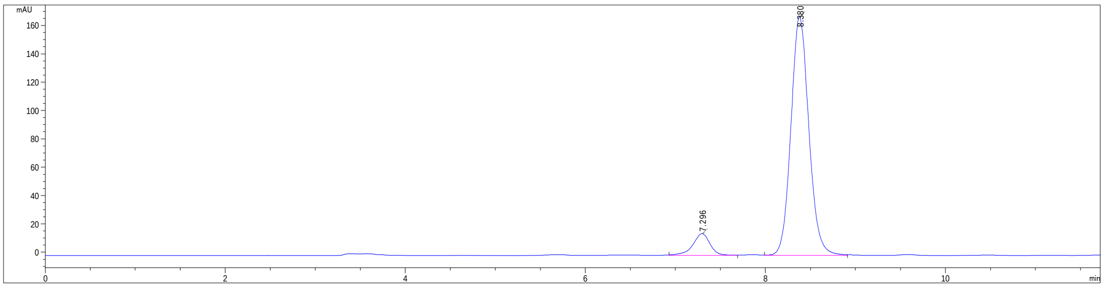

Entry	Retention Time	Area	Height	%Area
1	7.296	176.8	14.6	7.054
2	8.38	2329.5	168.4	92.946

Enantiomerically enriched **6d**

Supplementary Figure 111. HPLC spectra for compound **6d**

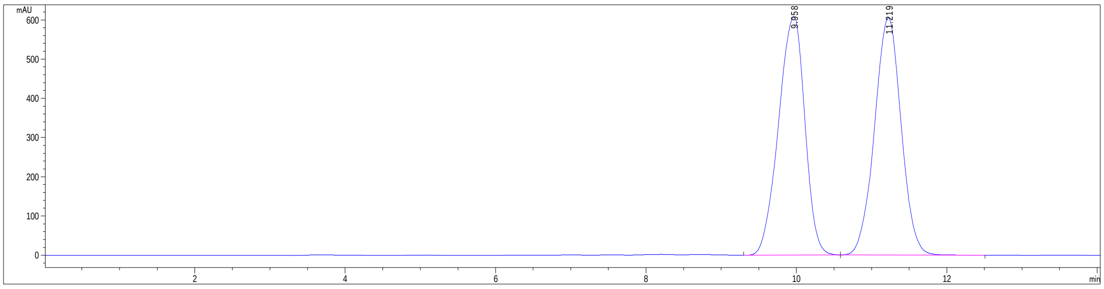

Entry	Retention Time	Area	Height	%Area
1	9.958	14774.5	608.4	49.852
2	11.219	14862	604.8	50.148

Racemic **6e**

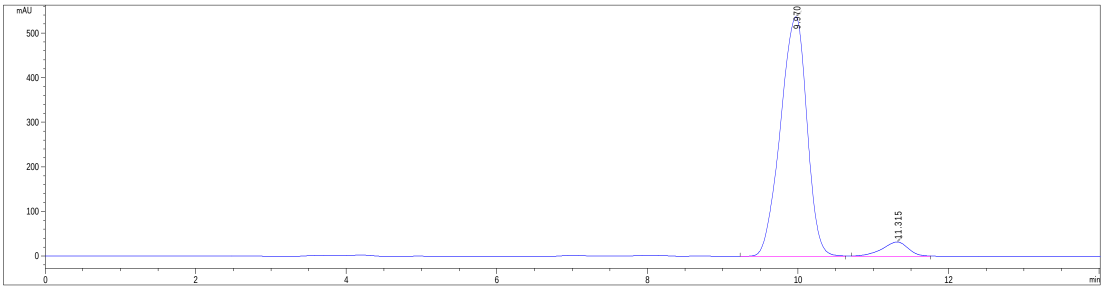

Entry	Retention Time	Area	Height	%Area
1	9.97	13070.8	536.9	94.924
2	11.315	698.9	30.9	5.076

Enantiomerically enriched **6e**

Supplementary Figure 112. HPLC spectra for compound **6e**

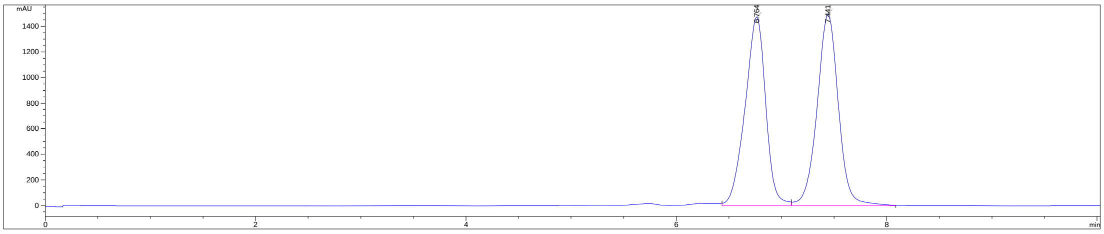

Entry	Retention Time	Area	Height	%Area
1	6.764	20172.3	1475.4	49.156
2	7.441	20864.9	1497.2	50.844

Racemic **6f**

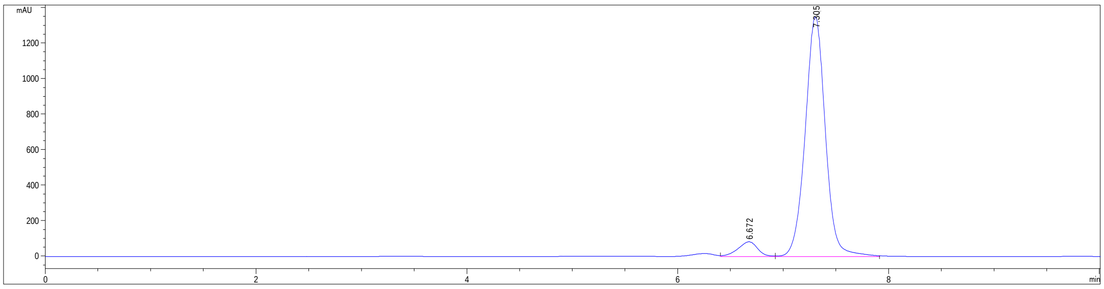

Entry	Retention Time	Area	Height	%Area
1	6.672	980	80.1	5.171
2	7.305	17972.9	1352.4	94.829

Enantiomerically enriched **6f**

Supplementary Figure 113. HPLC spectra for compound **6f**

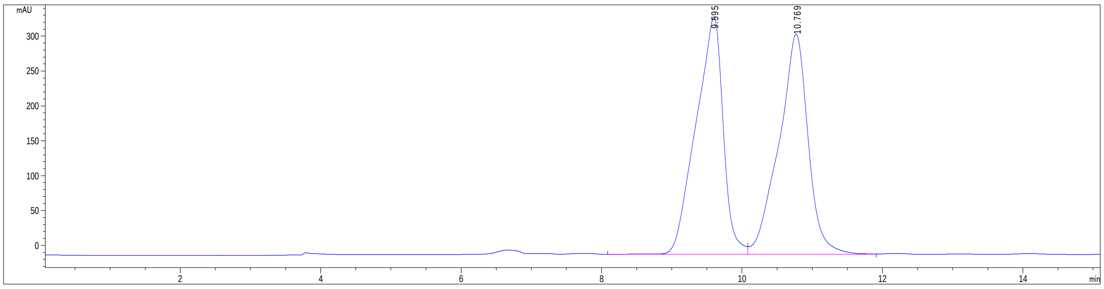

Entry	Retention Time	Area	Height	%Area
1	9.595	9201.4	340.5	50.060

2	10.769	9179.5	315.3	49.940

Racemic **6g**

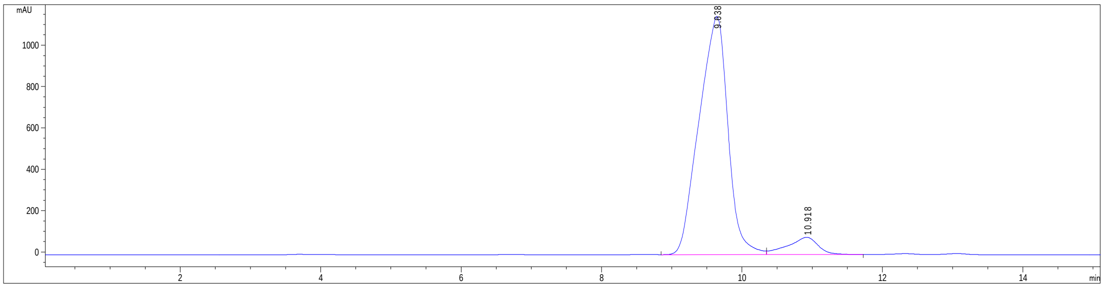

Entry	Retention Time	Area	Height	%Area
1	9.638	32854.8	1150.5	92.898
2	10.918	2511.9	83.4	7.102

Enantiomerically enriched **6g**

Supplementary Figure 114. HPLC spectra for compound **6g**

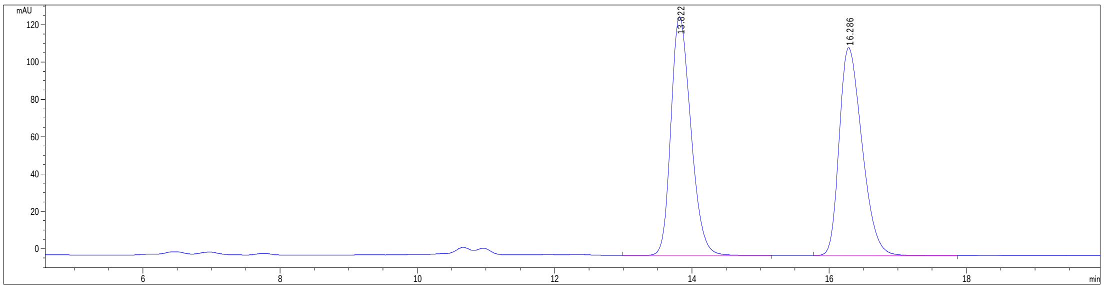

Entry	Retention Time	Area	Height	%Area
1	13.822	2580.3	127.6	50.144
2	16.286	2565.5	111.1	49.856

Racemic **6h**

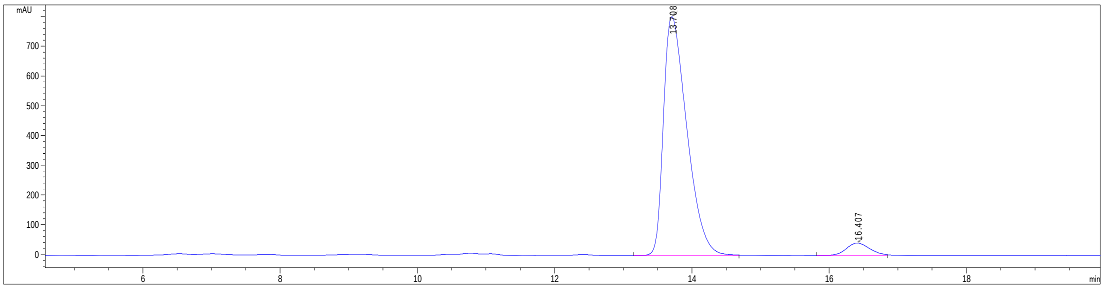

Entry	Retention Time	Area	Height	%Area
1	13.708	18986.1	803	95.029
2	16.407	993.1	41.7	4.971

Enantiomerically enriched **6h**

Supplementary Figure 115. HPLC spectra for compound **6h**

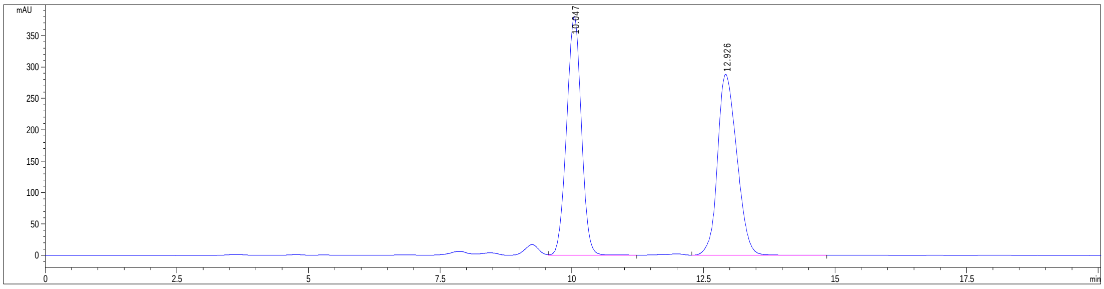

Entry	Retention Time	Area	Height	%Area
1	10.047	7379.6	379.4	50.072
2	12.926	7358.3	288.1	49.928

Racemic **6i**

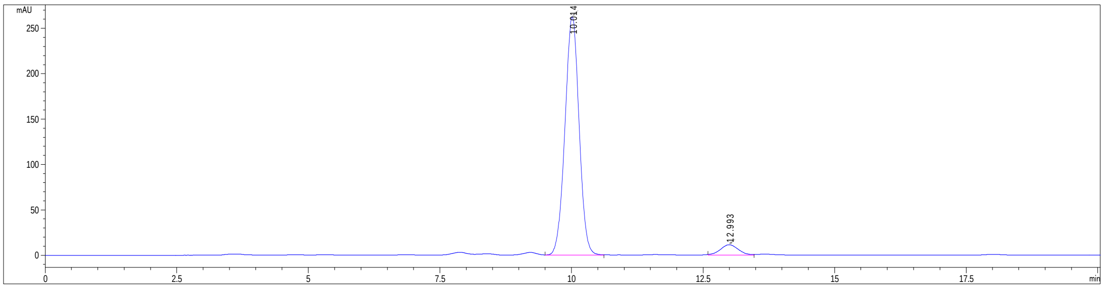

Entry	Retention Time	Area	Height	%Area
1	10.014	4898.1	262.9	94.786
2	12.993	269.5	11.4	5.214

Enantiomerically enriched **6i**

Supplementary Figure 116. HPLC spectra for compound **6i**

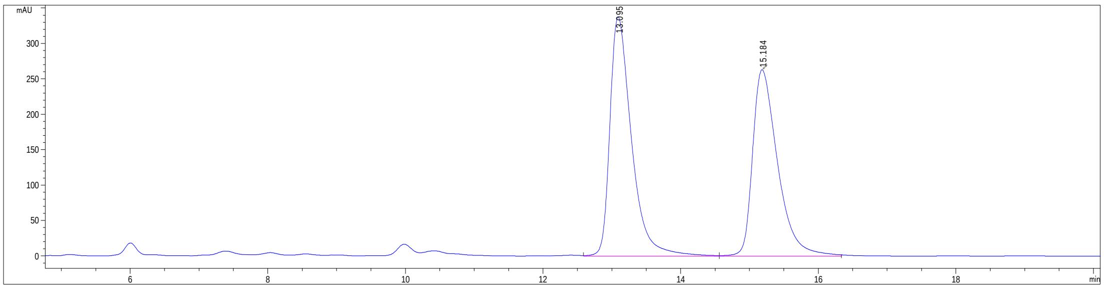

Entry	Retention Time	Area	Height	%Area
1	13.095	6765	331.9	50.360
2	15.181	6668.2	263.2	49.640

Racemic **6j**

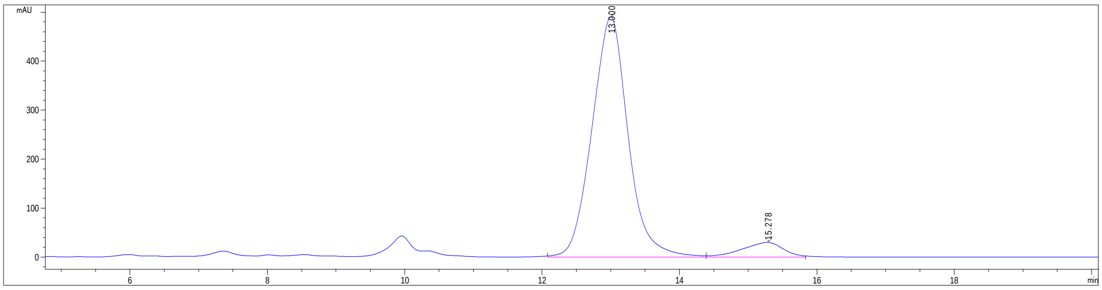

Entry	Retention Time	Area	Height	%Area
1	13	17905	490.6	93.383

2	15.278	1268.7	29.9	6.617

Enantiomerically enriched **6j**

Supplementary Figure 117. HPLC spectra for compound **6j**

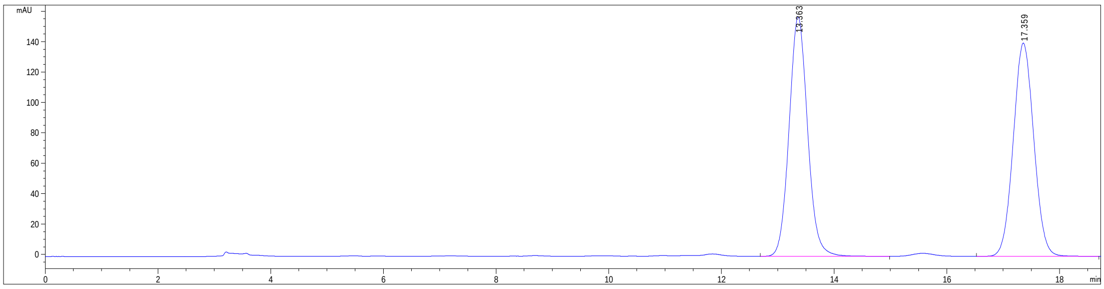

Entry	Retention Time	Area	Height	%Area
1	13.363	3597.7	157.4	50.326
2	17.359	3551.1	140.2	49.674

Racemic **6k**

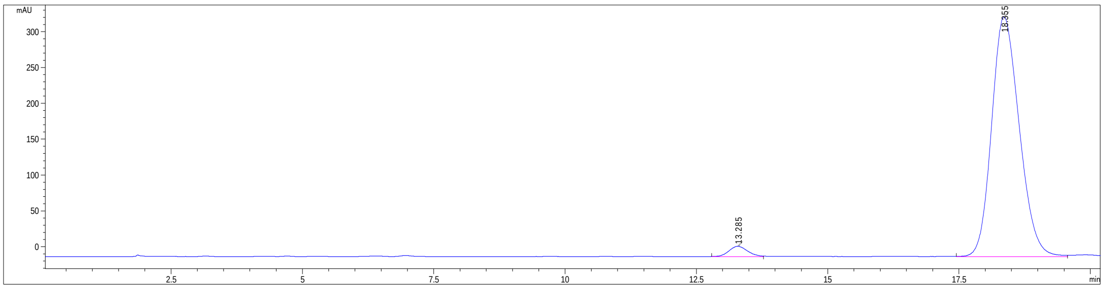

Entry	Retention Time	Area	Height	%Area
1	13.285	352.9	14.3	2.807
2	18.355	12219.2	334.8	97.193

Enantiomerically enriched **6k**

Supplementary Figure 118. HPLC spectra for compound **6k**

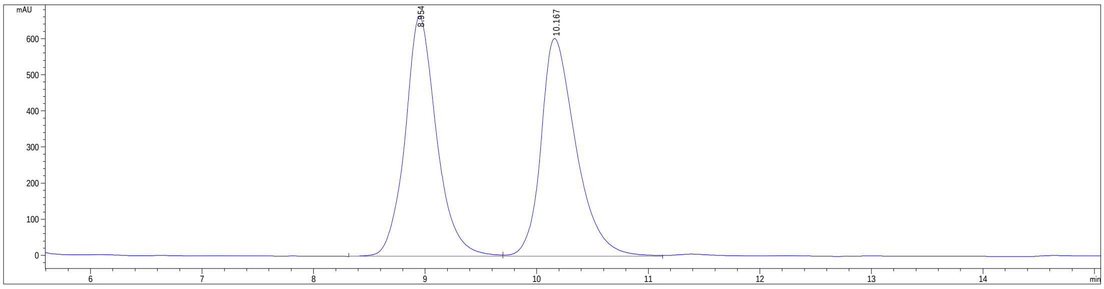

Entry	Retention Time	Area	Height	%Area
1	8.954	13045.7	662.1	49.860
2	10.167	13118.8	602.8	50.140

Racemic **6l**

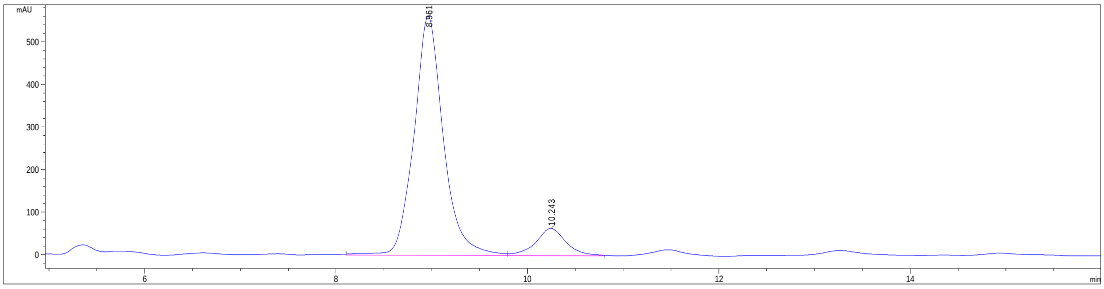

Entry	Retention Time	Area	Height	%Area
1	8.961	11959.2	561.6	89.985
2	10.243	1331	63.1	10.015

Enantiomerically enriched **6l**

Supplementary Figure 119. HPLC spectra for compound **6l**

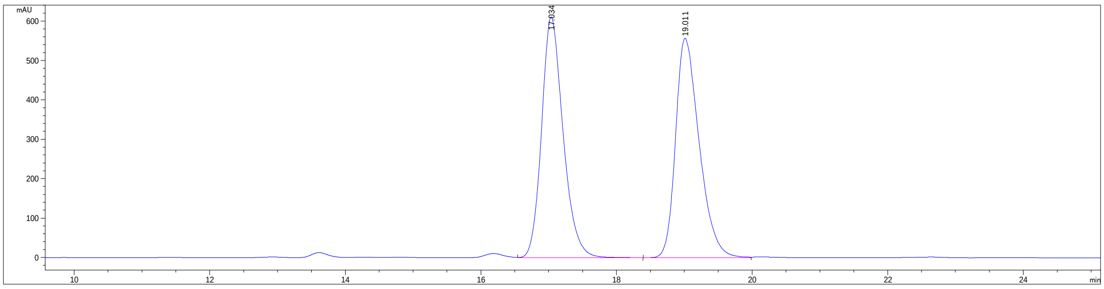

Entry	Retention Time	Area	Height	%Area
1	17.034	13481.6	611.1	50.011
2	19.011	13475.8	557	49.989

Racemic **6m**

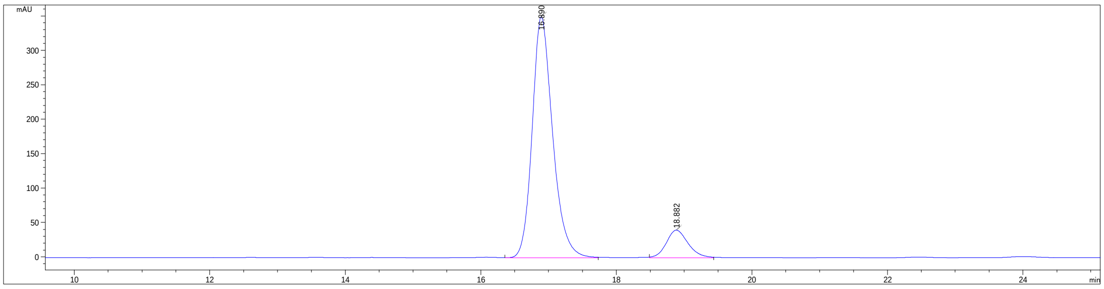

Entry	Retention Time	Area	Height	%Area
1	16.89	7339.9	350	89.117
2	18.882	896.4	40.1	10.883

Enantiomerically enriched **6m**

Supplementary Figure 120. HPLC spectra for compound **6m**

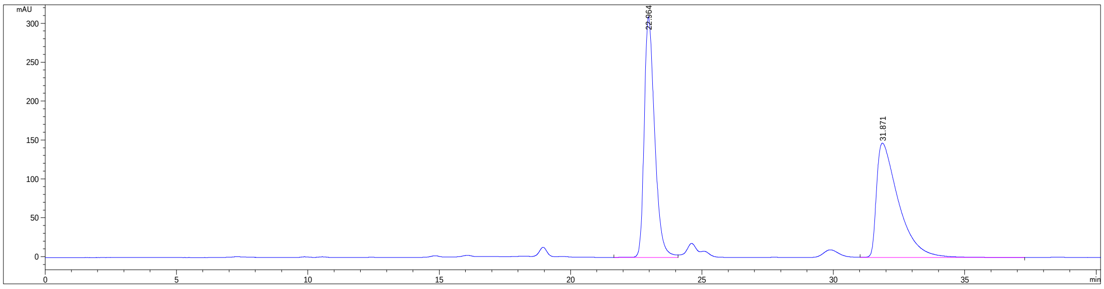

Entry	Retention Time	Area	Height	%Area

1	22.964	8582.9	309.5	50.056
2	31.871	8563.6	147.2	49.944

Racemic **8a**

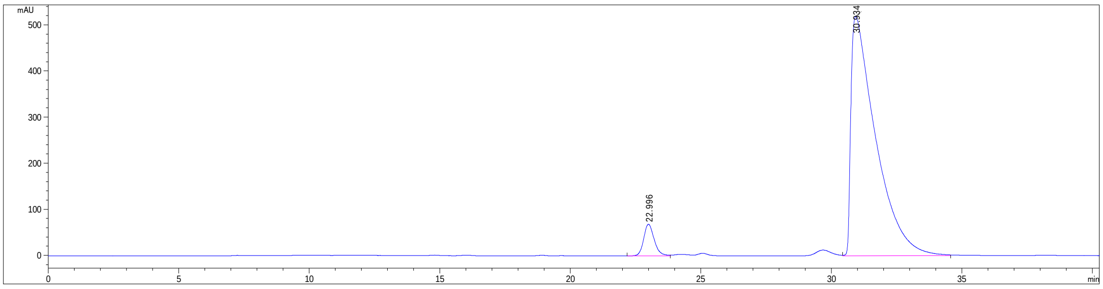

Entry	Retention Time	Area	Height	%Area
1	22.996	1885.8	68.5	5.221
2	30.934	34234.8	518.9	94.779

Enantiomerically enriched **8a**

Supplementary Figure 121. HPLC spectra for compound **8a**

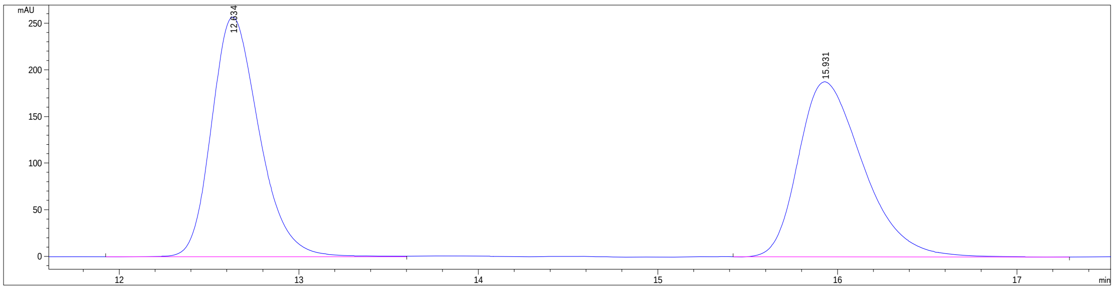

Entry	Retention Time	Area	Height	%Area
1	12.634	4731.4	256.9	50.163
2	15.931	4700.5	187.5	49.837

Racemic **8b**

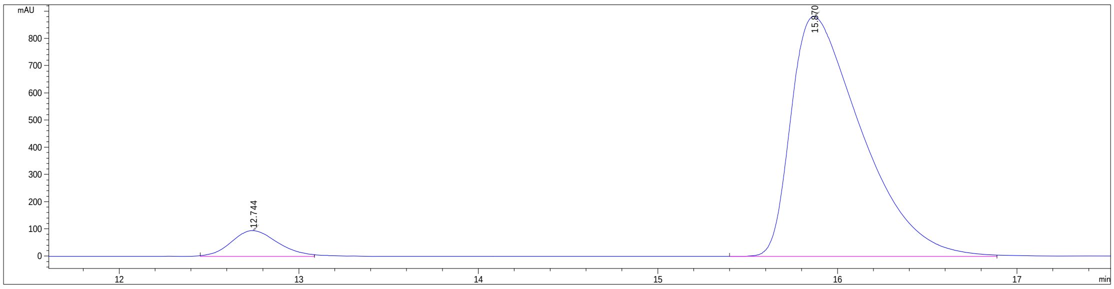

Entry	Retention Time	Area	Height	%Area
1	12.744	1677.6	94.2	6.511
2	15.87	24086.7	880.8	93.489

Enantiomerically enriched **8b**

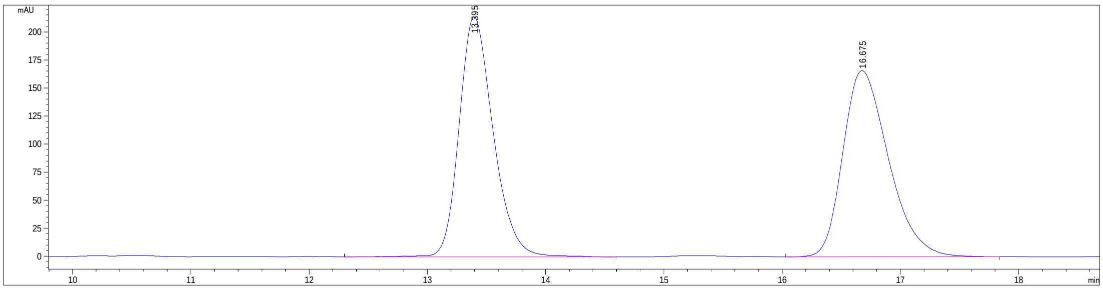

Entry	Retention Time	Area	Height	%Area
1	13.395	4296.4	213.9	49.142
2	16.675	4446.3	166	50.858

Racemic **8b**

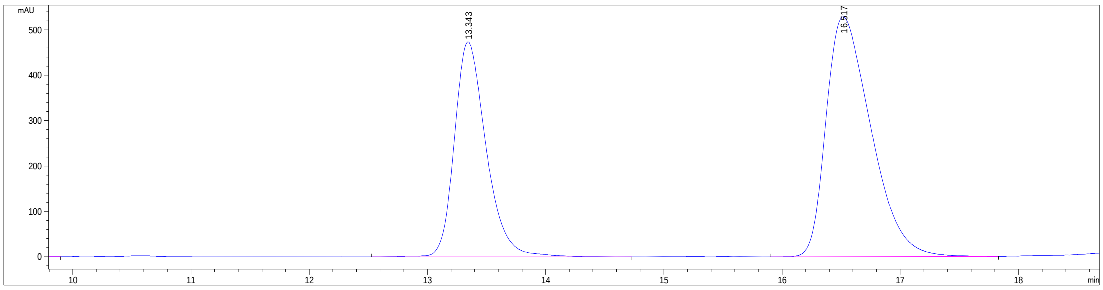

Entry	Retention Time	Area	Height	%Area
1	13.343	8871.8	474.4	38.878
2	16.517	13947.7	529.1	61.122

Enantiomerically enriched **8b** (From **7a** with **17**, see Fig. 6C)

Supplementary Figure 122. HPLC spectra for compound **8b**

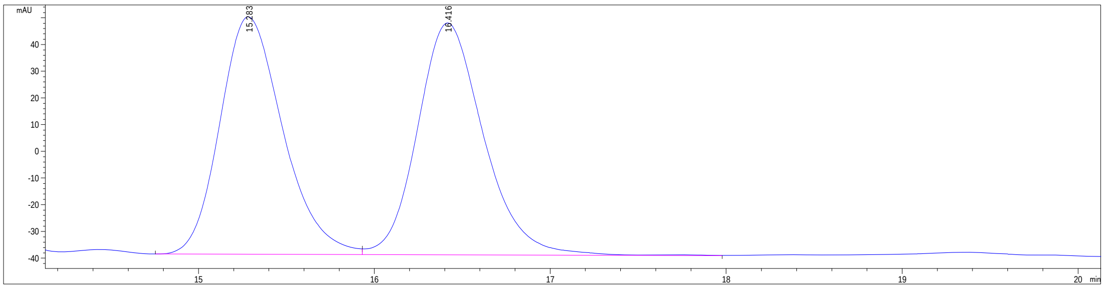

Entry	Retention Time	Area	Height	%Area
1	15.283	2214.5	88.9	49.475
2	16.416	2261.5	86.8	50.525

Racemic **8c**

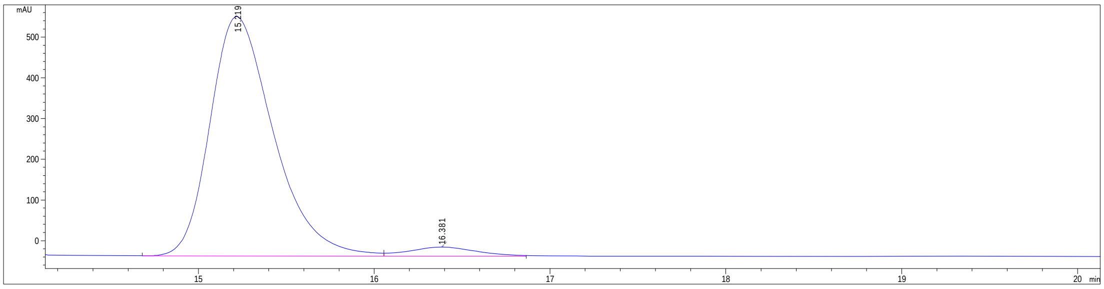

Entry	Retention Time	Area	Height	%Area
1	15.219	14749.1	586.8	96.163

2	16.381	588.5	22.2	3.837

Enantiomerically enriched **8c**

Supplementary Figure 123. HPLC spectra for compound **8c**

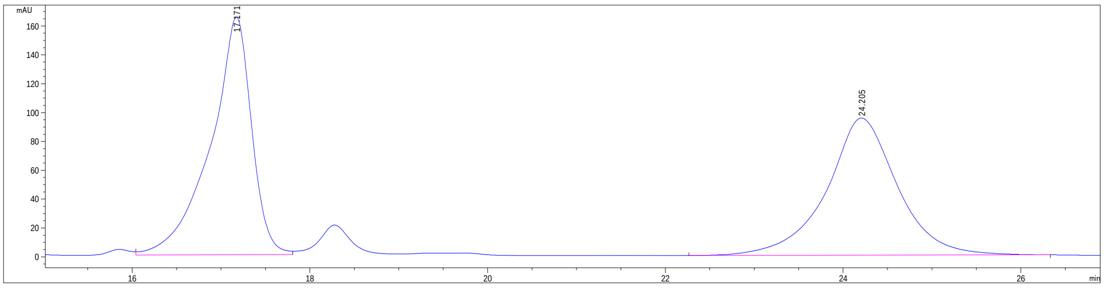

Entry	Retention Time	Area	Height	%Area
1	17.171	5303.9	164.9	50.063
2	24.205	5290.6	95.1	49.937

Racemic **8d**

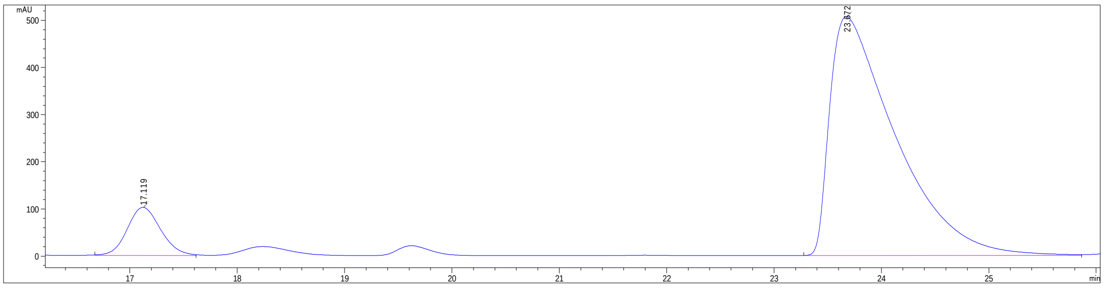

Entry	Retention Time	Area	Height	%Area
1	17.119	2184.9	102.1	9.186
2	23.672	21600.7	506.8	90.814

Enantiomerically enriched **8d**

Supplementary Figure 124. HPLC spectra for compound **8d**

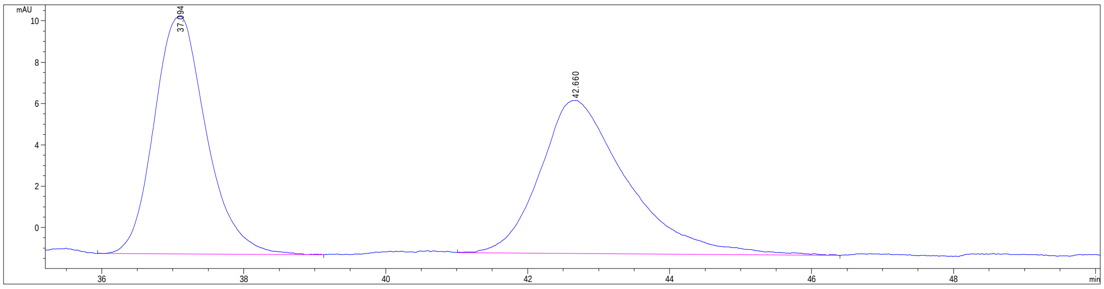

Entry	Retention Time	Area	Height	%Area
1	37.094	586.5	11.5	48.198
2	42.66	630.3	7.4	51.802

Racemic **8e**

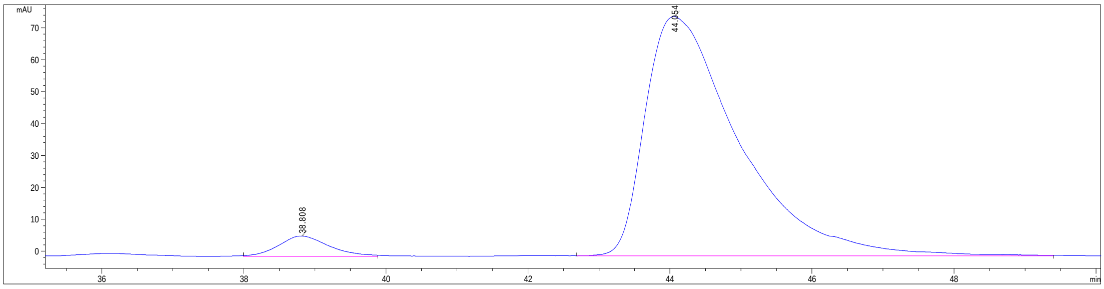

Entry	Retention Time	Area	Height	%Area
1	38.808	298.8	6.2	4.142
2	44.054	6915.8	75	95.858

Enantiomerically enriched **8e**

Supplementary Figure 125. HPLC spectra for compound **8e**

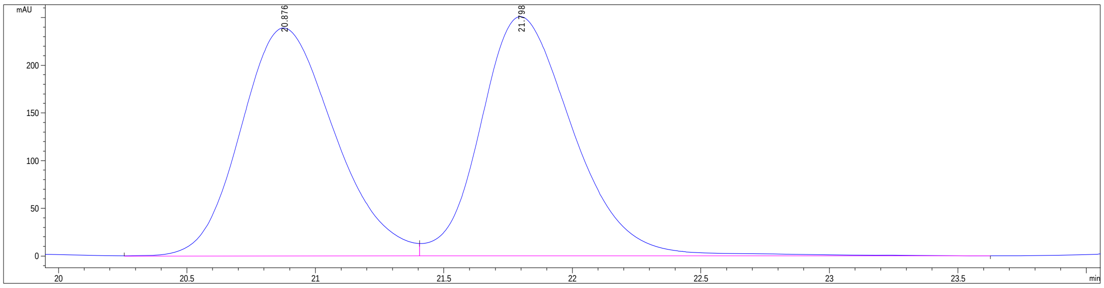

Entry	Retention Time	Area	Height	%Area
1	20.876	6062.1	239	49.123
2	21.798	6278.4	251	50.877

Racemic **8f**

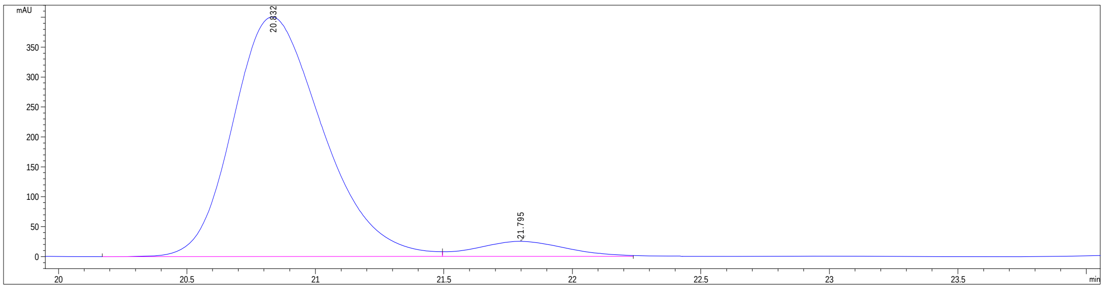

Entry	Retention Time	Area	Height	%Area
1	20.832	9656.8	400.7	94.896
2	21.795	519.4	22.6	5.104

Enantiomerically enriched **8f**

Supplementary Figure 126. HPLC spectra for compound **8f**

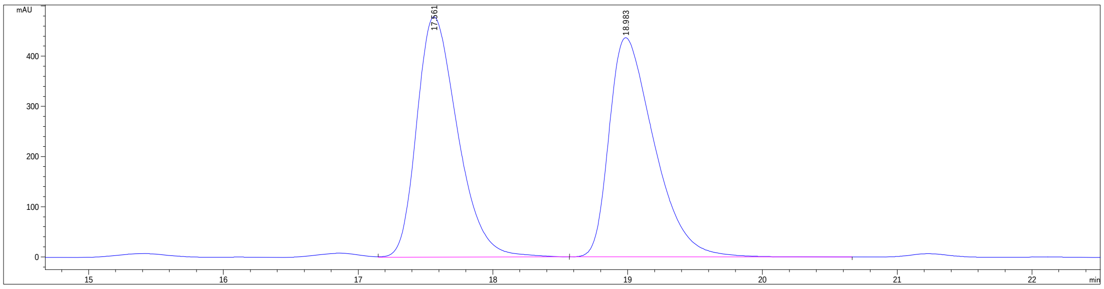

Entry	Retention Time	Area	Height	%Area

1	17.561	10082.9	478.7	49.888
2	18.983	10128.2	436.7	50.112

Racemic **8g**

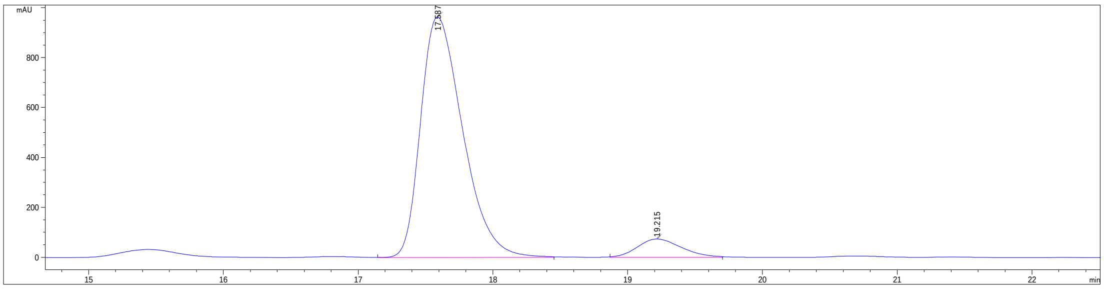

Entry	Retention Time	Area	Height	%Area
1	17.587	20912.6	964	92.520
2	19.215	1690.6	73.8	7.480

Enantiomerically enriched **8g**

Supplementary Figure 127. HPLC spectra for compound **8g**

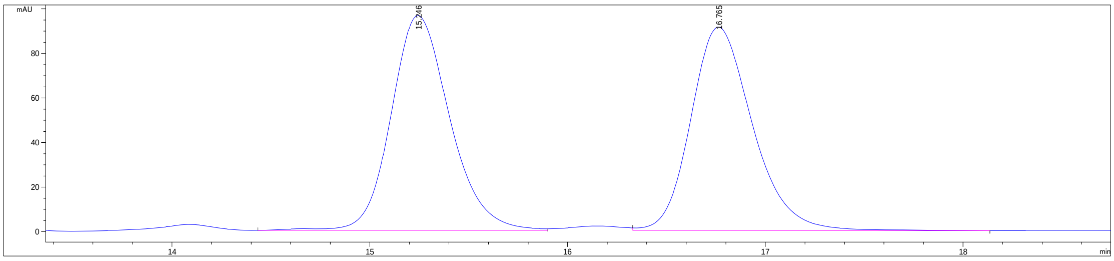

Entry	Retention Time	Area	Height	%Area
1	15.246	1996.1	96.3	50.118
2	16.765	1986.7	91.3	49.882

Racemic **8h**

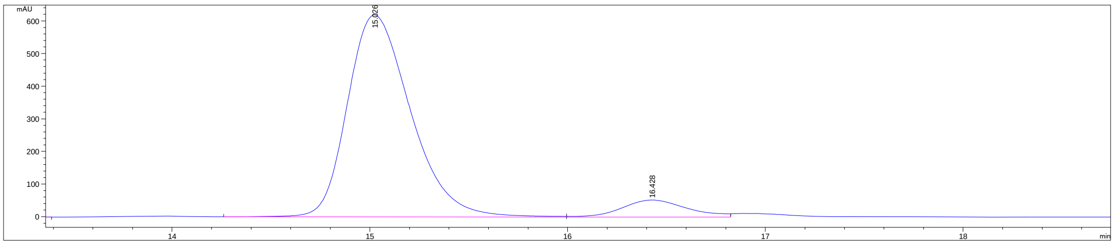

Entry	Retention Time	Area	Height	%Area
1	15.026	13746	617.7	92.942
2	16.428	1043.9	49.3	7.058

Enantiomerically enriched **8h**

Supplementary Figure 128. HPLC spectra for compound **8h**

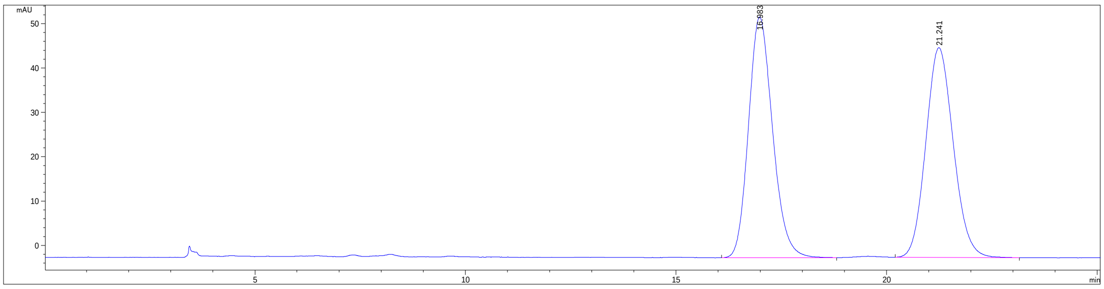

Entry	Retention Time	Area	Height	%Area
1	16.983	2158.4	54.3	50.132
2	21.241	2147	47.3	49.868

Racemic **8i**

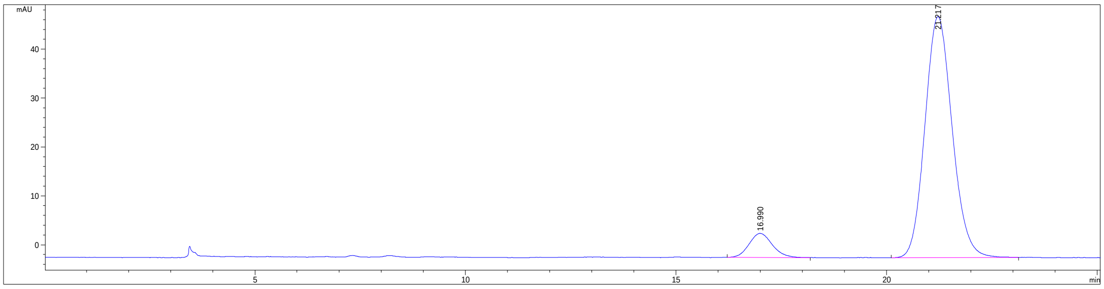

Entry	Retention Time	Area	Height	%Area
1	16.99	187.9	4.9	7.823
2	21.217	2213.4	49.3	92.177

Enantiomerically enriched **8i**

Supplementary Figure 129. HPLC spectra for compound **8i**

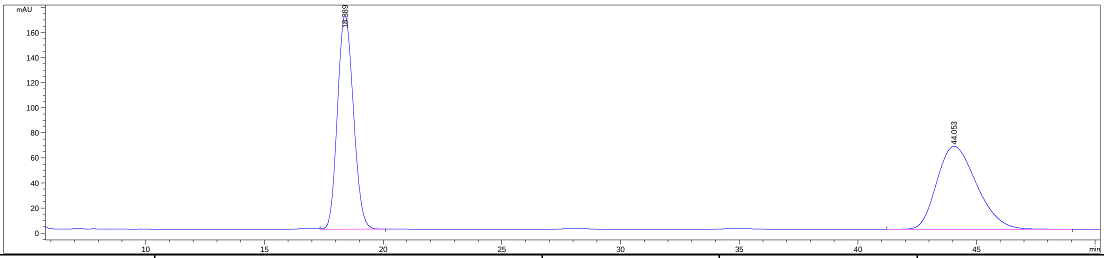

Entry	Retention Time	Area	Height	%Area
1	18.389	7930.1	170.3	50.839
2	44.053	7668.2	66	49.161

Racemic **10**

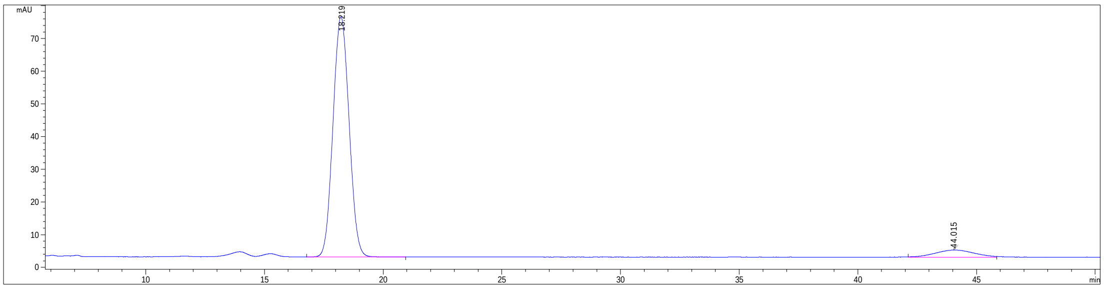

Entry	Retention Time	Area	Height	%Area
1	18.219	3450.2	73.4	93.353
2	44.015	245.7	2.2	6.647

Enantiomerically enriched **10**

Supplementary Figure 130. HPLC spectra for compound **10**

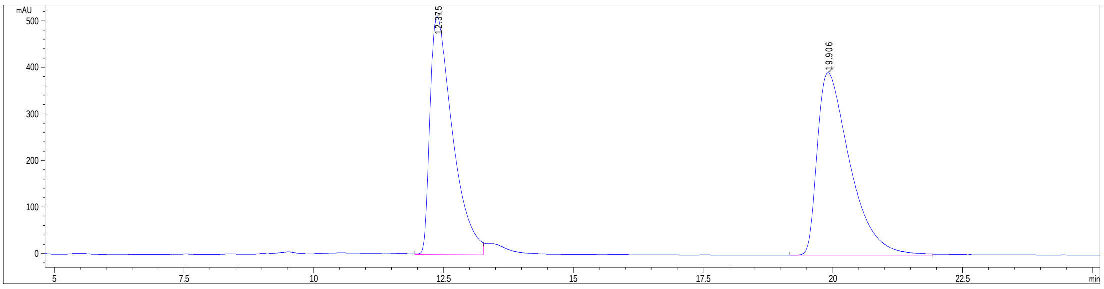

Entry	Retention Time	Area	Height	%Area
1	12.375	15529.3	511.7	49.581
2	19.906	15792	383.9	50.419

Racemic **11**

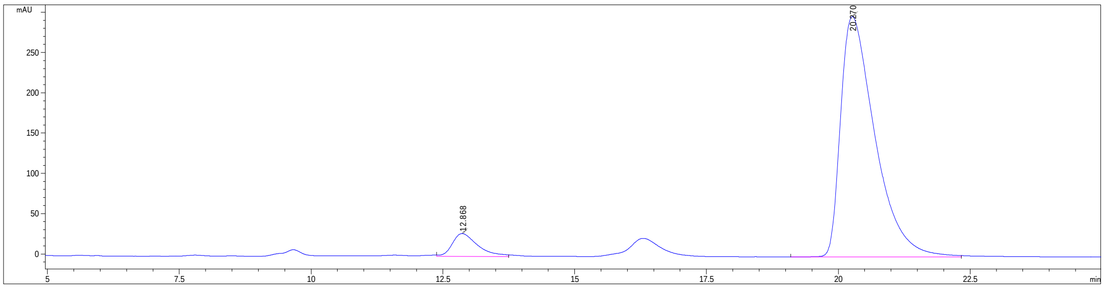

Entry	Retention Time	Area	Height	%Area
1	12.868	959.9	28.5	6.637
2	20.27	13502.8	298.3	93.363

Enantiomerically enriched **11**

Supplementary Figure 131. HPLC spectra for compound **11**

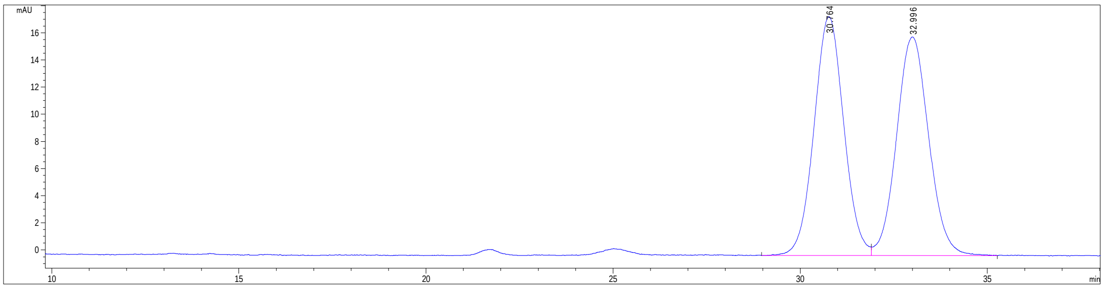

Entry	Retention Time	Area	Height	%Area
1	30.764	974.4	17.6	50.264
2	32.996	964.2	16.1	49.736

Racemic **12**

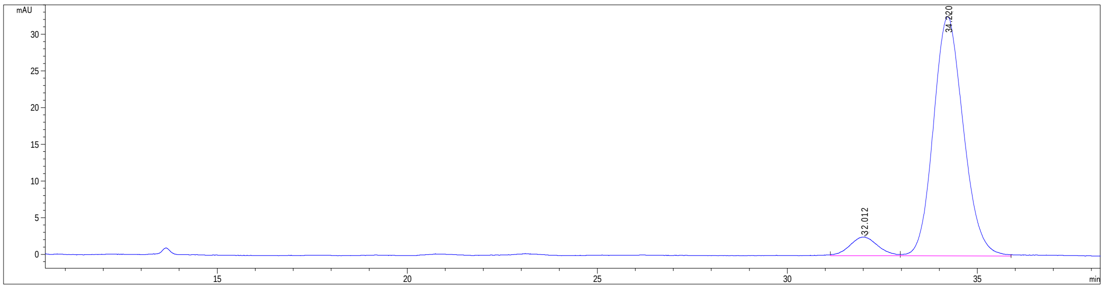

Entry	Retention Time	Area	Height	%Area
1	32.012	128.1	2.5	6.701
2	34.22	1783.4	32.6	93.299

Enantiomerically enriched **12**

Supplementary Figure 132. HPLC spectra for compound **12**

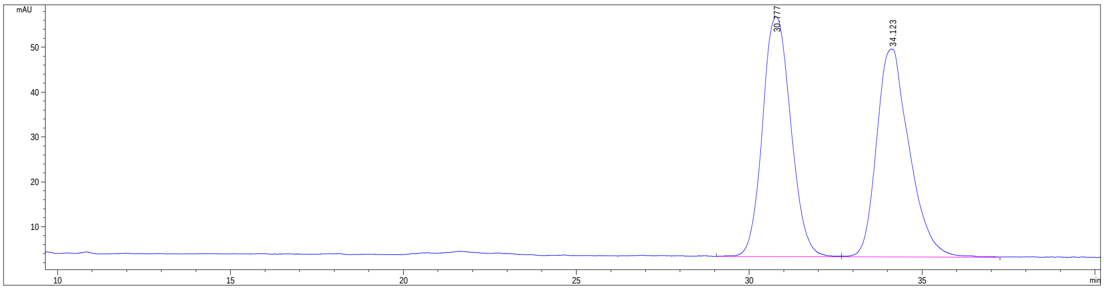

Entry	Retention Time	Area	Height	%Area
1	30.777	3043.3	53.4	50.006
2	34.123	3042.5	46.3	49.994

Racemic **25**

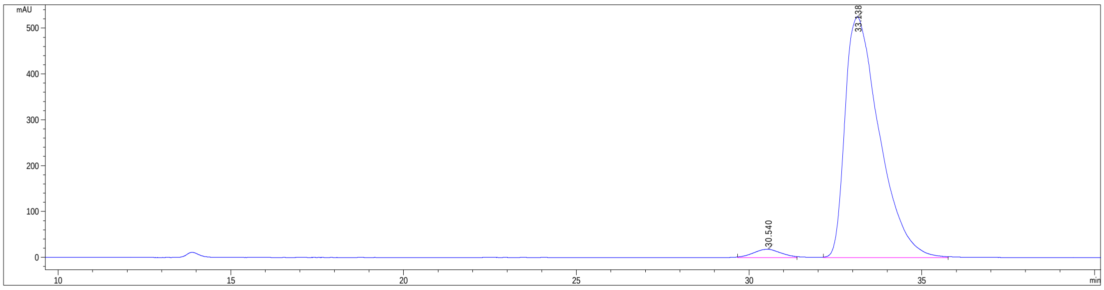

Entry	Retention Time	Area	Height	%Area
1	30.54	997.2	18.2	2.678
2	33.138	36244.7	525.5	97.322

Enantiomerically enriched **25**

Supplementary Figure 133. HPLC spectra for compound **25**

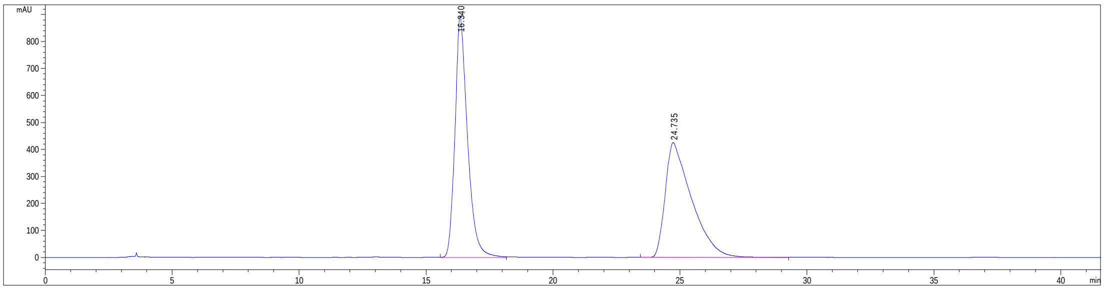

Entry	Retention Time	Area	Height	%Area
1	16.34	30689.1	892.5	50.080
2	24.735	30591.4	425.4	49.920

Racemic **26**

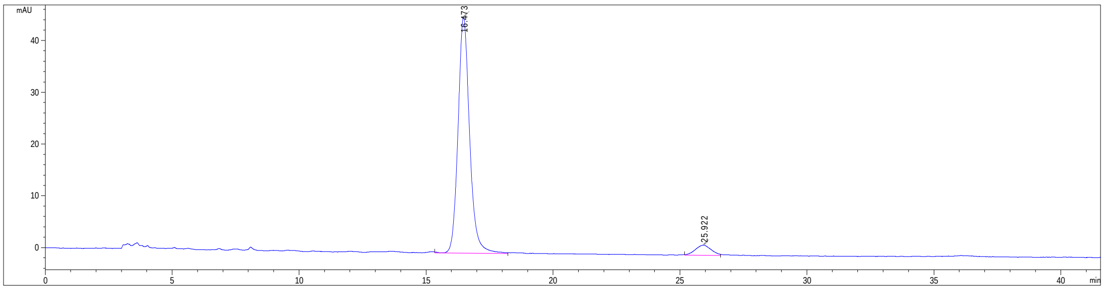

Entry	Retention Time	Area	Height	%Area
1	16.473	1450.5	45.7	94.872
2	25.922	78.4	2	5.128

Enantiomerically enriched **26**

Supplementary Figure 134. HPLC spectra for compound **26**

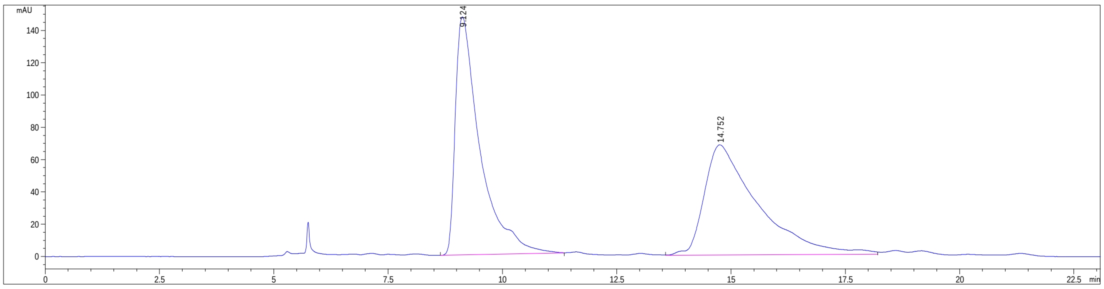

Entry	Retention Time	Area	Height	%Area
1	9.124	5539.1	147.5	49.834
2	14.752	5575.9	68.3	50.166

Supplementary Figure 135. HPLC spectra for racemic **C2**

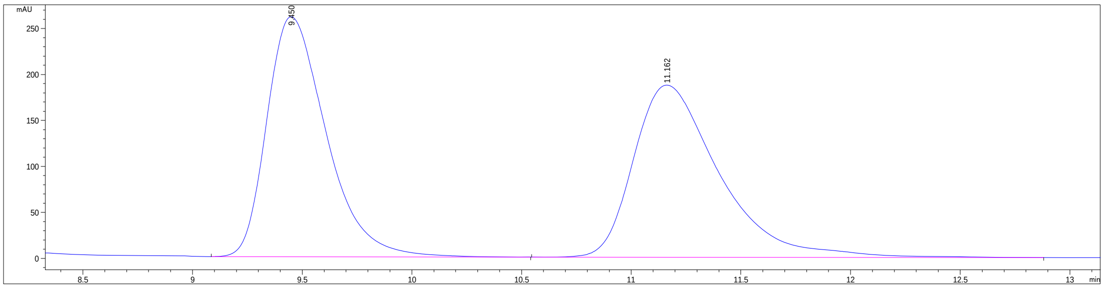

Entry	Retention Time	Area	Height	%Area
1	9.45	4913.5	261.1	49.092
2	11.162	5095.2	187.3	50.908

Supplementary Figure 136. HPLC spectra for racemic compound **3t**

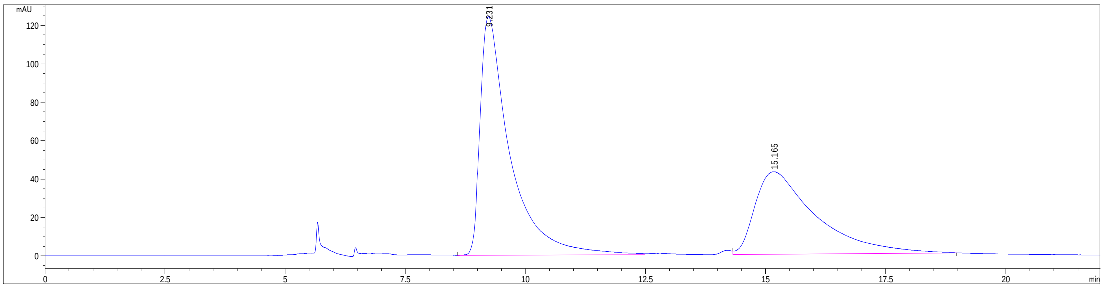

Entry	Retention Time	Area	Height	%Area

1	9.231	5359.2	124.2	58.664
2	15.165	3776.3	42.9	41.336

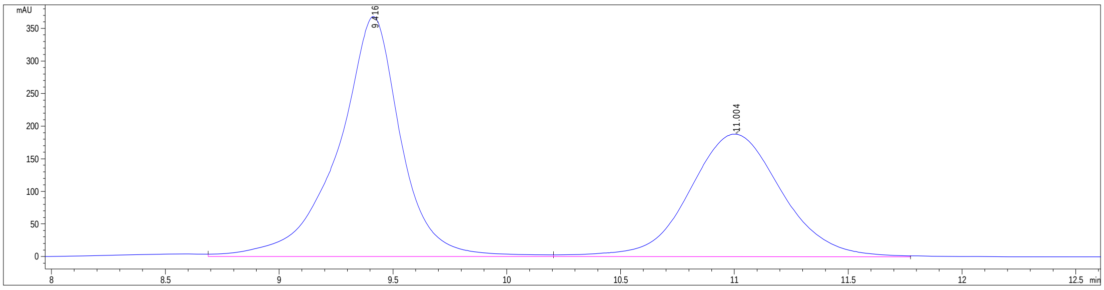

Entry	Retention Time	Area	Height	%Area
1	9.416	7077.8	368.1	57.113
2	11.004	5314.7	188	42.887

Supplementary Figure 137. HPLC spectra for the ee of **C2** and the ee of **3t**

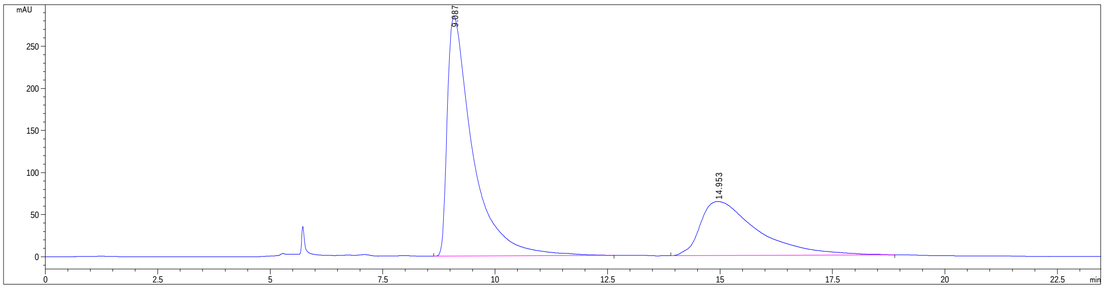

Entry	Retention Time	Area	Height	%Area
1	9.087	10687.1	284.9	65.194
2	14.953	5705.8	64.1	34.806

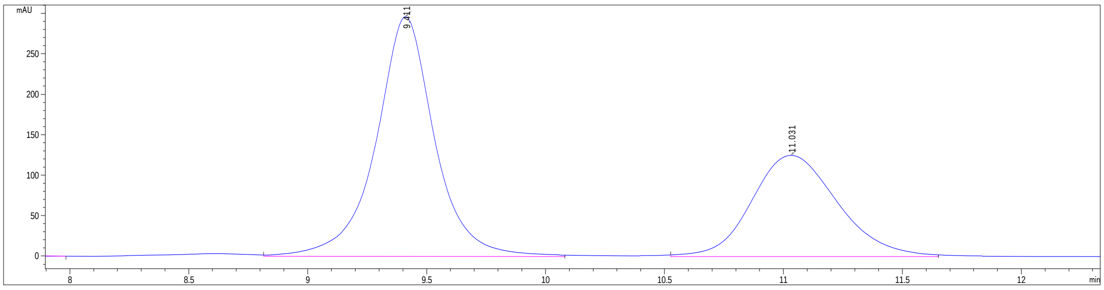

Entry	Retention Time	Area	Height	%Area
1	9.411	5090.2	296.1	62.882
2	11.031	3004.6	123.3	37.118

Supplementary Figure 138. HPLC spectra for the ee of **C2** and the ee of **3t**

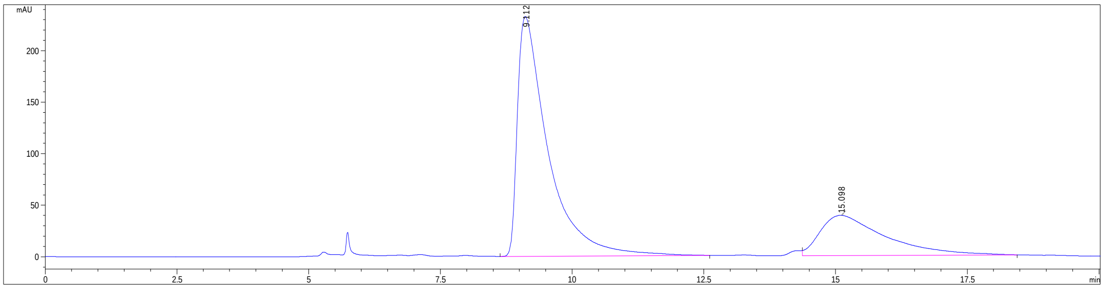

Entry	Retention Time	Area	Height	%Area
1	9.112	9105.9	232.8	73.232

2	15.098	3328.5	38.9	26.768

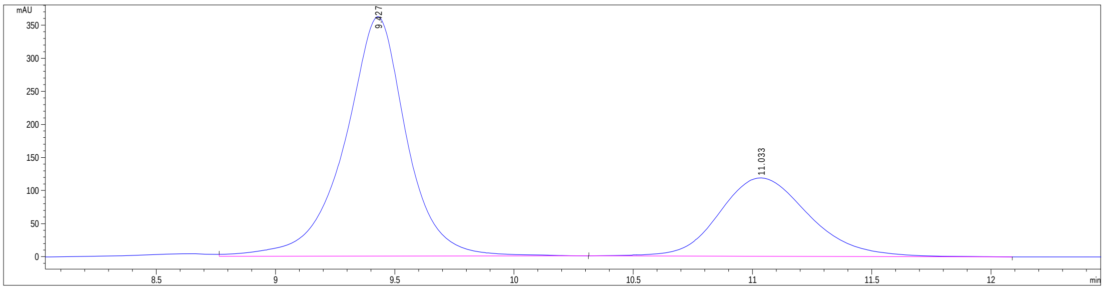

Entry	Retention Time	Area	Height	%Area
1	9.427	6535.4	361.9	67.944
2	11.033	3083.4	118.2	32.056

Supplementary Figure 139. HPLC spectra for the ee of **C2** and the ee of **3t**

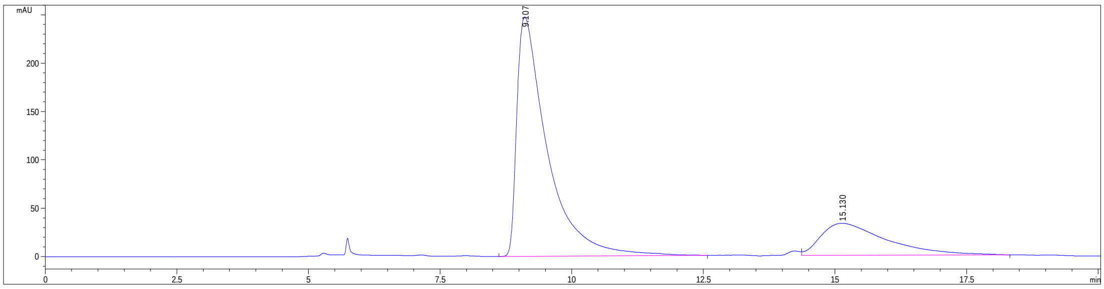

Entry	Retention Time	Area	Height	%Area
1	9.107	9966	247.6	77.713
2	15.13	2858.2	33.2	22.287

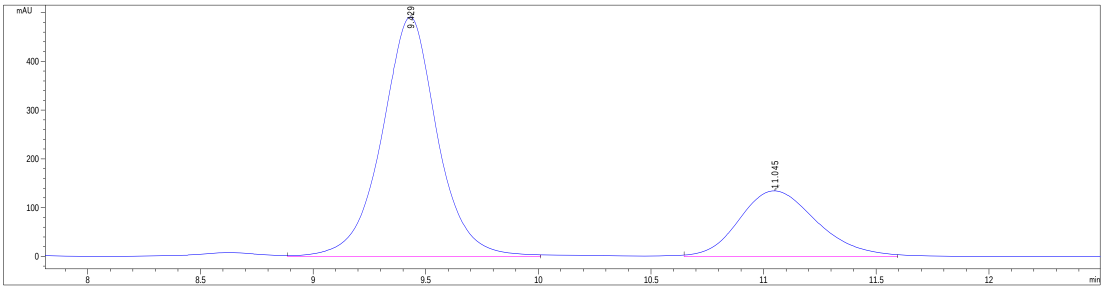

Entry	Retention Time	Area	Height	%Area
1	9.429	8430.6	491.4	72.486
2	11.045	3200	134.8	27.514

Supplementary Figure 140. HPLC spectra for the ee of **C2** and the ee of **3t**

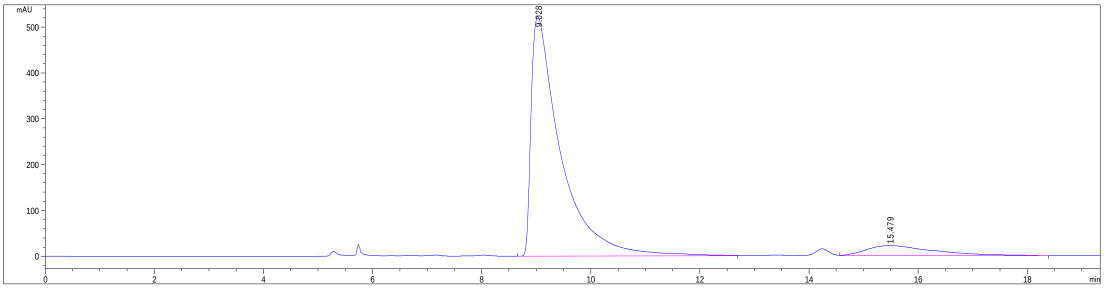

Entry	Retention Time	Area	Height	%Area
1	9.028	18358.2	522.7	90.660
2	15.479	1891.3	21.7	9.340

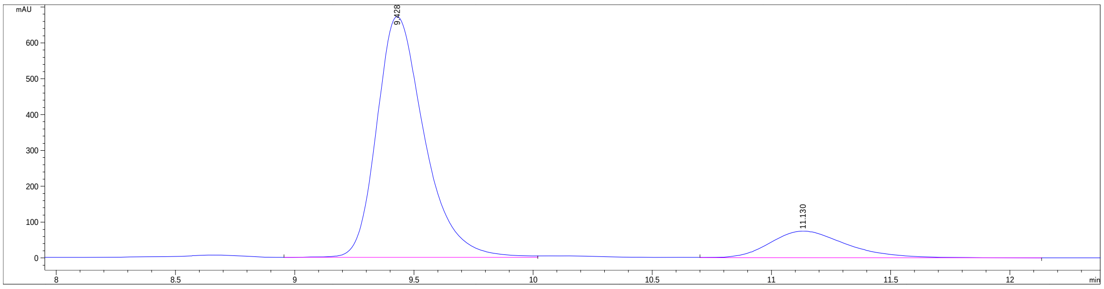

Entry	Retention Time	Area	Height	%Area
1	9.428	9366.6	672.4	85.088
2	11.13	1641.6	74	14.912

Supplementary Figure 141. HPLC spectra for the ee of **C2** and the ee of **3t**

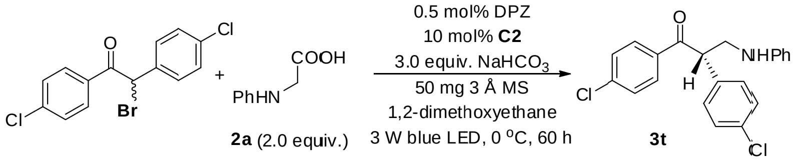

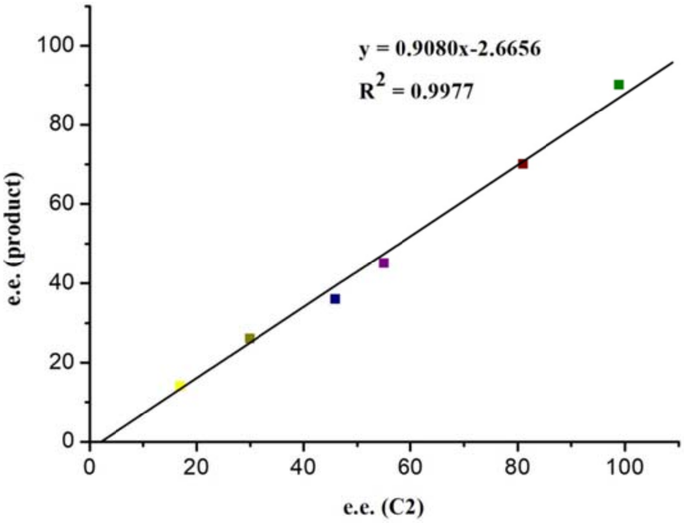

Supplementary Figure 142. Relationship between ee values of **C2** and **3t**.

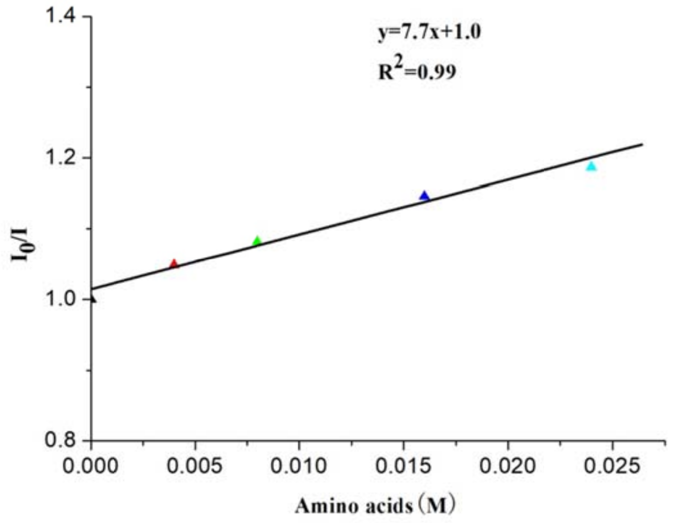

Supplementary Figure 143. Stern–Volmer quenching experiment of DPZ and **2a**.

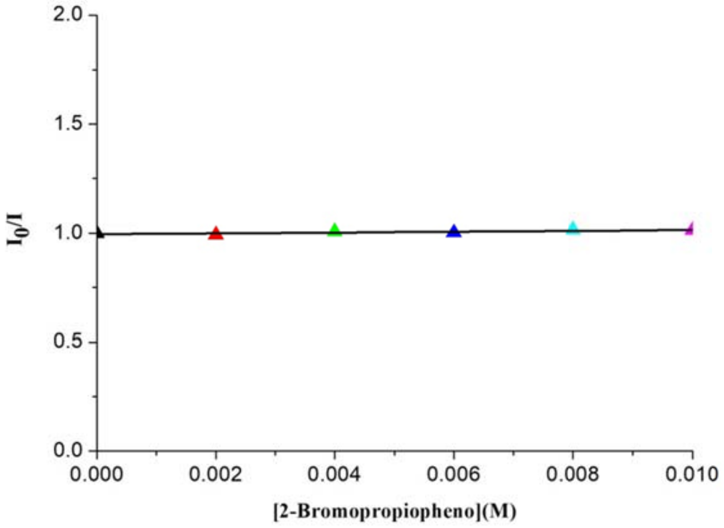

Supplementary Figure 144. Stern–Volmer quenching experiment of DPZ and **1a**. No quenching observed.

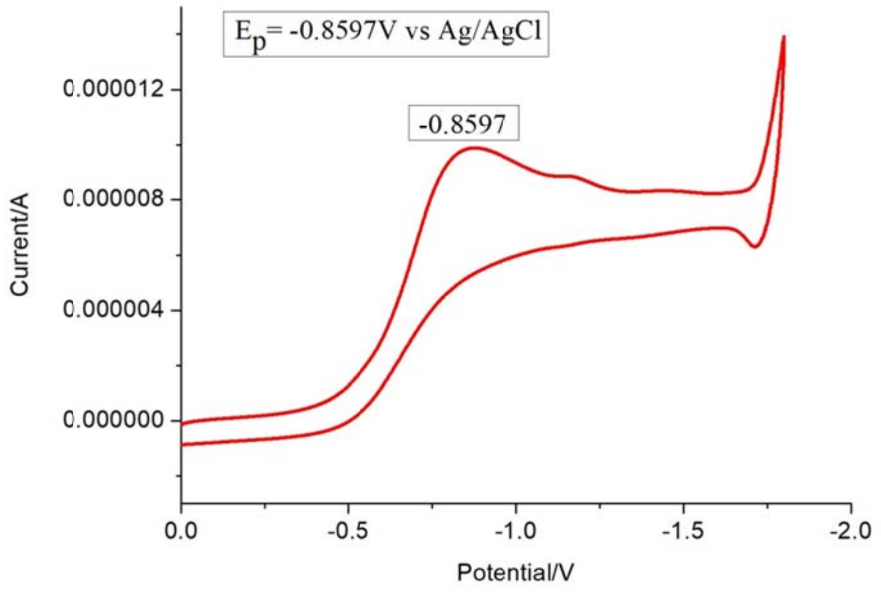

Supplementary Figure 145. Cyclic voltammogram of **1a** in MeCN.

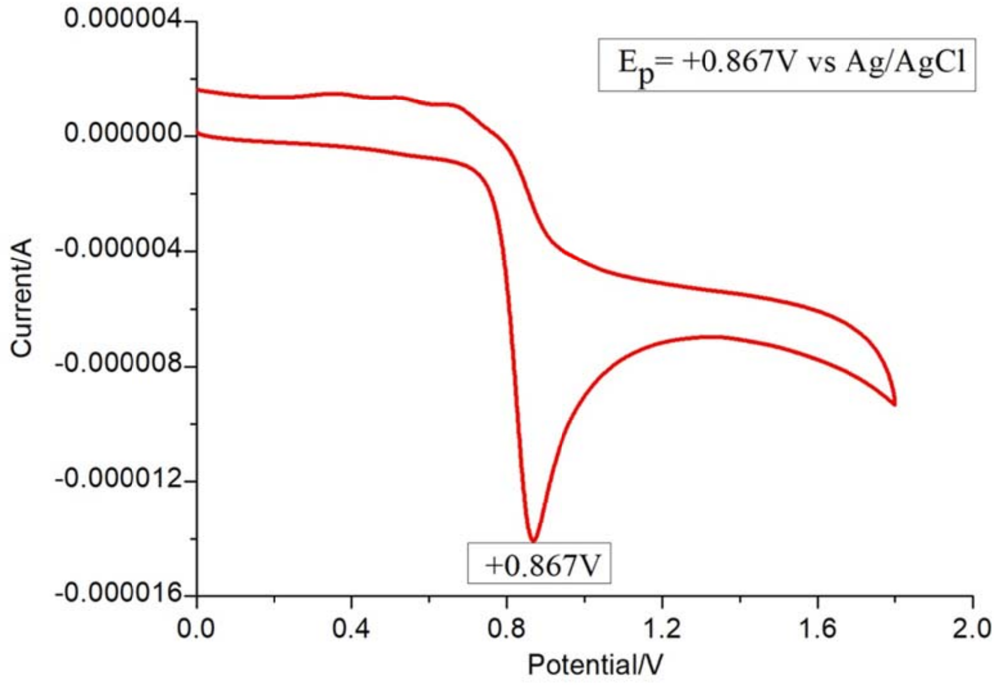

Supplementary Figure 146. Cyclic voltammogram of *N*-methyl aniline in MeCN.

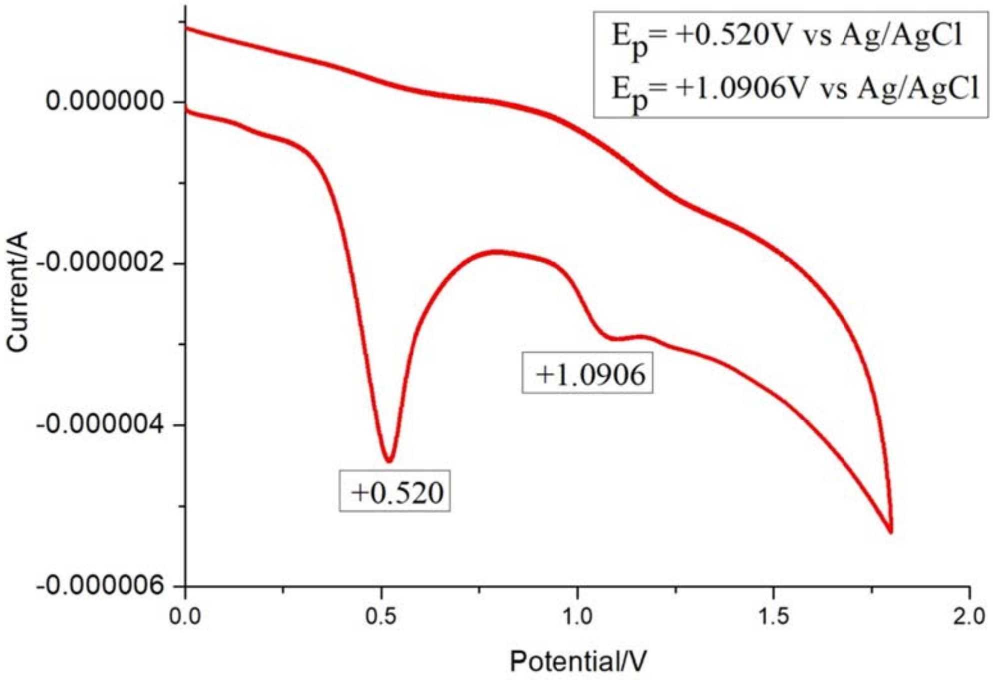

Supplementary Figure 147. Cyclic voltammogram of $PhNHCH_2CO_2Na$ in MeCN.

Supplementary Tables

Supplementary Table 1. Optimization of Reaction Conditions.

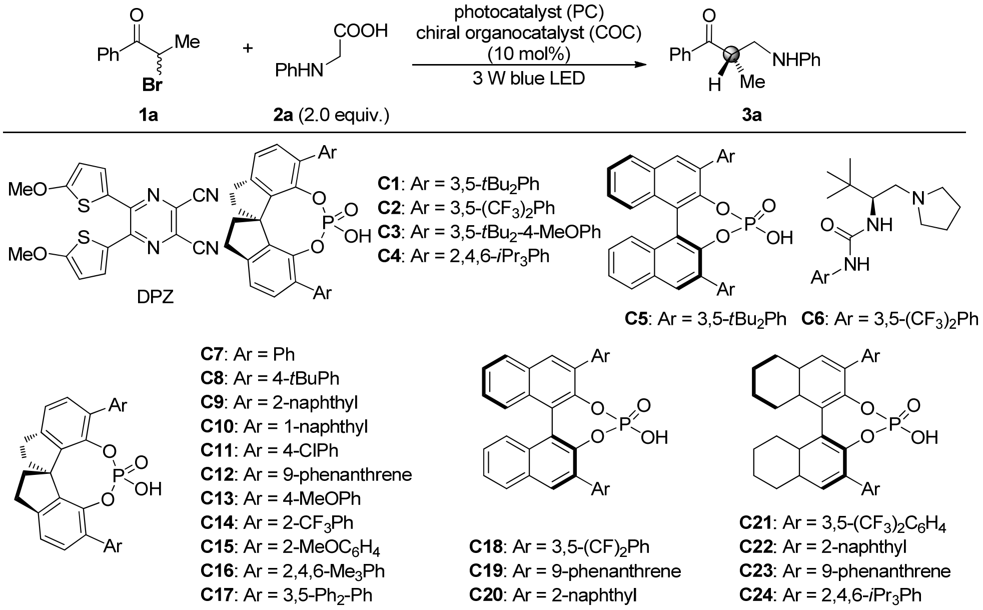

entry	PC (mol%)	COC	Base (3 equiv)	Molecular Sieve (25 mg)	solvent (750 μL)	*T* (°C)	ee (%)†
1	DPZ (1.0)	**C2**	$NaHCO_3$	--	CH_2Cl_2	25	61
2	DPZ (1.0)	**C4**	$NaHCO_3$	--	CH_2Cl_2	25	47
3	DPZ (1.0)	**C5**	$NaHCO_3$	--	CH_2Cl_2	25	23
4	DPZ (1.0)	**C6**	$NaHCO_3$	--	CH_2Cl_2	25	0
5	DPZ (1.0)	**C7**	$NaHCO_3$	--	CH_2Cl_2	25	40
6	DPZ (1.0)	**C8**	$NaHCO_3$	--	CH_2Cl_2	25	35
7	DPZ (1.0)	**C9**	$NaHCO_3$	--	CH_2Cl_2	25	52
8	DPZ (1.0)	**C10**	$NaHCO_3$	--	CH_2Cl_2	25	50
9	DPZ (1.0)	**C11**	$NaHCO_3$	--	CH_2Cl_2	25	38
10	DPZ (1.0)	**C12**	$NaHCO_3$	--	CH_2Cl_2	25	54
11	DPZ (1.0)	**C13**	$NaHCO_3$	--	CH_2Cl_2	25	39
12	DPZ (1.0)	**C14**	$NaHCO_3$	--	CH_2Cl_2	25	23
13	DPZ (1.0)	**C15**	$NaHCO_3$	--	CH_2Cl_2	25	37
14	DPZ (1.0)	**C16**	$NaHCO_3$	--	CH_2Cl_2	25	39
15	DPZ (1.0)	**C17**	$NaHCO_3$	--	CH_2Cl_2	25	48
16	DPZ (1.0)	**C18**	$NaHCO_3$	--	CH_2Cl_2	25	25
17	DPZ (1.0)	**C19**	$NaHCO_3$	--	CH_2Cl_2	25	32
18	DPZ (1.0)	**C20**	$NaHCO_3$	--	CH_2Cl_2	25	34
19	DPZ (1.0)	**C21**	$NaHCO_3$	--	CH_2Cl_2	25	29
20	DPZ (1.0)	**C22**	$NaHCO_3$	--	CH_2Cl_2	25	27
21	DPZ (1.0)	**C23**	$NaHCO_3$	--	CH_2Cl_2	25	23
22	DPZ (1.0)	**C24**	$NaHCO_3$	--	CH_2Cl_2	25	50
23	DPZ (1.0)	**C2**	$NaHCO_3$	--	toluene	25	55
24	DPZ (1.0)	**C2**	$NaHCO_3$	--	Et_2O	25	60
25	DPZ (1.0)	**C2**	$NaHCO_3$	--	CH_2Cl_2	10	61

26	DPZ (1.0)	**C2**	$NaHCO_3$	--	CH_2Cl_2	0	64
27	DPZ (1.0)	**C2**	$NaHCO_3$	--	CH_2Cl_2	−10	64
28	DPZ (1.0)	**C2**	$NaHCO_3$	--	THF	0	71
29	DPZ (1.0)	**C2**	$NaHCO_3$	--	CH_3CN	0	57
30	DPZ (1.0)	**C2**	$NaHCO_3$	--	Et_2O	0	64
31	DPZ (1.0)	**C2**	$NaHCO_3$	--	*i*Pr_2O	0	53
32	DPZ (1.0)	**C2**	$NaHCO_3$	--	*n*Bu_2O	0	60
33	DPZ (1.0)	**C2**	$NaHCO_3$	--	dioxane	0	67
34	DPZ (1.0)	**C2**	$NaHCO_3$	--	MTBE	0	62
35	DPZ (1.0)	**C2**	$NaHCO_3$	--	C_6F_5H	0	52
36	DPZ (1.0)	**C2**	$NaHCO_3$	--	$CHCl_3$	0	58
37	DPZ (1.0)	**C2**	$NaHCO_3$	--	$ClCH_2CH_2Cl$	0	60
38	DPZ (1.0)	**C2**	$NaHCO_3$	--	1,2-dimethoxyethane	0	70
39	DPZ (1.0)	**C2**	$NaHCO_3$	--	*t*BuPh	0	58
40	DPZ (1.0)	**C2**	$NaHCO_3$	--	mesitylene	0	57
41	DPZ (1.0)	**C2**	$NaHCO_3$	--	CPME	0	63
42	DPZ (1.0)	**C2**	$NaHCO_3$	5 Å	THF	0	75
43	DPZ (1.0)	**C1**	$NaHCO_3$	5 Å	THF	0	85
44	DPZ (1.0)	**C1**	$KHCO_3$	5 Å	THF	0	72
45	DPZ (1.0)	**C1**	Na_2CO_3	5 Å	THF	0	73
46	DPZ (1.0)	**C1**	K_2CO_3	5 Å	THF	0	N.P.
47	DPZ (1.0)	**C1**	K_2HPO_4	5 Å	THF	0	72
48	DPZ (1.0)	**C1**	AcONa	5 Å	THF	0	N.P.
49	DPZ (1.0)	**C1**	KH_2PO_4	5 Å	THF	0	N.P.
50	DPZ (1.0)	**C1**	NaH_2PO_4	5 Å	THF	0	N.P.
51	DPZ (1.0)	**C1**	Na_2HPO_4	5 Å	THF	0	74
52	DPZ (0.5)	**C1**	$NaHCO_3$	5 Å	THF	0	88
53	DPZ (0.3)	**C1**	$NaHCO_3$	5 Å	THF	0	88
54	DPZ (0.1)	**C1**	$NaHCO_3$	5 Å	THF	0	87
55	DPZ (0.5)	**C1**	$NaHCO_3$	5 Å	1,2-dimethoxyethane	0	93
56	DPZ (0.5)	**C1**	$NaHCO_3$	3 Å	1,2-dimethoxyethane	0	95
57	DPZ (0.5)	**C2**	$NaHCO_3$	3 Å	1,2-dimethoxyethane	0	87
58	DPZ (0.5)	**C3**	$NaHCO_3$	3 Å	1,2-dimethoxyethane	0	94
59	DPZ (0.5)	**C4**	$NaHCO_3$	3 Å	1,2-dimethoxyethane	0	72

Reaction conditions: **1a** (0.05 mmol), **2a** (0.10 mmol). N.P. = no product **3a** obtained.

†Determined by HPLC analysis on a chiral stationary phase.

Supplementary Table 2. Evaluation of Photoredox Catalysts under the Standard Conditions.

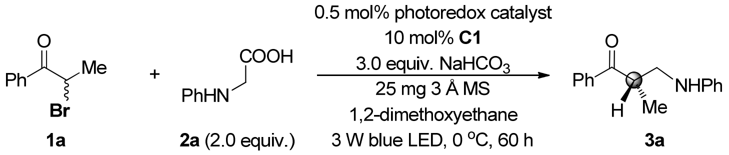

entry	photoredox catalyst	yield (%)†	ee (%)‡
1	Rose Bengal	65	94
2	Eosin Y	60	92
3	Rhodamine B	64	94
4	$Ru(bpy)_3Cl_3•6H_2O$	62	90

Reaction conditions: **1a** (0.05 mmol), **2a** (0.10 mmol).

†Yields were determined by isolation after chromatographic purification.

‡Determined by HPLC analysis on a chiral stationary phase.

Supplementary Table 3. Evaluation of Other α-Aminoalkyl Radical Precursors.

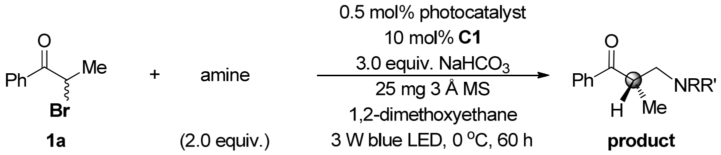

entry	amine	yield (%)†	ee (%)‡
1	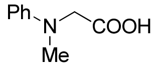	45	9
2	PhNHMe	no reaction	N.A.

Reaction conditions: **1a** (0.05 mmol), **2a** (0.10 mmol). N.A. = not available.

†Yields were determined by isolation after chromatographic purification.

‡Determined by HPLC analysis on a chiral stationary phase. N.A. = not available.

Supplementary Note 1

Synthesis of 13

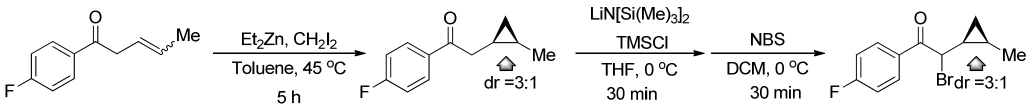

20 **21**: 80% yield **13**: 75% yield

To a solution of **20** (5 mmol, 1.0 equiv) in 10 mL of dry toluene was added CH_2I_2 (10 mmol, 2 equiv) under nitrogen at 0 °C. The solution of Et_2Zn (13 mmol, 1.0 M in THF) was added slowly. Then the reaction worked at 45 ºC. After 5 h, the reaction mixture was diluted with 30 mL EtOAc, and quenched with saturated NH_4Cl. The organic layer was separated and extracted with EtOAc, and the combined organics was then dried over Na_2SO_4 and concentrated in *vacuo*. The reaction mixture was directly loaded onto a short *silica gel* column, followed by gradient elution with petroleum ether/ethyl acetate (200/1–50/1 ratio). Removing the solvent in *vacuo*, afforded product **21** (Supplementary Figure 73).

To a solution of **21** (4 mmol, 1.0 equiv) in 10 mL of dry THF was added Lithium bis(trimethylsilyl)amide (6 mmol, 1.0 M in THF) under nitrogen at 0 °C. After stirred at 0 ºC for 30 mi, TMSCl (6 mmol, 1.5 equiv) was added slowly. The reaction worked at 0 ºC for additional 30 min, the reaction mixture was quenched with saturated $NaHCO_3$. The organic layer was separated and extracted with EtOAc, and the combined organics was then dried over Na_2SO_4 and concentrated in *vacuo*. The residue was dissolved in 10 mL CH_2Cl_2 and subsequently NBS (4.8 mmol, 1.2 equiv) was added at 0 ºC. After the starting material was consumed through analysis of TLC, the reaction mixture was directly loaded onto a short *silica gel* column, followed by gradient elution with petroleum ether/ethyl acetate (200/1–50/1 ratio). Removing the solvent in *vacuo*, afforded product **13** (Supplementary Figure 68).

Synthesis of 22 directly from 13

To determine the dr of the cyclopropyl moiety of **13** and compare the corresponding dr of compound **22** derived from **14** (Supplementary Note 2), we attempted to synthesize **22** directly from **13**.

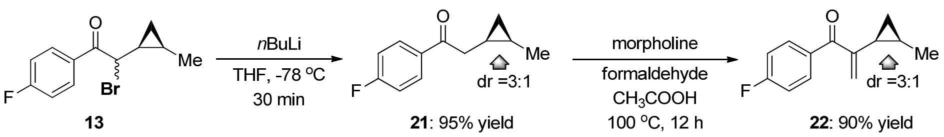

To a solution of **13** (0.2 mmol, 1.0 equiv) in 5.0 mL of dry THF was added *n*BuLi (0.3 mmol, 1.0 M in hexane) slowly at −78 °C. The reaction was conducted at the same temperature for 30 min. After quenched with saturated NH_4Cl, the organic layer was separated and extracted with EtOAc. The combined organic phase was dried over Na_2SO_4 and concentrated in *vacuo*. The reaction mixture was directly loaded onto a short *silica gel* column, followed by gradient elution with petroleum ether/ethyl acetate (200/1–50/1 ratio). Removing the solvent in *vacuo*, afforded product **21** with the maintained dr.

The mixture of **21** (0.5 mmol) and morpholine (0.25 mmol, 0.5 equiv) in 1.0 mL of glacial acetic acid was heated under reflux and a 37% aqueous formaldehyde solution (3 mmol, 6 equiv) was added slowly. The progress of the reaction was monitored by TCL. After completion of the reaction, acetic acid was pumped off under reduced pressure and the residue was diluted with ethyl acetate. The resulting solution was washed successively with an aqueous solution of $NaHCO_3$ (10%), followed by 10% aqueous hydrochloric acid, brine and water. The organic layer was separated and dried with anhydrous Na_2SO_4 and the solvent was evaporated off. The reaction mixture was directly loaded onto a short *silica gel* column, followed by gradient elution with petroleum ether/ethyl acetate (200/1–50/1 ratio). Removing the solvent in *vacuo*, afforded product **22** (supplementary Figure 74). The NMR analysis indicated that the dr of the cyclopropyl moiety was same as the obtained **22** from **13** (Supplementary Note 2).

Synthesis of 17

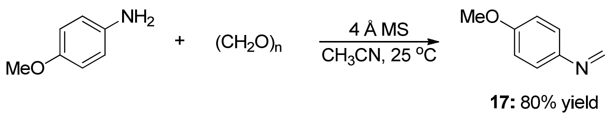

To a solution of 4-methoxy-phenylamine (1 mmol, 1.0 equiv) in 5.0 mL of CH_3CN was added 4Å Molecular Sieve (200.0 mg) and Paraformaldehyde (5.0 equiv). The mixture was stirred for 12 h at 25 °C, 4Å Molecular Sieve was filtered off and the solvent removed under vacuum to give crude **17**, which was used for the next step without further purification (Supplementary Figure 72).

Supplementary Note 2

Mechanism studies

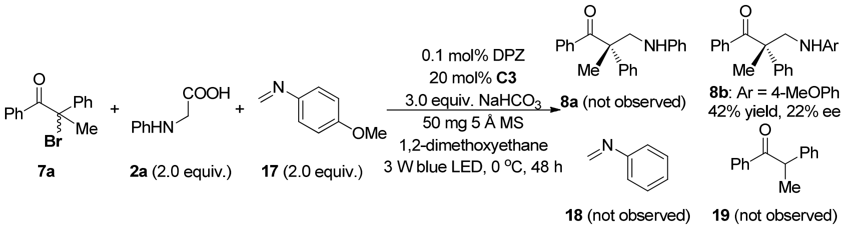

7.1 μL (0.0001 mmol, 0.001 equiv) of DPZ solution (1.0 mg of DPZ in 200 μL of toluene) was added into a 10 mL Schlenk tube, and then solvent was removed in *vacuo*. Subsequently, **7a** (0.1 mmol, 1.0 equiv), **2a** (0.2 mmol, 2.0 equiv), **17** (0.2 mmol, 2.0 equiv), **C3** (0.02 mmol, 0.2 equiv), 5Å Molecular Sieve (50.0 mg), $NaHCO_3$ (25.2 mg, 3.0 equiv), 1,2-dimethoxyethane (1.5 mL) were sequentially added, degassed three times by freeze-pump-thaw method. The reaction mixture was stirred under an argon atmosphere at 0 °C (the temperature was maintained in an incubator) for 30 min without light, then irradiated by a 3 W blue LED (λ = 450−455 nm) from 3.0 cm distance for another 48 h. The reaction mixture was directly loaded onto a short *silica gel* column, followed by gradient elution with petroleum ether/ethyl acetate (200/1–20/1 ratio). Removing the solvent in *vacuo*, afforded products **8b** in 42% yield with 22% ee.

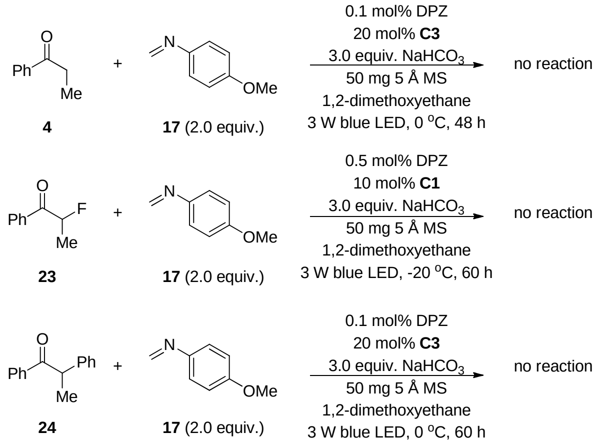

The reactions of ketone **4**, **23** and **24** with imine **17** were performed under the corresponding optimal conditions as described in the above figure. No reaction was observed in all of three transformations indicating the unavailability of Mannich reaction of ketone to imine under the established reaction conditions.

NMR analysis for bromoketones or *N*-aryl amino acids with catalyst

Acetophenone (12.0 mg, 0.1 mmol) and diphenyl phosphate (25.0 mg, 0.1 mmol) were dissolved in $CDCl_3$-*d*$_3$ (0.5 mL). The ^{13}C NMR analysis (Supplementary Figs 77–78) showed a shift of the carbonyl signal peak of acetophenone to the downfield, indicating a H-bonding interaction existing between them. Meanwhile, the ^{13}C NMR analysis of the mixture of *N*-methylaniline (10.7mg, 0.1 mmol) and diphenyl phosphate (25.0 mg, 0.1 mmol) in $CDCl_3$-*d*$_3$ (0.5 mL) exhibited a robust shift to the downfield for the methyl signal peak of *N*-methylaniline (Supplementary Figs 79–80). Accordingly, the H-bonding interaction of phosphoric acid with the secondary amine also remains.

Radical clock experiments

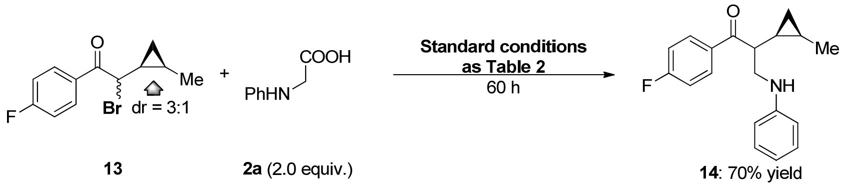

35.4 μL (0.0005 mmol, 0.005 equiv) of DPZ solution (1.0 mg of DPZ in 200 μL of toluene) was added into a 10 mL Schlenk tube, and then solvent was removed in *vacuo*. Subsequently, **13** (0.1 mmol, 1.0 equiv), **2a** (0.2 mmol, 2.0 equiv), **C1** (0.01 mmol, 0.1 equiv), 3Å MS (50.0 mg), $NaHCO_3$ (25.2 mg, 3.0 equiv), in 1,2-dimethoxyethane (1.5 mL) were sequentially added, degassed three times by freeze-pump-thaw method. The reaction mixture was stirred under an argon atmosphere at 0 °C (the temperature was maintained in an incubator) for 30 min without light, then irradiated by a 3 W blue LED (λ = 450−455 nm) from 3.0 cm distance for another 60 h. The reaction mixture was directly loaded onto a short *silica gel* column, followed by gradient elution with petroleum ether/ethyl acetate (200/1–20/1 ratio). Removing the solvent in *vacuo*, afforded product **14**.

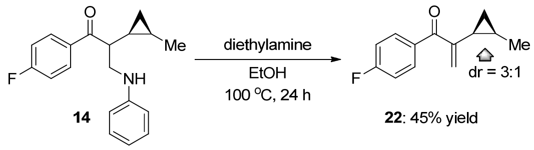

To a solution of **14** (0.2 mmol, 1.0 equiv) in 1.0 mL of dry EtOH was added diethylamine (1 mmol, 5 equiv) in Schlenk tube, which was stirred for 24 h at 100 °C. After the starting material was consumed through analysis of TLC, the reaction mixture was directly loaded onto a short *silica gel* column, followed by gradient elution with petroleum ether/ethyl acetate (200/1–50/1 ratio). Removing the solvent in *vacuo*, afforded product **22** with 3:1 dr

(supplementary Figs 74).

Radical trap experiments

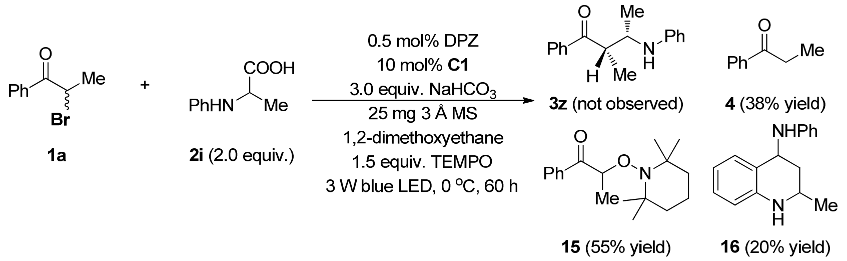

35.4 μL (0.0005 mmol, 0.005 equiv) of DPZ solution (1.0 mg of DPZ in 200 μL of toluene) was added into a 10 mL Schlenk tube, and then solvent was removed in *vacuo*. Subsequently, **1a** (0.1 mmol, 1.0 equiv), **2i** (33.0 mg, 2.0 equiv), **C1** (0.01 mmol, 0.1 equiv), 3Å Molecular Sieve (50.0 mg), $NaHCO_3$ (25.2 mg, 3.0 equiv), TEMPO (0.15 mmol, 1.5 equiv) in 1,2-dimethoxyethane (1.5 mL) were sequentially added, degassed three times by freeze-pump-thaw method. The reaction mixture was stirred under an argon atmosphere at 0 °C (the temperature was maintained in an incubator) for 30 min without light, then irradiated by a 3 W blue LED (λ = 450−455 nm) from 3.0 cm distance for another 60 h. The reaction mixture was directly loaded onto a short *silica gel* column, followed by gradient elution with petroleum ether/ethyl acetate (200/1–20/1 ratio). Removing the solvent in *vacuo*, afforded products **4**, **15**, **16**.

Linear effect experiments

These reactions were conducted according to the general procedure (see 3.1). 35.4 μL (0.0005 mmol, 0.005 equiv) of DPZ solution (1.0 mg of DPZ in 200 μL of toluene) was added into a 10 mL Schlenk tube, and then solvent was removed in *vacuo*. Subsequently, α-bromoketone (0.1 mmol, 1.0 equiv), **2** (0.2 mmol, 2.0 equiv), **C2** (0.01 mmol, 0.1 equiv, x% ee), 3Å Molecular Sieve (50.0 mg), $NaHCO_3$ (25.2 mg, 3.0 equiv), in 1,2-dimethoxyethane (1.5 mL) were sequentially added, degassed three times by freeze-pump-thaw method, The reaction mixture was stirred under an argon atmosphere at 0 °C (the temperature was maintained in an incubator) for 30 min without light, then irradiated by a 3 W blue LED (λ = 450−455 nm) from 3.0 cm distance for another 60 h. The product was separated by preparative TLC. Chiral HPLC analysis gave linear effect data. The ee of **C2** was determined by HPLC analysis: CHIRALPAK IE (4.6 mm i.d. x 250 mm); Hexane/2-propanol/Diethylamine = 97.9/2/0.1;

flow rate 1.0 mL/min; 25 °C; 254 nm; retention time: 9.1 min, 14.8 min.The ee of **3t** was determined by HPLC analysis: CHIRALPAK IE (4.6 mm i.d. x 250 mm); Hexane/2-propanol = 90/10; flow rate 1.0 mL/min; 25 °C; 254 nm; retention time: 9.5 min, 11.2 min (Supplementary Figs 135–142).

Emission quenching experiments

Emission intensities were recorded on a spectrofluorometer. DPZ solution was excited at 448 nm and the emission intensity at 544 nm was observed. A solution of DPZ (5.0×10^{-4} M) in 1,2-dimethoxyethane was added to the appropriate amount of quencher in 5.0 mL volumetric flask under N_2. The solution was transferred to a 1.5 mL quartz cell and the emission spectrum of the sample was collected (Supplementary Figs 143–144).

Cyclic voltammetry measurement

Electrochemical potentials were obtained with a standard set of conditions to main internal consistency. Cyclic voltammograms were collected with a potentiostat. Samples were prepared with 0.01 mmol of **1a**, *N*-methyl aniline, or $PhNHCH_2CO_2Na$ in 10 mL of 0.1 M tetrabutylammonium hexafluorophosphate in anhydrous acetonitrile. Measurements employed a radium glassy carbon working electrode, platinum wire counter electrode, saturated KCl silver-silver chloride reference electrode. The obtained value was referenced to Ag/AgCl (Supplementary Figs 145–147).

Supplementary Note 3

Synthetic applications

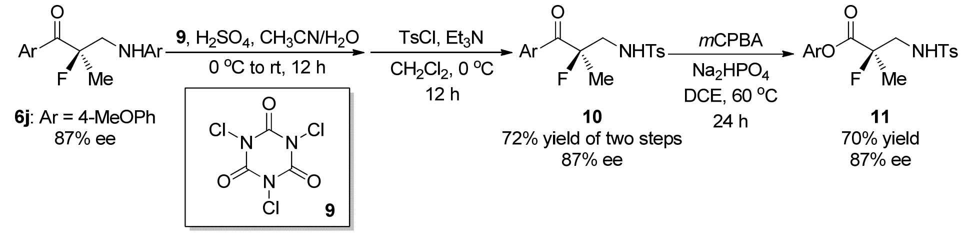

6j (0.2 mmol, 1.0 equiv) was dissolved in 2.0 mL acetonitrile and subsequently 1.0 mL water, **9** (0.2 mmol, 1.0 equiv), and 0.2 mL H_2SO_4 (1.0 M) were added at 0 °C. The mixture was stirred for 12 h. The reaction mixture was allowed to ambient temperature and was washed with 2.0 mL CH_2Cl_2 three times. The resulting aqueous phase was subsequently regulated to pH 8.0 through addition of saturated $NaHCO_3$ solution. The aqueous solution was extracted with 5.0 mL EtOAc for three times. The combined organic layers were dried over Na_2SO_4 and concentrated in *vacuo*. The residue was dissolved in 5.0 mL DCM and subsequently TsCl (0.6 mmol, 3.0 equiv) and Et_3N (0.6 mmol, 3.0 equiv) were added at 0 °C. The reaction was monitored by TLC. After completion of the reaction (12 h), the reaction mixture was directly loaded onto a short *silica gel* column, followed by gradient elution with petroleum ether/ethyl acetate (100/1–10/1 ratio). Removing the solvent in *vacuo*, afforded products $\beta^{2,2}$-amino ketone **10**.

$\beta^{2,2}$-amino ketone **10** (0.1 mmol, 1.0 equiv) was dissolved in 5.0 mL DCE and subsequently *m*CPBA (0.5 mmol, 5.0 equiv), and Na_2HPO_4 (0.3 mmol, 3.0 equiv) were added. The mixture was refluxed at 60 °C for 24 h. After the starting material was consumed by analysis of TLC (24 h), the heterogeneous mixture was dissolved in CH_2Cl_2 and quenched with a saturated aqueous solution of $Na_2S_2O_3$. The organic layer was then extracted with CH_2Cl_2, washed with a saturated aqueous solution of $NaHCO_3$. The organic layer was then dried over Na_2SO_4 and concentrated in *vacuo*. The reaction mixture was directly loaded onto a short *silica gel* column, followed by gradient elution with petroleum ether/ethyl acetate (100/1–10/1 ratio). Removing the solvent in *vacuo*, afforded products ester **11**.

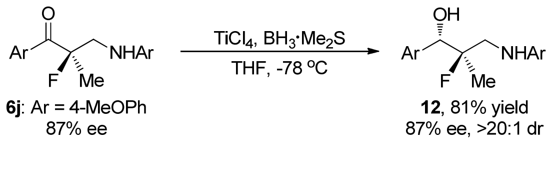

To a solution of **6j** (0.1 mmol, 1.0 equiv) in 5.0 mL of dry THF at -78 ^{o}C was added $TiCl_4$ (0.15 mmol, 1.5 equiv) to give immediately a yellow solution, which was stirred for 15 min at this temperature. The complex BH_3•Me_2S (0.4 mmol, solution 2.0 M in THF) was then added. After 24 h, the heterogeneous mixture was quenched with H_2O, and the reaction was warmed to room temperature. The organic layer was separated and extracted with EtOAc, and the combined organics was then dried over Na_2SO_4 and concentrated in *vacuo*. The reaction mixture was directly loaded onto a short *silica gel* column, followed by gradient elution with petroleum ether/ethyl acetate (100/1–10/1 ratio). Removing the solvent in *vacuo*, afforded product **12**.

Supplementary Note 4

Determination of the absolute configurations

(1) Absolute configurations of **3a-3z** are determined by *X*-ray structure analysis of the product **25** derived from **3y**

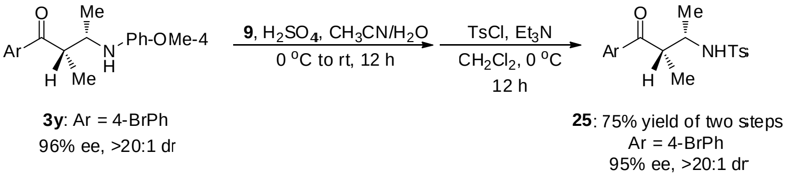

3y (0.2 mmol, 1.0 equiv) was dissolved in 2.0 mL acetonitrile and subsequently 1.0 mL water, **9** (0.2 mmol, 1.0 equiv), and 0.2 mL 1.0 M H_2SO_4 were added at 0 °C. The mixture was stirred for 12 h. The reaction mixture was allowed to ambient temperature and was washed with 2.0 mL CH_2Cl_2 three times. The resulting aqueous phase was subsequently regulated to pH 8.0 through addition of saturated $NaHCO_3$ solution. The aqueous solution was extracted with 5.0 mL EtOAc for three times. The combined organic layers were dried over Na_2SO_4 and concentrated in *vacuo*. The residue was dissolved in 5.0 mL DCM and subsequently TsCl (0.6 mmol, 3.0 equiv) and Et_3N (0.6 mmol, 3.0 equiv) were added at 0 °C. The reaction was monitored by TLC. After completion of the reaction (12 h), the reaction mixture was directly loaded onto a short *silica gel* column, followed by gradient elution with petroleum ether/ethyl acetate (100/1–10/1 ratio). Removing the solvent in *vacuo*, afforded product **25**.

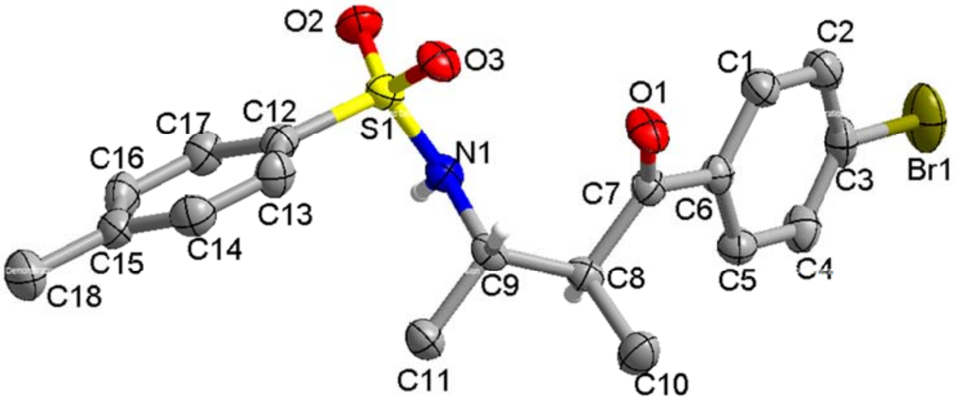

Supplementary Figure 148. Absolute configuration of **25** (CCDC 1589705).

Displacement ellipsoids are drawn at the 30% probability level. (Solvent: ethyl acetate)

Supplementary Table 4. Crystal data and structure refinement.

Identification code	ljtts_twin1_hklf4
Empirical formula	$C_{18}H_{20}BrNO_3S$

Formula weight	410.32
Temperature/K	293(2)
Crystal system	orthorhombic
Space group	$P2_12_12_1$
a/Å	5.2693(3)
b/Å	11.5391(7)
c/Å	30.1214(13)
α/°	90
β/°	90
γ/°	90
Volume/Å^3	1831.49(17)
Z	4
ρ_{calc}g/cm^3	1.488
μ/mm^{-1}	4.256
F(000)	840.0
Crystal size/mm^3	0.15 × 0.09 × 0.06
Radiation	CuKα (λ = 1.54184)
2Θ range for data collection/°	8.206 to 142.232
Index ranges	$-6 \le h \le 6$, $-13 \le k \le 13$, $-36 \le l \le 35$
Reflections collected	6638
Independent reflections	6638 [R_{int} = ?, R_{sigma} = 0.0441]
Data/restraints/parameters	6638/1/225
Goodness-of-fit on F^2	1.011
Final R indexes [I>=2σ (I)]	R_1 = 0.0417, wR_2 = 0.1033
Final R indexes [all data]	R_1 = 0.0607, wR_2 = 0.1085
Largest diff. peak/hole / e Å^{-3}	0.24/-0.30
Flack parameter	-0.04(2)

Experimental

The crystal was kept at 293(2) K during data collection. Using Olex2, the structure was solved with the ShelXS structure solution program using Direct Methods and refined with the ShelXL refinement package using Least Squares minimisation.

Crystal structure determination

Crystal Data for $C_{18}H_{20}BrNO_3S$ (*M* =410.32 g/mol): orthorhombic, space group $P2_12_12_1$ (no. 19), *a* = 5.2693(3) Å, *b* = 11.5391(7) Å, *c* = 30.1214(13) Å, *V* = 1831.49(17) Å^3, *Z* = 4, *T* = 293(2) K, μ(CuKα) = 4.256 mm^{-1}, *Dcalc* = 1.488 g/cm^3, 6638 reflections measured (8.206°

≤ 2Θ ≤ 142.232°), 6638 unique (R_{int} = ?, R_{sigma} = 0.0441) which were used in all calculations. The final R_1 was 0.0417 (I > 2σ(I)) and wR_2 was 0.1085 (all data).

(2) Absolute configurations of **6a-6m**, **10**, and **11** are determined by *X*-ray structure analysis of **12**

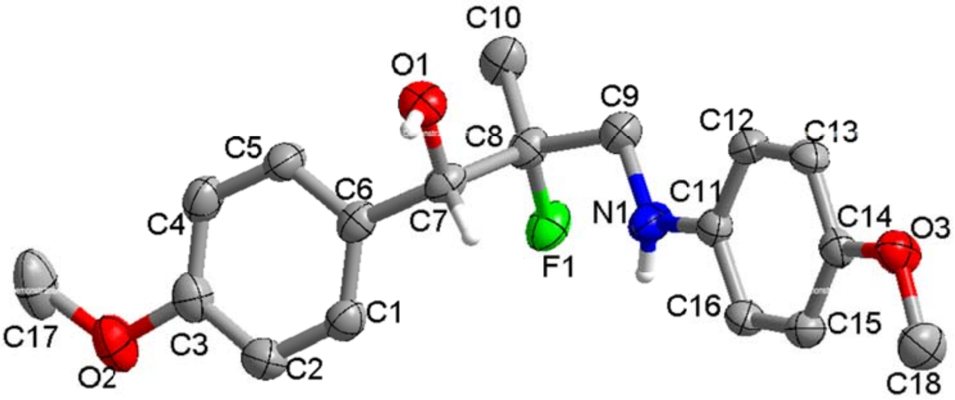

Supplementary Figure 149. Absolute configuration of **12** (CCDC 1587114).

Displacement ellipsoids are drawn at the 30% probability level.

(Solvent: ethyl acetate: hexane = 1:1)

Supplementary Table 5. Crystal data and structure refinement.

Identification code	201710218
Empirical formula	$C_{18}H_{22}FNO_3$
Formula weight	319.36
Temperature/K	293(2)
Crystal system	orthorhombic
Space group	$P2_12_12_1$
a/Å	5.69563(16)
b/Å	7.7161(2)
c/Å	38.1150(11)
α/°	90
β/°	90
γ/°	90
Volume/Å^3	1675.09(9)
Z	4
ρ_{calc}g/cm^3	1.266
μ/mm^{-1}	0.768
F(000)	680.0
Crystal size/mm^3	0.18 × 0.1 × 0.08
Radiation	CuKα (λ = 1.54184)
2Θ range for data collection/°	9.282 to 134.012
Index ranges	$-6 \leq h \leq 4$, $-7 \leq k \leq 9$, $-45 \leq l \leq 39$
Reflections collected	6035

Independent reflections	2996 [R_{int} = 0.0263, R_{sigma} = 0.0361]
Data/restraints/parameters	2996/0/219
Goodness-of-fit on F^2	1.036
Final R indexes [I>=2σ (I)]	R_1 = 0.0399, wR_2 = 0.0921
Final R indexes [all data]	R_1 = 0.0501, wR_2 = 0.0982
Largest diff. peak/hole / e $Å^{-3}$	0.10/-0.15
Flack parameter	-0.04(15)

Experimental

The crystal was kept at 293(2) K during data collection. Using Olex2, the structure was solved with the ShelXS structure solution program using Direct Methods and refined with the ShelXL refinement package using Least Squares minimisation.

Crystal structure determination

Crystal Data for $C_{18}H_{22}FNO_3$ (M =319.36 g/mol): orthorhombic, space group $P2_12_12_1$ (no. 19), a = 5.69563(16) Å, b = 7.7161(2) Å, c = 38.1150(11) Å, V = 1675.09(9) $Å^3$, Z = 4, T = 293(2) K, μ(CuKα) = 0.768 mm^{-1}, $Dcalc$ = 1.266 g/cm^3, 6035 reflections measured (9.282° ≤ 2Θ ≤ 134.012°), 2996 unique (R_{int} = 0.0263, R_{sigma} = 0.0361) which were used in all calculations. The final R_1 was 0.0399 (I > 2σ(I)) and wR_2 was 0.0982 (all data).

(3) Absolute configurations of **8a-8i** are determined by *X*-ray structure analysis of the product **26** derived from **8a**

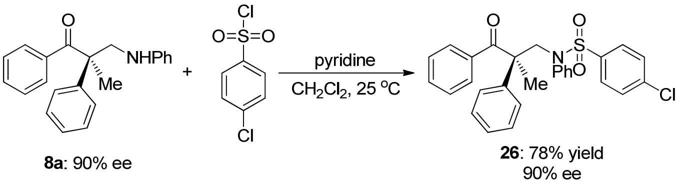

8a (0.1 mmol, 1.0 equiv) was dissolved in 5.0 mL DCM. Subsequently, 4-chloro-benzenesulfonicacichloride (0.5 mmol, 5.0 equiv) and pyridine (0.5 mmol, 5.0 equiv) were added at 0 °C. The mixture was stirred for 24 h at 25 °C. After the starting material was consumed through analysis of TLC (24 h), the heterogeneous mixture was quenched with 1.0 mL HCl (1.0 M). The aqueous solution was extracted with 5.0 mL DCM for three times. The combined organic layers were dried over Na_2SO_4 and concentrated in *vacuo*. The reaction mixture was directly loaded onto a short *silica gel* column, followed by gradient elution with petroleum ether/ethyl acetate (100/1–10/1 ratio). Removing the solvent in *vacuo*, afforded

product **26**.

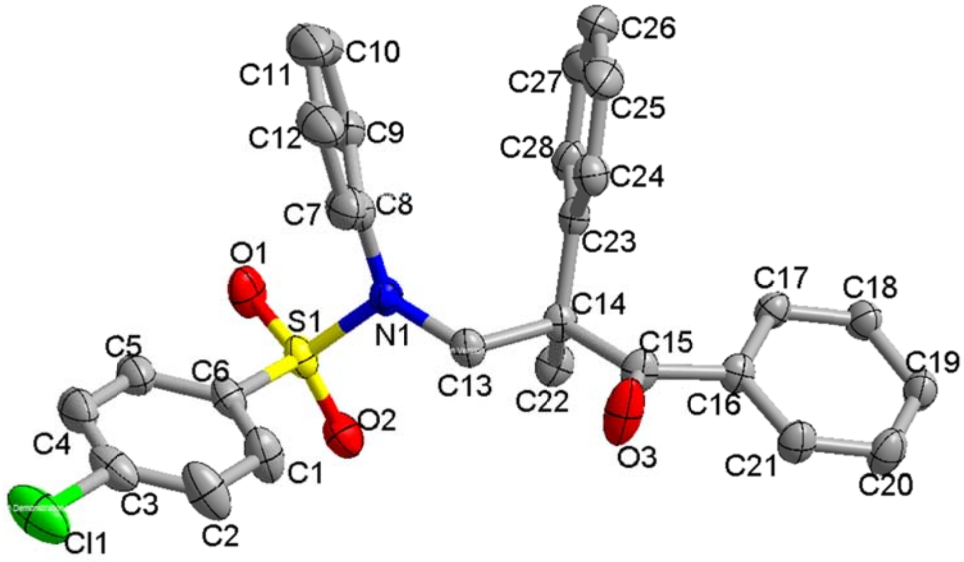

Supplementary Figure 150. Absolute configuration of **26** (CCDC 1814679).

Displacement ellipsoids are drawn at the 30% probability level. (Solvent: ethyl acetate)

Supplementary Table 6. Crystal data and structure refinement.

Identification code	20180101
Empirical formula	$C_{28}H_{24}ClNO_3S$
Formula weight	489.99
Temperature/K	293(2)
Crystal system	orthorhombic
Space group	$P2_12_12$
a/Å	39.8968(8)
b/Å	19.0996(4)
c/Å	6.46063(11)
α/°	90
β/°	90
γ/°	90
Volume/Å^3	4923.08(17)
Z	8
ρ_{calc}g/cm^3	1.322
μ/mm^{-1}	2.410
F(000)	2048.0
Crystal size/mm^3	0.3 × 0.08 × 0.07
Radiation	CuKα (λ = 1.54184)
2Θ range for data collection/°	8.102 to 134.126
Index ranges	$-43 \leq h \leq 47$, $-21 \leq k \leq 22$, $-7 \leq l \leq 4$
Reflections collected	14400

Independent reflections	8012 [R_{int} = 0.0281, R_{sigma} = 0.0416]
Data/restraints/parameters	8012/0/615
Goodness-of-fit on F^2	1.020
Final R indexes [I>=2σ (I)]	R_1 = 0.0461, wR_2 = 0.1137
Final R indexes [all data]	R_1 = 0.0573, wR_2 = 0.1224
Largest diff. peak/hole / e Å^{-3}	0.29/-0.28
Flack parameter	0.010(12)

Experimental

The crystal was kept at 293(2) K during data collection. Using Olex2, the structure was solved with the ShelXS structure solution program using Direct Methods and refined with the ShelXL refinement package using Least Squares minimisation.

Crystal structure determination

Crystal Data for $C_{28}H_{24}ClNO_3S$ (*M* =489.99 g/mol): orthorhombic, space group $P2_12_12$ (no. 18), *a* = 39.8968(8) Å, *b* = 19.0996(4) Å, *c* = 6.46063(11) Å, *V* = 4923.08(17) Å^3, *Z* = 8, *T* = 293(2) K, μ(CuKα) = 2.410 mm^{-1}, *Dcalc* = 1.322 g/cm^3, 14400 reflections measured (8.102° ≤ 2Θ ≤ 134.126°), 8012 unique (R_{int} = 0.0281, R_{sigma} = 0.0416) which were used in all calculations. The final R_1 was 0.0461 (I > 2σ(I)) and wR_2 was 0.1224 (all data).

Supplementary Methods

General information and materials: Experiments involving moisture and/or air sensitive components were performed under a positive pressure of argon in oven-dried glassware equipped with a rubber septum inlet. Dried solvents and liquid reagents were transferred by oven-dried syringes or hypodermic syringe cooled to ambient temperature in a desiccator. Reactions mixtures were stirred in 10 mL sample vial with Teflon-coated magnetic stirring bars unless otherwise stated. Moisture in non-volatile reagents/compounds was removed in high *vacuo* by means of an oil pump and subsequent purging with nitrogen. Solvents were removed *in vacuo* under ~30 mmHg and heated with a water bath at 30–35 ^oC using rotary evaporator with aspirator. The condenser was cooled with running water at 0 ^oC. All experiments were monitored by analytical thin layer chromatography (TLC). TLC was performed on pre-coated plates, 60 F_{254}. After elution, plate was visualized under UV illumination at 254 nm for UV active material. Further visualization was achieved by staining $Ce(SO_4)_2$ and anisaldehyde solution. For those using the aqueous stains, the TLC plates were heated on a hot plate. Columns for flash chromatography (FC) contained *silica gel* 200–300 mesh. Columns were packed as slurry of *silica gel* in petroleum ether and equilibrated solution using the appropriate solvent system. The elution was assisted by applying pressure of about 2 atm with an air pump.

Proton nuclear magnetic resonance (^1H NMR) and carbon NMR (^{13}C NMR) were recorded in $CDCl_3$ otherwise stated. Chemical shifts are reported in parts per million (ppm), using the residual solvent signal as an internal standard: $CDCl_3$ (^1H NMR: δ 7.26, singlet; ^{13}C NMR: δ 77.0, triplet). Multiplicities were given as: *s* (singlet), *d* (doublet), *t* (triplet), *q* (quartet), *quintet*, *m* (multiplets), *dd* (doublet of doublets), *dt* (doublet of triplets), and *br* (broad). Coupling constants (J) were recorded in Hertz (Hz). The number of proton atoms (n) for a given resonance was indicated by nH. The number of carbon atoms (n) for a given resonance was indicated by nC. HRMS (Analyzer: TOF) was reported in units of mass of charge ratio (m/z). Mass samples were dissolved in CH_3CN (HPLC Grade) unless otherwise stated. Optical rotations were recorded on a polarimeter with a sodium lamp of wavelength 589 nm and reported as follows; $[\alpha]_{\lambda}^{T°C}$ (c = g/100 mL, solvent). Melting points were determined on

a melting point apparatus. Enantiomeric excesses were determined by chiral High Performance Liquid Chromatography (HPLC) analysis. UV detection was monitored at 254 nm and 210 nm at the same time. HPLC samples were dissolved in HPLC grade isopropanol (IPA) unless otherwise stated. All commercial reagents were purchased with the highest purity grade. They were used without further purification unless specified. All solvents used, mainly petroleum ether (PE) and ethyl acetate (EtOAc) were distilled. Anhydrous dichloromethane (DCM), dioxane, $CHCl_3$, Et_3N, $ClCH_2CH_2Cl$, and CH_3CN were freshly distilled from CaH_2 and stored under N_2 atmosphere. THF, Et_2O, *i*Pr_2O, MTBE, *n*Bu_2O, 1,2-dimethoxyethane, *t*BuPh, mesitylene, CPME, and toluene were freshly distilled from sodium/benzophenone before use. All compounds synthesized were stored in a −20 °C freezer and light-sensitive compounds were protected with aluminium foil.

General experimental procedures

General procedure for reactions between *N*-aryl amino acids and secondary *α*-bromoketones

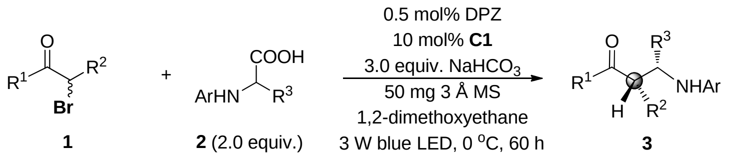

35.4 *μ*L (0.0005 mmol, 0.005 equiv) of DPZ solution (1.0 mg of DPZ in 200 *μ*L of toluene) was added into a 10 mL Schlenk tube, and then solvent was removed in *vacuo*. Subsequently, **1** (0.1 mmol, 1.0 equiv), **2** (0.2 mmol, 2.0 equiv), **C1** (0.01 mmol, 0.1 equiv) for **3a**–**3r**, **3u**, **3w**, **3x**, **3y**, **3z** or **C2** (0.01 mmol, 0.1 equiv) for **3s**, **3t**, **3v**, 3Å molecular Sieves (50.0 mg), $NaHCO_3$ (25.2 mg, 3.0 equiv), in 1,2-dimethoxyethane (1.5 mL) were sequentially added, degassed three times by freeze-pump-thaw method. The reaction mixture was stirred under an argon atmosphere at 0 °C (the temperature was maintained in an incubator) for 30 min without light, then irradiated by a 3 W blue LED (*λ* = 450−455 nm) from a 3.0 cm distance for another 60 h. The reaction mixture was directly loaded onto a short *silica gel* column, followed by gradient elution with petroleum ether/ethyl acetate (200/1–20/1 ratio). Removing the solvent in *vacuo*, afforded products **3a**−**3z**.

General procedure for enantioselective substitution of tertiary *α*-bromo-*α*-fluoroketones with *N*-aryl glycines

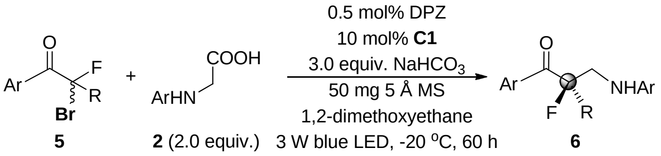

35.4 μL (0.0005 mmol, 0.005 equiv) of DPZ solution (1.0 mg of DPZ in 200 μL of toluene) was added into a 10 mL Schlenk tube, and then solvent was removed in *vacuo*. Subsequently, **5** (0.1 mmol, 1.0 equiv), **2** (0.2 mmol, 2.0 equiv), **C1** (0.01 mmol, 0.1 equiv) for **6a–6k** or **C3** (0.02 mmol, 0.2 equiv) for **6l** and **6m**, 5Å molecular sieves (50.0 mg), $NaHCO_3$ (25.2 mg, 3.0 equiv), in 1,2-dimethoxyethane (1.5 mL) were sequentially added, degassed three times by freeze-pump-thaw method. The reaction mixture was stirred under an argon atmosphere at −20 °C for **6a–6k** or −45 °C for **6l** and **6m** (the temperature was maintained in an incubator) for 30 min without light, then irradiated by a 3 W blue LED (λ = 450−455 nm) from a 3.0 cm distance for another 60 h. The reaction mixture was directly loaded onto a short *silica gel* column, followed by gradient elution with petroleum ether/ethyl acetate (200/1–20/1 ratio). Removing the solvent in *vacuo*, afforded products **6a−6m**.

General procedure for enantioselective substitution of tertiary *α*-bromoketones with *N*-phenyl glycine

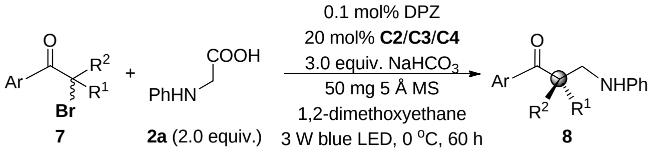

7.1 μL (0.0001 mmol, 0.001 equiv) of DPZ solution (1.0 mg of DPZ in 200 μL of toluene) was added into a 10 mL Schlenk tube, and then solvent was removed in *vacuo*. Subsequently, **7** (0.1 mmol, 1.0 equiv), **2** (0.2 mmol, 2.0 equiv), **C2** (0.02 mmol, 0.2 equiv) for **8f**, **8g**, **8h** or **C3** (0.02 mmol, 0.2 equiv) for **8a−8e** or **C4** (0.02 mmol, 0.2 equiv) for **8i**, 5Å molecular sieves (50.0 mg), $NaHCO_3$ (25.2 mg, 3.0 equiv), in 1,2-dimethoxyethane (1.5 mL) were sequentially added, degassed three times by freeze-pump-thaw method. The reaction mixture was stirred under an argon atmosphere at 0 °C (the temperature was maintained in an incubator) for 30 min without light, then irradiated by a 3 W blue LED (λ = 450−455 nm) from a 3.0 cm distance for another 60 h. The reaction mixture was directly loaded onto a short *silica gel* column, followed by gradient elution with petroleum ether/ethyl acetate (200/1–20/1 ratio). Removing the solvent in *vacuo*, afforded products **8a−8i**.

Characterization of adducts

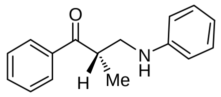

(*S*)-2-methyl-1-phenyl-3-(phenylamino)propan-1-one (3a): yellow oil; 18.7 mg, 78% yield; 95% ee; $[\alpha]_D^{22}$ +249.4 (*c* 1.0, $CHCl_3$); ^{1}H NMR (300 MHz, $CDCl_3$) δ 7.93 (d, *J* = 7.7 Hz, 2H), 7.57 (t, *J* = 7.0 Hz, 1H), 7.46 (t, *J* = 7.4 Hz, 2H), 7.17 (t, *J* = 7.4 Hz, 2H), 6.70 (t, *J* = 7.2 Hz, 1H), 6.61 (d, *J* = 7.9 Hz, 2H), 3.88 (dd, *J* = 13.1, 6.3 Hz, 1H), 3.61 (dd, *J* = 13.2, 7.8 Hz, 1H), 3.32 (dd, *J* = 13.3, 4.5 Hz, 1H), 1.28 (d, *J* = 7.3 Hz, 3H); ^{13}C NMR (75 MHz, $CDCl_3$) δ 203.5, 147.6, 136.2, 133.2, 129.3, 128.7, 128.3, 117.5, 112.9, 46.5, 40.2, 16.0; HRMS (ESI) m/z 240.1386 (M+H$^+$), calc. for $C_{16}H_{18}NO$ 240.1388.

The ee was determined by HPLC analysis: CHIRALPAK IE (4.6 mm i.d. x 250 mm); Hexane/2-propanol = 80/20; flow rate 1.0 mL/min; 25 °C; 254 nm; retention time: 8.2 min (minor) and 12.5 min (major).

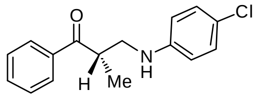

(*S*)-3-((4-chlorophenyl)amino)-2-methyl-1-phenylpropan-1-one (3b): yellow oil; 21.4 mg, 78% yield; 94% ee; $[\alpha]_D^{22}$ +459.4 (*c* 1.0, $CHCl_3$); ^{1}H NMR (300 MHz, $CDCl_3$) δ 7.92 (d, *J* = 7.6 Hz, 2H), 7.56 (d, *J* = 7.1 Hz, 1H), 7.46 (t, *J* = 6.9 Hz, 2H), 7.10 (d, *J* = 7.7 Hz, 2H), 6.52 (d, *J* = 7.8 Hz, 2H), 4.21 – 3.71 (m, 2H), 3.66 – 3.47 (m, 1H), 3.42 – 3.17 (m, 1H), 1.27 (d, *J* = 6.2 Hz, 3H); ^{13}C NMR (75 MHz, $CDCl_3$) δ 203.3, 146.3, 136.2, 133.3, 129.1, 128.7, 128.3, 121.9, 113.9, 46.5, 40.1, 16.0; HRMS (ESI) m/z 274.1003 (M+H$^+$), calc. for $C_{16}H_{17}NOCl$ 274.0999.

The ee was determined by HPLC analysis: CHIRALPAK IF (4.6 mm i.d. x 250 mm); Hexane/2-propanol = 90/10; flow rate 1.0 mL/min; 25 °C; 254 nm; retention time: 17.4 min (minor) and 23.0 min (major).

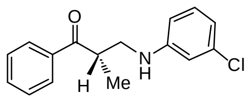

(*S*)-3-((3-chlorophenyl)amino)-2-methyl-1-phenylpropan-1-one (3c): yellow oil; 19.4 mg, 71% yield; 94% ee; $[\alpha]_D^{22}$ +769.4 (*c* 1.0, $CHCl_3$); ^{1}H NMR (300 MHz, $CDCl_3$) δ 7.93 (d, *J* = 7.3 Hz, 2H), 7.58 (t, *J* = 7.2 Hz, 1H), 7.46 (t, *J* = 7.4 Hz, 2H), 7.05 (t, *J* = 7.8 Hz, 1H), 6.65 (d, *J* = 7.8 Hz, 1H), 6.57 (s, 1H), 6.45 (d, *J* = 8.0 Hz, 1H), 4.17 (s, 1H), 3.93 – 3.76 (m, 1H), 3.65 – 3.51 (m, 1H), 3.36 – 3.22 (m, 1H), 1.27 (d, *J* = 6.9 Hz, 3H); ^{13}C NMR (75 MHz, $CDCl_3$) δ 203.2, 148.9, 136.1, 135.0, 133.3, 130.2, 128.8, 128.3, 117.3, 112.3, 111.2, 46.2, 40.1, 16.0; HRMS (ESI) m/z 274.0997 (M+H$^+$), calc. for $C_{16}H_{17}NOCl$ 274.0999.

The ee was determined by HPLC analysis: CHIRALPAK IF (4.6 mm i.d. x 250 mm); Hexane/2-propanol = 85/15; flow rate 1.0 mL/min; 25 ^{o}C; 254 nm; retention time: 8.3 min (minor) and 9.6 min (major).

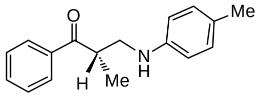

(*S*)-2-methyl-1-phenyl-3-(p-tolylamino)propan-1-one (3d): yellow oil; 19.2 mg, 76% yield; 95% ee; $[\alpha]_D^{22}$ +264.0 (*c* 1.0, $CHCl_3$); ^{1}H NMR (300 MHz, $CDCl_3$) δ 7.93 (d, *J* = 7.1 Hz, 2H), 7.62 – 7.51 (m, 1H), 7.50 – 7.39 (m, 2H), 6.98 (d, *J* = 7.0 Hz, 2H), 6.54 (d, *J* = 7.0 Hz, 2H), 3.94 – 3.80 (d, *J* = 6.3 Hz, 1H), 3.63 – 3.50 (m, 1H), 3.36 – 3.20 (m, 1H), 2.24 (s, 3H), 1.27 (d, *J* = 5.7 Hz, 3H); ^{13}C NMR (75 MHz, $CDCl_3$) δ 203.6, 145.4, 136.3, 133.2, 129.8, 128.7, 128.3, 126.7, 113.1, 47.0, 40.3, 20.3, 16.0; HRMS (ESI) m/z 276.1366 (M+Na^{+}), calc. for $C_{17}H_{19}NONa$ 276.1364.

The ee was determined by HPLC analysis: CHIRALPAK IE (4.6 mm i.d. x 250 mm); Hexane/2-propanol = 90/10; flow rate 1.0 mL/min; 25 ^{o}C; 254 nm; retention time: 12.2 min (minor) and 18.9 min (major).

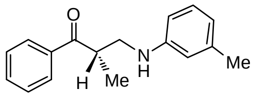

(*S*)-2-methyl-1-phenyl-3-(m-tolylamino)propan-1-one (3e): yellow oil; 20.3 mg, 80% yield; 95% ee; $[\alpha]_D^{22}$ +430.7 (*c* 1.0, $CHCl_3$); ^{1}H NMR (300 MHz, $CDCl_3$) δ 7.94 (d, *J* = 7.7 Hz, 2H), 7.57 (t, *J* = 7.1 Hz, 1H), 7.46 (t, *J* = 7.4 Hz, 2H), 7.06 (t, *J* = 7.7 Hz, 1H), 6.53 (d, *J* = 7.3 Hz, 1H), 6.47 – 6.37 (m, 2H), 3.86 (dd, *J* = 13.3, 6.5 Hz, 1H), 3.60 (dd, *J* = 13.0, 7.6 Hz, 1H), 3.30 (dd, *J* = 13.0, 4.7 Hz, 1H), 2.27 (s, 3H), 1.28 (d, *J* = 7.0 Hz, 3H); ^{13}C NMR (75 MHz, $CDCl_3$) δ 203.5, 147.7, 139.1, 136.3, 133.2, 129.2, 128.7, 128.3, 118.4, 113.7, 109.9, 46.5, 40.3, 21.6, 16.0; HRMS (ESI) m/z 276.1358 (M+Na^{+}), calc. for $C_{17}H_{19}NONa$ 276.1364.

The ee was determined by HPLC analysis: CHIRALPAK IE (4.6 mm i.d. x 250 mm); Hexane/2-propanol = 90/10; flow rate 1.0 mL/min; 25 ^{o}C; 254 nm; retention time: 10.1 min (minor) and 12.7 min (major).

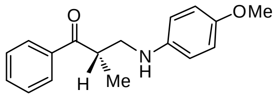

(*S*)-3-((4-methoxyphenyl)amino)-2-methyl-1-phenylpropan-1-one (3f): yellow oil; 18.6 mg, 69% yield; 94% ee; $[\alpha]_D^{22}$ +300.0 (*c* 1.0, $CHCl_3$); ^{1}H NMR (300 MHz, $CDCl_3$) δ 7.91 (d, *J* = 7.5 Hz, 2H), 7.56 (t, *J* = 7.1 Hz, 1H), 7.45 (t, *J* = 7.3 Hz, 2H), 6.77 (d, *J* = 8.1 Hz, 2H), 6.59 (d, *J* = 8.1 Hz, 2H), 3.84 (dd, *J* = 13.4, 7.0 Hz, 1H), 3.74 (s, 3H), 3.62 – 3.43 (m, 1H), 3.24 (dd, *J* = 13.0, 4.3 Hz, 1H), 1.25 (d, *J* = 6.4 Hz, 3H); ^{13}C NMR (75 MHz, $CDCl_3$) δ 203.6, 152.2, 141.9, 136.3, 133.2, 128.7, 128.3, 114.9,

114.4, 55.8, 47.7, 40.3, 16.0; HRMS (ESI) m/z 270.1487 ($M+H^+$), calc. for $C_{17}H_{20}NO_2$ 270.1494.

The ee was determined by HPLC analysis: CHIRALPAK IE (4.6 mm i.d. x 250 mm); Hexane/2-propanol = 70/30; flow rate 1.0 mL/min; 25 °C; 254 nm; retention time: 11.4 min (minor) and 17.1 min (major).

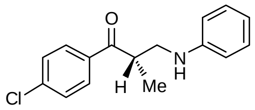

(*S*)-1-(4-chlorophenyl)-2-methyl-3-(phenylamino)propan-1-one (3g): yellow oil; 21.6 mg, 79% yield; 95% ee; $[\alpha]_D^{22}$ +704.8 (*c* 1.0, $CHCl_3$); ^{1}H NMR (300 MHz, $CDCl_3$) δ 7.85 (d, *J* = 7.8 Hz, 2H), 7.42 (d, *J* = 7.8 Hz, 2H), 7.17 (t, *J* = 7.2 Hz, 2H), 6.71 (t, *J* = 7.2 Hz, 1H), 6.60 (d, *J* = 7.7 Hz, 2H), 3.82 (dd, *J* = 13.1, 6.5 Hz, 1H), 3.60 (dd, *J* = 13.3, 7.9 Hz, 1H), 3.32 (dd, *J* = 13.3, 4.3 Hz, 1H), 1.26 (d, *J* = 6.8 Hz, 3H); ^{13}C NMR (75 MHz, $CDCl_3$) δ 202.3, 147.5, 139.7, 134.6, 129.7, 129.3, 129.0, 117.6, 112.8, 46.5, 40.3, 15.9; HRMS (ESI) m/z 274.1004 ($M+H^+$), calc. for $C_{16}H_{17}NOCl$ 274.0999.

The ee was determined by HPLC analysis: CHIRALPAK IF (4.6 mm i.d. x 250 mm); Hexane/2-propanol = 90/10; flow rate 1.0 mL/min; 25 °C; 254 nm; retention time: 12.7 min (minor) and 18.9 min (major).

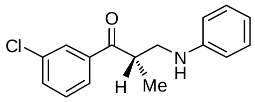

(*S*)-1-(3-chlorophenyl)-2-methyl-3-(phenylamino)propan-1-one (3h): yellow oil; 22.4 mg, 82% yield; 93% ee; $[\alpha]_D^{22}$ +769.4 (*c* 1.0, $CHCl_3$); ^{1}H NMR (300 MHz, $CDCl_3$) δ 7.89 (s, 1H), 7.78 (d, *J* = 7.7 Hz, 1H), 7.53 (d, *J* = 7.9 Hz, 1H), 7.39 (t, *J* = 7.8 Hz, 1H), 7.18 (t, *J* = 7.3 Hz, 2H), 6.71 (t, *J* = 7.0 Hz, 1H), 6.60 (d, *J* = 7.7 Hz, 2H), 3.81 (dd, *J* = 13.0, 6.3 Hz, 1H), 3.67 – 3.53 (m, 1H), 3.32 (dd, *J* = 13.3, 4.2 Hz, 1H), 1.27 (d, *J* = 6.8 Hz, 3H); ^{13}C NMR (75 MHz, $CDCl_3$) δ 202.3, 147.5, 137.9, 135.1, 133.1, 130.0, 129.4, 128.4, 126.4, 117.6, 112.8, 46.5, 40.5, 15.9; HRMS (ESI) m/z 274.0994 ($M+H^+$), calc. for $C_{16}H_{17}NOCl$ 274.0999.

The ee was determined by HPLC analysis: CHIRALPAK IF (4.6 mm i.d. x 250 mm); Hexane/2-propanol = 90/10; flow rate 1.0 mL/min; 25 °C; 254 nm; retention time: 9.4 min (minor) and 16.6 min (major).

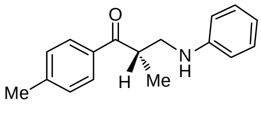

(*S*)-2-methyl-3-(phenylamino)-1-(p-tolyl)propan-1-one (3i): yellow oil; 21.8 mg, 86% yield; 94% ee; $[\alpha]_D^{22}$ +545.4 (*c* 1.0, $CHCl_3$); ^{1}H NMR (300 MHz, $CDCl_3$) δ 7.84 (d, *J* = 7.7 Hz, 2H), 7.25 (d, *J* = 6.6 Hz, 2H), 7.17 (t, *J* = 7.5 Hz, 2H), 6.70 (t, *J* = 7.2 Hz, 1H), 6.60 (d, *J* = 7.9 Hz, 2H), 3.84 (dd, *J* = 13.1, 6.5 Hz, 1H),

3.60 (dd, J = 13.1, 7.5 Hz, 1H), 3.31 (dd, J = 13.2, 4.9 Hz, 1H), 2.41 (s, 3H), 1.27 (d, J = 6.6 Hz, 3H); ^{13}C NMR (75 MHz, $CDCl_3$) δ 203.1, 147.8, 144.1, 133.8, 129.4，129.3, 128.,5, 117.4, 112.8, 46.6, 40.1, 21.6, 16.1; HRMS (ESI) m/z 254.1551 (M+H$^+$), calc. for $C_{17}H_{20}NO$ 254.1545.

The ee was determined by HPLC analysis: CHIRALPAK IF (4.6 mm i.d. x 250 mm); Hexane/2-propanol = 92/8; flow rate 1.0 mL/min; 25 °C; 254 nm; retention time: 13.3 min (minor) and 21.9 min (major).

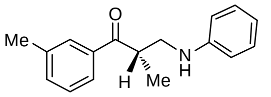

(*S*)-2-methyl-3-(phenylamino)-1-(m-tolyl)propan-1-one (3j): white solid, Mp 47.3−47.5 °C; 19.2 mg, 76% yield; 95% ee; $[\alpha]_D^{22}$ +282.7 (*c* 1.0, $CHCl_3$); ^{1}H NMR (300 MHz, $CDCl_3$) δ 7.77 – 7.68 (m, 2H), 7.42 – 7.29 (m, 2H), 7.17 (t, J = 7.4 Hz, 2H), 6.71 (t, J = 7.1 Hz, 1H), 6.61 (d, J = 7.9 Hz, 2H), 3.87 (dd, J = 13.0, 6.5 Hz, 1H), 3.60 (dd, J = 13.2, 7.8 Hz, 1H), 3.32 (dd, J = 13.2, 4.6 Hz, 1H), 2.39 (s, 3H), 1.27 (d, J = 6.4 Hz, 3H); ^{13}C NMR (75 MHz, $CDCl_3$) δ 203.8, 147.7, 138.5, 136.4, 134.0, 129.3, 128.9, 128.5, 125.5, 117.4, 112.8, 46.6, 40.2, 21.3, 16.0; HRMS (ESI) m/z 254.1548 (M+H$^+$), calc. for $C_{17}H_{20}NO$ 254.1545.

The ee was determined by HPLC analysis: CHIRALPAK IF (4.6 mm i.d. x 250 mm); Hexane/2-propanol = 90/10; flow rate 1.0 mL/min; 25 °C; 254 nm; retention time: 9.4 min (minor) and 15.3 min (major).

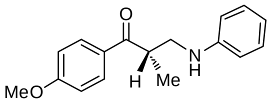

(*S*)-1-(4-methoxyphenyl)-2-methyl-3-(phenylamino)propan-1-one (3k): white solid, Mp 79.7−78.5 °C; 20.2 mg (0.1 mmol), 75% yield; 93% ee; $[\alpha]_D^{22}$ +716.1 (*c* 1.0, $CHCl_3$); ^{1}H NMR (300 MHz, $CDCl_3$) δ 7.92 (d, J = 8.3 Hz, 2H), 7.17 (t, J = 7.5 Hz, 2H), 6.92 (d, J = 8.2 Hz, 2H), 6.69 (t, J = 7.2 Hz, 1H), 6.60 (d, J = 7.9 Hz, 2H), 3.90 – 3.76 (m, 4H), 3.59 (dd, J = 13.2, 7.7 Hz, 1H), 3.30 (dd, J = 13.3, 4.9 Hz, 1H), 1.26 (d, J = 6.7 Hz, 3H); ^{13}C NMR (75 MHz, $CDCl_3$) δ 201.9, 163.6, 147.8, 130.7, 129.3, 117.4, 113.8, 112.8, 55.5, 46.7, 39.8, 16.1; HRMS (ESI) m/z 270.1490 (M+H$^+$), calc. for $C_{17}H_{20}NO_2$ 270.1494.

The ee was determined by HPLC analysis: CHIRALPAK IF (4.6 mm i.d. x 250 mm); Hexane/2-propanol = 85/15; flow rate 1.0 mL/min; 25 °C; 254 nm; retention time: 14.4 min (minor) and 23.4 min (major).

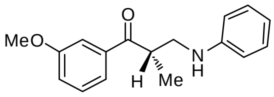

(*S*)-1-(3-methoxyphenyl)-2-methyl-3-(phenylamino)propan-1-one (3l): yellow oil; 18.3 mg, 68% yield; 94% ee; $[\alpha]_D^{22}$ +780.8 (*c* 1.0, $CHCl_3$); ^1H NMR (300 MHz, $CDCl_3$) δ 7.54 – 7.42 (m, 2H), 7.35 (t, *J* = 7.9 Hz, 1H), 7.23 – 7.04 (m, 3H), 6.70 (t, *J* = 7.3 Hz, 1H), 6.61 (d, *J* = 7.8 Hz, 2H), 3.92 – 3.76 (m, 4H), 3.60 (dd, *J* = 13.4, 8.0 Hz, 1H), 3.32 (dd, *J* = 13.1, 4.5 Hz, 1H), 1.27 (d, *J* = 6.8 Hz, 3H); ^{13}C NMR (75 MHz, $CDCl_3$) δ 203.4, 159.9, 147.7, 137.8, 129.6, 129.3, 120.9, 119.8, 117.5, 112.8, 112.5, 55., 46.7, 40.4, 16.0; HRMS (ESI) m/z 270.1487 ($M+H^+$), calc. for $C_{17}H_{20}NO_2$ 270.1494.

The ee was determined by HPLC analysis: CHIRALPAK IF (4.6 mm i.d. x 250 mm); Hexane/2-propanol = 85/15; flow rate 1.0 mL/min; 25 °C; 254 nm; retention time: 9.4 min (minor) and 17.3 min (major).

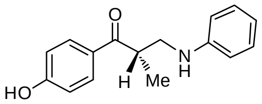

(*S*)-1-(4-hydroxyphenyl)-2-methyl-3-(phenylamino)propan-1-one (3m): white solid, Mp 58.4−58.9 °C; 17.4 mg, 68% yield; 88% ee; $[\alpha]_D^{22}$ +582.8 (*c* 1.0, $CHCl_3$); ^1H NMR (300 MHz, $CDCl_3$) δ 7.85 (d, *J* = 8.6 Hz, 2H), 7.16 (t, *J* = 7.8 Hz, 2H), 6.85 (d, *J* = 8.6 Hz, 2H), 6.70 (t, *J* = 7.3 Hz, 1H), 6.60 (d, *J* = 7.7 Hz, 2H), 3.89 – 3.73 (m, 1H), 3.58 (dd, *J* = 13.2, 7.8 Hz, 1H), 3.29 (dd, *J* = 13.2, 5.0 Hz, 1H), 1.26 (d, *J* = 6.9 Hz, 3H); ^{13}C NMR (75 MHz, $CDCl_3$) δ 202.5, 160.6, 147.7, 131.1, 129.3, 129.1, 117.6, 115.5, 113.0, 46.8, 39.8, 16.2; HRMS (ESI) m/z 256.1329 ($M+H^+$), calc. for $C_{16}H_{18}NO_2$ 256.1338.

The ee was determined by HPLC analysis: CHIRALPAK IF (4.6 mm i.d. x 250 mm); Hexane/2-propanol = 90/10; flow rate 1.0 mL/min; 25 °C; 254 nm; retention time: 14.4 min (minor) and 22.9 min (major).

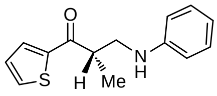

(*S*)-2-methyl-3-(phenylamino)-1-(thiophen-2-yl)propan-1-one (3n): yellow oil; 14.7 mg, 60% yield; 86% ee; $[\alpha]_D^{22}$ +474.8 (*c* 1.0, $CHCl_3$); ^1H NMR (300 MHz, $CDCl_3$) δ 7.76 – 7.58 (m, 2H), 7.17 (t, *J* = 7.9 Hz, 2H), 7.17 – 7.13 (m, 1H), 6.70 (t, *J* = 7.3 Hz, 1H), 6.60 (d, *J* = 7.7 Hz, 2H), 3.78 – 3.65 (m, 1H), 3.59 (dd, *J* = 13.2, 7.9 Hz, 1H), 3.31 (dd, *J* = 13.2, 5.0 Hz, 1H), 1.31 (d, *J* = 6.9 Hz, 3H); ^{13}C NMR (75 MHz, $CDCl_3$) δ 196.2, 147.6, 143.9, 134.2, 132.2, 129.3, 128.2, 117.5, 112.8, 46.7, 42.0, 16.2; HRMS (ESI) m/z 246.0948 ($M+H^+$), calc. for $C_{14}H_{16}NOS$ 246.0953.

The ee was determined by HPLC analysis: CHIRALPAK IE (4.6 mm i.d. x 250 mm); Hexane/2-propanol = 75/25; flow rate 1.0 mL/min; 25 °C; 254 nm; retention time: 7.5 min (minor) and 13.8 min (major).

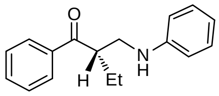

(*S*)-1-phenyl-2-((phenylamino)methyl)butan-1-one (3o): yellow oil; 17.2 mg, 68% yield; 90% ee; $[\alpha]_D^{22}$ +327.4 (*c* 1.0, $CHCl_3$); ^1H NMR (300 MHz, $CDCl_3$) δ 7.91 (d, *J* = 7.4 Hz, 2H), 7.56 (t, *J* = 6.9 Hz, 1H), 7.45 (t, *J* = 7.3 Hz, 2H), 7.16 (t, *J* = 7.3 Hz, 2H), 6.70 (t, *J* = 7.1 Hz, 1H), 6.59 (d, *J* = 7.7 Hz, 2H), 3.84 – 3.70 (s, 1H), 3.67 – 3.52 (m, 1H), 3.45 – 3.30 (m, 1H), 1.86 (dt, *J* = 13.9, 7.1 Hz, 1H), 1.67 (dt, *J* = 13.7, 6.8 Hz, 1H), 0.96 (t, *J* = 7.2 Hz, 3H); ^{13}C NMR (75 MHz, $CDCl_3$) δ 203.6, 147.7, 137.2, 133.2, 129.3, 128.7, 128.2, 117.5, 112.9, 47.0, 44.9, 23.8, 11.7; HRMS (ESI) m/z 254.1552 ($M+H^+$), calc. for $C_{17}H_{20}NO$ 254.1545.

The ee was determined by HPLC analysis: CHIRALPAK IF (4.6 mm i.d. x 250 mm); Hexane/2-propanol = 94/6; flow rate 1.0 mL/min; 25 °C; 254 nm; retention time: 11.2 min (major) and 12.5 min (minor).

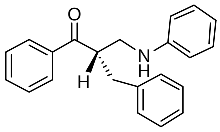

(*S*)-2-benzyl-1-phenyl-3-(phenylamino)propan-1-one (3p): white solid, Mp 43.8−44.2 °C; 21.1 mg, 67% yield; 94% ee; $[\alpha]_D^{22}$ +50.0 (*c* 1.0, $CHCl_3$); ^1H NMR (300 MHz, $CDCl_3$) δ 7.88 (d, *J* = 7.3 Hz, 2H), 7.55 (t, *J* = 7.3 Hz, 1H), 7.42 (t, *J* = 7.6 Hz, 2H), 7.35 – 7.26 (d, *J* = 7.7 Hz, 2H), 7.25 – 7.18 (m, 3H), 7.13 (t, *J* = 7.9 Hz, 2H), 6.69 (t, *J* = 7.3 Hz, 1H), 6.46 (d, *J* = 7.8 Hz, 2H), 4.16 – 4.03 (m, 1H), 3.59 (dd, *J* = 13.3, 7.6 Hz, 1H), 3.36 (dd, *J* = 13.3, 4.4 Hz, 1H), 3.15 (dd, *J* = 13.7, 6.3 Hz, 1H), 2.88 (dd, *J* = 13.7, 8.0 Hz, 1H); ^{13}C NMR (75 MHz, $CDCl_3$) δ 203.0, 147.4, 138.8, 136.8, 133.3, 129.2, 129.0, 128.7, 128.6, 128.3, 126.6, 117.6, 112.9, 47.5, 44.9, 36.5; HRMS (ESI) m/z 316.1711 ($M+H^+$), calc. for $C_{22}H_{22}NO$ 316.1701.

The ee was determined by HPLC analysis: CHIRALPAK IF (4.6 mm i.d. x 250 mm); Hexane/2-propanol = 90/10; flow rate 1.0 mL/min; 25 °C; 254 nm; retention time: 13.4 min (major) and 15.3 min (minor).

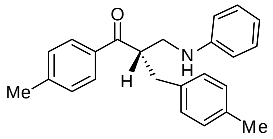

(*S*)-2-(4-methylbenzyl)-3-(phenylamino)-1-(p-tolyl)propan-1-one (3q): yellow oil; 25.1 mg, 73% yield; 90% ee; $[\alpha]_D^{22}$ –66.0 (*c* 1.0, $CHCl_3$); ^1H NMR (300 MHz, $CDCl_3$) δ 7.80 (d, *J* = 8.2 Hz, 2H), 7.22 (d, *J* = 8.0 Hz, 2H), 7.16 – 7.06 (m, 6H), 6.69 (t, *J* = 7.3 Hz, 1H), 6.47 (d, *J* = 7.7 Hz, 2H),

4.11 – 3.97 (m, 1H), 3.55 (dd, J = 13.2, 7.5 Hz, 1H), 3.32 (dd, J = 13.2, 4.4 Hz, 1H), 3.10 (dd, J = 13.8, 6.0 Hz, 1H), 2.83 (dd, J = 13.8, 8.2 Hz, 1H), 2.40 (s, 3H), 2.32 (s, 3H); ^{13}C NMR (75 MHz, $CDCl_3$) δ 202.6, 147.5, 144.1, 136.0, 135.8, 134.3, 129.4, 129.2, 129.1, 128.8, 128.5, 117.6, 113.0, 47.4, 44.8, 36.1, 21.6, 21.0; HRMS (ESI) m/z 344.2005 (M+H$^+$), calc. for $C_{24}H_{25}NO$ 344.2014.

The ee was determined by HPLC analysis: CHIRALPAK IC (4.6 mm i.d. x 250 mm); Hexane/2-propanol = 96/4; flow rate 1.0 mL/min; 25 °C; 254 nm; retention time: 15.8 min (minor) and 15.2 min (major).

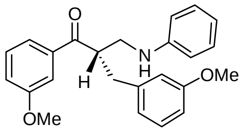

(*S*)-2-(3-methoxybenzyl)-1-(3-methoxyphenyl)-3-(phenylamino)propan-1-one (3r): yellow oil; 22.5 mg, 60% yield; 93% ee; $[\alpha]_D^{22}$ –74.0 (*c* 1.0, $CHCl_3$); ^{1}H NMR (300 MHz, $CDCl_3$) δ 7.44 (d, J = 7.7 Hz, 1H), 7.40 – 7.36 (m, 1H), 7.31 (t, J = 7.9 Hz, 1H), 7.19 (t, J = 7.9 Hz, 1H), 7.16 – 7.04 (m, 3H), 6.80 (d, J = 7.5 Hz, 1H), 6.78 – 6.72 (m, 2H), 6.69 (t, J = 7.3 Hz, 1H), 6.49 (d, J = 7.7 Hz, 2H), 4.13 – 4.01 (m, 1H), 3.78 (s, 3H), 3.74 (s, 3H), 3.57 (dd, J = 13.3, 7.6 Hz, 1H), 3.35 (dd, J = 13.3, 4.5 Hz, 1H), 3.11 (dd, J = 13.8, 6.4 Hz, 1H), 2.85 (dd, J = 13.8, 7.9 Hz, 1H); ^{13}C NMR (75 MHz, $CDCl_3$) δ 202.9, 159.9, 159.7, 147.3, 140.4, 138.3, 129.6, 129.5, 129.3, 121.3, 120.9, 120.0, 117.7, 114.7, 113.0, 112.4, 112.0, 55.3, 55.1, 47.5, 45.1, 36.6; HRMS (ESI) m/z 376.1918 (M+H$^+$), calc. for $C_{24}H_{26}NO_3$ 376.1913.

The ee was determined by HPLC analysis: CHIRALPAK IC (4.6 mm i.d. x 250 mm); Hexane/2-propanol = 95/5; flow rate 1.0 mL/min; 25 °C; 254 nm; retention time: 29.3 min (minor) and 32.1 min (major).

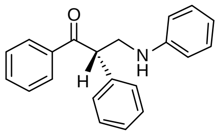

(*S*)-1,2-diphenyl-3-(phenylamino)propan-1-one (3s): white solid, Mp 70.4−70.8 °C; 16.6 mg, 55% yield; 87% ee; $[\alpha]_D^{22}$ –759.8 (*c* 1.0, $CHCl_3$); ^{1}H NMR (300 MHz, $CDCl_3$) δ 7.90 (d, J = 7.2 Hz, 2H), 7.46 (t, J = 7.4 Hz, 1H), 7.40 – 7.26 (m, 7H), 7.23 – 7.14(m, 2H), 6.74 (t, J = 7.3 Hz, 1H), 6.67 (d, J = 7.7 Hz, 2H), 4.94 (dd, J = 8.1, 5.7 Hz, 1H), 3.99 (dd, J = 13.8, 8.2 Hz, 1H), 3.55 (dd, J = 13.8, 5.7 Hz, 1H); ^{13}C NMR (75 MHz, $CDCl_3$) δ 199.1, 147.0, 137.3, 136.4, 133.1, 129.4, 129.3, 128.8, 128.5, 128.2, 127.6, 118.0, 113., 52.9, 47.3; HRMS (ESI) m/z 302.1537 (M+H$^+$), calc. for $C_{21}H_{20}NO$ 302.1545.

The ee was determined by HPLC analysis: CHIRALPAK IE (4.6 mm i.d. x 250 mm); Hexane/2-propanol = 95/5; flow rate 1.0 mL/min; 25 °C; 254 nm; retention time: 13.2 min (major) and 16.5 min (minor).

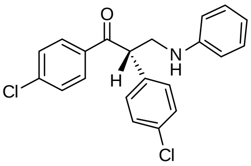

(*S*)-1,2-bis(4-chlorophenyl)-3-(phenylamino)propan-1-one (3t): yellow oil; 22.6 mg, 61% yield; 90% ee; $[\alpha]_D^{22}$ –202.62 (*c* 1.0, $CHCl_3$); ^1H NMR (300 MHz, $CDCl_3$) δ 7.79 (d, *J* = 8.6 Hz, 2H), 7.37 – 7.27 (m, 4H), 7.25 – 7.14 (m, 4H), 6.77 (t, *J* = 7.3 Hz, 1H), 6.66 (d, *J* = 7.9 Hz, 2H), 4.87 (dd, *J* = 8.0, 5.8 Hz, 1H), 3.96 (dd, *J* = 13.9, 8.2 Hz, 1H), 3.50 (dd, *J* = 13.9, 5.6 Hz, 1H); ^{13}C NMR (75 MHz, $CDCl_3$) δ 197.5, 146.3, 139.9, 135.4, 134.3, 133.8, 130.2, 129.6, 129.5, 129.4, 129.0, 118.5, 113.5, 52.0, 47.3; HRMS (ESI) m/z 370.0765 ($M+H^+$), calc. for $C_{21}H_{18}NOCl_2$ 370.0765.

The ee was determined by HPLC analysis: CHIRALPAK IE (4.6 mm i.d. x 250 mm); Hexane/2-propanol = 90/10; flow rate 1.0 mL/min; 25 °C; 254 nm; retention time: 8.1 min (major) and 9.4 min (minor).

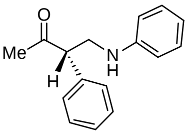

(*S*)-3-phenyl-4-(phenylamino)butan-2-one (3u): yellow oil; 15.6 mg, 65% yield; 90% ee; $[\alpha]_D^{22}$ –46.0 (*c* 1.0, $CHCl_3$); ^1H NMR (300 MHz, $CDCl_3$) δ 7.45 – 7.29 (m, 3H), 7.26 – 7.11 (m, 4H), 6.72 (t, *J* = 7.3 Hz, 1H), 6.60 (d, *J* = 7.9 Hz, 2H), 4.06 – 3.95 (m, 1H), 3.84 (dd, *J* = 13.6, 8.3 Hz, 1H), 3.37 (dd, *J* = 13.6, 5.7 Hz, 1H), 2.06 (s, 3H); ^{13}C NMR (75 MHz, $CDCl_3$) δ 207.9, 147.3, 136.7, 129.4, 129.3, 128.3, 127.9, 117.7, 113.1, 58.4, 45.6, 29.5; HRMS (ESI) m/z 240.1392 ($M+H^+$), calc. for $C_{16}H_{18}NO$ 240.1388.

The ee was determined by HPLC analysis: CHIRALPAK IE (4.6 mm i.d. x 250 mm); Hexane/2-propanol = 95/5; flow rate 1.0 mL/min; 25 °C; 254 nm; retention time: 10.2 min (minor) and 13.7 min (major).

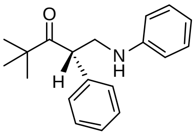

(*S*)-4,4-dimethyl-2-phenyl-1-(phenylamino)pentan-3-one (3v): white solid, Mp 66.9–67.8 °C; 15.5 mg, 55% yield; 88% ee; $[\alpha]_D^{22}$ –331.23 (*c* 1.0, $CHCl_3$); ^1H NMR (300 MHz, $CDCl_3$) δ 7.36 – 7.28 (m,, 7.8 Hz, 4H), 7.26 – 7.23 (m, 1H), 7.18 (t, *J* = 7.9 Hz, 2H), 6.71 (t, *J* = 7.3 Hz, 1H), 6.59 (d, *J* = 7.7 Hz, 2H), 4.52 (dd, *J* = 8.4, 6.0 Hz, 1H), 3.75 (dd, *J* = 13.8, 8.5 Hz, 1H), 3.38 (dd, *J* = 13.9, 5.8 Hz, 1H), 1.01 (s, 9H); ^{13}C NMR (75 MHz, $CDCl_3$) δ 212.9, 149.1, 134.9, 129.5, 129.1, 128.3, 126.6, 117.3,

112.5, 44.6, 43.2, 30.7, 26.3; HRMS (ESI) m/z 282.1859 (M+H$^+$), calc. for $C_{19}H_{24}NO$ 282.1858.

The ee was determined by HPLC analysis: CELLULOSE–1 (4.6 mm i.d. x 250 mm); Hexane/2-propanol = 98/2; flow rate 1.0 mL/min; 25 ^{o}C; 254 nm; retention time: 8.8 min (major) and 10.0 min (minor).

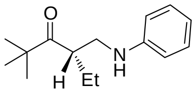

(*S*)-2,2-dimethyl-4-((phenylamino)methyl)hexan-3-one (3w): yellow oil; 14.2 mg, 61% yield; 93% ee; $[\alpha]_D^{22}$ +740.8 (*c* 1.0, $CHCl_3$); ^{1}H NMR (300 MHz, $CDCl_3$) δ 7.22 – 7.11 (m, 2H), 6.69 (t, *J* = 7.3 Hz, 1H), 6.58 (d, *J* = 7.7 Hz, 2H), 3.42 (dd, *J* = 12.4, 7.0 Hz, 1H), 3.35 – 3.14 (m, 2H), 1.77 – 1.43 (m, 2H), 1.12 (s, 9H), 0.95 (t, *J* = 7.5 Hz, 3H); ^{13}C NMR (75 MHz, $CDCl_3$) δ 218.7, 147.8, 129.3, 117.3, 112.6, 46.0, 45.2, 44.8, 26.1, 23.9, 12.0; HRMS (ESI) m/z 234.1858 (M+H$^+$), calc. for $C_{15}H_{24}NO$ 234.1858.

The ee was determined by HPLC analysis: CELLULOSE–2 (4.6 mm i.d. x 250 mm); Hexane/2-propanol = 98/2; flow rate 1.0 mL/min; 25 ^{o}C; 254 nm; retention time: 9.8 min (major) and 11.5 min (minor).

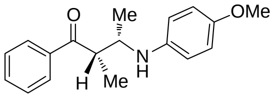

(2*S*,3*S*)-3-((4-methoxyphenyl)amino)-2-methyl-1-phenylbutan-1-one (3x): white solid, Mp 89.8−90.2 ^{o}C; 14.2 mg, 50% yield; 96% ee; >20:1 dr; $[\alpha]_D^{22}$ +410.7 (*c* 1.0, $CHCl_3$); ^{1}H NMR (300 MHz, $CDCl_3$) δ 8.10 – 7.86 (m, 2H), 7.68 – 7.54 (m, 1H), 7.50 – 7.37 (m, 2H), 6.88 – 6.70 (m, 2H), 6.62 (d, *J* = 7.0 Hz, 2H), 3.92 – 2.80 (m, 1H), 3.75 (s, 3H), 1.27 – 1.18 (m, 3H), 1.17 – 1.07 (m, 3H); ^{13}C NMR (75 MHz, $CDCl_3$) δ 204.1, 152.4, 141.2, 137.2, 133.0, 128.7, 128.1, 115.7, 115.0, 55.8, 52.2, 44.8, 17.5, 12.7; HRMS (ESI) m/z 306.1473 (M+Na$^+$), calc. for $C_{18}H_{21}NO_2Na$ 306.1470.

The ee was determined by HPLC analysis: AMYLOSE–2 and CHIRALPAK IE (4.6 mm i.d. x 250 mm); Hexane/2-propanol = 90/10; flow rate 1.0 mL/min; 25 ^{o}C; 254 nm; retention time: 24.8 min (major) and 32.7 min (minor).

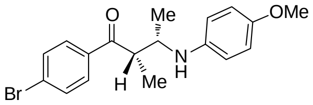

(2*S*,3*S*)-1-(4-bromophenyl)-3-((4-methoxyphenyl)amino)-2-methylbutan-1-one (3y): yellow oil; 16.7 mg, 46% yield; 96% ee; >20:1 dr; $[\alpha]_D^{22}$ +380.2 (*c* 1.0, $CHCl_3$); ^{1}H NMR (300 MHz, $CDCl_3$) δ 7.79 (d, *J* = 8.5 Hz, 2H), 7.61 (d, *J* = 8.5 Hz, 2H), 6.78 (d, *J* = 8.8 Hz, 2H), 6.65 (d, *J* = 8.8 Hz, 2H), 3.89 – 3.77 (m, 1H), 3.77 – 3.65 (m, 4H), 1.20 (d, *J* = 6.9 Hz, 3H), 1.14 (d, *J* = 6.5 Hz, 3H); ^{13}C NMR (75

MHz, $CDCl_3$) δ 202.9, 152.9, 135.8, 132.0, 129.7, 128.2, 116.3, 114.9, 55.7, 52.8, 44.8, 17.2, 12.7; HRMS (ESI) m/z 384.0566 ($M+Na^+$), calc. for $C_{18}H_{20}NO_2NaBr$ 384.0575.

The ee was determined by HPLC analysis: AMYLOSE-2 and CHIRALPAK IE (4.6 mm i.d. x 250 mm); Hexane/2-propanol = 90/10; flow rate 1.0 mL/min; 25 °C; 254 nm; retention time: 28.2 min (major) and 41.5 min (minor).

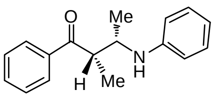

3z

(2*S*,3*S*)-2-methyl-1-phenyl-3-(phenylamino)butan-1-one (3z): yellow oil; 12.9 mg, 51% yield; 97% ee; >20:1 dr; $[\alpha]_D^{22}$ +320.2 (*c* 1.0, $CHCl_3$); ^1H NMR (300 MHz, $CDCl_3$) δ 7.96 (d, *J* = 7.7 Hz, 2H), 7.64 – 7.55 (m, 1H), 7.50 (t, *J* = 7.5 Hz, 2H), 7.18 (t, *J* = 7.3 Hz, 2H), 6.77 – 6.58 (m, 3H), 4.17 (s, 1H), 3.99 – 3.87 (m, 1H), 3.86 – 3.72 (m, 1H), 1.25 (d, *J* = 6.9 Hz, 3H), 1.17 (d, *J* = 6.3 Hz, 3H); ^{13}C NMR (75 MHz, $CDCl_3$) δ 204.2, 147.1, 137.2, 133.1, 129.4, 128.8, 128.2, 117.5, 113.7, 50.9, 44.6, 17.7, 13.3; HRMS (ESI) m/z 254.1550 ($M+H^+$), calc. for $C_{17}H_{20}NO$ 254.1545.

The ee was determined by HPLC analysis: AMYLOSE–2 and CHIRALPAK IE (4.6 mm i.d. x 250 mm); Hexane/2-propanol = 99/1; flow rate 1.2 mL/min; 25 °C; 254 nm; retention time: 31.6 min (major) and 41.1 min (minor).

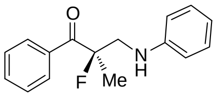

(*R*)-2-fluoro-2-methyl-1-phenyl-3-(phenylamino)propan-1-one (6a): white solid, Mp 158.9−159.9 °C; 19.3 mg, 75% yield; 90% ee; $[\alpha]_D^{22}$ –45.0 (*c* 1.0, $CHCl_3$); ^1H NMR (300 MHz, $CDCl_3$) δ 8.02 (d, *J* = 8.1 Hz, 2H), 7.57 (t, *J* = 7.3 Hz, 1H), 7.44 (t, *J* = 7.6 Hz, 2H), 7.17 (t, *J* = 7.8 Hz, 2H), 6.80 – 6.61 (m, 3H), 3.87 (dd, *J* = 24.8, 13.9 Hz, 1H), 3.60 (dd, *J* = 16.6, 14.0 Hz, 1H), 1.75 (d, *J* = 21.7 Hz, 3H); ^{13}C NMR (75 MHz, $CDCl_3$) δ 201.2 (d, $J_{F–C}$ = 26.4 Hz), 147.7, 135.0 (d, $J_{F–C}$ = 4.0 Hz), 133.2, 129.7 (d, $J_{F–C}$ = 8.0 Hz), 129.2, 128.3 (d, $J_{F–C}$ = 1.1 Hz), 118.2, 113.4, 102.5 (d, $J_{F–C}$ = 187.0 Hz), 51.1 (d, $J_{F–C}$ = 22.1 Hz), 22.5 (d, $J_{F–C}$ = 23.5 Hz); ^{19}F NMR (376 MHz, $CDCl_3$) δ −156.5; HRMS (ESI) m/z 258.1303 ($M+H^+$), calc. for $C_{16}H_{17}NOF$ 258.1294.

The ee was determined by HPLC analysis: CHIRALPAK IE (4.6 mm i.d. x 250 mm); Hexane/2-propanol = 99/1; flow rate 1.0 mL/min; 25 °C; 254 nm; retention time: 15.2 min (major) and 18.3 min (minor).

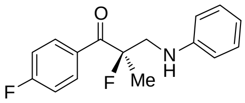

(*R*)-2-fluoro-1-(4-fluorophenyl)-2-methyl-3-(phenylamino)propan-1-one (6b): yellow oil; 17.9 mg, 65% yield; 86% ee; $[\alpha]_D^{22}$ −110.6 (*c* 1.0, $CHCl_3$); ^1H NMR (300 MHz, $CDCl_3$) δ 8.15 – 8.00 (m, 2H), 7.22 – 7.14 (m, 2H), 7.11 (t,

J = 8.7 Hz, 2H), 6.74 (t, J = 7.3 Hz, 1H), 6.67 (d, J = 7.9 Hz, 2H), 4.13 – 3.74 (m, 2H), 3.59 (dd, J = 16.3, 14.0 Hz, 1H), 1.73 (d, J = 21.8 Hz, 3H); ^{13}C NMR (75 MHz, $CDCl_3$) δ 199.5 (d, $J_{F–C}$ = 26.2 Hz), 165.7 (d, $J_{F–C}$ = 255.8 Hz), 147.7, 132.7 (t, $J_{F–C}$ = 9.1 Hz), 131.2 (t, $J_{F–C}$ = 3.6 Hz), 129.2, 118.2, 115.5 (dd, $J_{F–C}$ = 21.7, 1.2 Hz), 113.3, 102.8 (d, $J_{F–C}$ = 186.9 Hz), 51.1 (d, $J_{F–C}$ = 22.0 Hz), 22.4 (d, $J_{F–C}$ = 23.4 Hz); ^{19}F NMR (376 MHz, $CDCl_3$) δ −104.3, −156.2; HRMS (ESI) m/z 276.1196 (M+H$^+$), calc. for $C_{16}H_{16}NOF_2$ 276.1200.

The ee was determined by HPLC analysis: CELLULOSE–4 (4.6 mm i.d. x 250 mm); Hexane/2-propanol = 95/5; flow rate 1.0 mL/min; 25 ^{o}C; 254 nm; retention time: 6.0 min (minor) and 6.9 min (major).

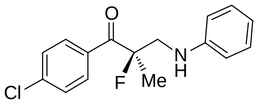

(*R*)-1-(4-chlorophenyl)-2-fluoro-2-methyl-3-(phenylamino)propan-1-one (6c): white solid, Mp 49.8−50.5 ^{o}C; 17.5 mg, 60% yield; 86% ee; $[\alpha]_D^{22}$ +50 (*c* 1.0, $CHCl_3$); ^{1}H NMR (300 MHz, $CDCl_3$) δ 7.96 (d, J = 7.9 Hz, 2H), 7.41 (d, J = 8.6 Hz, 2H), 7.17 (t, J = 7.9 Hz, 2H), 6.74 (t, J = 7.3 Hz, 1H), 6.67 (d, J = 7.8 Hz, 2H), 3.86 (dd, J = 25.8, 14.0 Hz, 1H), 3.58 (dd, J = 16.2, 14.1 Hz, 1H), 1.72 (d, J = 21.8 Hz, 3H); ^{13}C NMR (75 MHz, $CDCl_3$) δ 200.1 (d, $J_{F–C}$ = 26.5 Hz), 147.6, 139.8, 133.2 (d, $J_{F–C}$ = 4.1 Hz), 131.3 (d, $J_{F–C}$ = 8.6 Hz), 129.3, 128.7 (d, $J_{F–C}$ = 1.3 Hz), 118.3, 113.4, 104.0, 101.5, 51.2 (d, $J_{F–C}$ = 21.9 Hz), 22.4 (d, $J_{F–C}$ = 23.4 Hz); ^{19}F NMR (376 MHz, $CDCl_3$) δ −156.2; HRMS (ESI) m/z 292.0906 (M+H$^+$), calc. for $C_{16}H_{15}NOFCl$ 292.0804.

The ee was determined by HPLC analysis: CELLULOSE–1 (4.6 mm i.d. x 250 mm); Hexane/2-propanol = 97/3; flow rate 1.0 mL/min; 25 ^{o}C; 254 nm; retention time: 21.1 min (major) and 23.0 min (minor).

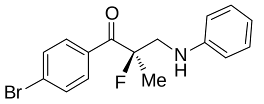

(*R*)-1-(4-bromophenyl)-2-fluoro-2-methyl-3-(phenylamino)propan-1-one (6d): white solid, Mp 37.5−38.0 ^{o}C; 23.2 mg, 69% yield; 86% ee; $[\alpha]_D^{22}$ −111.29 (*c* 1.0, $CHCl_3$); ^{1}H NMR (300 MHz, $CDCl_3$) δ 7.88 (d, J = 7.7 Hz, 2H), 7.58 (d, J = 8.6 Hz, 2H), 7.18 (t, J = 7.9 Hz, 2H), 6.74 (t, J = 7.3 Hz, 1H), 6.67 (d, J = 7.9 Hz, 2H), 4.05 – 3.75 (m, 2H), 3.58 (dd, J = 16.2, 14.1 Hz, 1H), 1.72 (d, J = 21.8 Hz, 3H); ^{13}C NMR (75 MHz, $CDCl_3$) δ 200.3 (d, $J_{F–C}$ = 26.6 Hz), 147.6, 133.7 (d, $J_{F–C}$ = 4.1 Hz), 131.6 (d, $J_{F–C}$ = 1.2 Hz), 131.3 (d, $J_{F–C}$ = 8.6 Hz), 129.2, 128.6, 118.3, 113.3, 102.8 (d, $J_{F–C}$ = 186.9 Hz), 51.2 (d, $J_{F–C}$ = 21.9 Hz), 22.4 (d, $J_{F–C}$ = 23.4 Hz); ^{19}F NMR (376 MHz, $CDCl_3$) δ −156.6; HRMS (ESI) m/z 336.0405 (M+H$^+$), calc. for $C_{16}H_{16}NOFBr$ 336.0399.

The ee was determined by HPLC analysis: CELLULOSE–4 (4.6 mm i.d. x 250 mm); Hexane/2-propanol = 95/5; flow rate 1.0 mL/min; 25 °C; 254 nm; retention time: 7.3 min (minor) and 8.4 min (major).

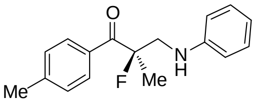

(*R*)-2-fluoro-2-methyl-3-(phenylamino)-1-(p-tolyl)propan-1-one (6e): yellow oil; 19.0 mg, 70% yield; 90% ee; $[\alpha]_D^{22}$ –148.0 (*c* 1.0, $CHCl_3$); ^{1}H NMR (300 MHz, $CDCl_3$) δ 7.95 (d, *J* = 7.5 Hz, 2H), 7.26 – 7.21 (m, 2H), 7.17 (t, *J* = 7.9 Hz, 2H), 6.79 – 6.60 (m, 3H), 3.85 (dd, *J* = 24.2, 13.8 Hz, 1H), 3.59 (dd, *J* = 16.9, 13.9 Hz, 1H), 2.41 (s, 3H), 1.73 (d, *J* = 21.8 Hz, 3H); ^{13}C NMR (75 MHz, $CDCl_3$) δ 200.5 (d, $J_{F\text{–}C}$ = 25.9 Hz), 147.8, 144.2, 132.3 (d, $J_{F\text{–}C}$ = 4.1 Hz), 123.0 (d, $J_{F\text{–}C}$ = 8.2 Hz), 129.2, 129.1 (d, $J_{F\text{–}C}$ = 1.1 Hz), 118.0, 113.3, 102.5 (d, $J_{F\text{–}C}$ = 186.9 Hz), 51.0 (d, $J_{F\text{–}C}$ = 22.2 Hz), 22.5 (d, $J_{F\text{–}C}$ = 23.5 Hz), 21.7; ^{19}F NMR (376 MHz, $CDCl_3$) δ −156.5; HRMS (ESI) m/z 294.1266 (M+Na$^+$), calc. for $C_{17}H_{18}NOFNa$ 294.1270.

The ee was determined by HPLC analysis: CHIRALPAK IE (4.6 mm i.d. x 250 mm); Hexane/2-propanol = 97/3; flow rate 1.0 mL/min; 25 °C; 254 nm; retention time: 10.0 min (major) and 11.3 min (minor).

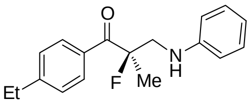

(*R*)-1-(4-ethylphenyl)-2-fluoro-2-methyl-3-(phenylamino)propan-1-one (6f): yellow oil; 20.0 mg, 70% yield; 90% ee; $[\alpha]_D^{22}$ –209.3 (*c* 1.0, $CHCl_3$); ^{1}H NMR (300 MHz, $CDCl_3$) δ 7.98 (d, *J* = 8.1 Hz, 2H), 7.27 (d, *J* = 8.3 Hz, 2H), 7.17 (t, *J* = 7.8 Hz, 2H), 6.78 – 6.63 (m, 3H), 3.85 (dd, *J* = 24.4, 13.8 Hz, 1H), 3.59 (dd, *J* = 16.9, 13.8 Hz, 1H), 2.71 (q, *J* = 7.6 Hz, 2H), 1.74 (d, *J* = 21.8 Hz, 3H), 1.26 (t, *J* = 7.6 Hz, 3H); ^{13}C NMR (75 MHz, $CDCl_3$) δ 200.5 (d, $J_{F\text{–}C}$ = 25.9 Hz), 150.4, 147.8, 132.4 (d, $J_{F\text{–}C}$ = 4.0 Hz), 130.1 (d, $J_{F\text{–}C}$ = 8.2 Hz), 129.2, 127.9 (d, $J_{F\text{–}C}$ = 1.1 Hz), 118.0, 113.3, 102.5 (d, $J_{F\text{–}C}$ = 186.9 Hz), 51.0 (d, $J_{F\text{–}C}$ = 22.1 Hz), 28.9, 22.5 (d, $J_{F\text{–}C}$ = 23.5 Hz), 15.1; ^{19}F NMR (376 MHz, $CDCl_3$) δ −156.2; HRMS (ESI) m/z 286.1601 (M+H$^+$), calc. for $C_{18}H_{21}NOF$ 286.1607.

The ee was determined by HPLC analysis: CELLULOSE–4 (4.6 mm i.d. x 250 mm); Hexane/2-propanol = 95/5; flow rate 1.0 mL/min; 25 °C; 254 nm; retention time: 6.7 min (minor) and 7.3 min (major).

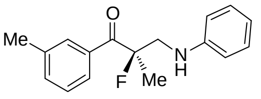

(*R*)-2-fluoro-2-methyl-3-(phenylamino)-1-(m-tolyl)propan-1-one (6g): yellow oil; 18.4 mg, 68% yield; 86% ee; $[\alpha]_D^{22}$ –138.0 (*c* 1.0, $CHCl_3$); ^{1}H NMR (300 MHz, $CDCl_3$) δ 7.88 – 7.77 (m, 2H), 7.44 – 7.28 (m, 2H), 7.18 (t, *J* =

7.9 Hz, 2H), 6.68 (d, J = 7.8 Hz, 1H), 6.68 (d, J = 7.8 Hz, 2H), 3.87 (dd, J = 24.7, 13.8 Hz, 1H), 3.59 (dd, J = 16.6, 13.9 Hz, 1H), 2.39 (s, 3H), 1.74 (d, J = 21.7 Hz, 3H); ^{13}C NMR (75 MHz, $CDCl_3$) δ 201.3 (d, $J_{F–C}$ = 26.3 Hz), 147.7, 138.1, 135.0 (d, $J_{F–C}$ = 3.9 Hz), 134.0, 130.1 (d, $J_{F–C}$ = 6.8 Hz), 129.2, 128.2 (d, $J_{F–C}$ = 1.3 Hz), 126.9 (d, $J_{F–C}$ = 8.9 Hz), 118.1, 113.3, 102.5 (d, $J_{F–C}$ = 187.1 Hz), 51.1 (d, $J_{F–C}$ = 22.1 Hz), 22.5 (d, $J_{F–C}$ = 23.5 Hz), 21.4; ^{19}F NMR (376 MHz, $CDCl_3$) δ −156.4; HRMS (ESI) m/z 272.1453 (M+H$^+$), calc. for $C_{17}H_{19}NOF$ 272.1451.

The ee was determined by HPLC analysis: CHIRALPAK IE (4.6 mm i.d. x 250 mm); Hexane/2-propanol = 98/2; flow rate 1.0 mL/min; 25 °C; 254 nm; retention time: 9.6 min (major) and 10.9 min (minor).

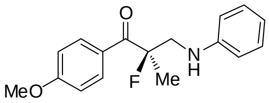

(R)-2-fluoro-1-(4-methoxyphenyl)-2-methyl-3-(phenylamino)propan-1-one (6h): white solid, Mp 67.6−68.0 °C; 20.1 mg, 70% yield; 90% ee; $[\alpha]_D^{22}$ +47.34 (c 1.0, $CHCl_3$); ^{1}H NMR (300 MHz, $CDCl_3$) δ 8.09 (d, J = 8.3 Hz, 2H), 7.17 (t, J = 7.9 Hz, 2H), 6.92 (d, J = 8.9 Hz, 2H), 6.79 – 6.60 (m, 3H), 3.97 – 3.76 (m, 4H), 3.59 (dd, J = 17.1, 13.9 Hz, 1H), 1.73 (d, J = 21.9 Hz, 3H); ^{13}C NMR (75 MHz, $CDCl_3$) δ 198.9 (d, $J_{F–C}$ = 25.2 Hz), 163.7, 147.8, 132.5 (d, $J_{F–C}$ = 9.0 Hz), 129.2, 127.6 (d, $J_{F–C}$ = 4.3 Hz), 118.0, 113.6 (d, $J_{F–C}$ = 1.4 Hz), 113.3, 103.8, 101.4, 55.5, 51.0 (d, $J_{F–C}$ = 22.3 Hz), 22.6 (d, $J_{F–C}$ = 23.5 Hz); ^{19}F NMR (376 MHz, $CDCl_3$) δ −155.6; HRMS (ESI) m/z 288.1390 (M+H$^+$), calc. for $C_{17}H_{18}NO_2F$ 288.1400.

The ee was determined by HPLC analysis: CHIRALPAK IE (4.6 mm i.d. x 250 mm); Hexane/2-propanol = 95/5; flow rate 1.0 mL/min; 25 °C; 254 nm; retention time: 13.7 min (major) and 16.4 min (minor).

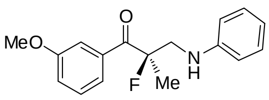

(R)-2-fluoro-1-(3-methoxyphenyl)-2-methyl-3-(phenylamino)propan-1-one (6i): yellow oil; 19.0 mg, 66% yield; 90% ee; $[\alpha]_D^{22}$ –91.3 (c 1.0, $CHCl_3$); ^{1}H NMR (300 MHz, $CDCl_3$) δ 7.64 (d, J = 7.7 Hz, 1H), 7.50 (s, 1H), 7.35 (t, J = 8.0 Hz, 1H), 7.24 – 7.04 (m, 3H), 6.80 – 6.59 (m, 3H), 3.94 – 3.75 (m, 4H), 3.59 (dd, J = 16.3, 14.0 Hz, 1H), 1.74 (d, J = 21.7 Hz, 3H); ^{13}C NMR (75 MHz, $CDCl_3$) δ 200.9 (d, $J_{F–C}$ = 26.2 Hz), 159.4, 147.7, 136.1 (d, $J_{F–C}$ = 4.0 Hz), 129.3 (d, $J_{F–C}$ = 1.5 Hz), 129.2, 122.3 (d, $J_{F–C}$ = 9.8 Hz), 119.8, 118.1, 114.1 (d, $J_{F–C}$ = 6.7 Hz), 102.6 (d, $J_{F–C}$ = 187.4 Hz), 55.4, 51.1 (d,

$J_{F–C}$ = 22.1 Hz), 22.5 (d, $J_{F–C}$ = 23.5 Hz); ^{19}F NMR (376 MHz, $CDCl_3$) δ −156.2; HRMS (ESI) m/z 288.1401 (M+H$^+$), calc. for $C_{17}H_{19}NO_2F$ 288.1400.

The ee was determined by HPLC analysis: CHIRALPAK IE (4.6 mm i.d. x 250 mm); Hexane/2-propanol = 95/5; flow rate 1.0 mL/min; 25 °C; 254 nm; retention time: 10.0 min (major) and 13.0 min (minor).

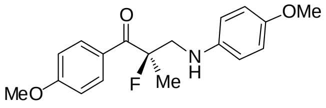

(*R*)-2-fluoro-1-(4-methoxyphenyl)-3-((4-methoxyphenyl)amino)-2-methylpropan-1-one (6j): white solid, Mp 56.6−57.2 °C; 21.6 mg, 68% yield; 87% ee; $[\alpha]_D^{22}$ –291.3 (*c* 1.0, $CHCl_3$); ^{1}H NMR (300 MHz, $CDCl_3$) δ 8.08 (d, *J* = 8.4 Hz, 2H), 6.92 (d, *J* = 9.0 Hz, 2H), 6.81 – 6.70 (m, 2H), 6.63 (d, *J* = 8.9 Hz, 2H), 3.87 (s, 3H), 3.84 – 3.68 (m, 4H), 3.51 (dd, *J* = 16.8, 13.6 Hz, 1H), 1.72 (d, *J* = 21.9 Hz, 3H). ^{13}C NMR (75 MHz, $CDCl_3$) δ 199.1 (d, $J_{F–C}$ = 25.4 Hz), 163.6, 152.5, 142.1, 132.4 (d, $J_{F–C}$ = 9.0 Hz), 127.7 (d, $J_{F–C}$ = 4.2 Hz), 114.8, 114.7, 113.6 (d, $J_{F–C}$ = 1.3 Hz), 103.9, 101.5, 55.8, 55.4, 52.3 (d, $J_{F–C}$ = 22.1 Hz), 22.6 (d, $J_{F–C}$ = 23.6 Hz); ^{19}F NMR (376 MHz, $CDCl_3$) δ −155.7; HRMS (ESI) m/z 340.1335 (M+H$^+$), calc. for $C_{18}H_{20}NO_3FNa$ 340.1325.

The ee was determined by HPLC analysis: CHIRALPAK IE (4.6 mm i.d. x 250 mm); Hexane/2-propanol = 80/20; flow rate 1.0 mL/min; 25 °C; 254 nm; retention time: 13.0 min (major) and 15.3 min (minor).

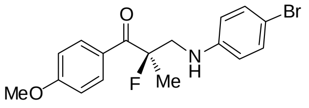

(*R*)-3-((4-bromophenyl)amino)-2-fluoro-1-(4-methoxyphenyl)-2-methylpropan-1-one (6k): white solid, Mp 82.0−82.6 °C; 29.3 mg, 80% yield; 94% ee; $[\alpha]_D^{22}$ –193.9 (*c* 1.0, $CHCl_3$); ^{1}H NMR (300 MHz, $CDCl_3$) δ 8.07 (d, *J* = 8.3 Hz, 2H), 7.23 (d, *J* = 8.8 Hz, 2H), 6.92 (d, *J* = 9.0 Hz, 2H), 6.54 (d, *J* = 8.8 Hz, 2H), 4.02 (s, 1H), 3.91 – 3.72 (m, 4H), 3.61 – 3.46 (m, 1H), 1.71 (d, *J* = 21.8 Hz, 3H). ^{13}C NMR (75 MHz, $CDCl_3$) δ198.7 (d, $J_{F–C}$ = 25.3 Hz), 163.8, 146.8, 132.4 (d, $J_{F–C}$ = 9.0 Hz), 131.8, 127.4 (d, $J_{F–C}$ = 4.2 Hz), 114.8, 113.6 (d, $J_{F–C}$ = 1.4 Hz), 109.5, 102.6 (d, $J_{F–C}$ = 187.3 Hz), 55.5, 50.9 (d, $J_{F–C}$ = 22.3 Hz), 22.6 (d, $J_{F–C}$ = 23.4 Hz); ^{19}F NMR (376 MHz, $CDCl_3$) δ −155.6; HRMS (ESI) m/z 366.0507 (M+H$^+$), calc. for $C_{17}H_{17}NO_2FBr$ 366.0505.

The ee was determined by HPLC analysis: CELLULOSE–4 (4.6 mm i.d. x 250 mm); Hexane/2-propanol = 95/5; flow rate 1.0 mL/min; 25 °C; 254 nm; retention time: 13.3 min (minor) and 18.4 min (major).

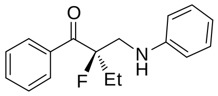

(*R*)-2-fluoro-1-phenyl-2-((phenylamino)methyl)butan-1-one (6l): white solid, Mp 57.8−58.4 °C; 17.6 mg, 65% yield; 80% ee; $[\alpha]_D^{22}$ −161.9 (*c* 1.0, $CHCl_3$); ^1H NMR (300 MHz, $CDCl_3$) δ 7.98 (d, *J* = 8.1 Hz, 2H), 7.55 (t, *J* = 7.4 Hz, 1H), 7.43 (t, *J* = 7.5 Hz, 2H), 7.16 (t, *J* = 7.7 Hz, 2H), 6.73 (t, *J* = 7.3 Hz, 1H), 6.66 (d, *J* = 7.7 Hz, 2H), 3.85 (dd, *J* = 26.6, 13.7 Hz, 1H), 3.64 – 3.50 (m, 1H), 2.34 – 1.94 (m, 2H), 1.02 (t, *J* = 7.5 Hz, 3H); ^{13}C NMR (75 MHz, $CDCl_3$) δ 202.1 (d, $J_{F\text{–}C}$ = 26.7 Hz), 147.7, 136.2 (d, $J_{F\text{–}C}$ = 3.9 Hz), 133.0, 129.5 (d, $J_{F\text{–}C}$ = 8.5 Hz), 129.2, 128.3 (d, $J_{F\text{–}C}$ = 1.1 Hz), 118.1, 113.4, 105.7 (d, $J_{F\text{–}C}$ = 190.1 Hz), 50.3 (d, $J_{F\text{–}C}$ = 22.0 Hz), 29.1 (d, $J_{F\text{–}C}$ = 22.2 Hz), 7.7 (d, $J_{F\text{–}C}$ = 5.0 Hz); ^{19}F NMR (376 MHz, $CDCl_3$) δ −166.5; HRMS (ESI) m/z 272.1459 ($M+H^+$), calc. for $C_{17}H_{18}NOF$ 272.1451.

The ee was determined by HPLC analysis: CHIRALPAK IE (4.6 mm i.d. x 250 mm); Hexane/2-propanol = 97/3; flow rate 1.0 mL/min; 25 °C; 254 nm; retention time: 9.0 min (major) and 10.2 min (minor).

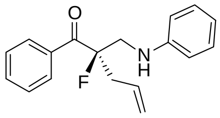

(*R*)-2-fluoro-1-phenyl-2-((phenylamino)methyl)pent-4-en-1-one (6m): white solid, Mp 70.6−70.9 °C; 18.4 mg, 65% yield; 78% ee; $[\alpha]_D^{22}$ +86 (*c* 1.0, $CHCl_3$); ^1H NMR (300 MHz, $CDCl_3$) δ 7.94 (d, *J* = 8.1 Hz, 2H), 7.55 (t, *J* = 7.4 Hz, 1H), 7.42 (t, *J* = 7.6 Hz, 2H), 7.17 (t, *J* = 7.9 Hz, 2H), 6.75 (t, *J* = 7.4 Hz, 1H), 6.69 (d, *J* = 7.9 Hz, 2H), 5.81 (td, *J* = 17.2, 7.3 Hz, 1H), 5.32 – 5.08 (m, 2H), 3.88 (dd, *J* = 26.7, 13.8 Hz, 1H), 3.69 – 3.53 (m, 1H), 3.05 – 2.64 (m, 2H); ^{13}C NMR (75 MHz, $CDCl_3$) δ 201.4 (d, $J_{F\text{–}C}$ = 26.4 Hz), 147.3, 135.9 (d, $J_{F\text{–}C}$ = 4.0 Hz), 133.1, 130.2 (d, $J_{F\text{–}C}$ = 4.6 Hz), 129.6 (d, *J* = 8.3 Hz), 129.2, 128.2 (d, $J_{F\text{–}C}$ = 1.2 Hz), 120.4, 118.5, 113.7, 104.3 (d, $J_{F\text{–}C}$ = 192.5 Hz), 50.4 (d, $J_{F\text{–}C}$ = 21.8 Hz), 40.4 (d, $J_{F\text{–}C}$ = 21.7 Hz); ^{19}F NMR (376 MHz, $CDCl_3$) δ −164.6; HRMS (ESI) m/z 306.1266 ($M+Na^+$), calc. for $C_{18}H_{18}FNNaO$ 306.1265.

The ee was determined by HPLC analysis: CHIRALPAK IF and CHIRALPAK IE (4.6 mm i.d. x 250 mm); Hexane/2-propanol = 97/3; flow rate 1.0 mL/min; 25 °C; 254 nm; retention time: 16.9 min (major) and 18.9 min (minor).

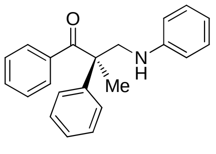

(*R*)-2-methyl-1,2-diphenyl-3-(phenylamino)propan-1-one (8a): yellow oil; 21.8 mg, 69% yield; 90% ee; $[\alpha]_D^{22}$ +309.4 (*c* 1.0, $CHCl_3$); ^1H NMR (300 MHz, $CDCl_3$) δ 7.51 (d, *J* = 7.5 Hz, 2H), 7.46 – 7.28 (m, 6H), 7.23 (t, *J* = 6.0 Hz, 2H), 7.11 (t, *J* = 7.8 Hz, 2H), 6.64 (t, *J* = 7.3 Hz, 1H), 6.56 (d, *J* = 7.9 Hz, 2H),

4.08 (s, 1H), 3.77 (d, *J* = 13.0 Hz, 1H), 3.47 (d, *J* = 12.9 Hz, 1H), 1.78 (s, 3H); ^{13}C NMR (75 MHz, $CDCl_3$) δ 203.3, 148.5, 141.5, 135.8, 132.1, 129.7, 129.2, 129.1, 128.0, 127.5, 126.4, 117.3, 113.0, 56.1, 53.9, 21.4; HRMS (ESI) m/z 338.1519 ($M+Na^+$), calc. for $C_{22}H_{21}NONa$ 338.1521.

The ee was determined by HPLC analysis: CHIRALPAK IE and CHIRALPAK IE (4.6 mm i.d. x 250 mm); Hexane/2-propanol = 97/3; flow rate 1.0 mL/min; 25 °C; 254 nm; retention time: 23.0 min (minor) and 30.1 min (major).

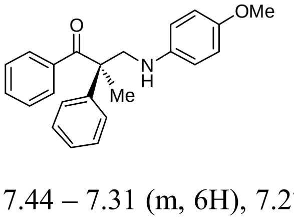

(*R*)-3-((4-methoxyphenyl)amino)-2-methyl-1,2-diphenylpropan-1-one (8b): yellow oil; 24.1 mg, 70% yield; 87% ee; $[\alpha]_D^{22}$ +259.4 (*c* 1.0, $CHCl_3$); ^{1}H NMR (300 MHz, $CDCl_3$) δ 7.55 – 7.47 (m, 2H), 7.44 – 7.31 (m, 6H), 7.27 – 7.20 (m, 2H), 6.71 (dd, *J* = 9.6, 2.7 Hz, 2H), 6.55 (d, *J* = 8.8 Hz, 2H), 3.77 – 3.66 (m, 4H), 3.41 (d, *J* = 12.6 Hz, 1H), 1.80 (s, 3H); ^{13}C NMR (75 MHz, $CDCl_3$) δ 203.3, 152.2, 141.6, 135.9, 132.0, 129.7, 129.2, 128.0, 127.5, 126.5, 114.8, 55. 9, 55.8, 55.4, 21.6; HRMS (ESI) m/z 346.1801 ($M+H^+$), calc. for $C_{23}H_{24}NO_2$ 346.1802.

The ee was determined by HPLC analysis: CHIRALPAK IE (4.6 mm i.d. x 250 mm); Hexane/2-propanol = 70/30; flow rate 1.0 mL/min; 25 °C; 254 nm; retention time: 12.7 min (minor) and 15.9 min (major)

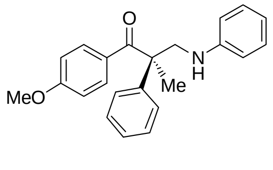

(*R*)-1-(4-methoxyphenyl)-2-methyl-2-phenyl-3-(phenylamino)propan-1-one (8c): yellow oil; 22.8 mg, 66% yield; 92% ee; $[\alpha]_D^{22}$ +41.1 (*c* 1.0, $CHCl_3$); ^{1}H NMR (300 MHz, $CDCl_3$) δ 7.51 (d, *J* = 7.3 Hz, 2H), 7.44 – 7.29 (m, 6H), 7.23 (t, *J* = 7.5 Hz, 2H), 6.72 (d, *J* = 8.9 Hz, 2H), 6.53 (d, *J* = 8.9 Hz, 2H), 3.77 – 3.68 (m, 4H), 3.41 (d, *J* = 12.6 Hz, 1H), 1.79 (s, 3H); ^{13}C NMR (75 MHz, $CDCl_3$) δ 203.3, 152.1, 142.7, 141.7, 135.9, 132.0, 129.7, 129.2, 128.0, 127.5, 126.5, 114.8, 114.5, 56.0, 55.8, 55.2, 21.6; HRMS (ESI) m/z 346.1801 ($M+H^+$), calc. for $C_{23}H_{24}NO_2$ 346.1802.

The ee was determined by HPLC analysis: CHIRALPAK IF (4.6 mm i.d. x 250 mm); Hexane/2-propanol = 97/3; flow rate 1.0 mL/min; 25 °C; 254 nm; retention time: 15.2 min (major) and 16.4 min (minor).

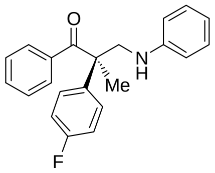

(*R*)-2-(4-fluorophenyl)-2-methyl-1-phenyl-3-(phenylamino)propan-1-one (8d): yellow oil; 22.7 mg, 68% yield; 82% ee; $[\alpha]_D^{22}$ +579.4 (*c* 1.0, $CHCl_3$); ^1H NMR (300 MHz, $CDCl_3$) δ 7.51 (d, *J* = 7.6 Hz, 2H), 7.41 (t, *J* = 7.3 Hz, 1H), 7.37 – 7.27 (m, 3H), 7.25 – 7.19 (m, 1H), 7.14 – 7.03 (m, 4H), 6.66 (t, *J* = 7.2 Hz, 1H), 6.56 (d, *J* = 7.7 Hz, 2H), 3.72 (d, *J* = 13.0 Hz, 1H), 3.46 (d, *J* = 13.1 Hz, 1H), 1.78 (s, 3H); ^{13}C NMR (75 MHz, $CDCl_3$) δ 203.1, 162.1 (d, $J_{F\text{–}C}$ = 247.2 Hz), 137.4 (d, $J_{F\text{–}C}$ = 3.3 Hz), 135.6, 132.3, 129.7, 129.2, 128.2, 128.1, 128.0, 127.8, 117.6, 116.1 (d, $J_{F\text{–}C}$ = 21.3 Hz), 113.2, 55.6, 54.3, 21.6; HRMS (ESI) m/z 356.1427 (M+Na^+), calc. for $C_{22}H_{20}NONaF$ 356.1427.

The ee was determined by HPLC analysis: CHIRALPAK IF and CHIRALPAK IE (4.6 mm i.d. x 250 mm); Hexane/2-propanol = 97/3; flow rate 1.0 mL/min; 25 °C; 254 nm; retention time: 17.1 min (minor) and 23.7 min (major).

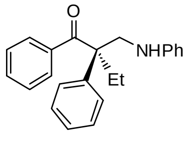

(*R*)-1,2-diphenyl-2-((phenylamino)methyl)butan-1-one (8e): yellow oil; 23.1 mg, 70% yield; 92% ee; $[\alpha]_D^{22}$ +518.8 (*c* 1.0, $CHCl_3$); ^1H NMR (300 MHz, $CDCl_3$) δ 7.47 (d, *J* = 7.6 Hz, 2H), 7.43 – 7.29(m, 6H), 7.22 (t, *J* = 7.7 Hz, 2H), 7.10 (t, *J* = 7.8 Hz, 2H), 6.65 (t, *J* = 7.3 Hz, 1H), 6.52 (d, *J* = 7.9 Hz, 2H), 3.85 (d, *J* = 12.5 Hz, 1H), 3.59 (d, *J* = 12.5 Hz, 1H), 2.46 (td, *J* = 14.9, 7.5 Hz, 1H), 2.26 (td, *J* = 14.3, 7.2 Hz, 1H), 0.79 (t, *J* = 7.4 Hz, 3H); ^{13}C NMR (75 MHz, $CDCl_3$) δ 202.5, 148.3, 141.4, 136.4, 132.0, 129.3, 129.2, 129.1, 128.1, 127.6, 126.9, 117.5, 113.1, 58.9, 48.4, 25.7, 8.6; HRMS (ESI) m/z 352.1678 (M+Na^+), calc. for $C_{23}H_{23}NONa$ 352.1677.

The ee was determined by HPLC analysis: CHIRALPAK IF and CHIRALPAK IE (4.6 mm i.d. x 250 mm); Hexane/2-propanol = 98/2; flow rate 1.0 mL/min; 25 °C; 254 nm; retention time: 38.8 min (minor) and 44.0 min (major).

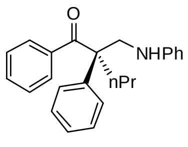

(*R*)-1,2-diphenyl-2-((phenylamino)methyl)pentan-1-one (8f): yellow oil; 22.3 mg, 65% yield; 90% ee; $[\alpha]_D^{22}$ +92.7 (*c* 1.0, $CHCl_3$); ^1H NMR (300 MHz, $CDCl_3$) δ 7.47 (d, *J* = 7.4 Hz, 2H), 7.43 – 7.29 (m, 6H), 7.22 (t, *J* = 7.6 Hz, 2H), 7.10 (t, *J* = 7.8 Hz, 2H), 6.65 (t, *J* = 7.3 Hz, 1H), 6.52 (d, *J* = 7.8 Hz, 2H), 3.83 (d, *J* = 12.6 Hz, 1H), 3.59 (d, *J* = 12.5 Hz, 1H), 2.43 – 2.92 (m, 1H), 2.14 – 2.207 (m, 1H), 1.39 – 1.30 (m, 1H), 1.09 – 0.95 (m, 1H), 0.78 (t, *J* = 7.2 Hz, 3H); ^{13}C NMR (75 MHz, $CDCl_3$) δ 202.5, 148.4, 141.5, 136.3, 132.0, 129.3, 129.2, 129.1, 128.1, 127.5, 126.8, 117.4, 113.1,

58.7, 49.1, 35.3, 17.4, 14.6; HRMS (ESI) m/z 366.1830 (M+Na$^+$), calc. for $C_{24}H_{25}NONa$ 366.834.

The ee was determined by HPLC analysis: CHIRALPAK IF and CHIRALPAK IE (4.6 mm i.d. x 250 mm); Hexane/2-propanol = 97/3; flow rate 0.8 mL/min; 25 °C; 254 nm; retention time: 20.8 min (major) and 21.8 min (minor).

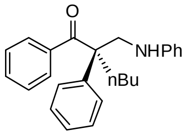

(*R*)-1,2-diphenyl-2-((phenylamino)methyl)hexan-1-one (8g): yellow oil; 21.8 mg, 61% yield; 85% ee; $[\alpha]_D^{22}$ +356.7 (*c* 1.0, $CHCl_3$); ^{1}H NMR (300 MHz, $CDCl_3$) δ 7.47 (d, *J* = 7.7 Hz, 2H), 7.43 – 7.29 (m, 6H), 7.22 (t, *J* = 7.7 Hz, 2H), 7.10 (t, *J* = 7.8 Hz, 2H), 6.65 (t, *J* = 7.2 Hz, 1H), 6.52 (d, *J* = 8.1 Hz, 2H), 3.83 (d, *J* = 12.4 Hz, 1H), 3.59 (d, *J* = 12.2 Hz, 1H), 2.44 – 2.30 (m, 1H), 2.25 – 2.11 (m, 1H), 1.22 – 1.04 (m, 2H), 1.02 – 0.77 (m, 2H), 0.71 (t, *J* = 7.2 Hz, 3H); ^{13}C NMR (75 MHz, $CDCl_3$) δ 202.6, 141.5, 136.4, 132.0, 129.3, 129.2, 129.1, 128.0, 127.6, 126.9, 117.5, 113.2, 58.6, 49.2, 32.7, 26.2, 23.1, 13.7; HRMS (ESI) m/z 380.1993 (M+Na$^+$), calc. for $C_{25}H_{27}NONa$ 380.1990

The ee was determined by HPLC analysis: CHIRALPAK IF and CHIRALPAK IE (4.6 mm i.d. x 250 mm); Hexane/2-propanol = 95/5; flow rate 1.0 mL/min; 25 °C; 254 nm; retention time: 17.6 min (major) and 19.2 min (minor).

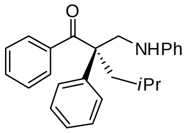

(*R*)-4-methyl-1,2-diphenyl-2-((phenylamino)methyl)pentan-1-one (8h): yellow oil; 21.4 mg, 60% yield; 86% ee; $[\alpha]_D^{22}$ +208.0 (*c* 1.0, $CHCl_3$); ^{1}H NMR (300 MHz, $CDCl_3$) δ 7.46 (d, *J* = 7.6 Hz, 2H), 7.41 – 7.28 (m, 6H), 7.25 – 7.17 (m, 2H), 7.09 (t, *J* = 7.7 Hz, 2H), 6.64 (t, *J* = 7.1 Hz, 1H), 6.50 (d, *J* = 7.9 Hz, 2H), 3.84 (d, *J* = 12.0 Hz, 1H), 3.65 (d, *J* = 12.2 Hz, 1H), 2.41 (dd, *J* = 14.3, 5.6 Hz, 1H), 2.18 (dd, *J* = 14.3, 5.3 Hz, 1H), 1.64 – 1.49 (m, 1H), 0.82 (d, *J* = 6.6 Hz, 3H), 0.59 (d, *J* = 6.6 Hz, 3H); ^{13}C NMR (75 MHz, $CDCl_3$) δ 202.8, 148.3, 141.5, 136.7, 131.9, 129.5, 129.2, 129.0, 128.0, 127.6, 126.9, 117.4, 113.2, 58.6, 49.3, 41.6, 24.9, 24.4, 24.3; HRMS (ESI) m/z 304.1671 (M+Na$^+$), calc. for $C_{19}H_{23}NNaO$ 304.1672.

The ee was determined by HPLC analysis: CHIRALPAK IF and CHIRALPAK IE (4.6 mm i.d. x 250 mm); Hexane/2-propanol = 97/3; flow rate 1.0 mL/min; 25 °C; 254 nm; retention time: 15.0 min (major) and 16.4 min (minor).

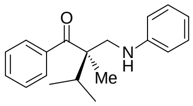

(*R*)-2,3-dimethyl-1-phenyl-2-((phenylamino)methyl)butan-1-one (8i): yellow oil; 17.7 mg, 63% yield; 84% ee; $[\alpha]^{22}_{D}$ –42.0 (*c* 1.0, $CHCl_3$); ^{1}H NMR (300 MHz, $CDCl_3$) δ 7.64 – 7.56 (m, 2H), 7.50 – 7.34 (m, 3H), 7.19 (t, *J* = 7.9 Hz, 2H), 6.73 (t, *J* = 7.3 Hz, 1H), 6.63 (d, *J* = 7.8 Hz, 2H), 3.90 (s, 1H), 3.63 (d, *J* = 12.0 Hz, 1H), 3.24 (d, *J* = 12.0 Hz, 1H), 2.57 – 2.37 (m, 1H), 1.32 (s, 3H), 0.98 (d, *J* = 6.9 Hz, 3H), 0.94 (d, *J* = 6.8 Hz, 3H); ^{13}C NMR (75 MHz, $CDCl_3$) δ 210.1, 148.3, 139.9, 130.6, 129.2, 128.2, 127.0, 117.6, 113.0, 55.6, 50.1, 32.7, 18.2, 17.3, 16.5; HRMS (ESI) m/z 363.1353 ($M+H^+$), calc. for $C_{21}H_{19}N_2O_4$ 363.1345. The ee was determined by HPLC analysis: CELLULOSE-3 (4.6 mm i.d. x 250 mm); Hexane/2-propanol = 95/5; flow rate 1.0 mL/min; 25 °C; 254 nm; retention time: 17.0 min (minor) and 21.2 min (major).

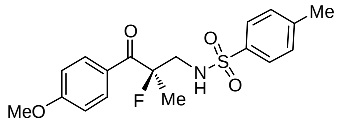

(*R*)-N-(2-fluoro-3-(4-methoxyphenyl)-2-methyl-3-oxopropyl)-4-methylbenzenesulfonamide (10): white solid, Mp 114.8−115.8 °C; 52.6 mg, 72% yield; 87% ee; $[\alpha]^{22}_{D}$ +156.3 (*c* 1.0, $CHCl_3$); ^{1}H NMR (300 MHz, $CDCl_3$) δ 8.01 (d, *J* = 8.5 Hz, 2H), 7.72 (d, *J* = 8.1 Hz, 2H), 7.27 (d, *J* = 7.8 Hz, 2H), 6.90 (d, *J* = 8.9 Hz, 2H), 5.03 (t, *J* = 6.7 Hz, 1H), 3.87 (s, 3H), 3.54 – 3.37 (m, 2H), 2.41 (s, 3H), 1.68 (d, *J* = 21.8 Hz, 3H); ^{13}C NMR (75 MHz, $CDCl_3$) δ 197.4 (d, $J_{F\text{–}C}$ = 24.5 Hz), 164.0, 143.5, 136.8, 132.5 (d, $J_{F\text{–}C}$ = 8.5 Hz), 129.7, 127.0, 126.4 (d, $J_{F\text{–}C}$ = 4.4 Hz), 113.7 (d, $J_{F\text{–}C}$ = 1.2 Hz), 100.2 (d, $J_{F\text{–}C}$ = 187.9 Hz), 55.5, 49.1 (d, $J_{F\text{–}C}$ = 25.1 Hz), 22.4 (d, $J_{F\text{–}C}$ = 22.9 Hz), 21.5; ^{19}F NMR (376 MHz, $CDCl_3$) δ −154.6; HRMS (ESI) m/z 388.0996 ($M+Na^+$), calc. for $C_{18}H_{20}NO_4FNaS$ 388.0995.

The ee was determined by HPLC analysis: CHIRALPAK IE (4.6 mm i.d. x 250 mm); Hexane/2-propanol = 40/60; flow rate 1.0 mL/min; 25 °C; 254 nm; retention time: 18.2 min (major) and 44.0 min (minor).

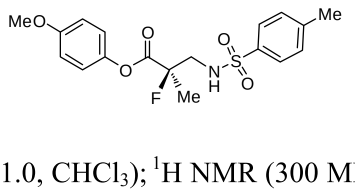

(*R*)-4-methoxyphenyl 2-fluoro-2-methyl-3-(4-methyl-phenylsulfonamido)propanoate (11): white solid, Mp 108.8−109.8 °C; 26.7 mg, 70% yield; 87% ee; $[\alpha]^{22}_{D}$ +207.3 (*c* 1.0, $CHCl_3$); ^{1}H NMR (300 MHz, $CDCl_3$) δ 8.08 (d, *J* = 8.4 Hz, 2H), 6.92 (d, *J* = 9.0 Hz, 2H), 6.76 (d, *J* = 8.9 Hz, 2H), 6.63 (d, *J* = 8.9 Hz, 2H), 3.87 (s, 3H), 3.82 – 3.68 (m, 4H), 3.51 (dd, *J* = 16.8, 13.6 Hz, 1H), 1.72 (d, *J* = 21.9 Hz, 3H); ^{13}C NMR (75 MHz, $CDCl_3$) δ 199.1 (d, $J_{F\text{–}C}$ = 25.4 Hz), 163.6, 152.5, 142.1, 132.4 (d, $J_{F\text{–}C}$ = 9.0 Hz), 127.7 (d, $J_{F\text{–}C}$ = 4.2 Hz), 114.8,

114.7, 113.6 (d, $J_{F\text{–}C}$ = 1.3 Hz), 102.7 (d, $J_{F\text{–}C}$ = 186.6 Hz), 55.8, 55.4, 52.3 (d, $J_{F\text{–}C}$ = 22.1 Hz), 22.6 (d, $J_{F\text{–}C}$ = 23.6 Hz); ^{19}F NMR (376 MHz, $CDCl_3$) δ −159.6; HRMS (ESI) m/z 404.0952 (M+Na^+), calc. for $C_{18}H_{20}NO_5FNaS$ 404.0944.

The ee was determined by HPLC analysis: CHIRALPAK IB (4.6 mm i.d. x 250 mm); Hexane/2-propanol = 75/25; flow rate 1.0 mL/min; 25 °C; 254 nm; retention time: 12.9 min (minor) and 20.3 min (major).

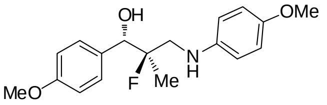

(1*S*,2*R*)-2-fluoro-1-(4-methoxyphenyl)-3-((4-methoxyphenyl)amino)-2-methylpropan-1-ol (12): white solid, Mp 55.1−55.7 °C; 25.9 mg, 81% yield; 87% ee; >20:1 dr; $[\alpha]_D^{22}$ +207.4 (*c* 1.0, $CHCl_3$); ^{1}H NMR (300 MHz, $CDCl_3$) δ 7.33 (d, *J* = 8.5 Hz, 2H), 6.89 (d, *J* = 8.7 Hz, 2H), 6.79 (d, *J* = 8.9 Hz, 2H), 6.67 (d, *J* = 8.9 Hz, 2H), 4.95 (d, *J* = 9.0 Hz, 1H), 3.82 (s, 3H), 3.75 (s, 3H), 3.60 – 3.47 (m, 1H), 3.24 (dd, *J* = 17.7, 13.3 Hz, 1H), 1.28 (d, *J* = 22.6 Hz, 3H); ^{13}C NMR (75 MHz, $CDCl_3$) δ 159.3, 152.7, 141.8, 131.3 (d, $J_{F\text{–}C}$ = 3.2 Hz), 128.4 (d, $J_{F\text{–}C}$ = 1.1 Hz), 115.1, 114.8, 113.5, 98.1 (d, $J_{F\text{–}C}$ = 173.4 Hz), 76.5 (d, $J_{F\text{–}C}$ = 26.6 Hz), 55.7, 55.2, 50.3 (d, $J_{F\text{–}C}$ = 21.5 Hz), 19.5 (d, $J_{F\text{–}C}$ = 23.0 Hz); ^{19}F NMR (376 MHz, $CDCl_3$) δ −158.8; HRMS (ESI) m/z 342.475 (M+Na^+), calc. for $C_{18}H_{22}NO_3FNa$ 342.1481.

The ee was determined by HPLC analysis: CHIRALPAK IG (4.6 mm i.d. x 250 mm); Hexane/2-propanol = 90/10; flow rate 1.0 mL/min; 25 °C; 254 nm; retention time: 32.0 min (minor) and 34.2 min (major).

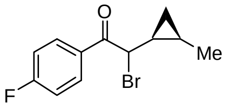

2-bromo-1-(4-fluorophenyl)-2-((1*S*,2*R*/1R,2*S*)-2-methylcyclopropyl)ethanone (13); yellow oil; 810.0 mg, 75% yield; ^{1}H NMR (300 MHz, $CDCl_3$) δ 8.11 – 7.95 (m, 2H), 7.21 – 7.07 (m, 2H), 4.80 – 4.64 (m, 0.25H), 4.48 – 4.41 (m, 0.77H), 1.91 – 1.77 (m, 0.33H), 1.60 – 1.47 (m, 0.80 H), 1.25 – 0.92 (m, 4H), 0.90 – 0.54 (m, 2H); ^{13}C NMR (75 MHz, $CDCl_3$) δ 191.4, 190.9, 190.8, 190.7, 167.6, 164.2, 131.8, 131.7, 131.6, 131.5, 130.6, 130.5, 116.1, 116.0, 115.8, 115.7, 53.7, 53.5, 50.0, 49.4, 22.8, 22.4, 19.6, 18.7, 18.2, 18.0, 17.5, 16.0, 15.5, 15.1, 14.9, 14.7, 13.5, 12.8, 11.7; HRMS (ESI) m/z 271.0132 (M+H^+), calc. for $C_{12}H_{13}BrFO$ 271.0134.

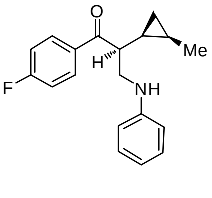

(*S*)-1-(4-fluorophenyl)-2-((1*S*,2*R*/1*R*,2*S*)-2-methylcyclopropyl)-3-(phenylamino)propan-1-one (14); yellow oil; 20.8 mg, 70% yield; $[\alpha]_D^{22}$

–89.2 (*c* 1.0, $CHCl_3$); ^{1}H NMR (300 MHz, $CDCl_3$) δ 7.947.74 (m, 2H), 7.24 – 6.90 (m, 4H), 6.73 (t, *J* = 7.1 Hz, 1H), 6.61 (d, *J* = 8.1 Hz, 2H), 3.78 – 3.60 (m, 1H), 3.52 – 3.38 (m, 1H), 3.24 – 3.05 (m, 1H), 1.10 – 0.84 (m, 3H), 0.77 – 0.54 (m, 2H), 0.52 – 0.17 (m, 2H); ^{13}C NMR (75 MHz, $CDCl_3$) δ 202.9, 201.7, 201.4, 167.5, 167.4, 164.1, 164.0, 147.5, 147.4, 140.0, 133.9, 133.6, 131.0, 130.9, 130.8, 130.7, 129.4, 129.3, 117.7, 117.6, 115.8, 115.5, 112.9, 112.8, 49.4, 49.3, 47.5, 46.0, 45.8, 45.4, 44.8, 20.6, 20.5, 18.8, 18.4, 17.2, 14.4, 14.1, 12.6, 12.5, 12.1, 12.0, 11.8, 10.0, 9.0; HRMS (ESI) m/z 298.1605 (M+H$^+$), calc. for $C_{19}H_{21}FNO$ 298.1607.

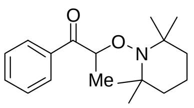

1-phenyl-2-((2,2,6,6-tetramethylpiperidin-1-yl)oxy)propan-1-one (15): yellow oil; 15.9 mg, 55% yield; ^{1}H NMR (300 MHz, $CDCl_3$) δ 8.08 (d, *J* = 7.2 Hz, 2H), 7.56 (t, *J* = 7.2 Hz, 1H), 7.46 (t, *J* = 7.5 Hz, 2H), 5.01 (q, *J* = 7.1 Hz, 1H), 1.61 (s, 3H), 1.51 (d, *J* = 7.1 Hz, 3H), 1.36 – 1.23 (m, 6H), 1.18 (s, 3H), 1.03 (s, 3H), 0.87 (s, 3H); ^{13}C NMR (75 MHz, $CDCl_3$) δ 201.9, 135.2, 133.0, 129.3, 128.5, 86.4, 59.7, 40.3, 20.3, 19.3, 17.1; HRMS (ESI) m/z 312.1942 (M+Na$^+$), calc. for $C_{18}H_{27}NO_2Na$ 312.1939.

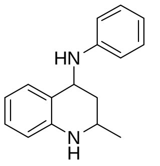

2-methyl-N-phenyl-1,2,3,4-tetrahydroquinolin-4-amine (16): colorless oil; 4.8 mg, 20 % yield; ^{1}H NMR (300 MHz, $CDCl_3$) δ 7.32 – 7.04 (m, 4H), 6.81 – 6.50 (m, 5H), 4.56 (s, brs, 1H), 3.88 (br, *NH*, 2H), 3.54 – 3.36 (m, 1H), 2.23 – 2.18 (m, 1H), 1.60 – 1.52 (m, 1H), 1.23 (d, *J* = 6.3 Hz, 3H); ^{13}C NMR (75 MHz, $CDCl_3$) δ 146.4, 144.9, 130.7, 129.3, 128.5, 121.1, 117.3, 117.03, 114.5, 112.6, 48.8, 42.3, 35.0, 21.9; HRMS (ESI) m/z 239.1540 (M+H$^+$), calc. for $C_{16}H_{19}N_2$ 239.1548.

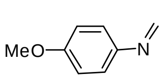

4-methoxy-*N*-methyleneaniline (17): white solid, Mp 126.8−127.7 °C; 108.2 mg, 80% yield ^{1}H NMR (300 MHz, CD_3CN) δ 7.01 (d, *J* = 9.0 Hz, 1H), 6.78 (d, *J* = 9.0 Hz, 1H), 4.67 (s, 2H), 3.70 (s, 3H); ^{13}C NMR (75 MHz, CD_3CN) δ 155.2, 143.7, 120.6, 115.2, 71.2, 56.0.

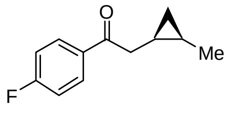

1-(4-fluorophenyl)-2-((1*R*,2*R*/1*S*,2*S*)-2-methylcyclopropyl)ethanone (21): white solid; 768.4 mg, 80% yield; ^{1}H NMR (300 MHz, $CDCl_3$) δ 8.02 – 7.92 (m, 2H), 7.17 – 7.07 (m, 2H), 2.93 (dd, *J* = 10.4, 7.0 Hz, 0.5H), 2.85 (dd, *J* = 6.7, 4.1 Hz, 1.5H), 1.09 – 1.02 (m, 3H), 0.86 – 0.75 (m, 1H), 0.67 – 0.47 (m, 1H), 0.45 – 0.18 (m, 2H); ^{13}C NMR (75 MHz, $CDCl_3$) δ 198.8, 198.4, 167.3, 163.9, 133.4, 133.3, 130.8, 130.7,

130.6, 130.5, 115.7, 115.5, 43.3, 37.9, 18.6, 15.1, 13.6, 13.0, 12.2, 11.2, 9.3; HRMS (ESI) m/z 193.1032 (M+H$^+$), calc. for $C_{12}H_{14}FO$ 193.1029.

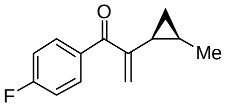

1-(4-fluorophenyl)-2-((1*S*,2*R*/1*R*,2*S*)-2-methylcyclopropyl)prop-2-en-1-one (22); colourless oil; 21.6 mg, 45% yield; ^{1}H NMR (300 MHz, $CDCl_3$) δ 7.87 – 7.75 (m, 2H), 7.11 (t, *J* = 8.6 Hz, 2H), 5.69 – 5.60 (M, 0.50H), 5.46 – 5.28 (M, 1.55H), 1.60 – 1.44 (m, 1H), 1.16 – 0.91 (m, 4H), 0.85 – 0.74 (m, 1H), 0.71 – 0.55 (m, 1H); ^{13}C NMR (75 MHz, $CDCl_3$) δ 196.8, 167.0, 163.7, 149.9, 146.0, 133.7, 133.6, 132.3, 132.2, 132.1, 132.0, 124.8, 118.9, 115.5, 115.2, 21.1, 18.8, 17.2, 16.2, 13.0, 12.9, 10.5; HRMS (ESI) m/z 205.1024 (M+H$^+$), calc. for $C_{19}H_{21}FNO$ 205.1029.

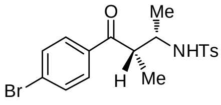

***N*-((2*S*,3*S*)-4-(4-bromophenyl)-3-methyl-4-oxobutan-2-yl)-4-methylbenzenesulfonamide (25)**: white solid, Mp 123.6−124.7 °C; 61.5 mg, 75% yield; 95% ee; >20:1 dr; $[\alpha]_D^{22}$ –156.0 (*c* 1.0, $CHCl_3$); ^{1}H NMR (300 MHz, $CDCl_3$) δ 7.81 – 7.70 (m, 4H), 7.59 (d, *J* = 8.5 Hz, 2H), 7.27 (d, *J* = 7.7 Hz, 2H), 5.54 (d, *J* = 8.7 Hz, 1H), 3.75 – 3.51 (m, 2H), 2.41 (s, 3H), 1.15 (d, *J* = 6.9 Hz, 3H), 0.98 (d, *J* = 6.7 Hz, 3H); ^{13}C NMR (75 MHz, $CDCl_3$) δ 202.2, 143.2, 138.3, 134.8, 132.1, 129.9, 129.6, 128.7, 126.9, 51.8, 45.2, 21.5, 18.8, 13.9; HRMS (ESI) m/z 432.0259 (M+Na$^+$), calc. for $C_{18}H_{20}NO_3NaSBr$ 432.0245.

The ee was determined by HPLC analysis: CHIRALPAK IG (4.6 mm i.d. x 250 mm); Hexane/2-propanol = 85/15; flow rate 1.0 mL/min; 25 °C; 254 nm; retention time: 30.5 min (minor) and 33.1 min (major).

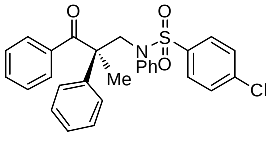

(*R*)-4-chloro-N-(2-methyl-3-oxo-2,3-diphenylpropyl)-N-phenylbenzenesulfonamide (26): white solid, Mp 104.6−105.7 °C; 38.1 mg, 78% yield; 90% ee; $[\alpha]_D^{22}$ +95.4 (*c* 1.0, $CHCl_3$); ^{1}H NMR (300 MHz, $CDCl_3$) δ 7.72 – 7.36 (m, 4H), 7.35 – 7.27 (m, 2H), 7.25 – 7.22 (m, 1H), 7.20 – 7.05 (m, 7H), 7.05 – 6.95 (m, 3H), 6.67 (d, *J* = 7.0 Hz, 2H), 4.45 (d, *J* = 14.3 Hz, 1H), 4.22 (d, *J* = 14.3 Hz, 1H), 1.91 (s, 3H). ^{13}C NMR (75 MHz, $CDCl_3$) δ 202.6, 139.9, 139.1, 136.4, 136.2, 131.8, 129.2, 129.1, 129.0, 128.9, 128.3, 127.9, 127.3, 127.2, 126.9, 59.3, 55.1, 21.4; HRMS (ESI) m/z 512.1075 (M+Na$^+$), calc. for $C_{28}H_{24}NO_3NaSCl$ 512.1063.

The ee was determined by HPLC analysis: CHIRALPAK IG (4.6 mm i.d. x 250 mm); Hexane/2-propanol = 75/25; flow rate 1.0 mL/min; 25 °C; 254 nm; retention time: 16.5 min (major) and 25.9 min (minor).
